# Supplementary material for: Excess years of life lost to COVID-19 and other causes of death by sex, neighbourhood deprivation, and region in England and Wales during 2020: A registry-based study
Source: PLoS Med. 2022 Feb 15;19(2):e1003904. doi: 10.1371/journal.pmed.1003904 (PMC8846534; doi:10.1371/journal.pmed.1003904)

Supplementary file to "Excess years of life lost to COVID-19 and other causes of death by sex, neighbourhood deprivation and region in England & Wales during 2020"

October 6, 2021

Contents

1 Notes 4

2 Tables 5

3 Tables 9

4 Tables 10

5 Total 11

5.1 AASMRs . . . 11

5.2 YLLs . . . 16

5.2.1 England-Wales aggregate . . . 16

5.2.2 By sex . . . 19

5.2.3 By deprivation quintile . . . 24

5.2.4 By Strategic Health Authority . . . 29

5.2.5 By deprivation quintile & Strategic Health Authority . . . 34

5.3 YLLs per $100,000$ population . . . 41

5.3.1 England-Wales aggregate . . . 41

5.3.2 By sex . . . 44

5.3.3 By deprivation quintile . . . 49

5.3.4 By Strategic Health Authority . . . 54

5.3.5 By deprivation quintile & Strategic Health Authority . . . 59

6 Direct 66

6.1 AASMRs . . . 66

6.2 YLLs . . . 71

6.2.1 England-Wales aggregate . . . 71

6.2.2 By sex . . . 74

6.2.3 By deprivation quintile . . . 79
6.2.4 By Strategic Health Authority . . . 84
6.2.5 By deprivation quintile & Strategic Health Authority . . . 89
6.3 YLLs per 100,000 population . . . 96
6.3.1 England-Wales aggregate . . . 96
6.3.2 By sex . . . 99
6.3.3 By deprivation quintile . . . 104
6.3.4 By Strategic Health Authority . . . 109
6.3.5 By deprivation quintile & Strategic Health Authority . . . 114

7 Cardiovascular & diabetes 121
7.1 AASMRs . . . 121
7.2 YLLs . . . 126
7.2.1 England-Wales aggregate . . . 126
7.2.2 By sex . . . 131
7.2.3 By deprivation quintile . . . 136
7.2.4 By Strategic Health Authority . . . 141
7.3 YLLs per 100,000 population . . . 145
7.3.1 England-Wales aggregate . . . 145
7.3.2 By sex . . . 150
7.3.3 By deprivation quintile . . . 155
7.3.4 By Strategic Health Authority . . . 160

8 Cancer 165
8.1 AASMRs . . . 165
8.2 YLLs . . . 169
8.2.1 England-Wales aggregate . . . 169
8.2.2 By sex . . . 174
8.2.3 By deprivation quintile . . . 179
8.2.4 By Strategic Health Authority . . . 184
8.3 YLLs per 100,000 population . . . 188
8.3.1 England-Wales aggregate . . . 188
8.3.2 By sex . . . 193
8.3.3 By deprivation quintile . . . 198
8.3.4 By Strategic Health Authority . . . 203

9 All other indirect 208
9.1 AASMRs . . . 208
9.2 YLLs . . . 213
9.2.1 England-Wales aggregate . . . 213
9.2.2 By sex . . . 218
9.2.3 By deprivation quintile . . . 223
9.2.4 By Strategic Health Authority . . . 228
9.3 YLLs per 100,000 population . . . 232
9.3.1 England-Wales aggregate . . . 232
9.3.2 By sex . . . 237

9.3.3 By deprivation quintile 242
9.3.4 By Strategic Health Authority 247

1 Notes

On years of life lost (YLLs) and age-standardised mortality rates (ASMRs):

1. YLLs calculated against 2019 life expectancy, which was 79.4 for males and 83.1 for females
2. For each death, potential years of life lost (YLL) were calculated using 2019 single year sex-specific life tables for England & Wales
3. Linear regression models using data from Jan 2015 are used to estimate excess YLLs following the start of the pandemic, in a monthly time series
4. ASMRs are age-standardised using 5-year band (plus 90 or over) extrapolated estimates from the 2011 census (available from the ONS), and the 2020-2025 WHO reference population (see this WHO link)
5. ASMRs are mortality rates per $100,000$ population
6. ASMRs are reported separately for males and females (And hence no ASMRs figures are reported under 'by sex' subsections)
7. YLLs and ASMRs are available for these groups of deaths:
   - Direct (COVID-19 identified or suspected, or respiratory)
   - Respiratory only (not reported in this file, to save some space)
   - Cardiovascular & diabetes
   - Cancer
   - All other indirect (including a few cases where no underlying cause of death, or preliminary)
   - Total (i.e. direct, cardiovascular & diabetes, cancer, and all other indirect)
8. Sums of subgroups not necessarily adding up to England-Wales aggregate (especially for deprivation when residence postcode is not available or missing)
9. Cardiovascular excludes alcoholic cardiomyopathy, which is included in all other indirect
10. Other groupings are available but not reported here, for example deprivation by region

2 Tables

Table A: YLL denominators by group for weeks 11-52 (week ending 13 Mar to week ending 25 Dec 2020)

	**Direct**		**Indirect**			
	COVID or respiratory	Respiratory only	Cardio-vascular & diabetes	Neoplasm	Other indirect deaths	Any cause
England	1173914	449854	1231248	1674052	1781211	5860425
Sex						
male	648478	233544	723654	836099	946400	3154631
female	525435	216310	507594	837953	834811	2705794
Regions						
North East	64755	25023	63946	90602	104999	324303
North West	199250	72870	171953	226771	253822	851796
Yorkshire & Humber	125860	47392	123552	166875	178431	594719
East Midlands	97674	37866	106935	145066	148402	498077
West Midlands	131248	48048	134615	172769	197101	635733
East of England	105300	44205	119013	171563	178251	574128
London	161599	43268	134467	169811	191390	657267
South East Coast	80225	34465	92515	135823	141547	450110
South Central	61527	26541	78500	114260	112393	366680
South West	73469	39666	120054	172764	168367	534655
Wales	71080	29976	82150	104389	101881	359500
Deprivation quintiles						
1 (least deprived)	160413	58428	187051	307000	258954	913418
2	195872	74186	214563	327606	304527	1042569
3	217610	84146	243931	334083	342801	1138425
4	262227	97946	266398	337917	384693	1251235
5 (most deprived)	335866	134613	315758	364088	485610	1501323

[a] Using single ICD-10 code reported in underlying cause field (mutually exclusive)
[b] Sums of subgroups not necessarily adding up to England-Wales aggregate
[c] Direct deaths: where underlying cause was COVID (confirmed or suspected) or respiratory

Table B: Percentage of estimated excess YLL over the total of YLLs (and 95% CI) for weeks 11-52 (week ending 13 Mar to week ending 25 Dec 2020)

	**Direct**		**Indirect**			
	COVID or respiratory	Respiratory only	Cardio-vascular & diabetes	Neoplasm	Other indirect deaths	Any cause
England	55(54,56)	-17(-20,-14)	5(4,7)	-1(-2,0)	4(1,6)	13(12,14)
Sex						
male	60(59,61)	-12(-15,-9)	7(6,8)	-1(-3,-0)	4(1,6)	15(14,16)
female	49(48,51)	-23(-26,-20)	3(1,5)	-1(-2,1)	4(2,6)	11(10,12)
Regions						
North East	55(54,57)	-15(-20,-11)	7(5,9)	-1(-2,1)	12(10,14)	16(15,17)
North West	56(55,58)	-20(-23,-16)	4(2,5)	-1(-3,0)	2(0,4)	14(13,15)
Yorkshire & Humber	56(55,57)	-17(-20,-14)	3(1,4)	0(-1,2)	4(1,7)	14(13,14)
East Midlands	55(54,56)	-16(-20,-13)	8(6,9)	1(-1,2)	4(1,8)	14(13,15)
West Midlands	58(57,59)	-15(-18,-12)	9(7,10)	-0(-1,1)	5(3,6)	15(14,16)
East of England	50(48,51)	-20(-23,-17)	2(0,3)	-4(-6,-2)	4(0,8)	10(8,11)
London	70(69,71)	-12(-15,-9)	7(6,9)	-2(-3,-1)	4(1,6)	19(18,20)
South East Coast	50(48,51)	-17(-20,-13)	6(4,7)	-2(-4,-1)	8(6,11)	12(11,13)
South Central	53(51,55)	-10(-15,-5)	8(6,10)	-1(-3,0)	2(-1,6)	11(9,13)
South West	34(32,36)	-22(-26,-18)	4(2,6)	1(-1,2)	2(-0,4)	7(5,8)
Wales	49(48,51)	-20(-24,-17)	8(6,10)	1(-1,3)	-1(-3,2)	12(11,13)
Deprivation quintiles						
1 (least deprived)	58(56,59)	-16(-19,-12)	6(5,8)	1(-1,2)	5(1,8)	13(12,14)
2	56(55,57)	-16(-20,-12)	3(1,5)	-1(-3,0)	5(2,7)	12(11,13)
3	54(53,55)	-19(-22,-16)	5(4,7)	-2(-3,-1)	4(2,7)	12(11,13)
4	55(54,56)	-20(-23,-17)	6(4,7)	-1(-2,0)	3(2,5)	13(12,15)
5 (most deprived)	54(53,55)	-15(-18,-12)	7(6,8)	-0(-2,1)	4(2,5)	15(14,16)

[a] Using single ICD-10 code reported in underlying cause field (mutually exclusive)
[b] Sums of subgroups not necessarily adding up to England-Wales aggregate
[c] Direct deaths: where underlying cause was COVID (confirmed or suspected) or respiratory

Table C: YLL denominators by group for weeks 11-52 in 2019

	**Direct**		**Indirect**			
	COVID or respiratory	Respiratory only	Cardio-vascular & diabetes	Neoplasm	Other indirect deaths	Any cause
England	525747	525747	1146098	1669307	1734200	5075352
Sex						
male	265988	265988	665188	834610	933391	2699178
female	259758	259758	480910	834697	800809	2376174
Regions						
North East	31466	31466	60709	91497	94984	278656
North West	85058	85058	162036	228128	247841	723065
Yorkshire & Humber	54909	54909	115093	162543	178288	510833
East Midlands	44231	44231	98092	144400	146067	432790
West Midlands	55149	55149	118099	169146	185740	528133
East of England	51182	51182	115136	173473	172102	511893
London	46769	46769	124503	170577	186141	527991
South East Coast	40294	40294	84757	134269	133940	393260
South Central	31289	31289	72630	116695	112621	333235
South West	47225	47225	111190	167657	163310	489382
Wales	37016	37016	76188	104899	104532	322635
Deprivation quintiles						
1 (least deprived)	72092	72092	173528	302562	256129	804312
2	89389	89389	203652	330742	294091	917874
3	100110	100110	225879	338356	338398	1002744
4	114623	114623	249117	333735	373880	1071355
5 (most deprived)	148373	148373	286257	357890	463068	1255588

[a] Using single ICD-10 code reported in underlying cause field (mutually exclusive)
[b] Sums of subgroups not necessarily adding up to England-Wales aggregate
[c] Direct deaths: where underlying cause was COVID (confirmed or suspected) or respiratory

Table D: Percentage of estimated excess YLL for weeks 11-52 in 2020 over the total of YLLs for equivalent weeks in 2019 (and 95% CI)

	**Direct**		**Indirect**			
	COVID or respiratory	Respiratory only	Cardio-vascular & diabetes	Neoplasm	Other indirect deaths	Any cause
England	123(120,126)	-15(-17,-12)	6(4,7)	-1(-2,0)	4(1,6)	15(14,16)
Sex						
male	146(143,148)	-10(-13,-8)	8(7,9)	-1(-3,-0)	4(1,6)	17(16,18)
female	100(97,102)	-19(-22,-17)	3(1,6)	-1(-2,1)	4(2,6)	13(11,14)
Regions						
North East	114(110,118)	-12(-16,-8)	7(6,9)	-1(-2,1)	13(11,16)	19(18,20)
North West	132(128,135)	-17(-20,-14)	4(2,6)	-1(-3,0)	2(0,4)	17(15,18)
Yorkshire & Humber	128(125,130)	-15(-17,-12)	3(1,5)	0(-1,2)	4(1,7)	16(15,17)
East Midlands	121(118,124)	-14(-17,-11)	8(7,10)	1(-1,2)	4(1,8)	16(15,17)
West Midlands	138(135,141)	-13(-16,-10)	10(8,12)	-0(-1,1)	5(3,7)	18(17,20)
East of England	102(99,105)	-17(-20,-14)	2(0,4)	-4(-6,-2)	4(0,8)	11(9,12)
London	242(239,245)	-11(-14,-8)	8(7,9)	-2(-3,-1)	4(2,6)	24(23,25)
South East Coast	99(96,102)	-14(-17,-11)	6(4,8)	-2(-4,-1)	9(6,11)	14(12,15)
South Central	103(99,107)	-9(-13,-5)	8(6,11)	-1(-3,0)	2(-1,6)	12(10,14)
South West	53(50,56)	-19(-22,-15)	5(3,7)	1(-1,2)	2(-0,5)	7(6,9)
Wales	95(92,97)	-16(-19,-14)	9(7,11)	1(-1,3)	-1(-3,2)	13(12,14)
Deprivation quintiles						
1 (least deprived)	129(126,132)	-13(-16,-10)	7(5,9)	1(-1,2)	5(1,8)	15(13,16)
2	123(120,126)	-13(-16,-10)	3(1,5)	-1(-3,0)	5(2,7)	14(12,15)
3	117(115,120)	-16(-18,-14)	6(4,8)	-2(-3,-1)	4(2,7)	14(13,15)
4	126(124,129)	-17(-20,-15)	6(5,7)	-1(-3,0)	4(2,6)	16(15,17)
5 (most deprived)	122(120,125)	-13(-16,-11)	8(7,9)	-0(-2,1)	4(2,6)	18(16,19)

[a] Using single ICD-10 code reported in underlying cause field (mutually exclusive)
[b] Sums of subgroups not necessarily adding up to England-Wales aggregate
[c] Direct deaths: where underlying cause was COVID (confirmed or suspected) or respiratory

3 Tables

Table E: Excess deaths (and 95% CI) by age-group for weeks 11-52 (week ending 13 Mar to week ending 25 Dec 2020)

COVID/repiratory

	15-44	45-64	65-74	75-84	85+
IMD					
1(least)	51(46,57)	681(658,703)	1377(1317,1436)	3655(3515,3795)	5636(5389,5883)
2	75(69,81)	856(827,884)	1538(1467,1609)	3801(3646,3957)	5376(5143,5609)
3	87(79,95)	1087(1051,1123)	1694(1612,1777)	4059(3908,4211)	4902(4684,5120)
4	153(142,164)	1599(1546,1653)	2122(2021,2224)	4613(4444,4781)	5015(4806,5223)
5(most)	268(250,285)	2008(1927,2090)	2774(2648,2900)	5069(4896,5242)	5302(5125,5479)
Regions					
NE	9(5,13)	237(220,254)	519(487,550)	1312(1260,1365)	1799(1734,1864)
NW	108(99,116)	1081(1042,1120)	1757(1685,1830)	3879(3756,4003)	4106(3965,4248)
York&H	41(35,48)	610(583,637)	1050(999,1102)	2481(2396,2566)	3104(3004,3204)
EastM	40(35,45)	477(455,498)	807(766,847)	1766(1695,1836)	2314(2225,2404)
WestM	92(85,99)	730(706,754)	1113(1065,1160)	2514(2427,2602)	2922(2811,3034)
East	43(37,48)	500(479,522)	827(784,870)	1862(1779,1944)	2304(2183,2425)
London	205(199,211)	1490(1465,1514)	1615(1575,1655)	2844(2779,2909)	3098(3006,3189)
SECoast	30(26,35)	333(315,351)	564(528,601)	1353(1286,1419)	2123(2020,2227)
SouthC	25(21,29)	278(264,292)	410(381,438)	1051(998,1104)	1564(1484,1643)
SW	12(8,17)	231(210,251)	366(326,406)	933(856,1010)	1563(1448,1679)
Wales	30(26,34)	283(264,302)	519(483,555)	1243(1181,1304)	1408(1329,1486)

Total

	15-44	45-64	65-74	75-84	85+
IMD					
1(least)	42(-13,98)	1050(862,1237)	1722(1419,2024)	4279(3669,4890)	6094(5092,7096)
2	104(43,165)	1268(1060,1476)	1795(1471,2118)	3883(3281,4486)	5717(4776,6659)
3	29(-49,107)	1710(1477,1942)	2236(1909,2563)	4535(3961,5109)	5772(4905,6639)
4	273(176,369)	2361(2080,2643)	2393(2037,2748)	5435(4869,6001)	5471(4702,6241)
5(most)	480(345,615)	3150(2786,3515)	3317(2929,3705)	5916(5382,6450)	5771(5127,6416)
Regions					
NE	165(135,195)	500(421,580)	577(472,683)	1401(1243,1560)	1703(1488,1918)
NW	127(70,184)	1757(1583,1930)	2124(1896,2352)	4137(3768,4506)	4270(3803,4738)
York&H	137(93,182)	903(778,1029)	1077(907,1248)	2462(2178,2747)	3150(2779,3522)
EastM	29(-9,68)	833(725,941)	1000(851,1149)	2087(1839,2334)	2379(2046,2712)
WestM	139(91,187)	1169(1042,1296)	1553(1381,1726)	3306(3007,3605)	3509(3095,3923)
East	43(2,85)	478(347,609)	966(789,1144)	1842(1540,2145)	2782(2324,3240)
London	273(216,330)	2002(1858,2147)	1805(1648,1962)	3356(3120,3592)	3338(2993,3682)
SECoast	57(23,91)	632(534,730)	807(671,944)	1762(1522,2001)	2637(2252,3022)
SouthC	29(-3,61)	392(304,479)	562(449,674)	1188(1002,1375)	1704(1410,1999)
SW	1(-39,42)	493(375,611)	600(434,765)	1330(1046,1614)	2313(1853,2773)
Wales	-39(-71,-7)	512(420,604)	563(445,682)	1422(1233,1611)	1391(1135,1647)

4 Tables

Table F: Excess deaths (and 95% CI) by age-group for weeks 11-52 (week ending 13 Mar to week ending 25 Dec 2020), per 100,000 population

COVID/repiratory

	15-44	45-64	65-74	75-84	85+
IMD					
1(least)	1(1,2)	22(21,23)	107(102,112)	407(386,427)	1545(1462,1627)
2	2(2,2)	28(27,29)	120(114,126)	435(411,458)	1567(1485,1649)
3	2(2,2)	36(34,37)	140(133,148)	513(488,538)	1546(1463,1628)
4	3(3,3)	55(53,57)	204(193,216)	723(690,756)	1933(1837,2028)
5(most)	5(5,6)	73(69,76)	306(288,323)	989(947,1030)	2524(2423,2625)
Regions					
NE	1(0,1)	35(32,37)	177(166,189)	762(729,795)	2650(2542,2757)
NW	4(3,4)	57(54,59)	235(224,246)	828(796,861)	2295(2201,2389)
York&H	2(2,2)	44(42,46)	191(181,201)	724(695,753)	2311(2226,2396)
EastM	2(2,3)	38(36,39)	160(151,168)	545(518,572)	1912(1829,1994)
WestM	4(4,4)	49(47,51)	196(187,205)	644(617,671)	1978(1893,2063)
East	2(2,2)	31(29,32)	129(122,136)	444(421,468)	1307(1229,1385)
London	5(5,5)	72(70,73)	272(265,280)	829(808,849)	2016(1949,2083)
SECoast	2(2,2)	26(24,27)	113(105,121)	401(377,426)	1474(1393,1556)
SouthC	2(1,2)	24(23,26)	99(92,106)	374(352,396)	1329(1254,1404)
SW	1(0,1)	15(14,17)	56(49,63)	207(185,229)	888(814,961)
Wales	3(2,3)	35(32,37)	148(137,159)	557(526,588)	1651(1549,1754)

Total

	15-44	45-64	65-74	75-84	85+
IMD					
1(least)	3(2,5)	47(40,54)	189(163,215)	454(367,541)	1713(1397,2029)
2	4(3,6)	53(46,61)	190(161,218)	403(313,493)	1725(1413,2036)
3	2(-0,4)	65(56,75)	217(185,249)	538(445,631)	1884(1576,2191)
4	6(3,8)	84(72,96)	236(194,277)	826(715,937)	2174(1842,2505)
5(most)	8(4,11)	106(89,123)	331(276,386)	1166(1041,1290)	2789(2442,3137)
Regions					
NE	17(14,21)	85(72,97)	204(164,245)	861(758,963)	2447(2097,2798)
NW	4(2,7)	97(86,108)	303(268,338)	849(754,944)	2381(2090,2672)
York&H	7(5,10)	72(62,82)	216(182,251)	720(624,816)	2349(2046,2651)
EastM	2(-0,4)	72(62,81)	226(193,258)	562(466,657)	2068(1771,2364)
WestM	6(4,8)	83(73,93)	307(273,341)	765(670,861)	2425(2115,2735)
East	3(1,5)	34(25,43)	179(148,209)	405(319,492)	1597(1314,1880)
London	8(6,9)	96(88,104)	306(276,337)	1053(977,1129)	2165(1916,2414)
SECoast	4(1,6)	49(40,58)	182(152,213)	455(367,543)	1897(1606,2189)
SouthC	4(2,6)	42(33,50)	174(146,202)	401(322,479)	1530(1263,1797)
SW	1(-1,3)	37(28,46)	109(81,138)	221(138,304)	1456(1171,1740)
Wales	-3(-6,-0)	70(58,82)	181(144,217)	593(495,690)	1634(1312,1956)

5 Total

5.1 AASMRs

Figure 1: Age-standardised mortality trend, total deaths, 2015-2020

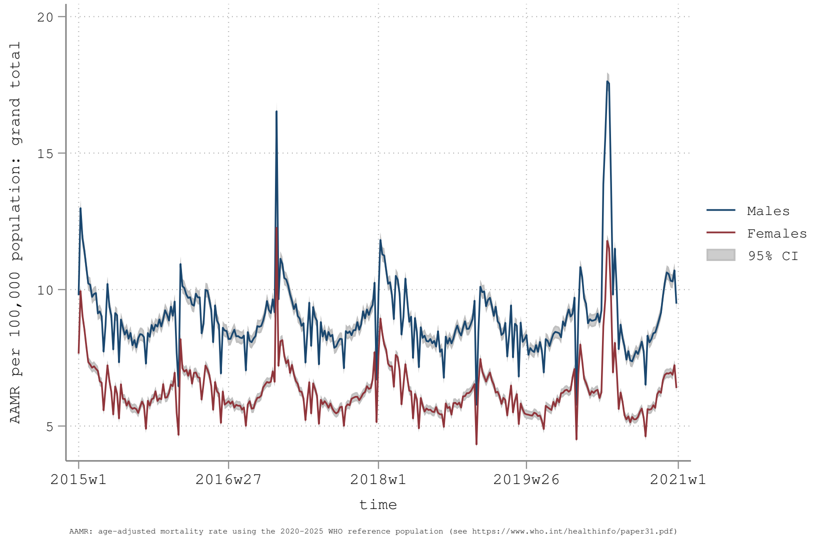

Figure 2: Age-standardised mortality trend, total deaths, 2019-2020

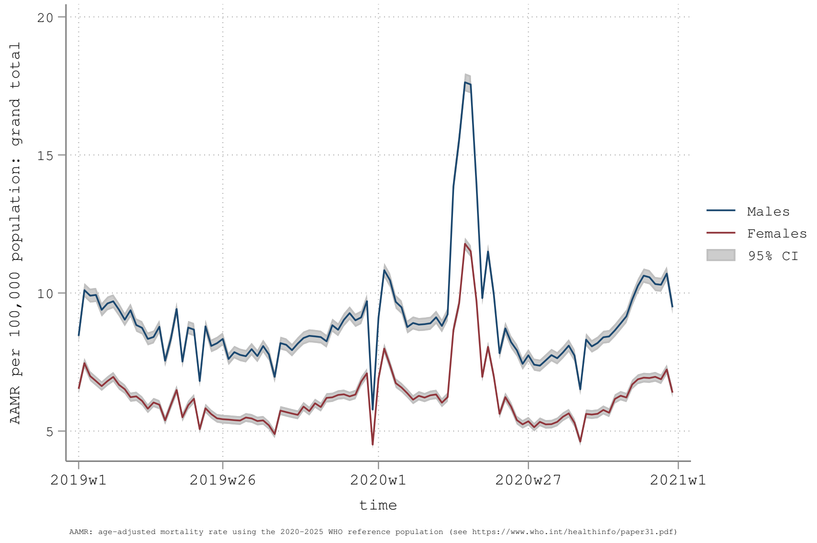

Figure 3: Age-standardised mortality trend, total deaths by deprivation quintile, 2015-2020

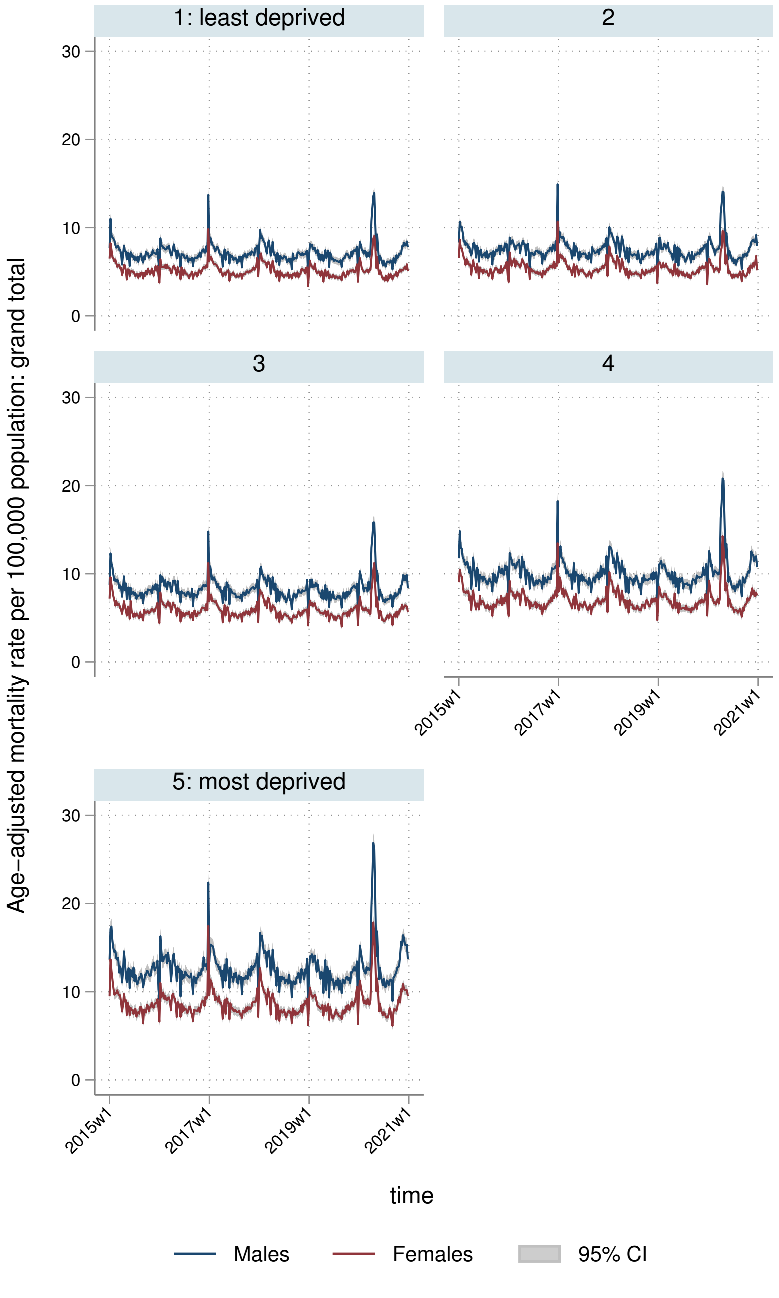

Figure 4: Age-standardised mortality trend, total deaths by deprivation quintile, 2019-2020

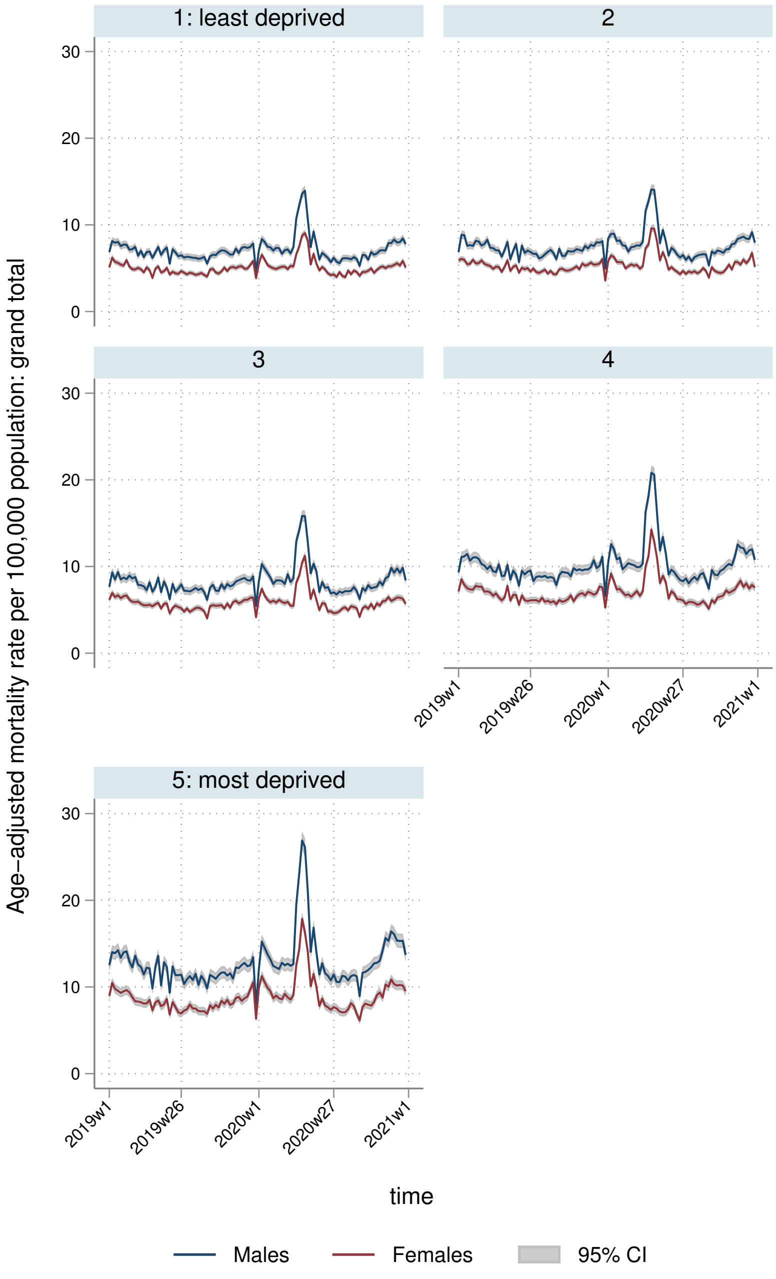

Figure 5: Age-standardised mortality trend, total deaths by region, 2015-2020

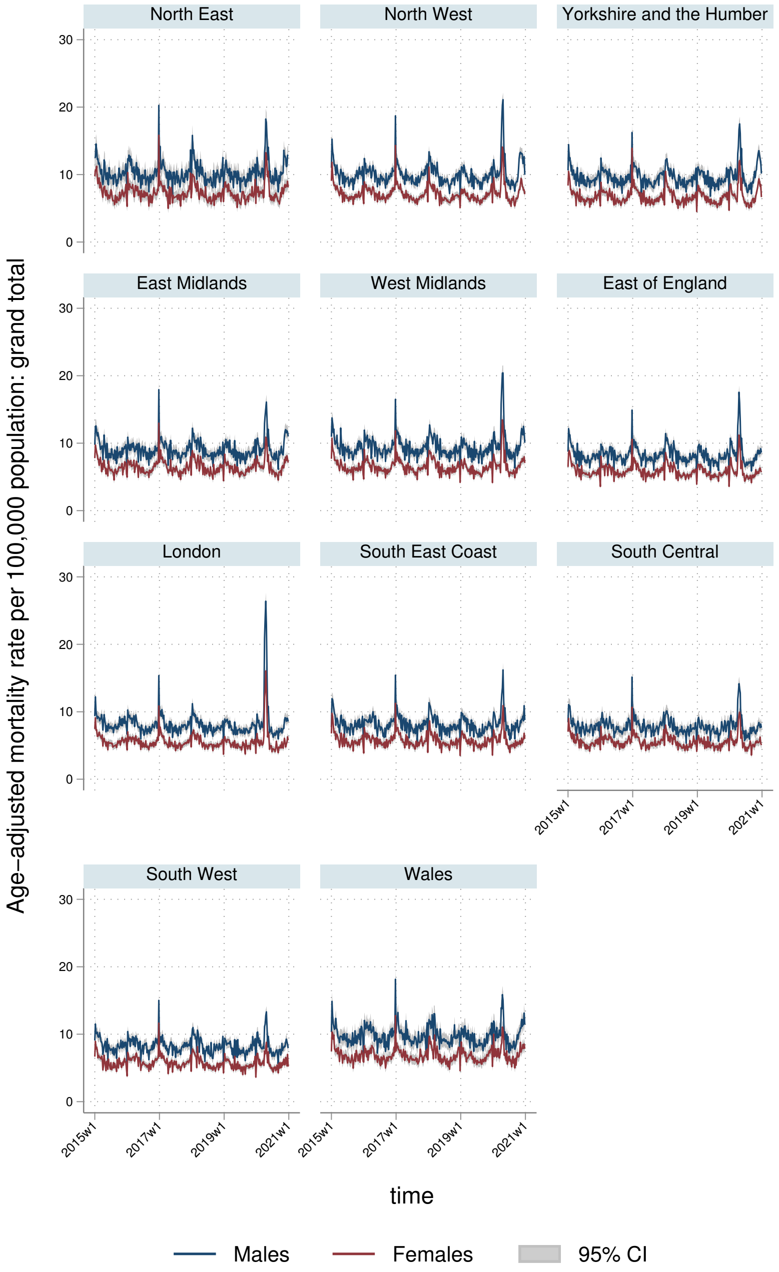

Figure 6: Age-standardised mortality trend, total deaths by region, 2019-2020

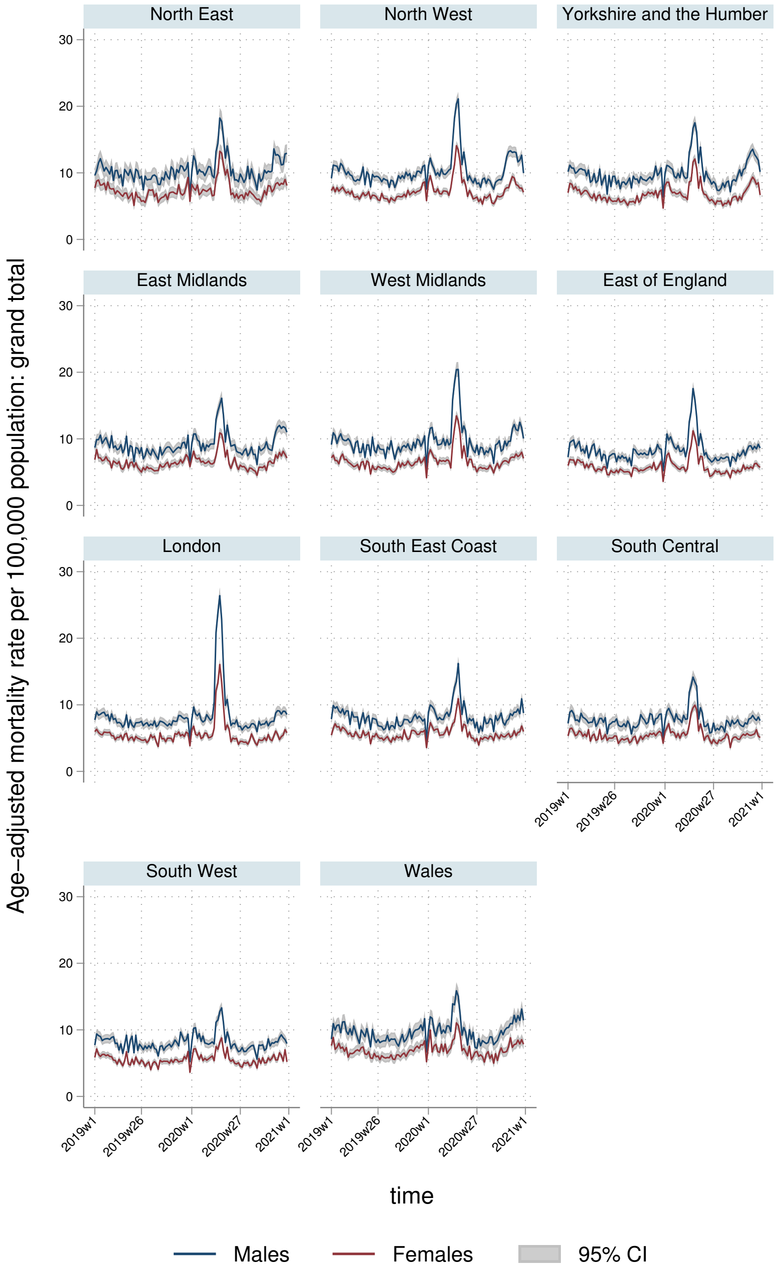

5.2 YLLs

5.2.1 England-Wales aggregate

Figure 7: Years of Life Lost trend, total deaths, 2015-2020

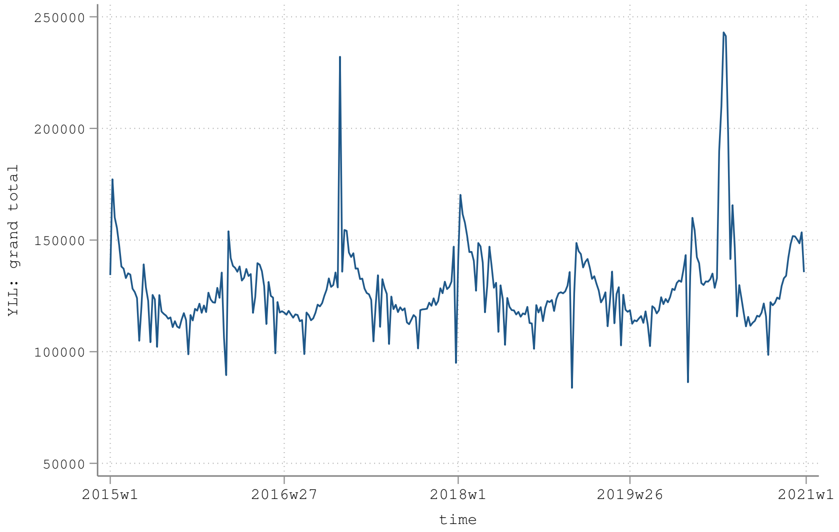

Figure 8: Years of Life Lost trend, total deaths, 2019-2020

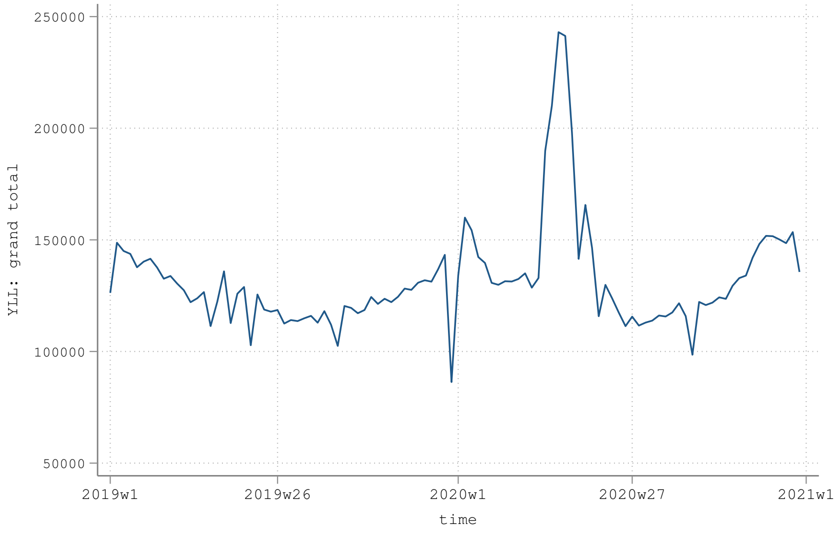

Figure 9: Excess Years of Life Lost trend, total deaths, 2015-2020

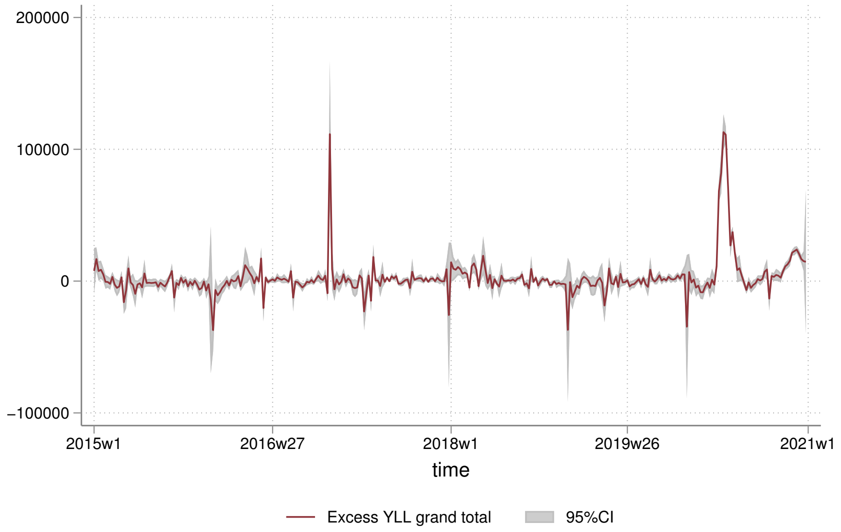

Figure 10: Excess Years of Life Lost trend, total deaths, 2019-2020

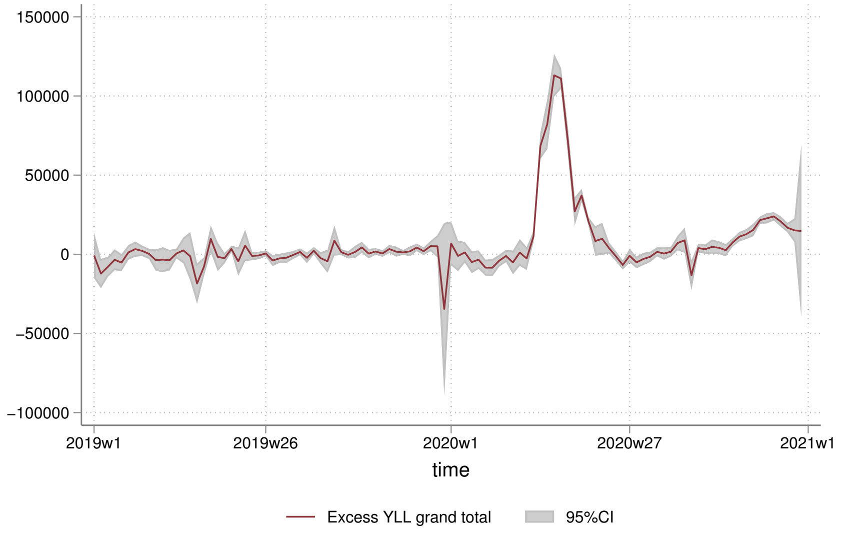

Figure 11: Observed vs Predicted Years of Life Lost trends, total deaths, 2015-2020

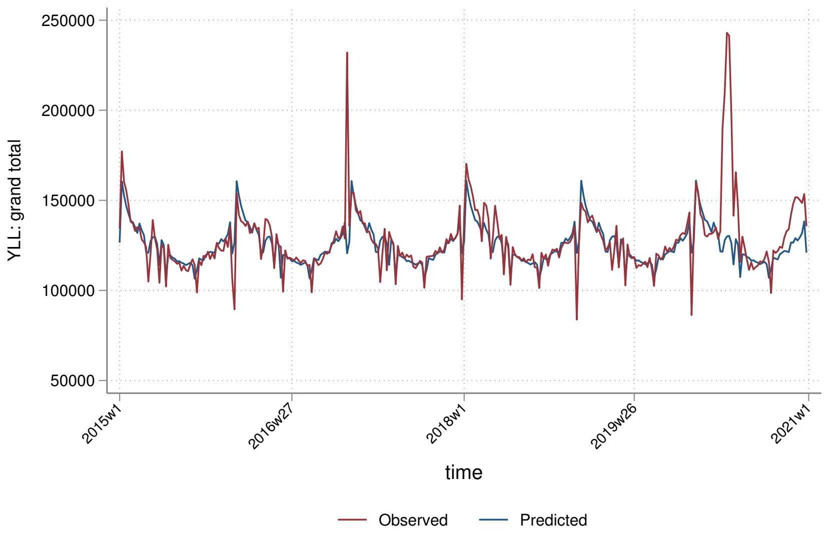

Figure 12: Observed vs Predicted Years of Life Lost trends, total deaths, 2019-2020

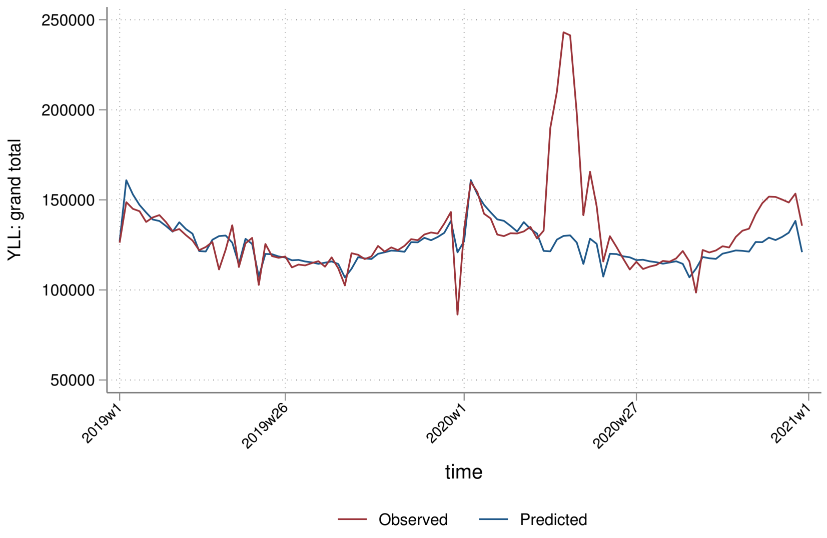

5.2.2 By sex

Figure 13: Years of Life Lost trend, total deaths by sex, 2015-2020

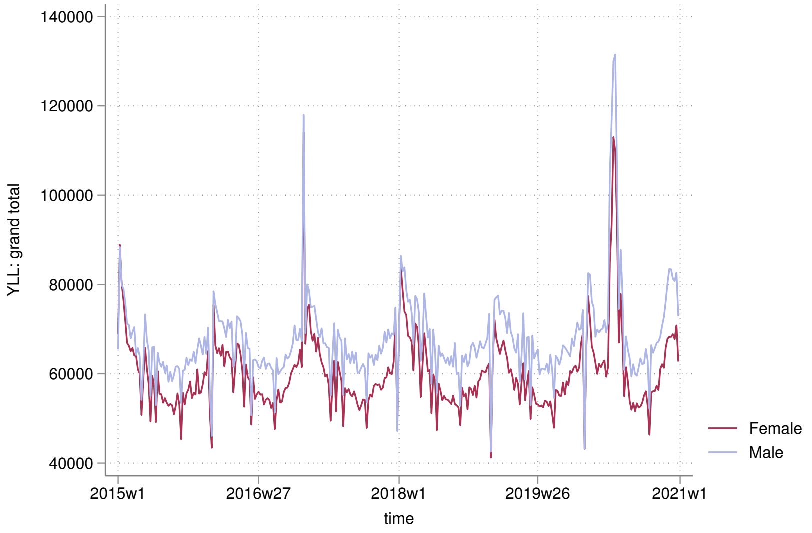

Figure 14: Years of Life Lost trend, total deaths by sex, 2019-2020

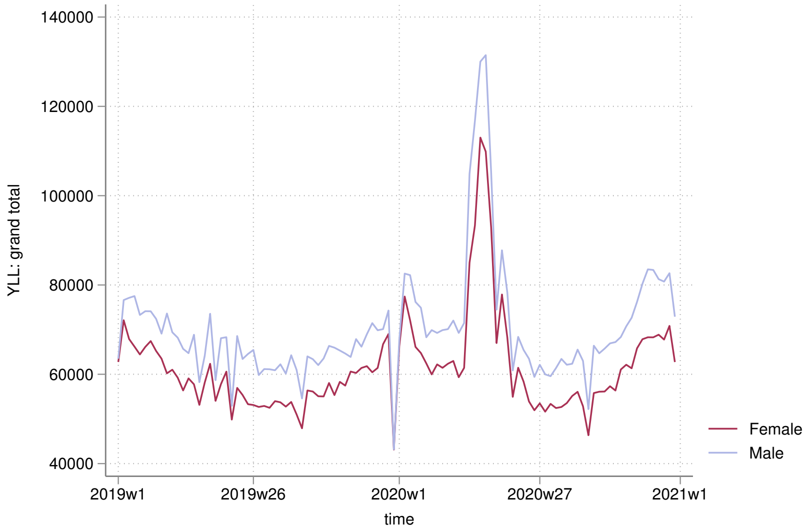

Figure 15: Excess Years of Life Lost trend, total deaths by sex, 2015-2020

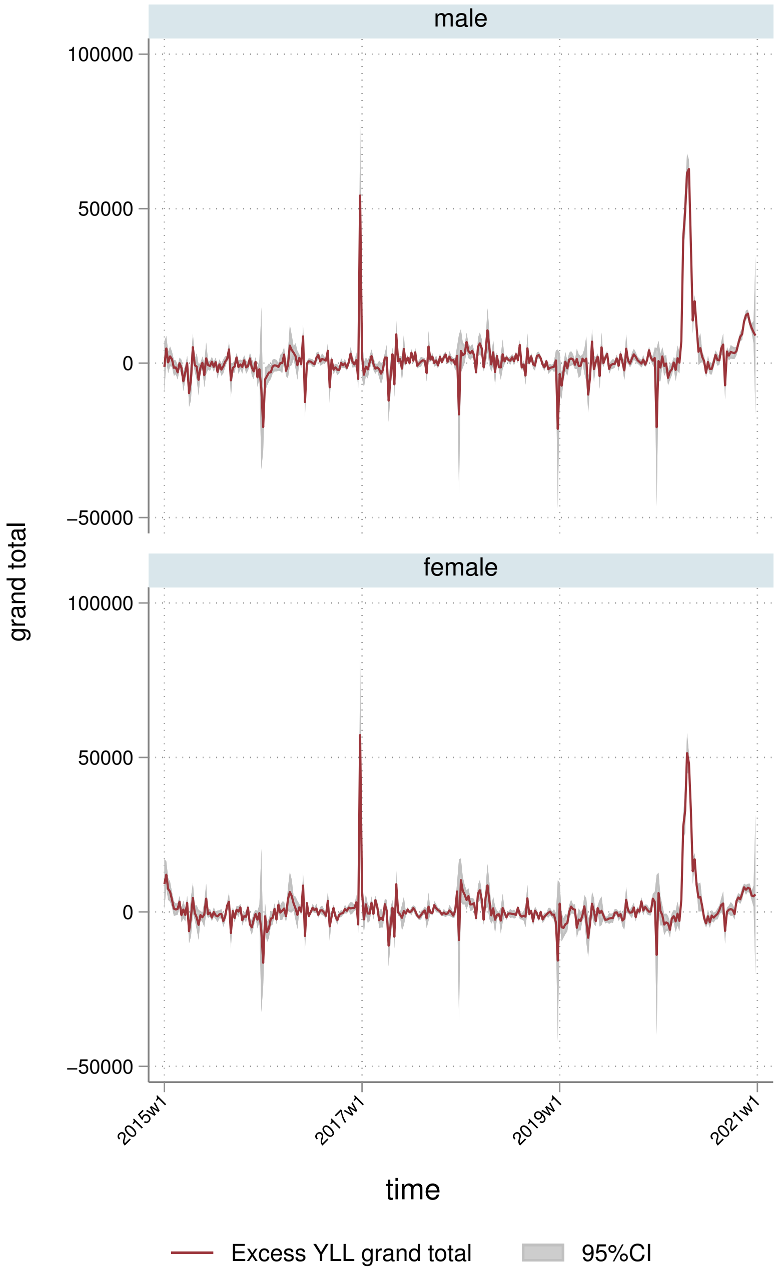

Figure 16: Excess Years of Life Lost trend, total deaths by sex, 2019-2020

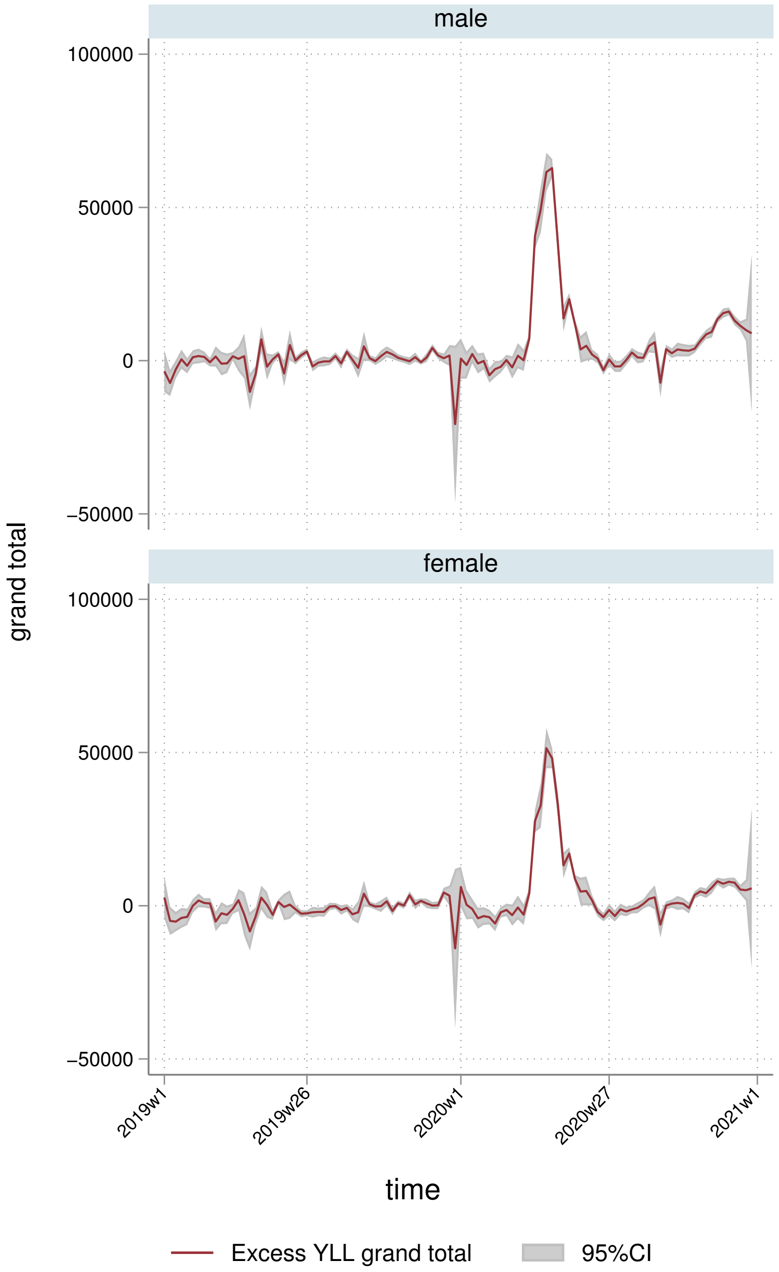

Figure 17: Observed vs Predicted Years of Life Lost trends, total deaths by sex, 2015-2020

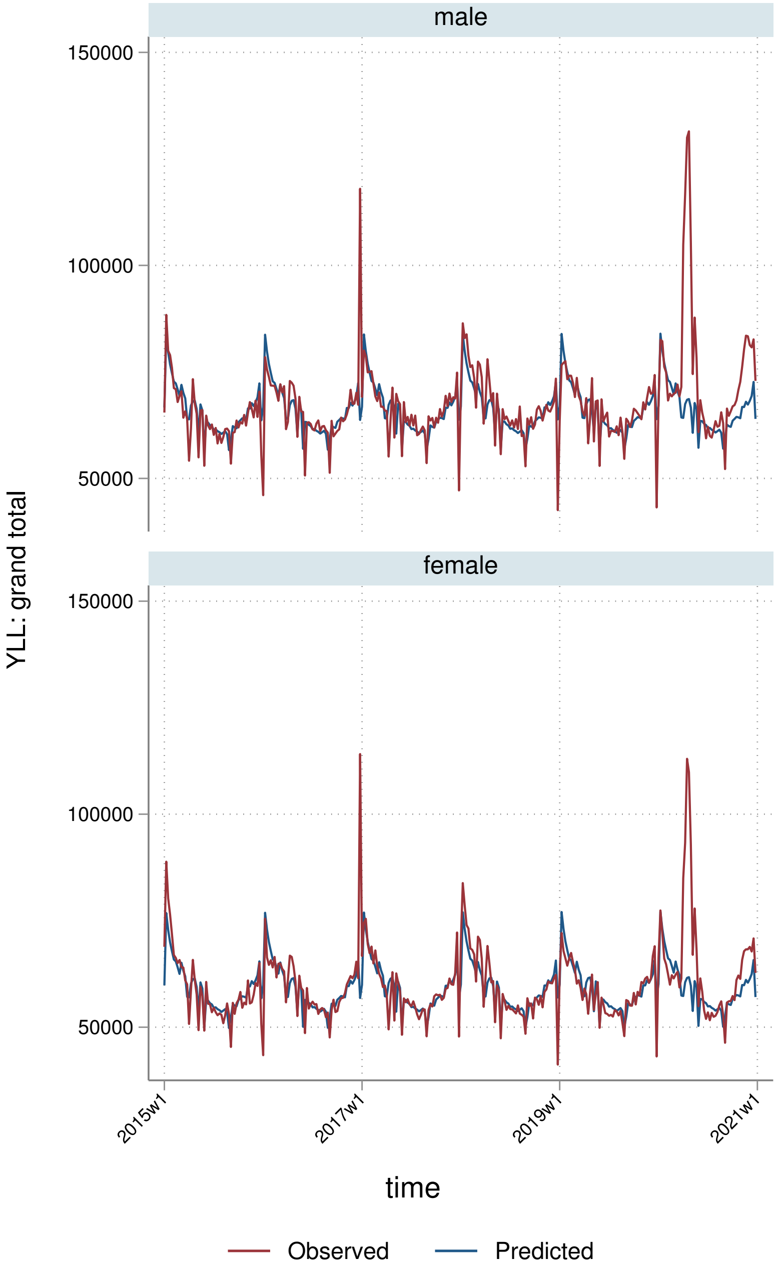

Figure 18: Observed vs Predicted Years of Life Lost trends, total deaths by sex, 2019-2020

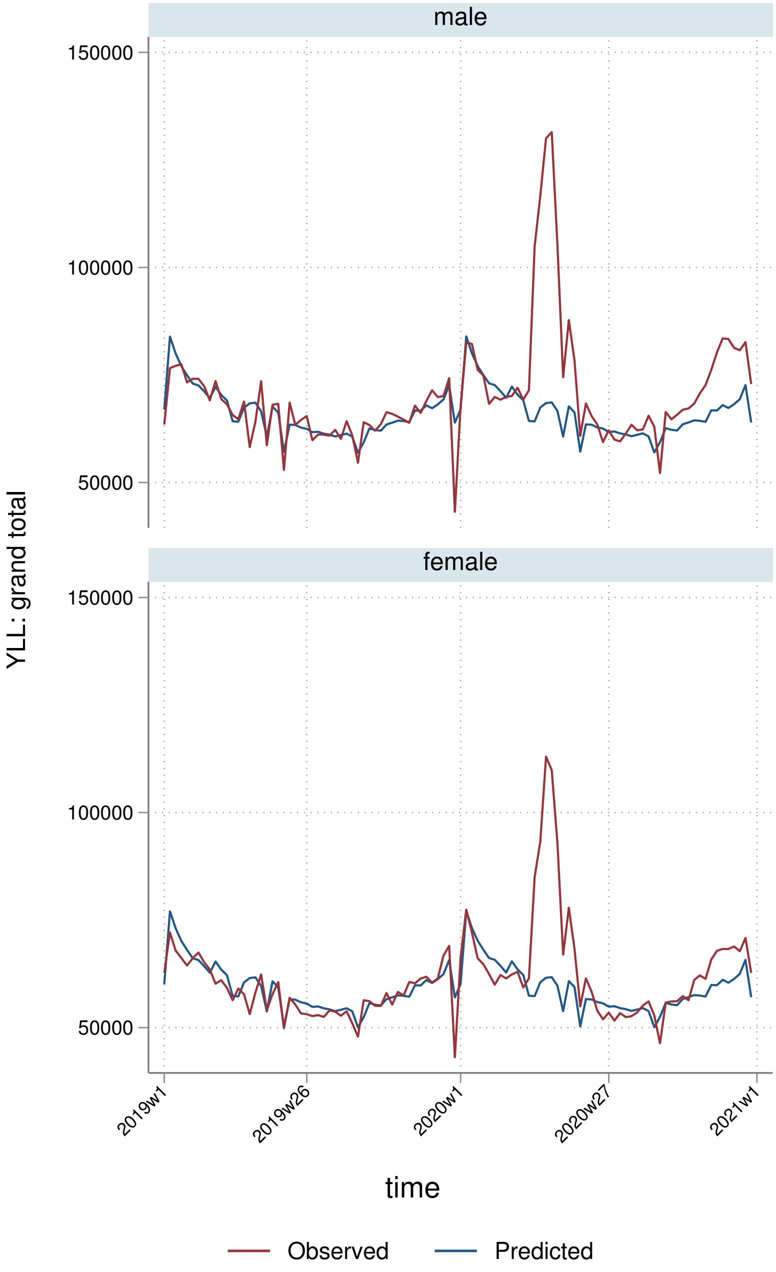

5.2.3 By deprivation quintile

Figure 19: Years of Life Lost trend, total deaths by deprivation quintile, 2015-2020

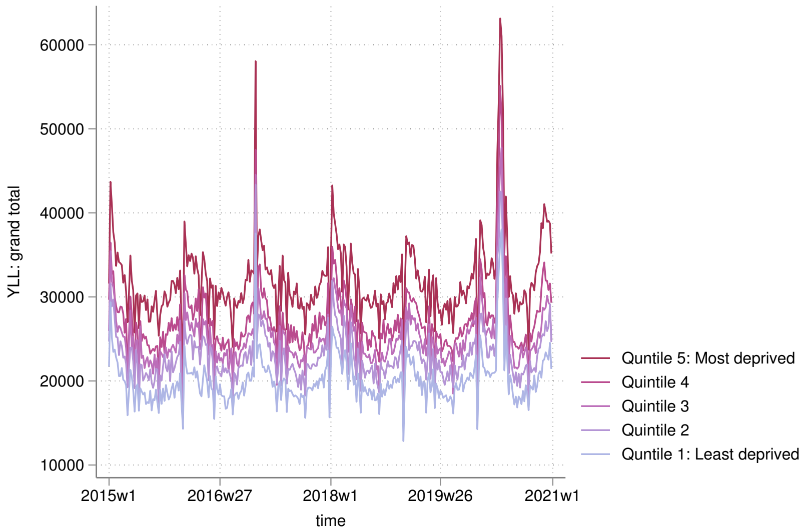

Figure 20: Years of Life Lost trend, total deaths by deprivation quintile, 2019-2020

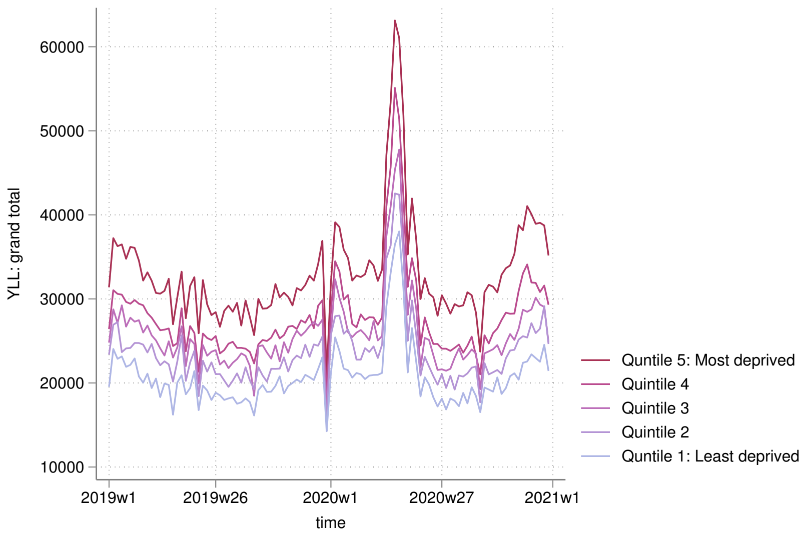

Figure 21: Excess Years of Life Lost trend, total deaths by deprivation quintile, 2015-2020

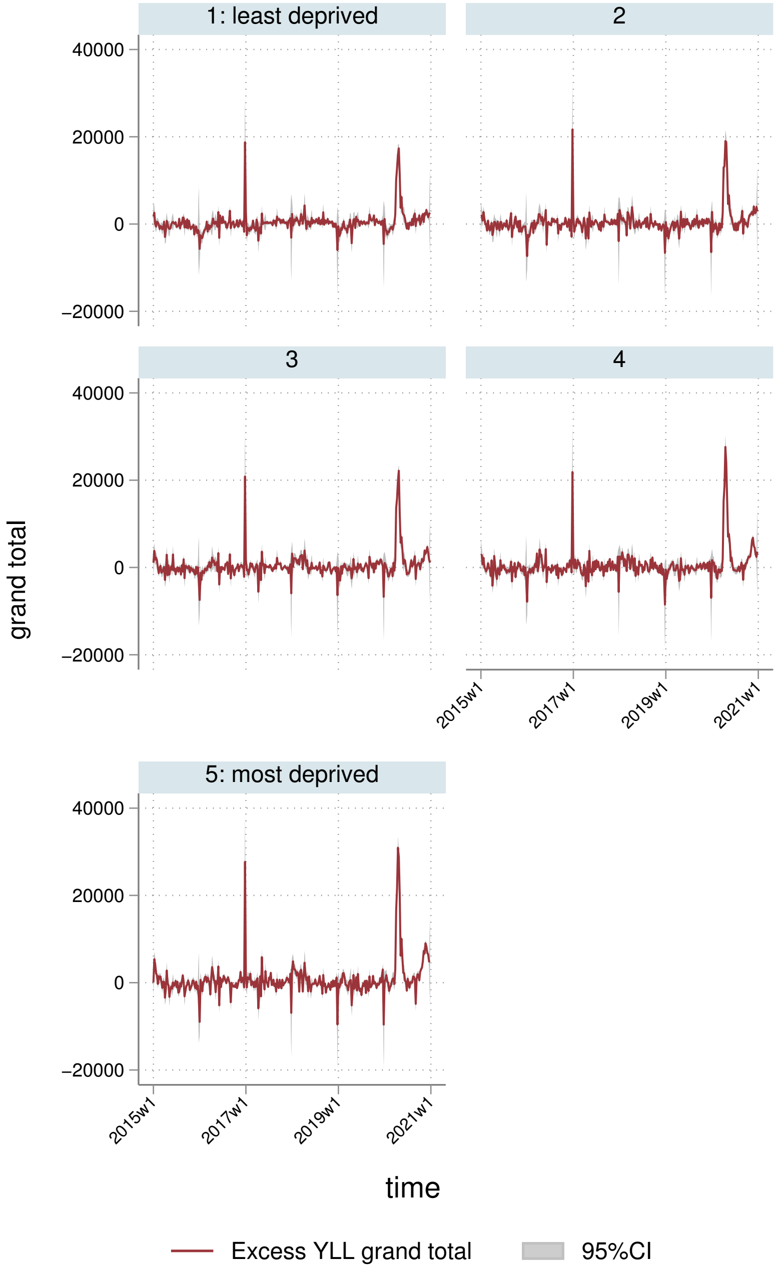

Figure 22: Excess Years of Life Lost trend, total deaths by deprivation quintile, 2019-2020

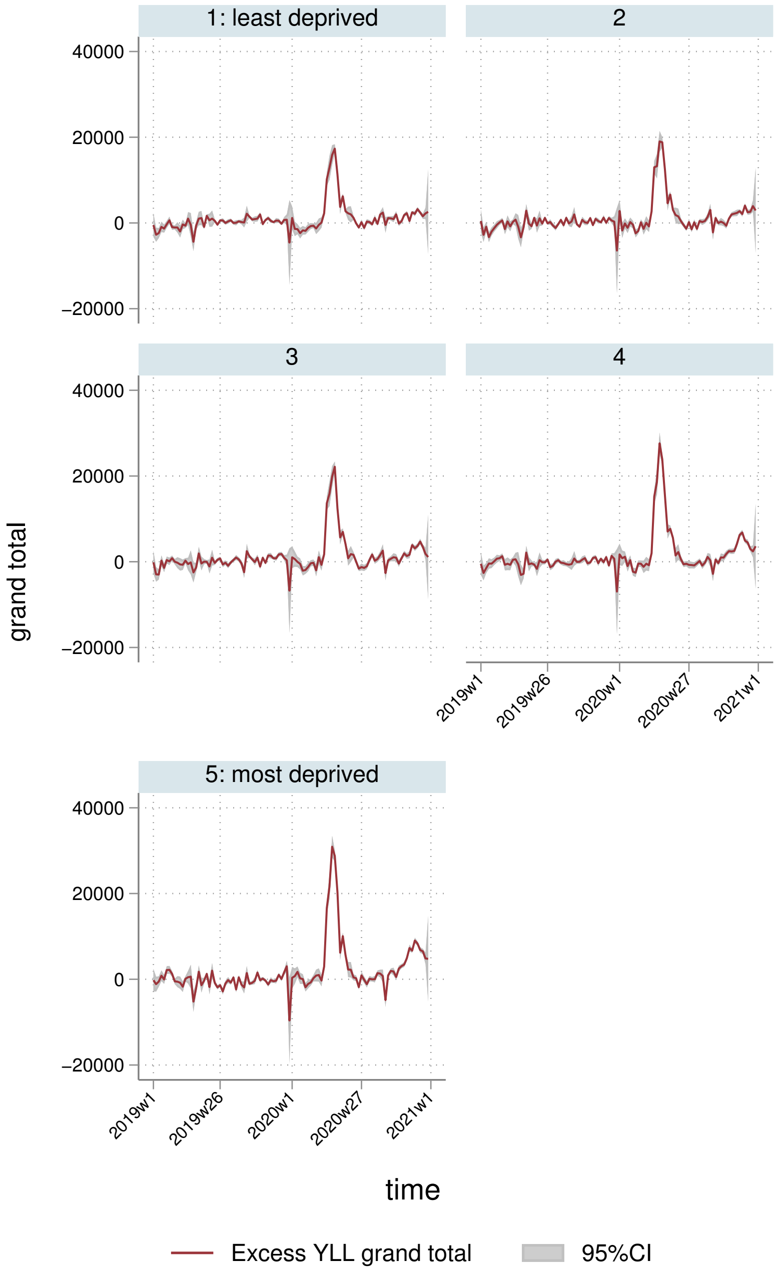

Figure 23: Observed vs Predicted Years of Life Lost trends, total deaths by deprivation quintile, 2015-2020

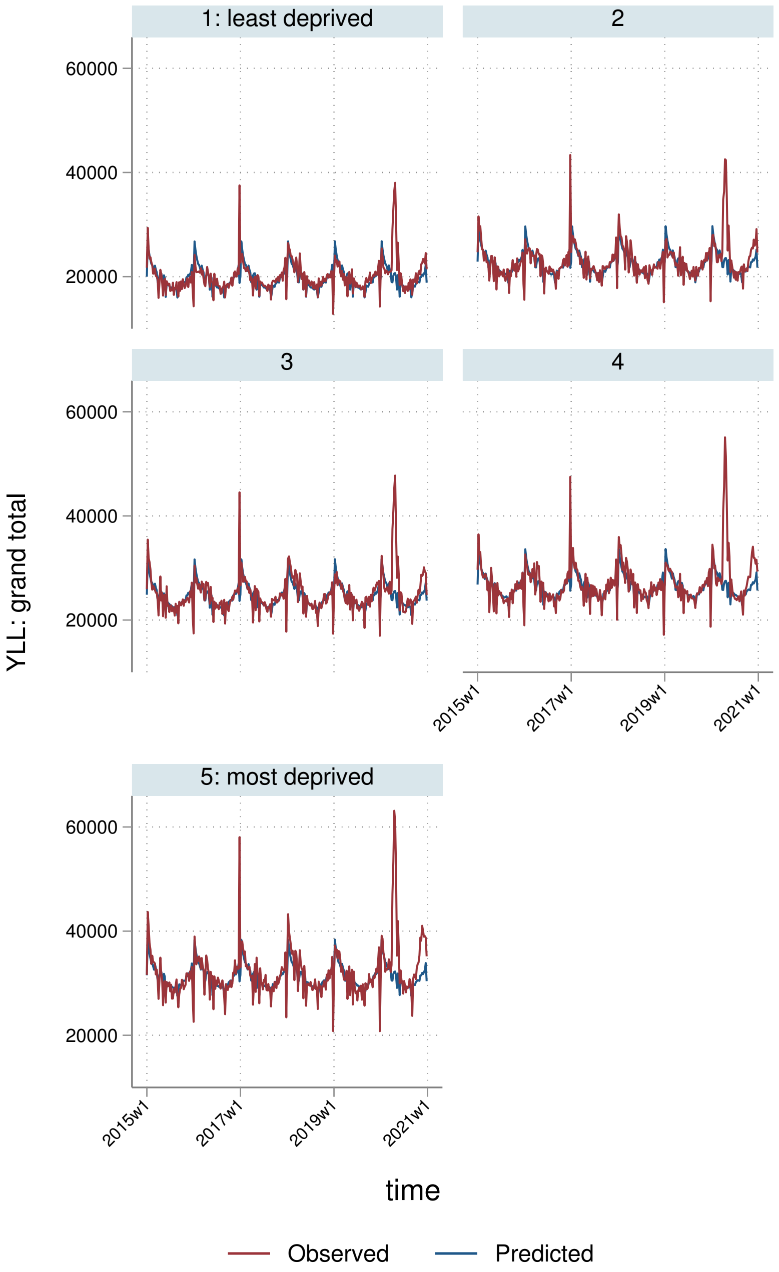

Figure 24: Observed vs Predicted Years of Life Lost trends, total deaths by deprivation quintile, 2019-2020

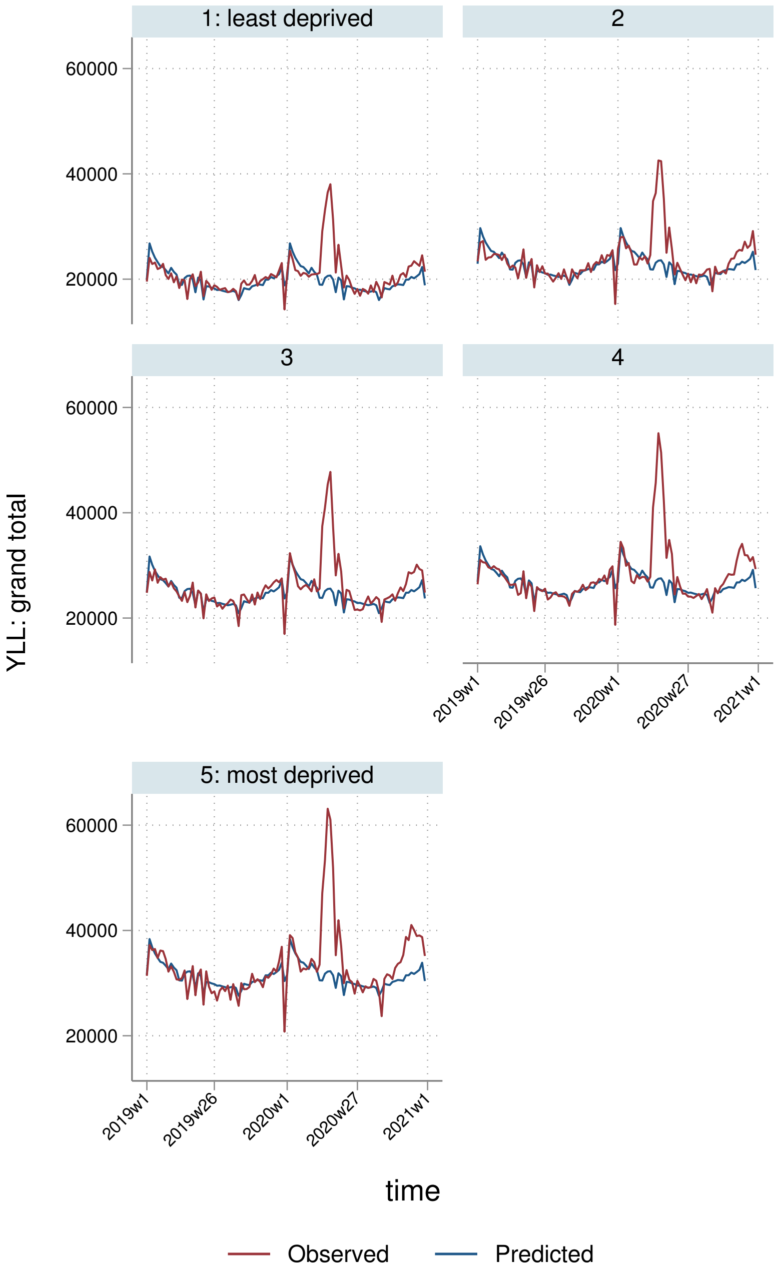

5.2.4 By Strategic Health Authority

Figure 25: Years of Life Lost trend, total deaths by region, 2015-2020

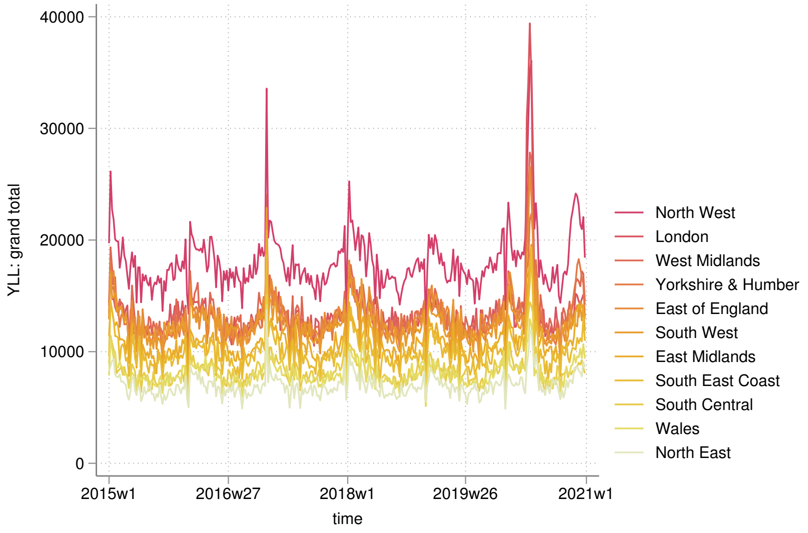

Figure 26: Years of Life Lost trend, total deaths by region, 2019-2020

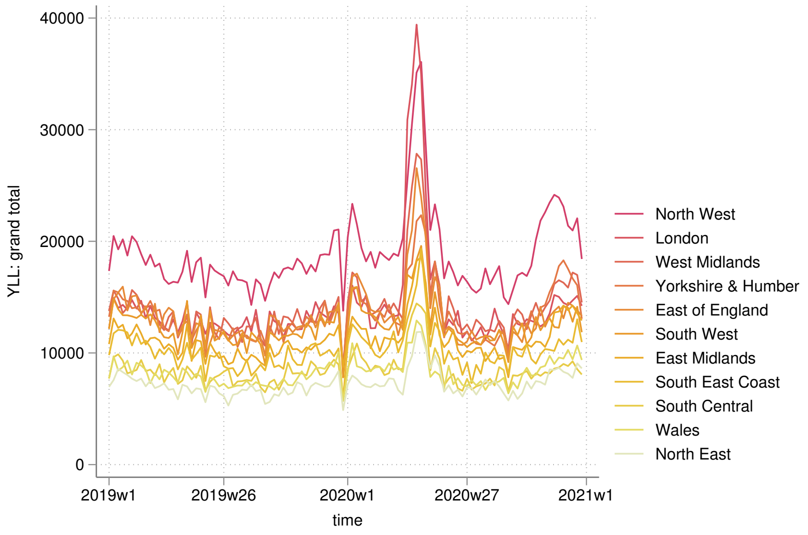

Figure 27: Excess Years of Life Lost trend, total deaths by region, 2015-2020

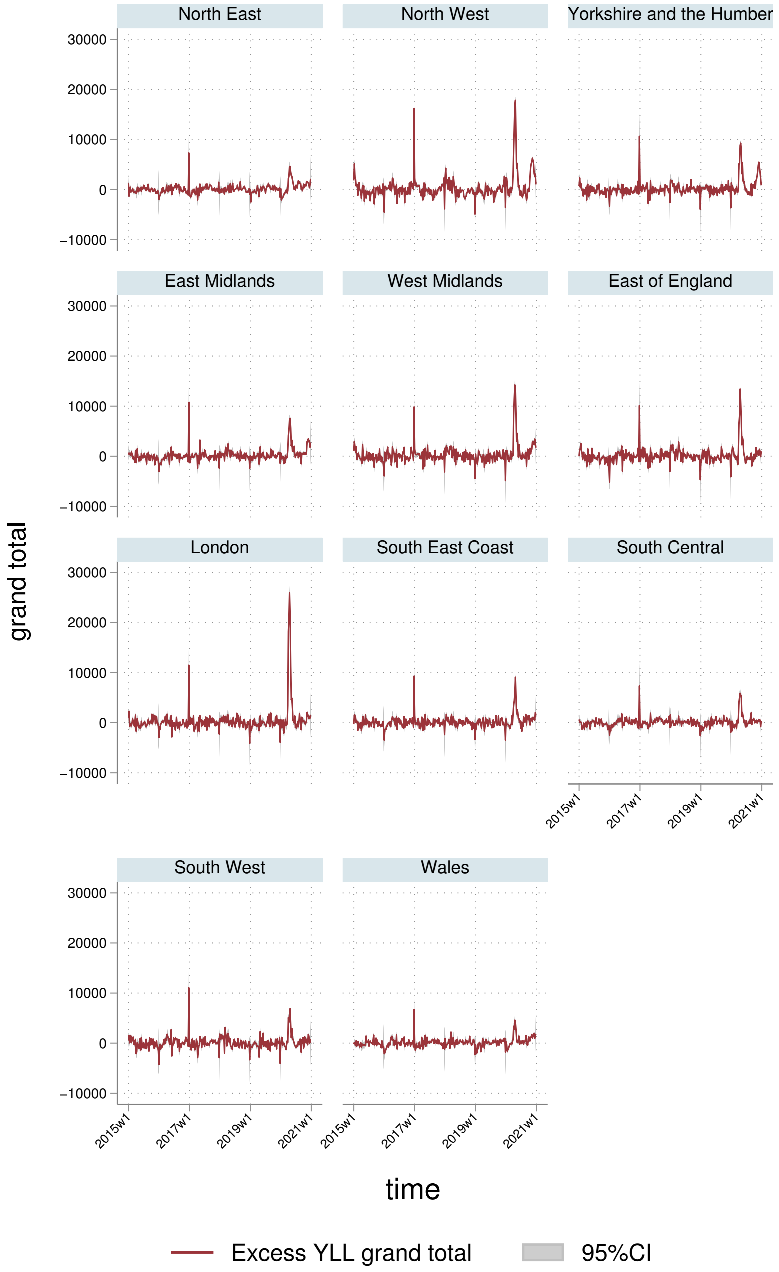

Figure 28: Excess Years of Life Lost trend, total deaths by region, 2019-2020

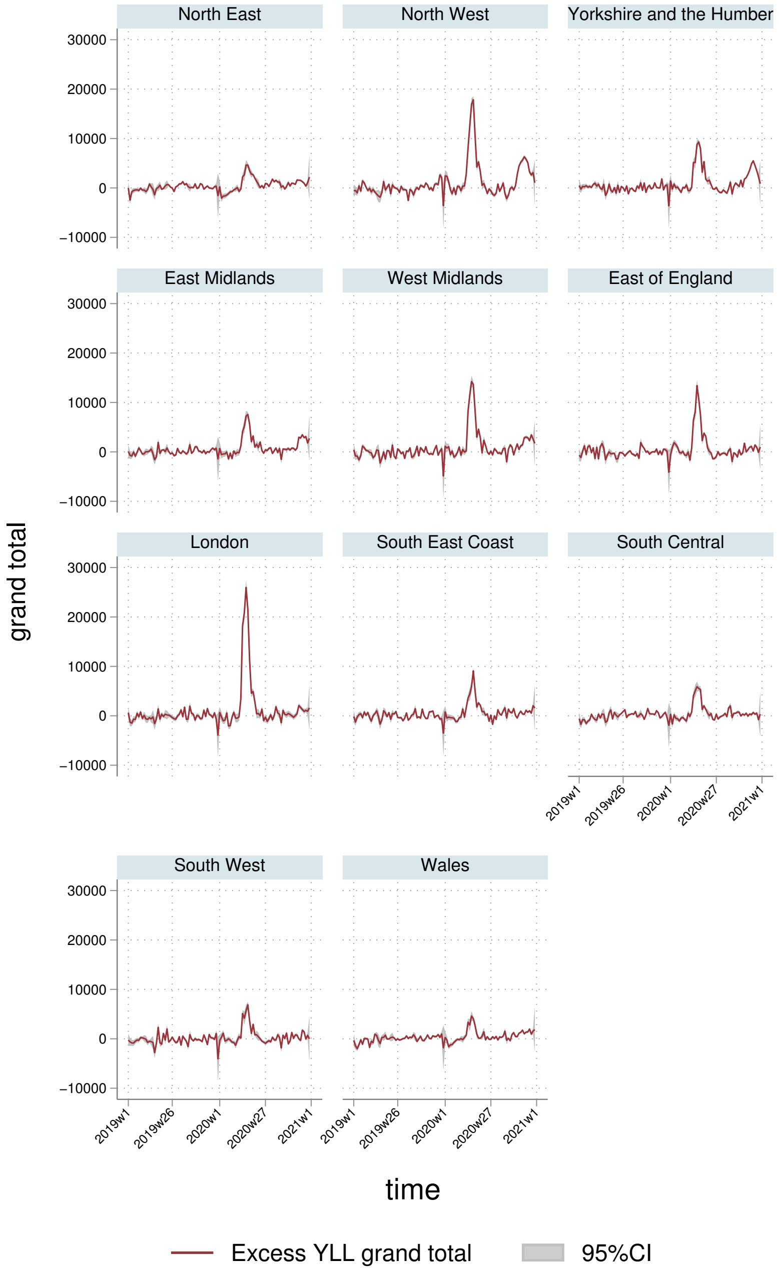

Figure 29: Observed vs Predicted Years of Life Lost trends, total deaths by region, 2015-2020

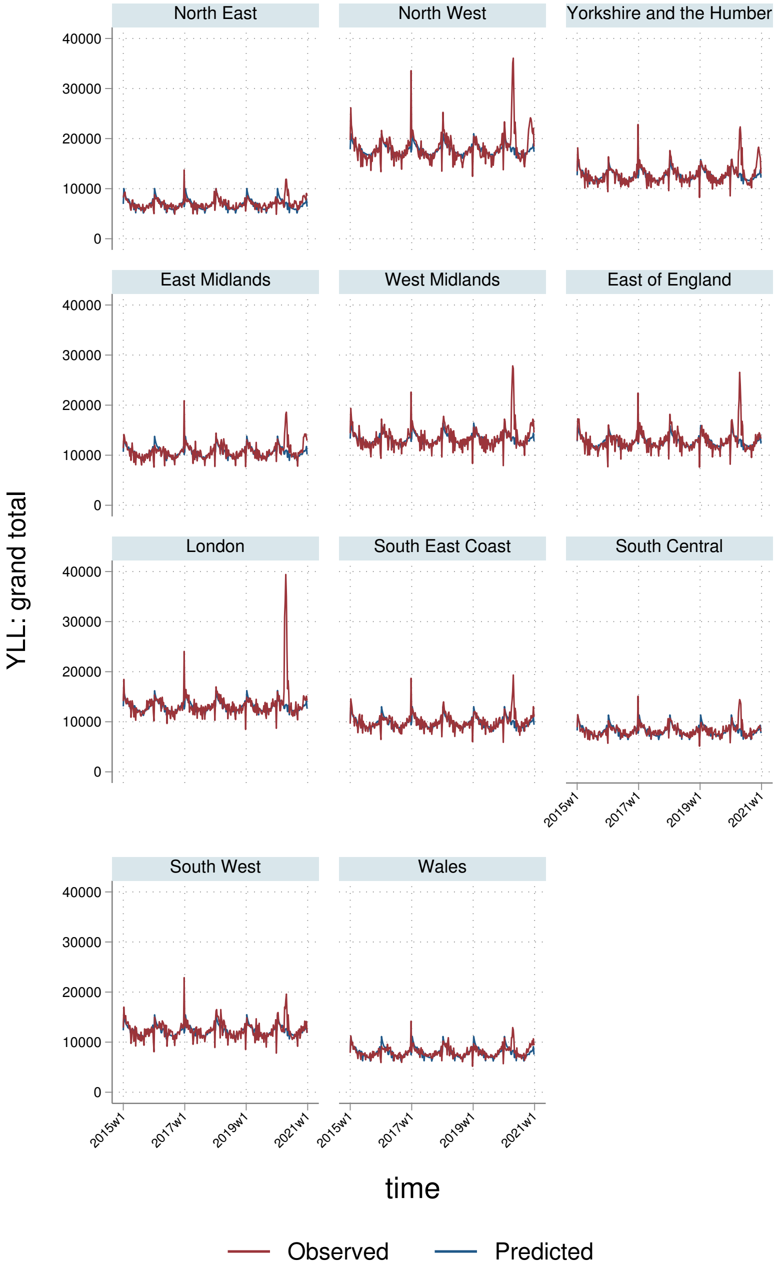

Figure 30: Observed vs Predicted Years of Life Lost trends, total deaths by region, 2019-2020

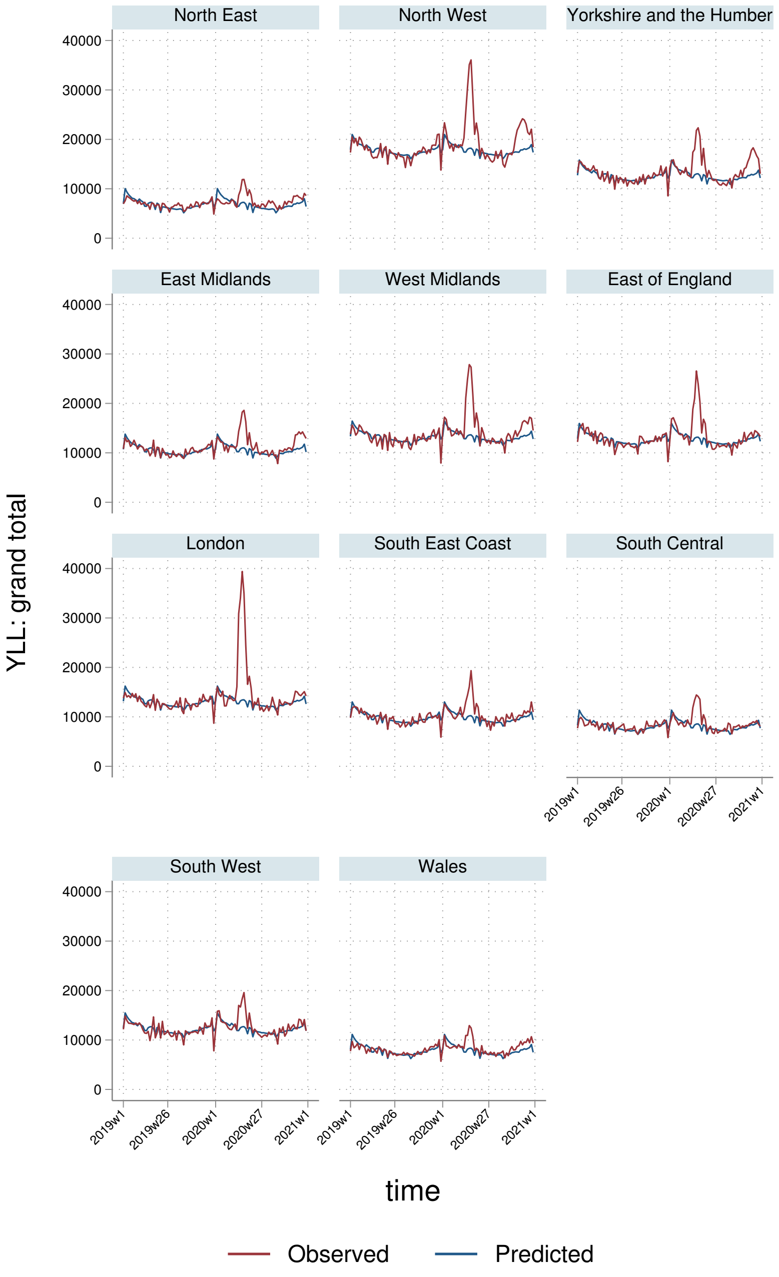

5.2.5 By deprivation quintile & Strategic Health Authority

Figure 31: Years of Life Lost trend, total deaths by regionXdeprivation, 2015-2020

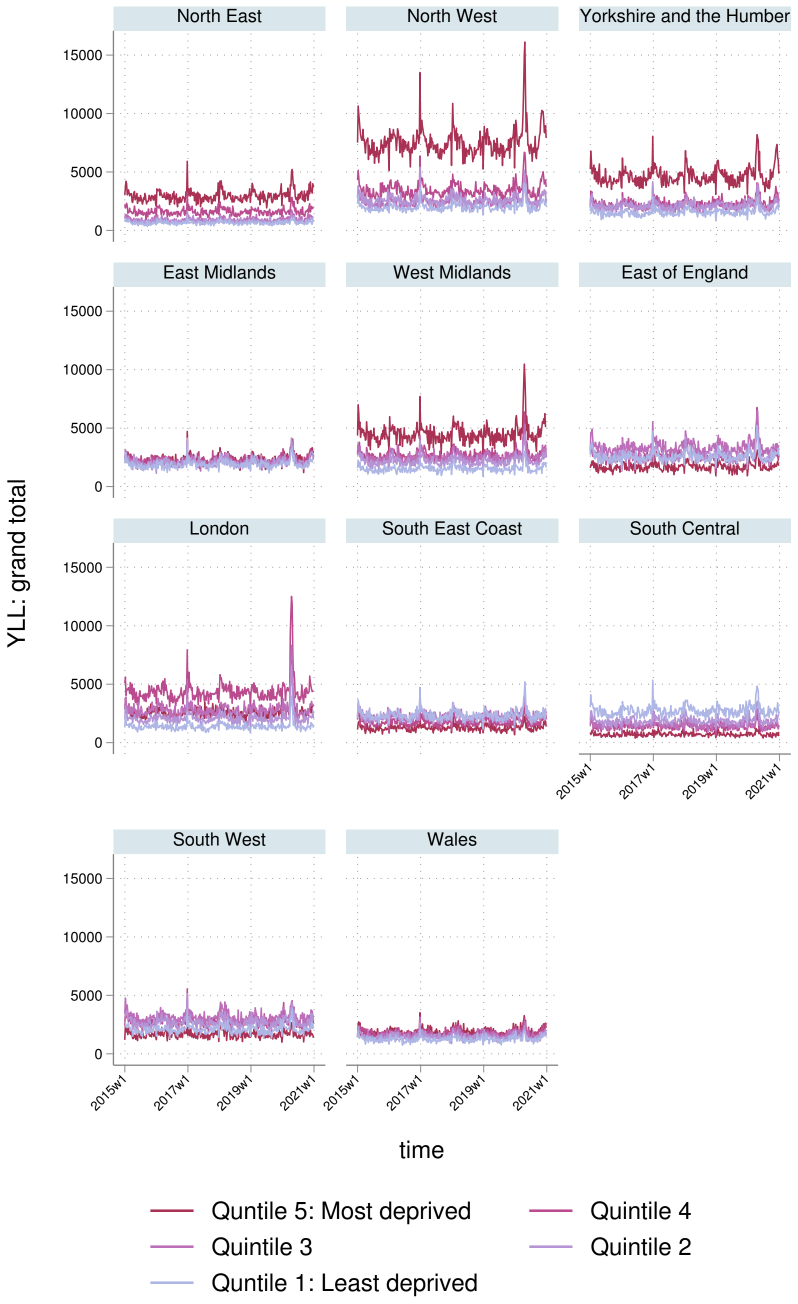

Figure 32: Years of Life Lost trend, total deaths by region, 2019-2020

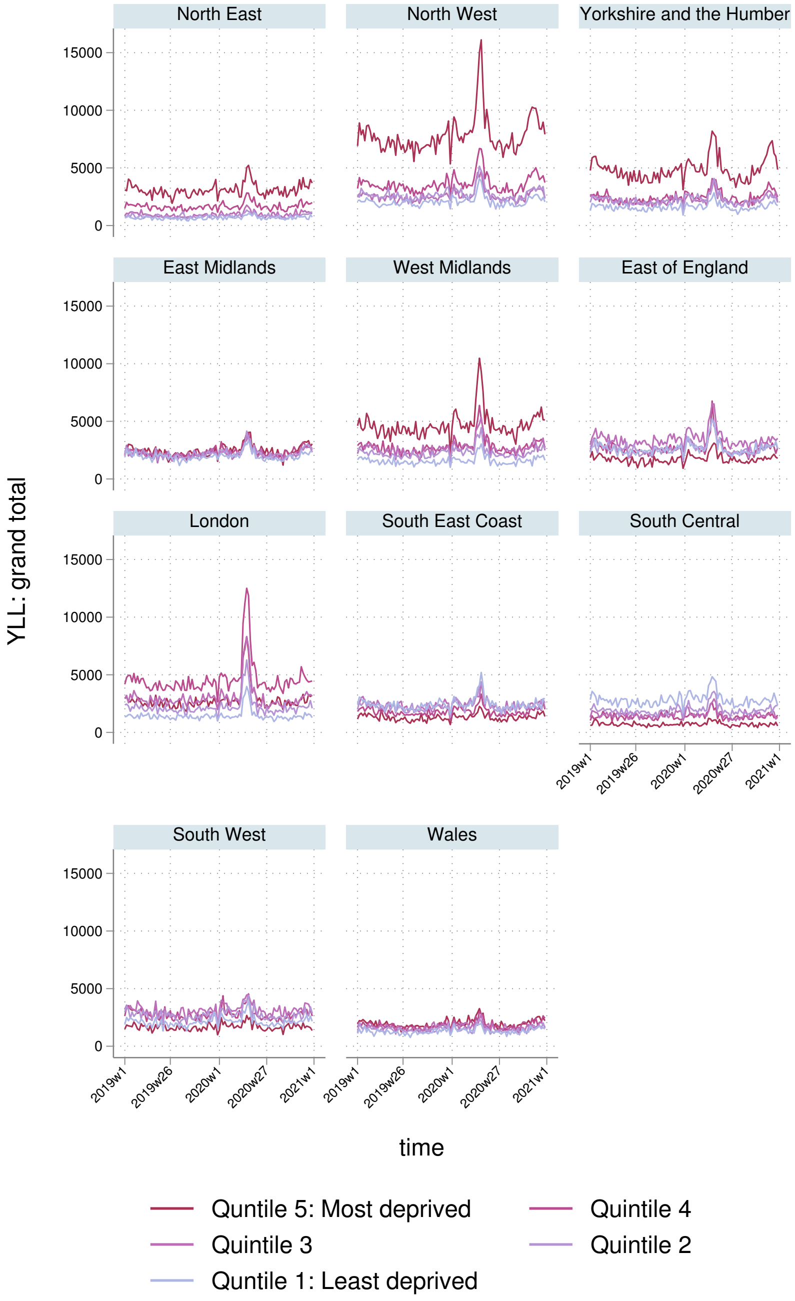

Figure 33: Excess Years of Life Lost trend, total deaths by regionXdeprivation, 2015-2020

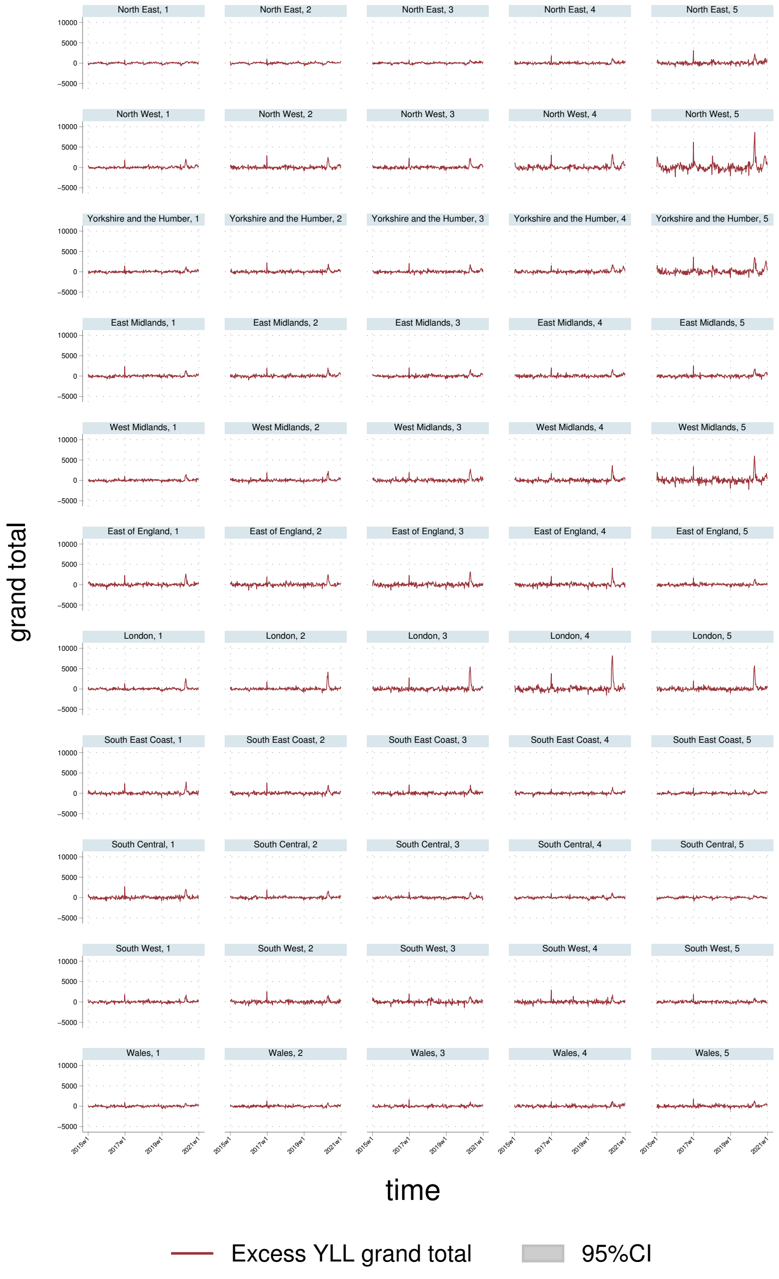

Figure 34: Excess Years of Life Lost trend, total deaths by regionXdeprivation, 2019-2020

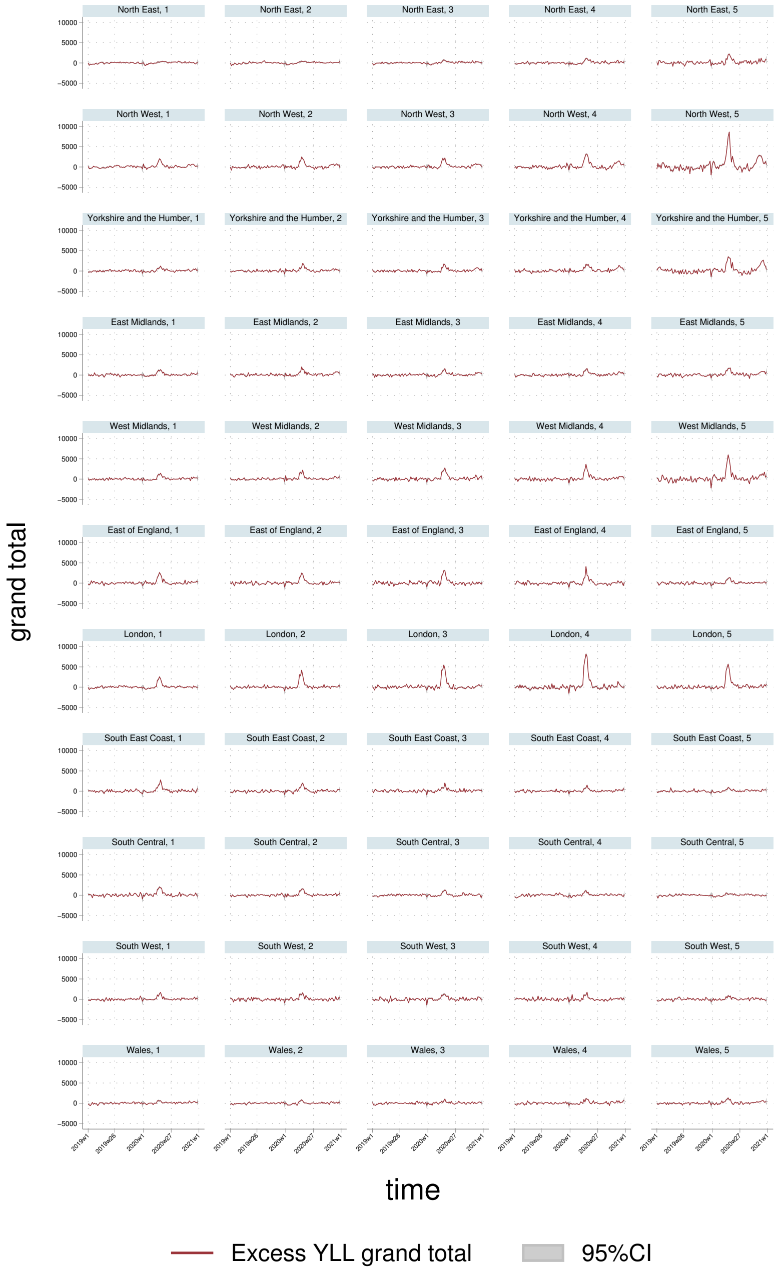

Figure 35: Observed vs Predicted Years of Life Lost trends, total deaths by regionXdeprivation, 2015-2020

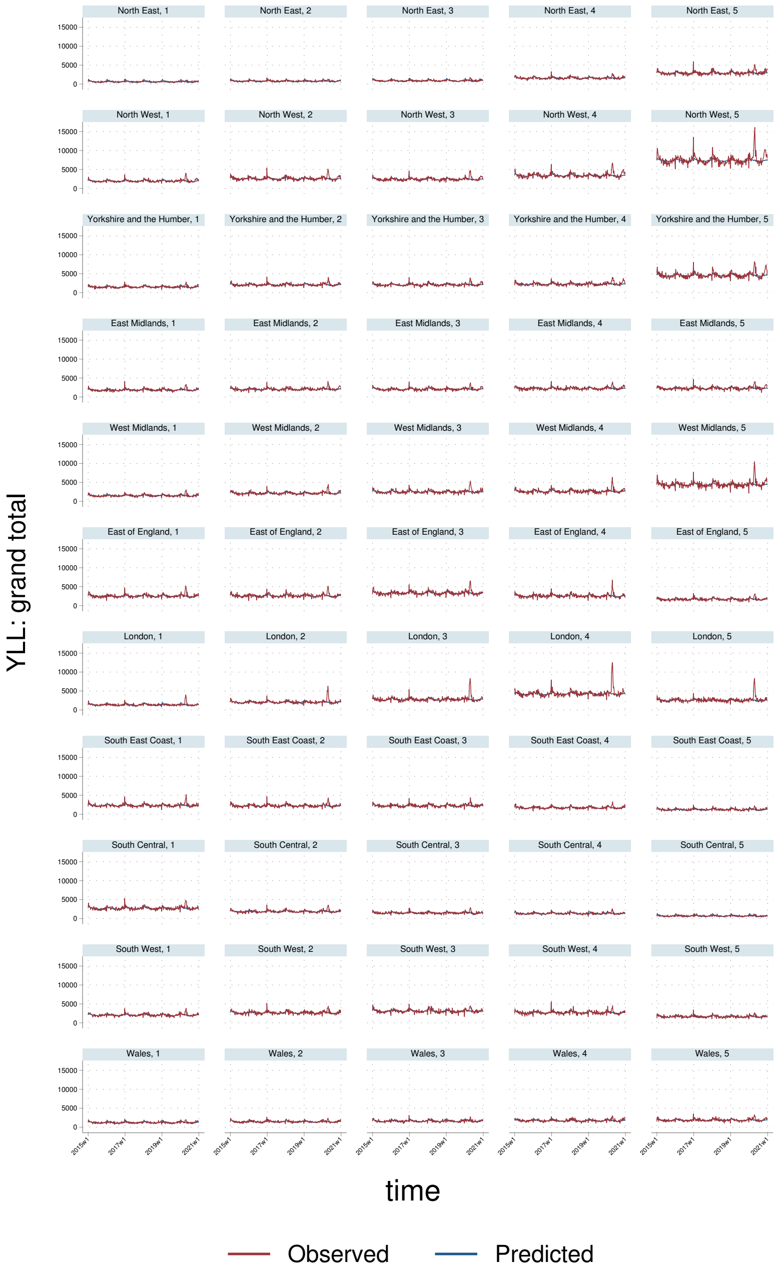

Figure 36: Observed vs Predicted Years of Life Lost trends, total deaths by regionXdeprivation, 2019-2020

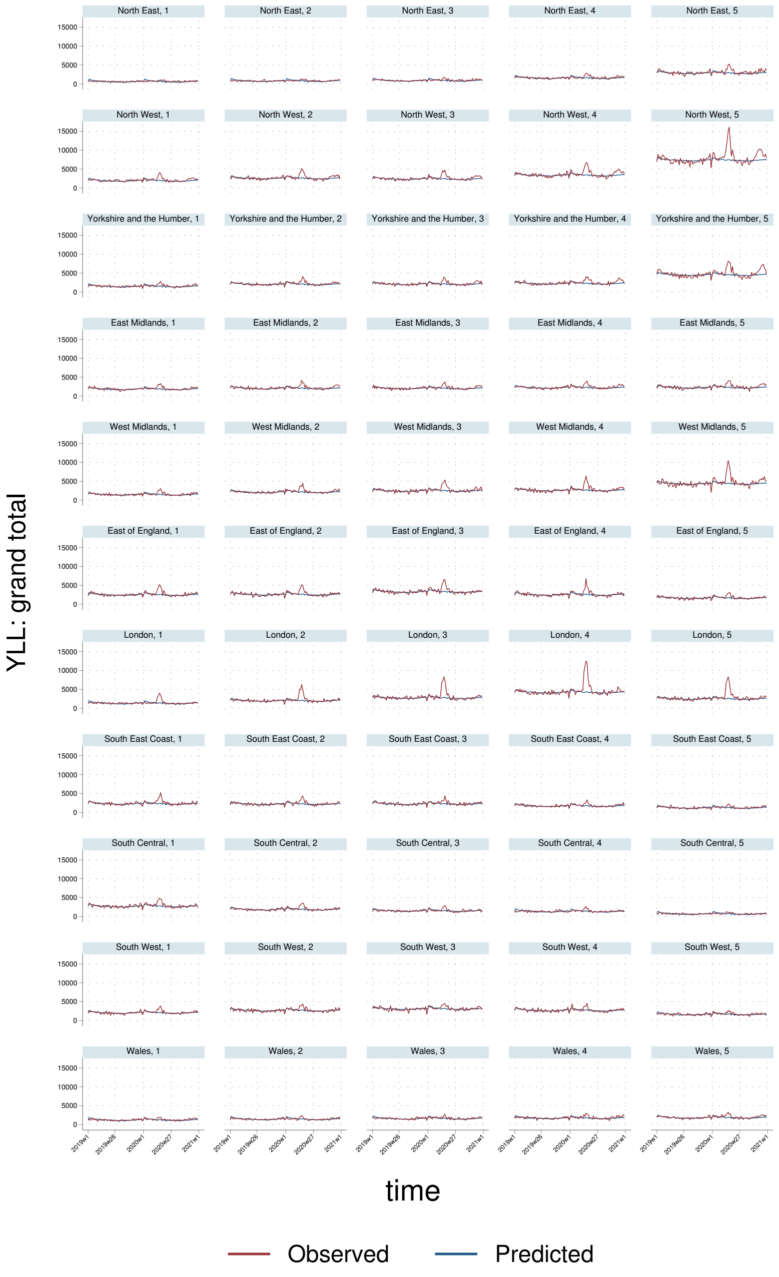

5.3 YLLs per 100,000 population

5.3.1 England-Wales aggregate

Figure 37: Years of Life Lost trend per 100,000 population, total deaths, 2015-2020

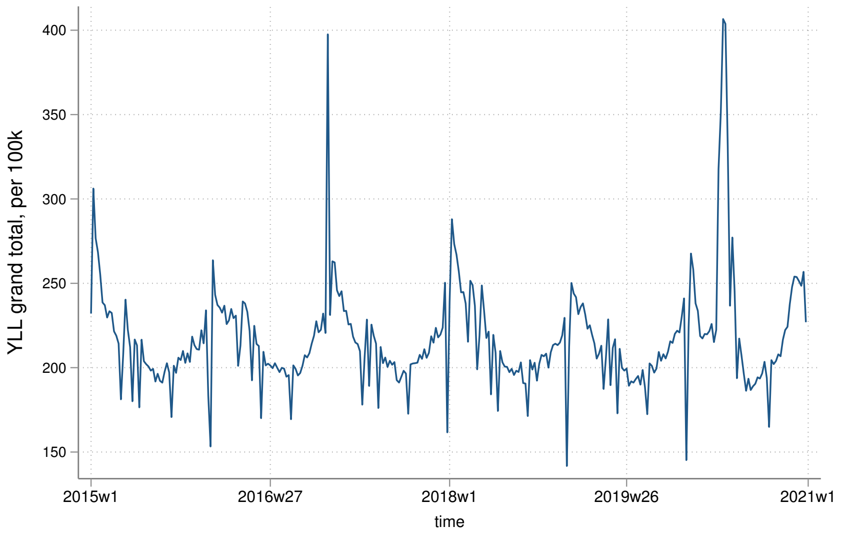

Figure 38: Years of Life Lost trend per 100,000 population, total deaths, 2019-2020

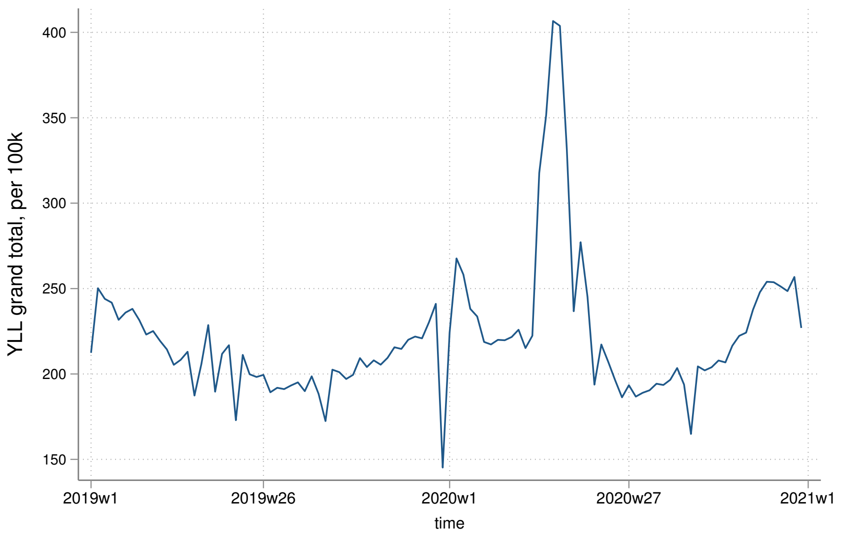

Figure 39: Excess Years of Life Lost trend per 100,000 population, total deaths, 2015-2020

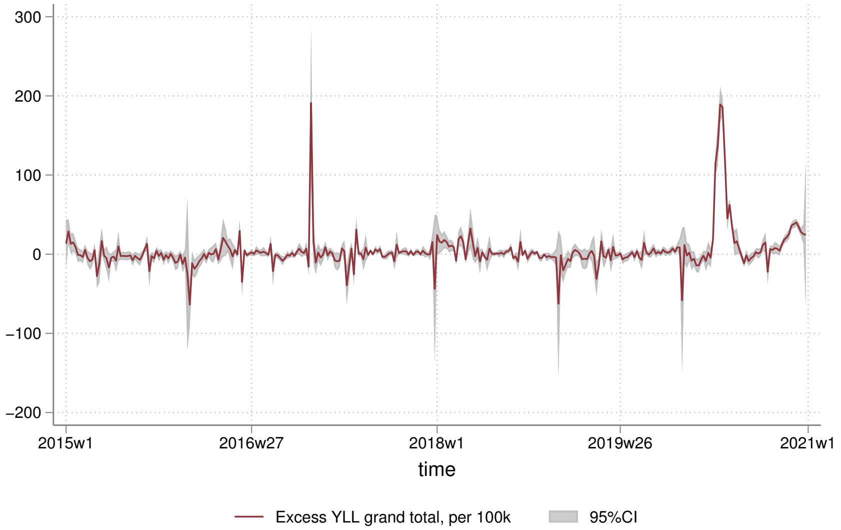

Figure 40: Excess Years of Life Lost trend per 100,000 population, total deaths, 2019-2020

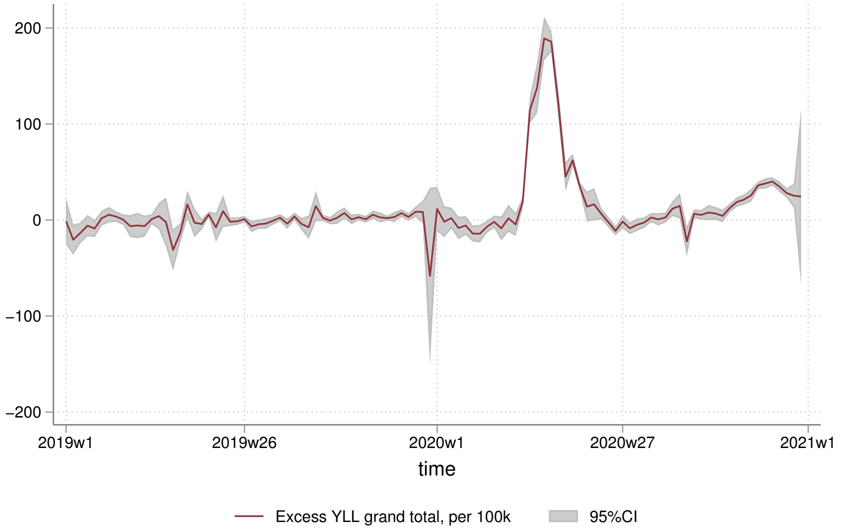

Figure 41: Observed vs Predicted Years of Life Lost trends per 100, 000 population, total deaths, 2015-2020

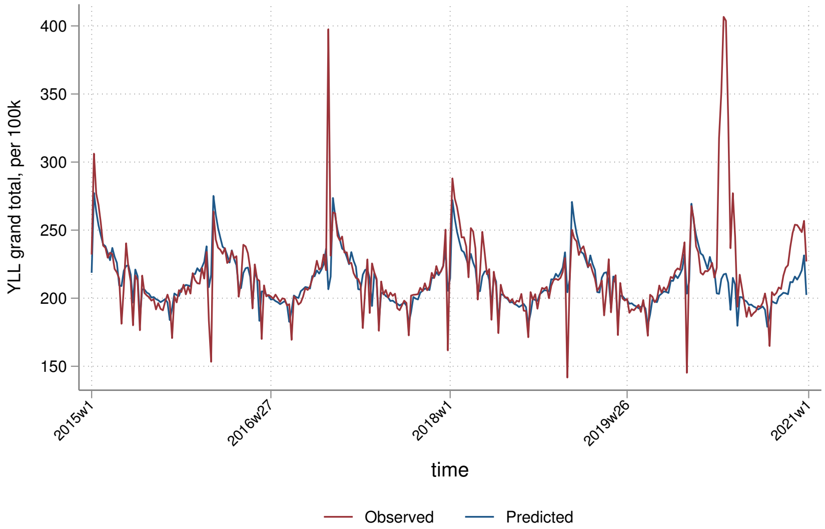

Figure 42: Observed vs Predicted Years of Life Lost trends per 100, 000 population, total deaths, 2019-2020

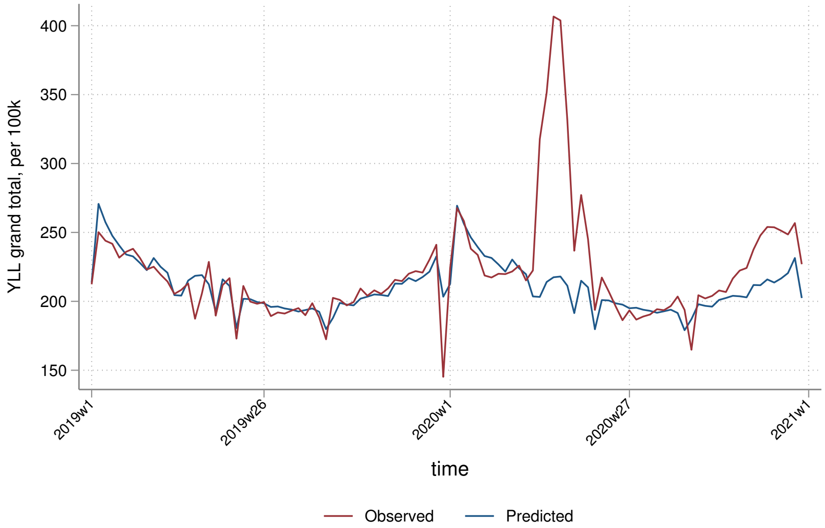

5.3.2 By sex

Figure 43: Years of Life Lost trend per 100,000 population, total deaths by sex, 2015-2020

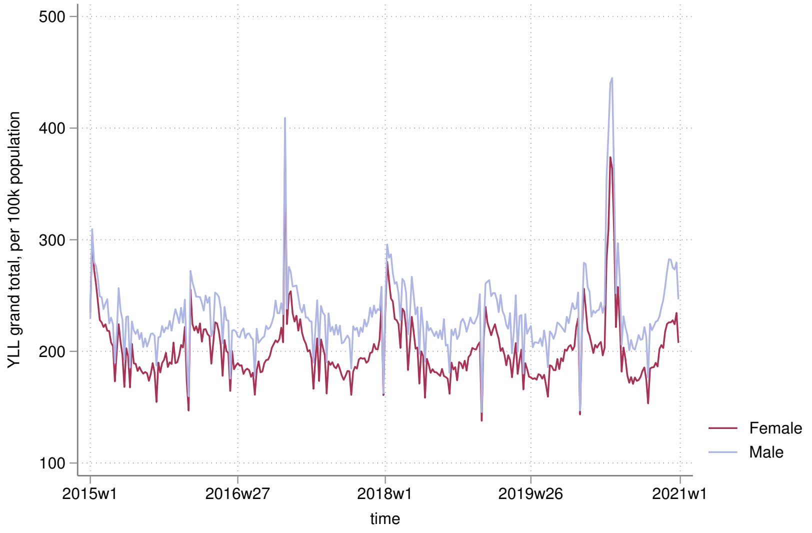

Figure 44: Years of Life Lost trend per 100,000 population, total deaths by sex, 2019-2020

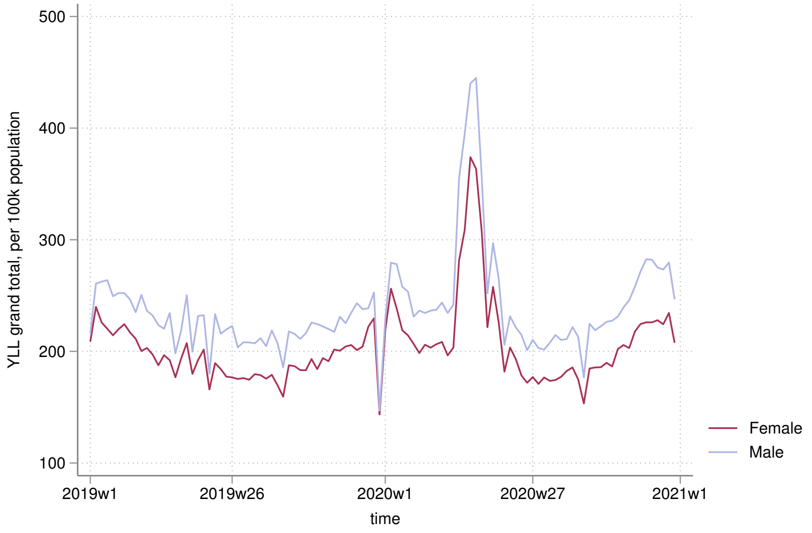

Figure 45: Excess Years of Life Lost trend per 100,000 population, total deaths by sex, 2015-2020

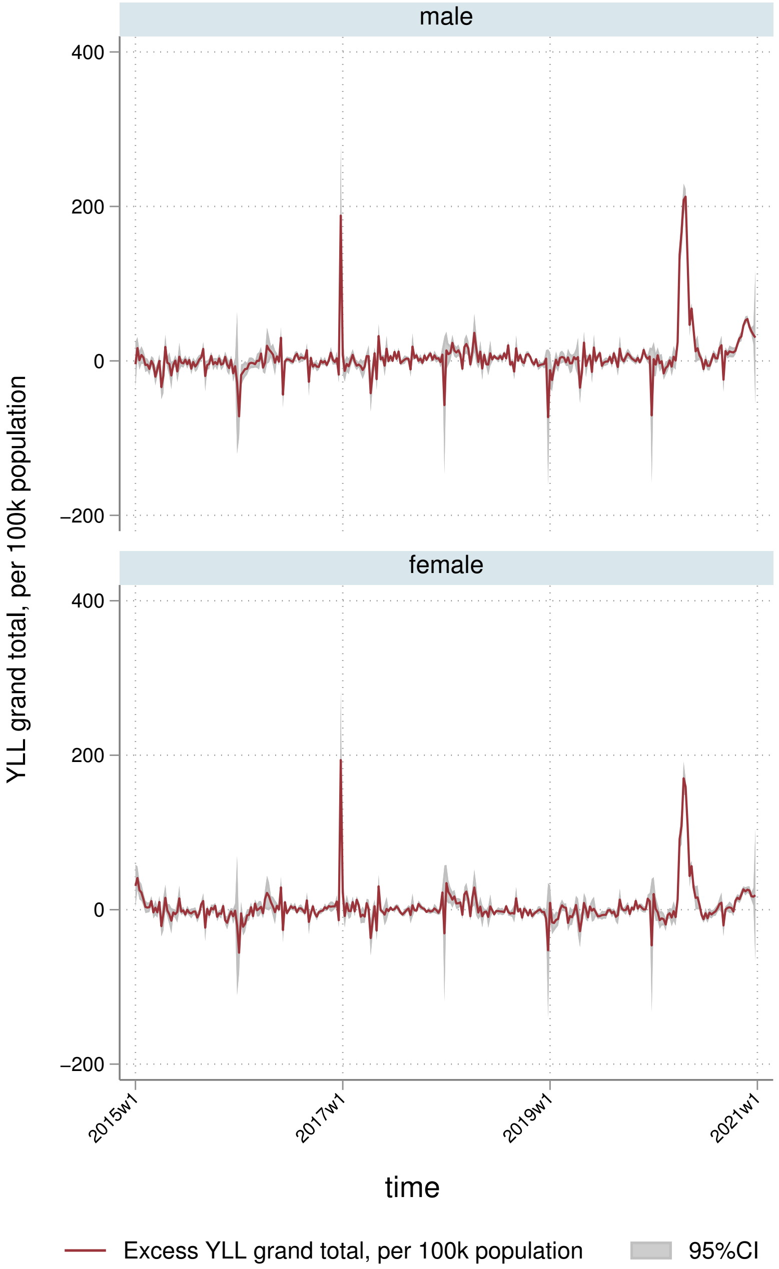

Figure 46: Excess Years of Life Lost trend per $100,000$ population, total deaths by sex, 2019-2020

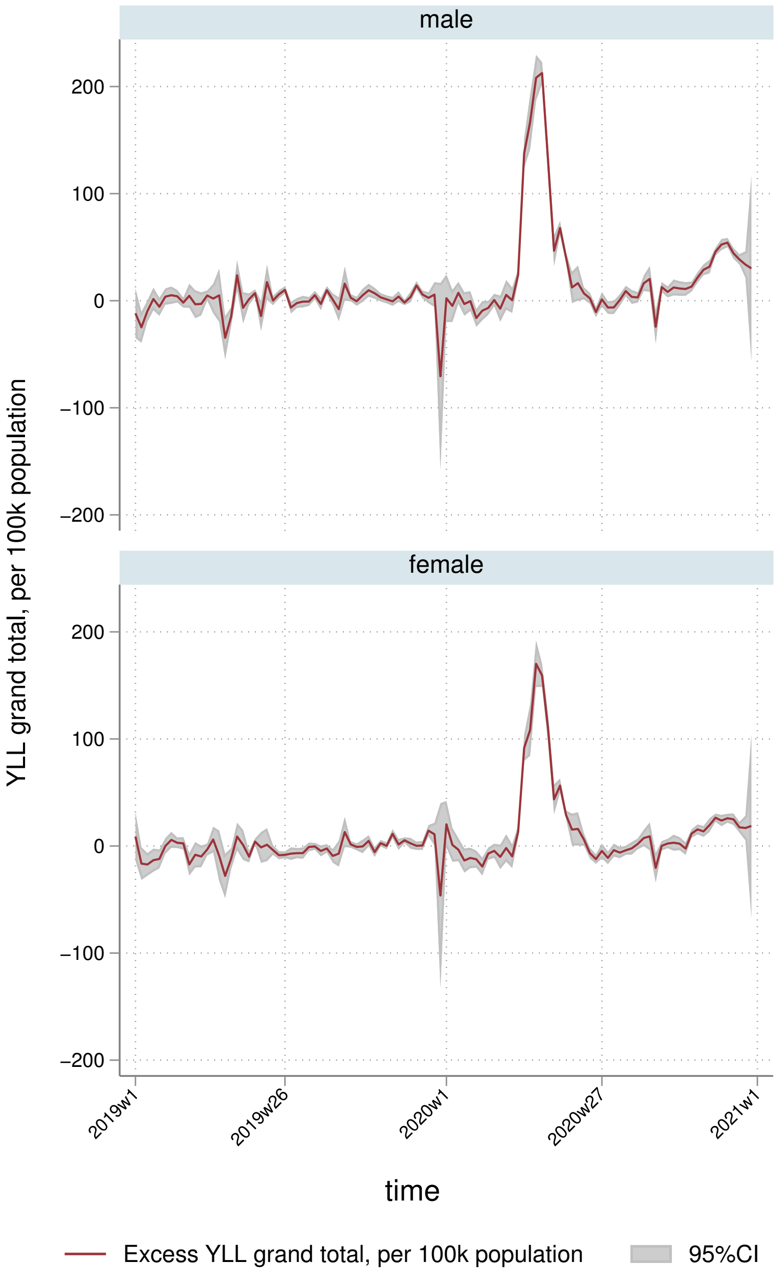

Figure 47: Observed vs Predicted Years of Life Lost trends per 100,000 population, total deaths by sex, 2015-2020

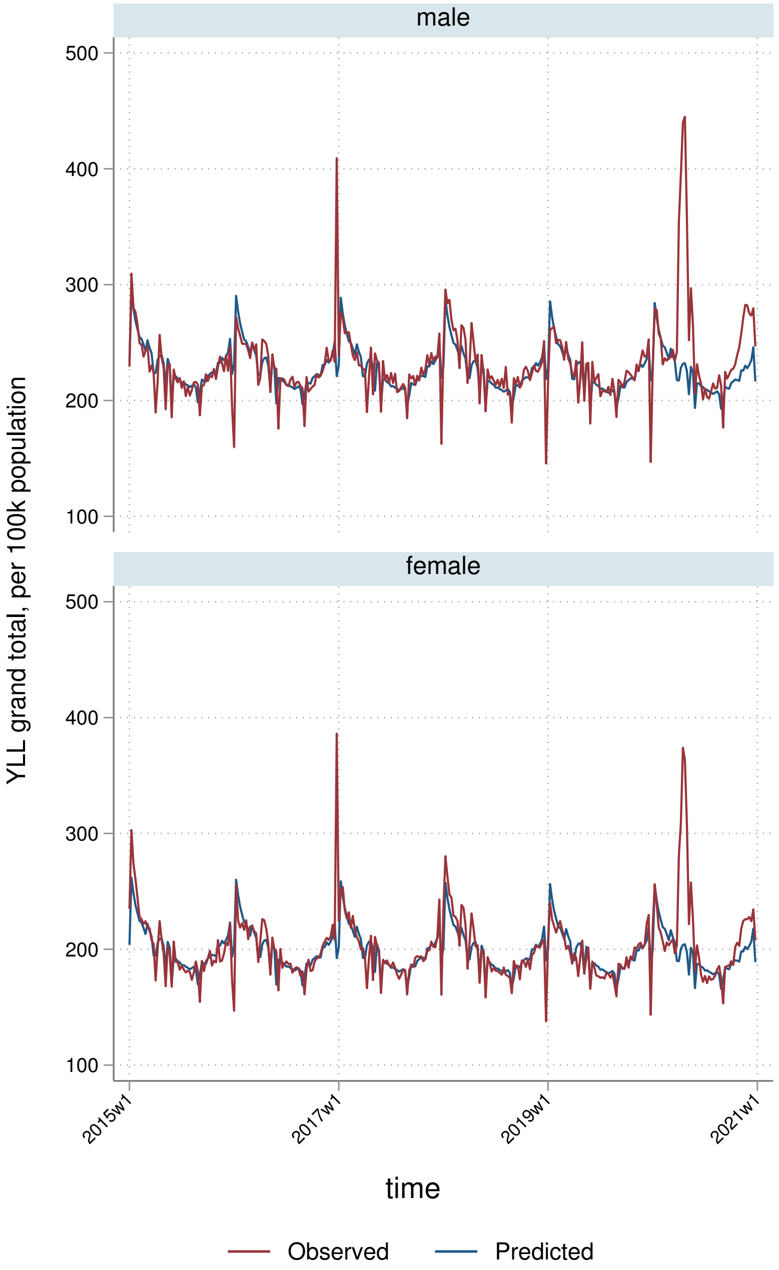

Figure 48: Observed vs Predicted Years of Life Lost trends per 100,000 population, total deaths by sex, 2019-2020

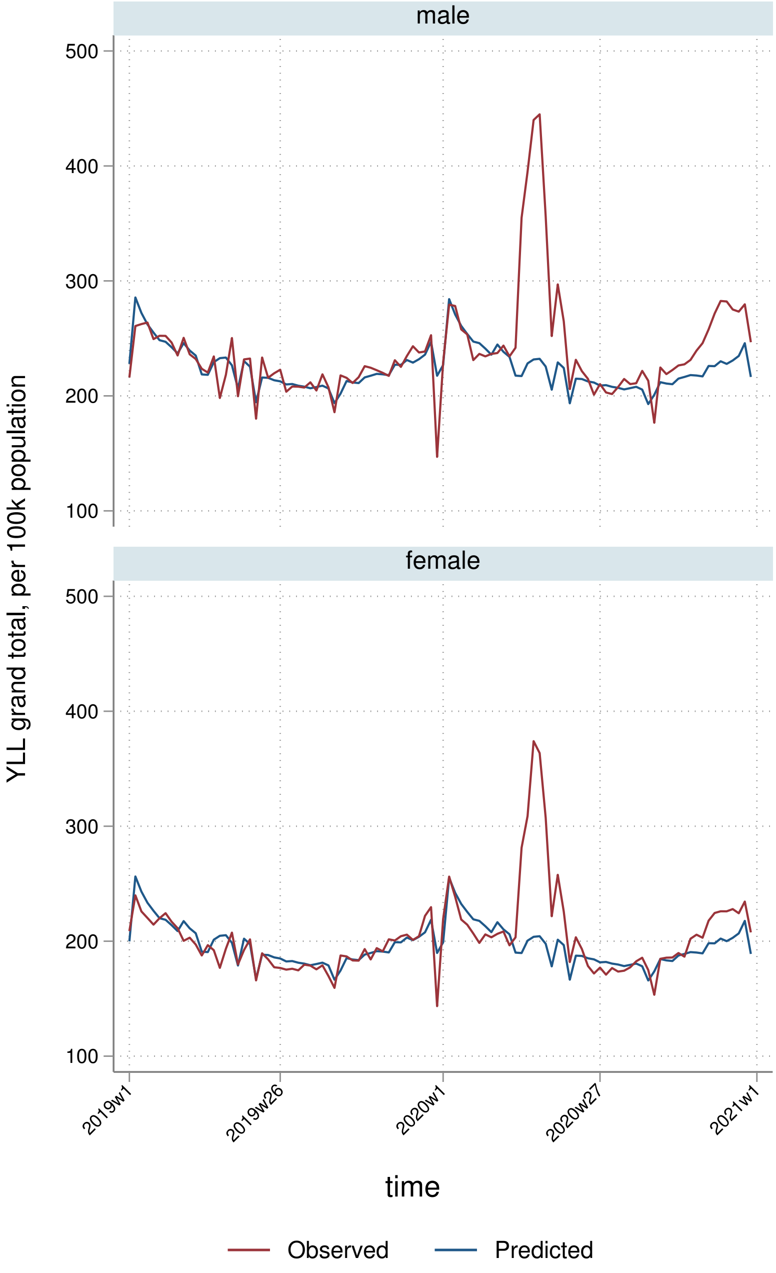

5.3.3 By deprivation quintile

Figure 49: Years of Life Lost trend per 100,000 population, total deaths by deprivation quintile, 2015-2020

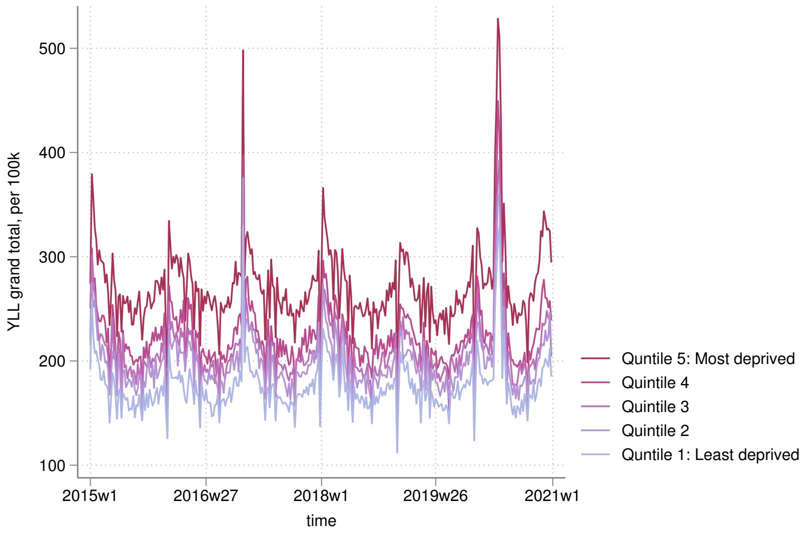

Figure 50: Years of Life Lost trend per 100,000 population, total deaths by deprivation quintile, 2019-2020

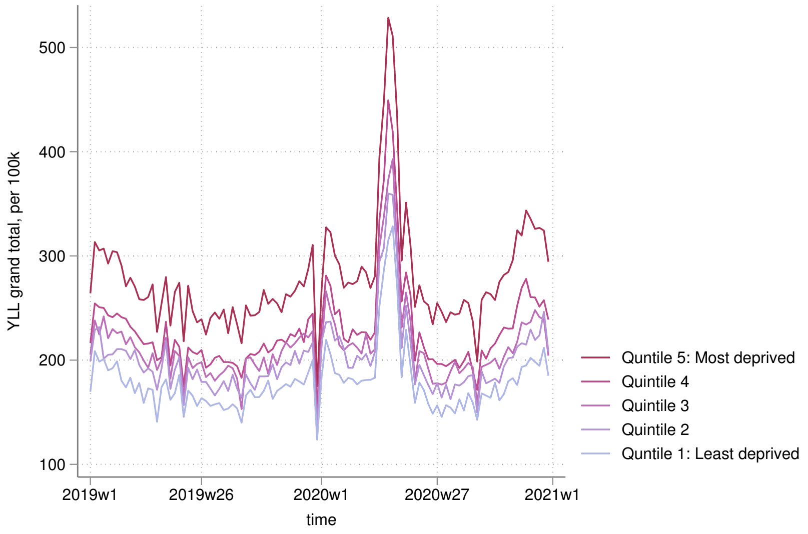

Figure 51: Excess Years of Life Lost trend per 100,000 population, total deaths by deprivation quintile, 2015-2020

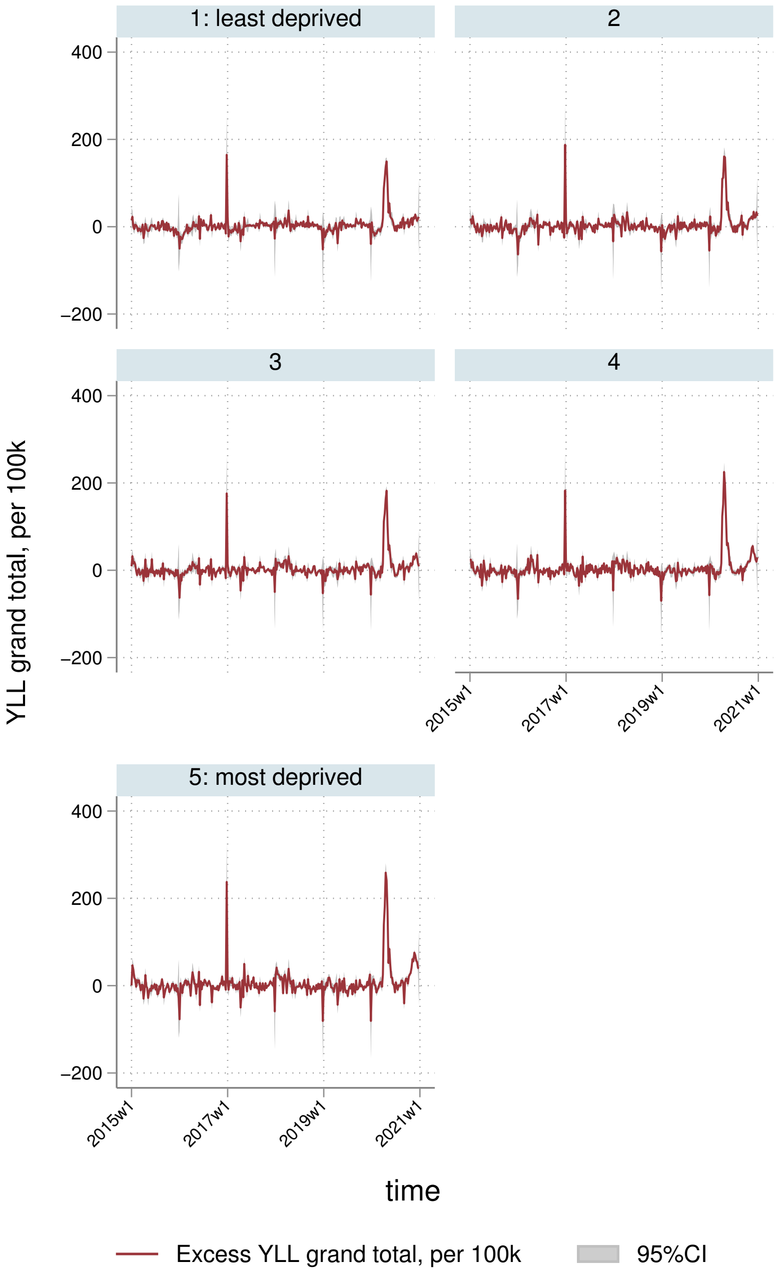

Figure 52: Excess Years of Life Lost trend per 100,000 population, total deaths by deprivation quintile, 2019-2020

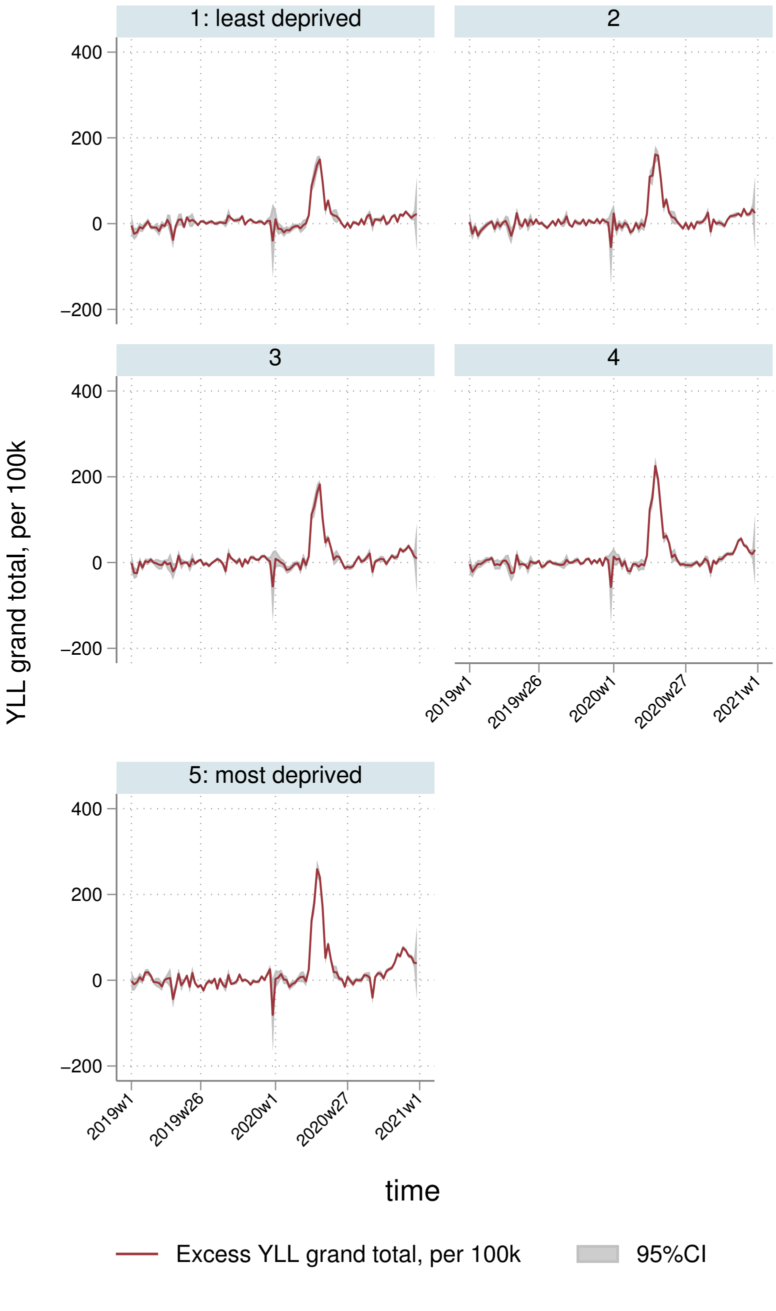

Figure 53: Observed vs Predicted Years of Life Lost trends per 100,000 population, total deaths by deprivation quintile, 2015-2020

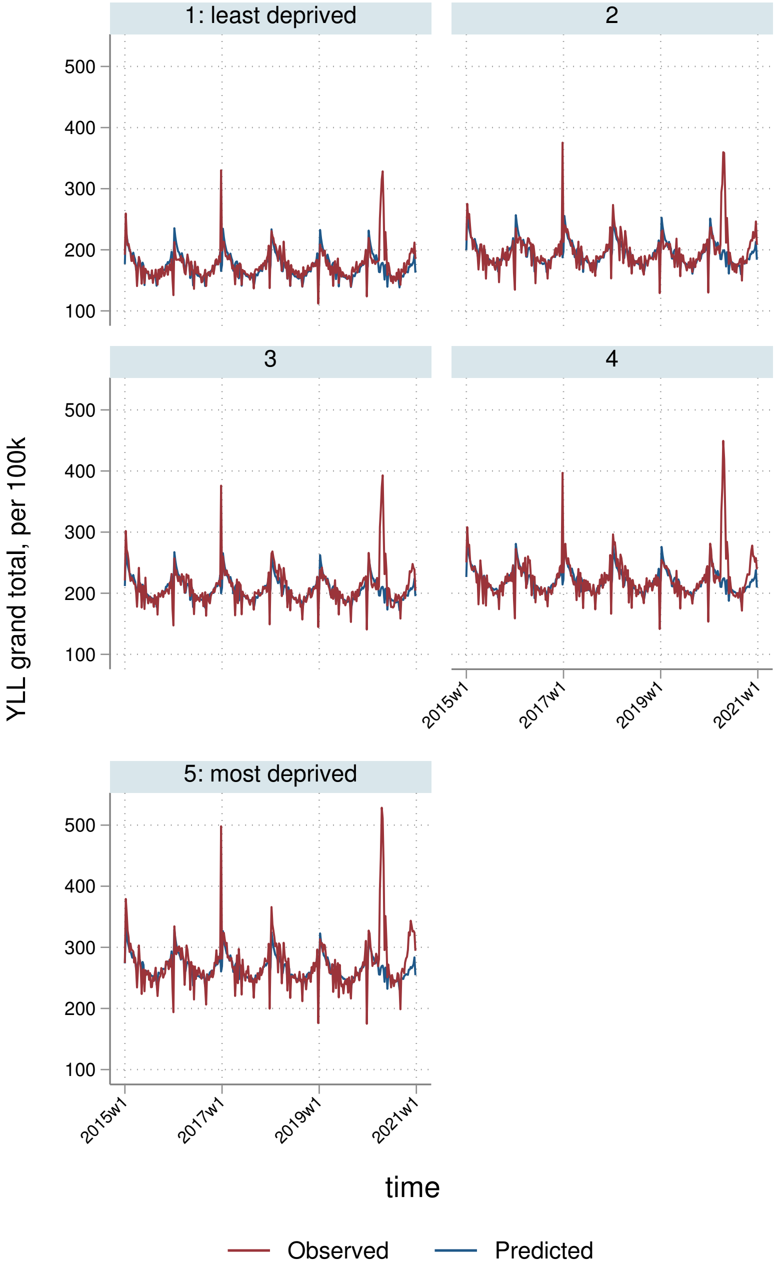

Figure 54: Observed vs Predicted Years of Life Lost trends per 100, 000 population, total deaths by deprivation quintile, 2019-2020

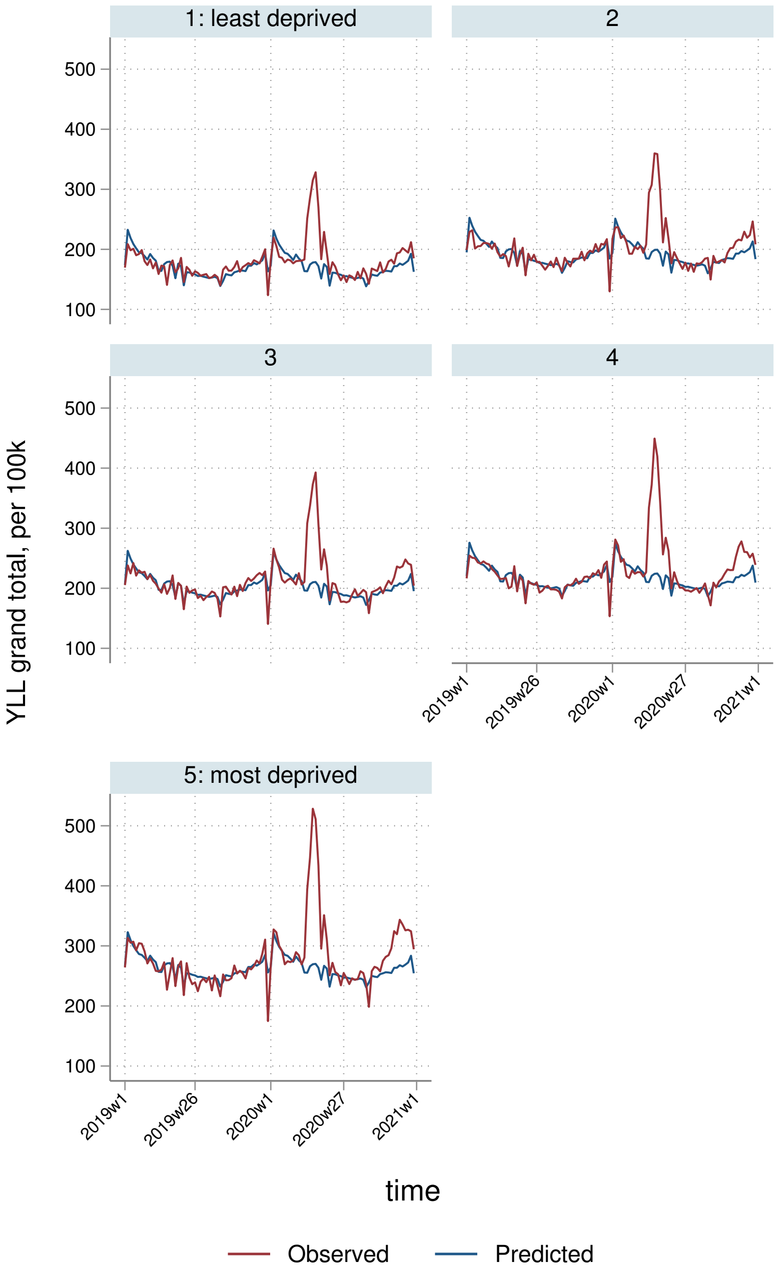

5.3.4 By Strategic Health Authority

Figure 55: Years of Life Lost trend per 100,000 population, total deaths by region, 2015-2020

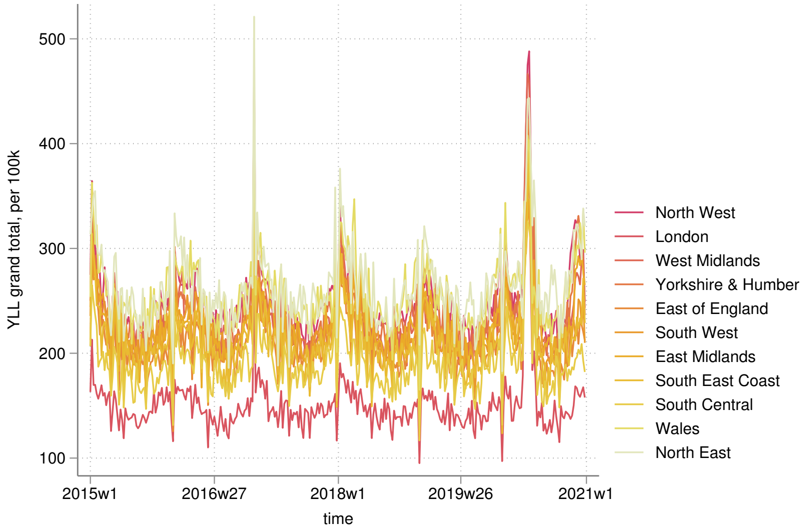

Figure 56: Years of Life Lost trend per 100,000 population, total deaths by region, 2019-2020

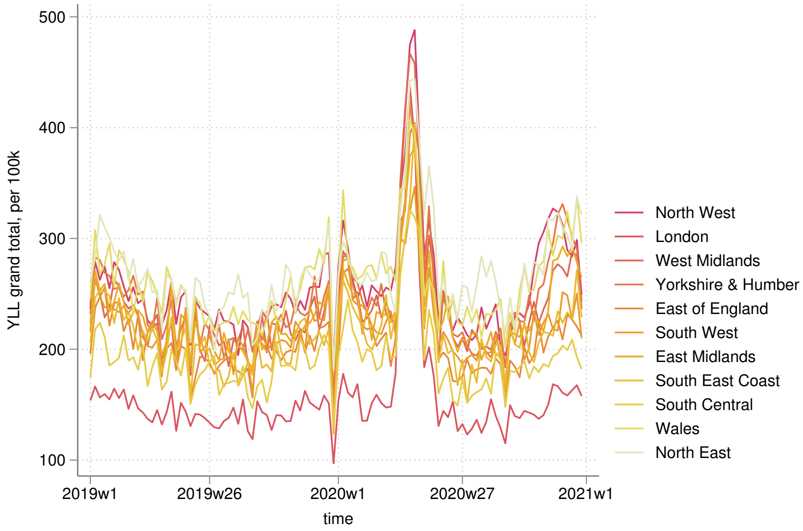

Figure 57: Excess Years of Life Lost trend per 100,000 population, total deaths by region, 2015-2020

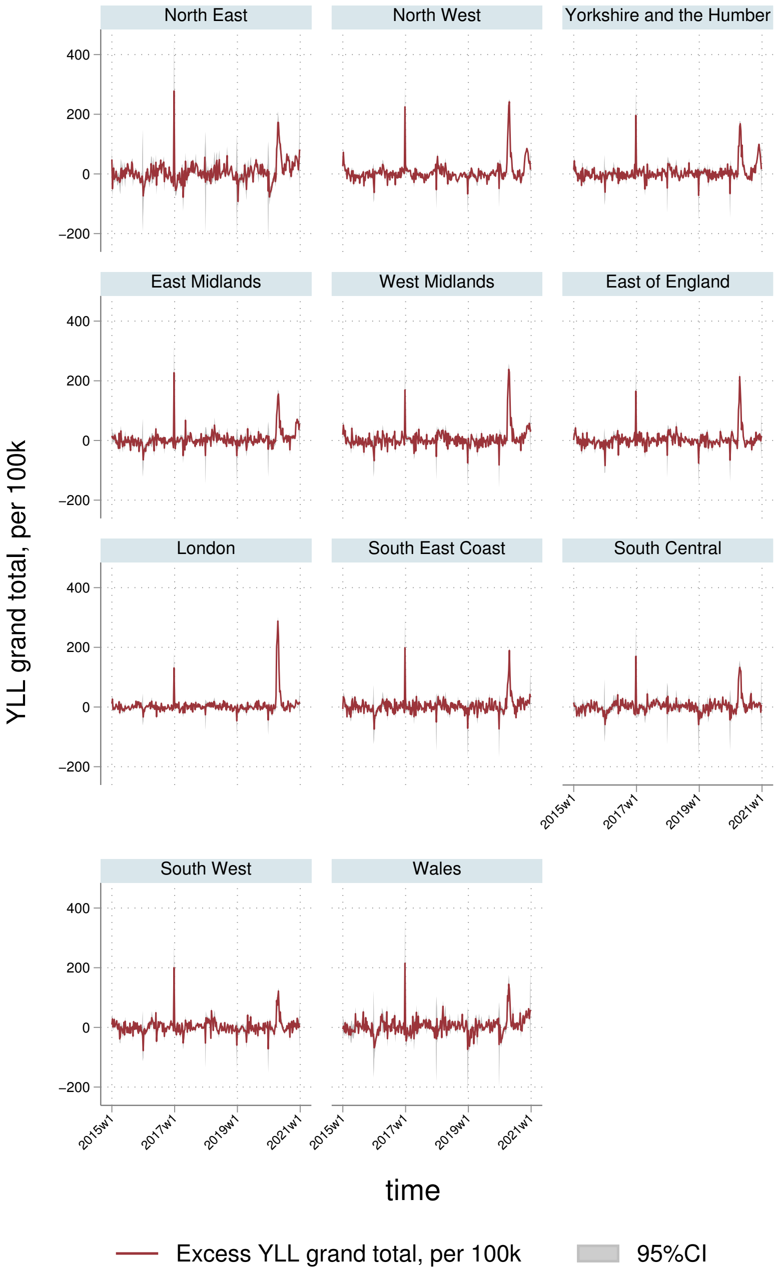

Figure 58: Excess Years of Life Lost trend per 100,000 population, total deaths by region, 2019-2020

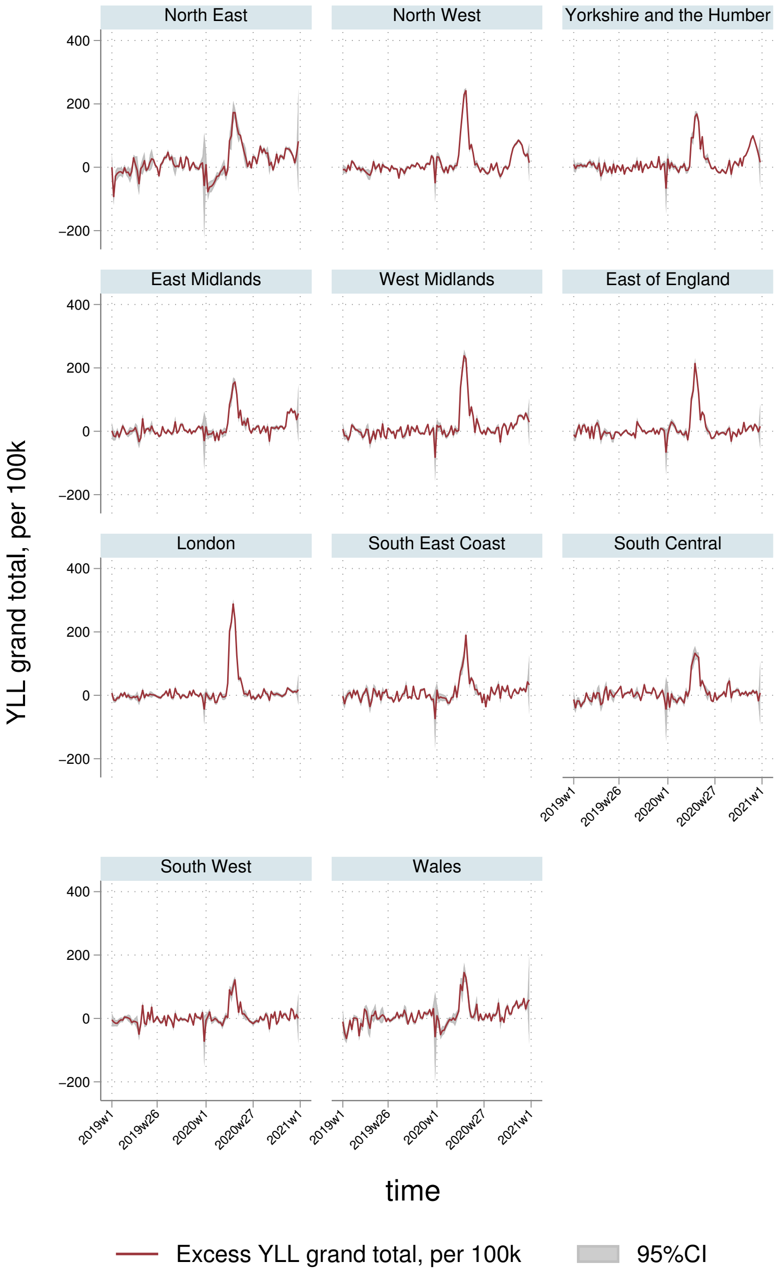

Figure 59: Observed vs Predicted Years of Life Lost trends per 100,000 population, total deaths by region, 2015-2020

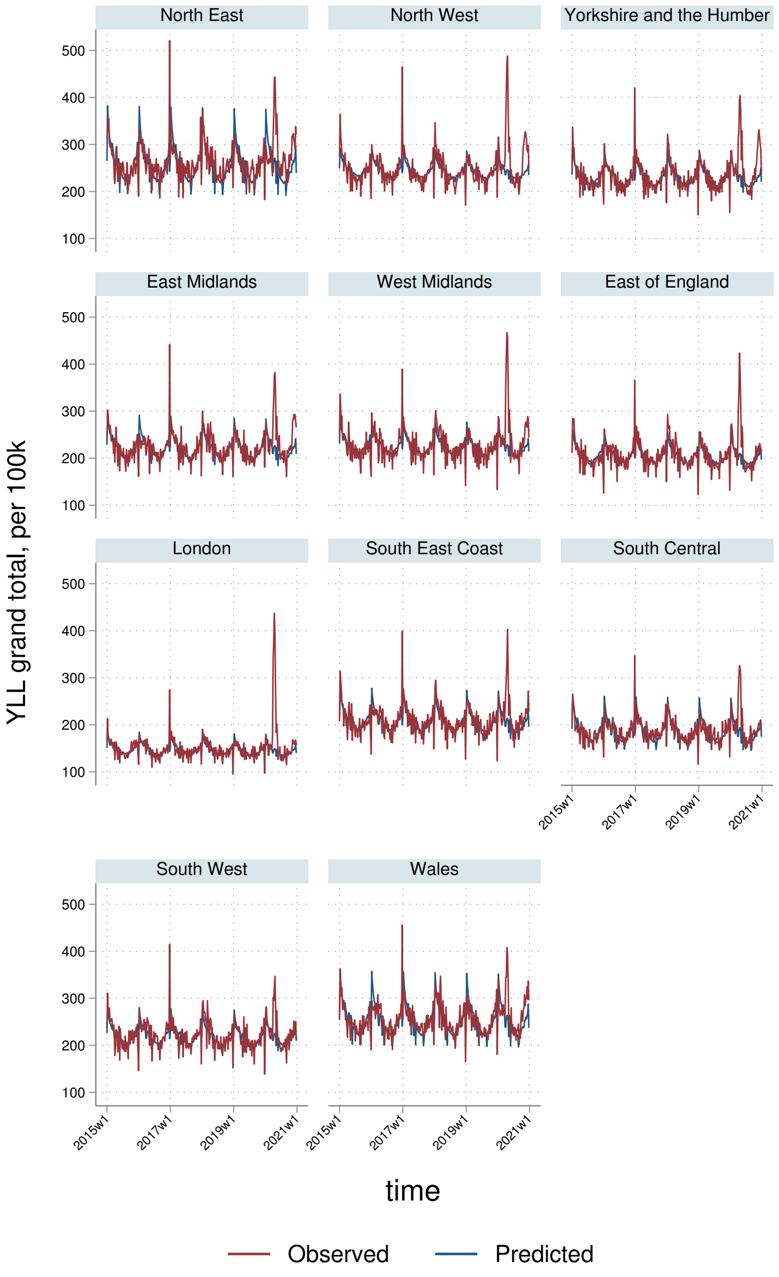

Figure 60: Observed vs Predicted Years of Life Lost trends per 100, 000 population, total deaths by region, 2019-2020

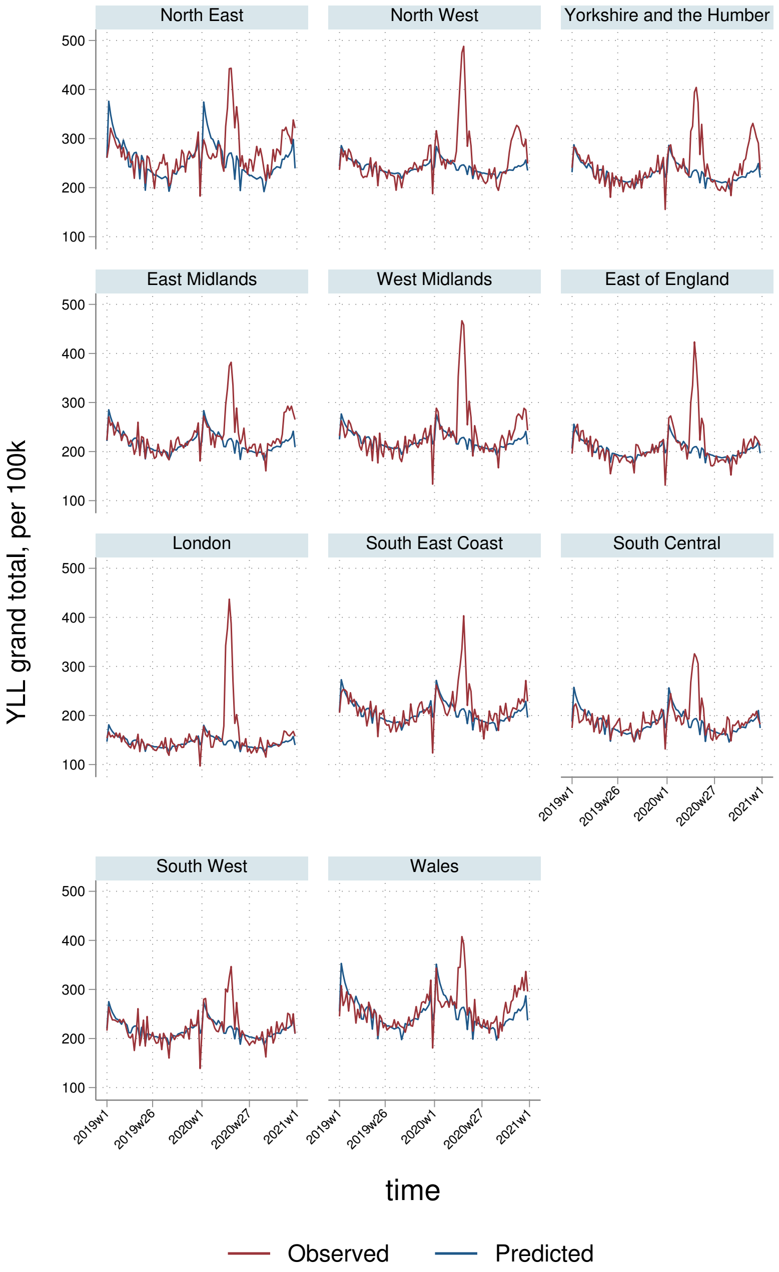

5.3.5 By deprivation quintile & Strategic Health Authority

Figure 61: Years of Life Lost trend per 100,000 population, total deaths by regionXdeprivation, 2015-2020

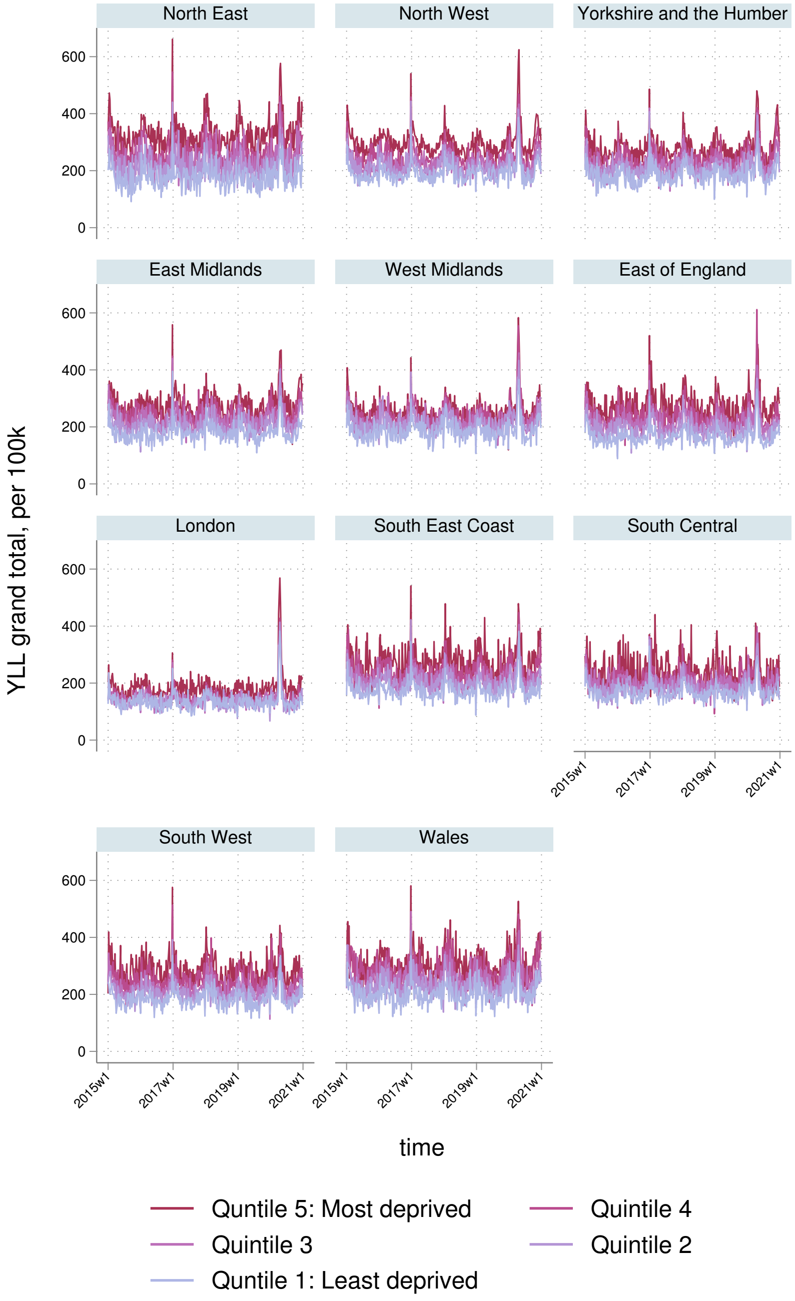

Figure 62: Years of Life Lost trend per 100,000 population, total deaths by region, 2019-2020

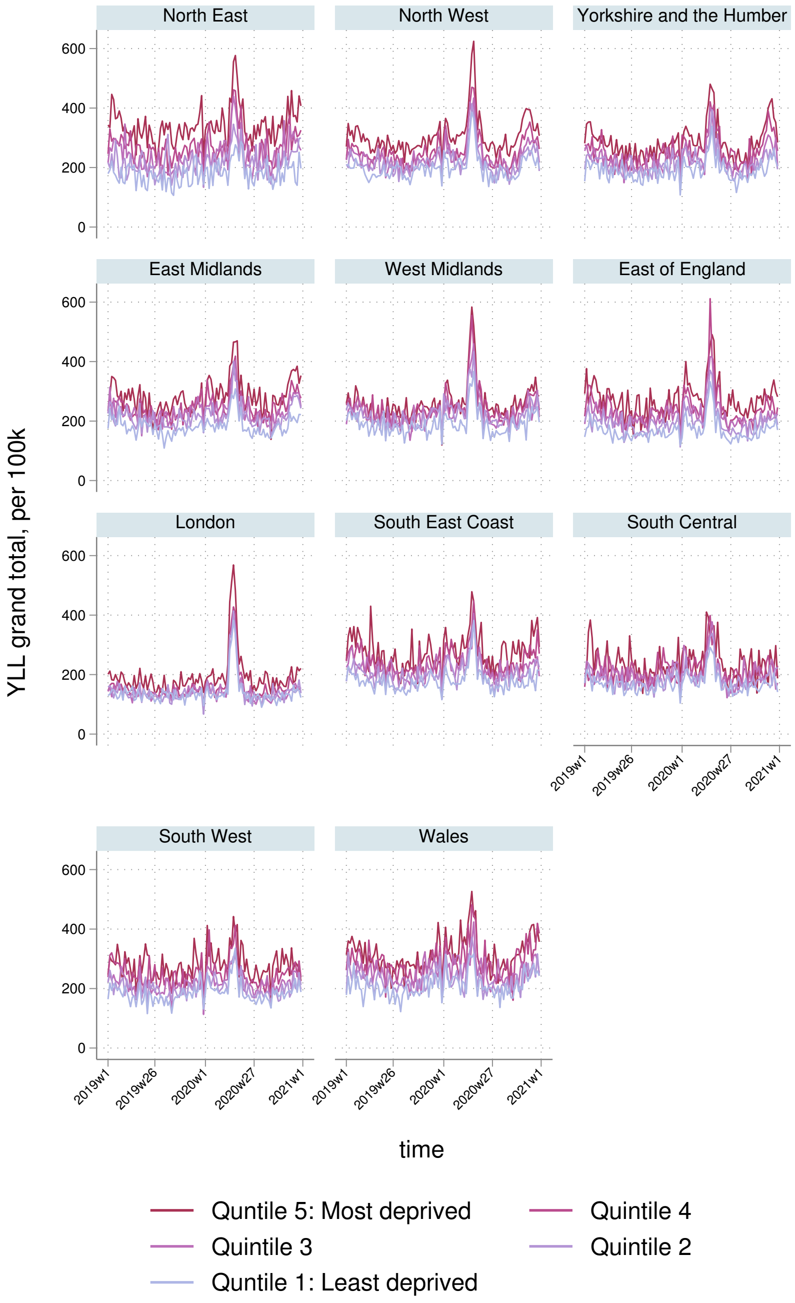

Figure 63: Excess Years of Life Lost trend per 100,000 population, total deaths by regionXdeprivation, 2015-2020

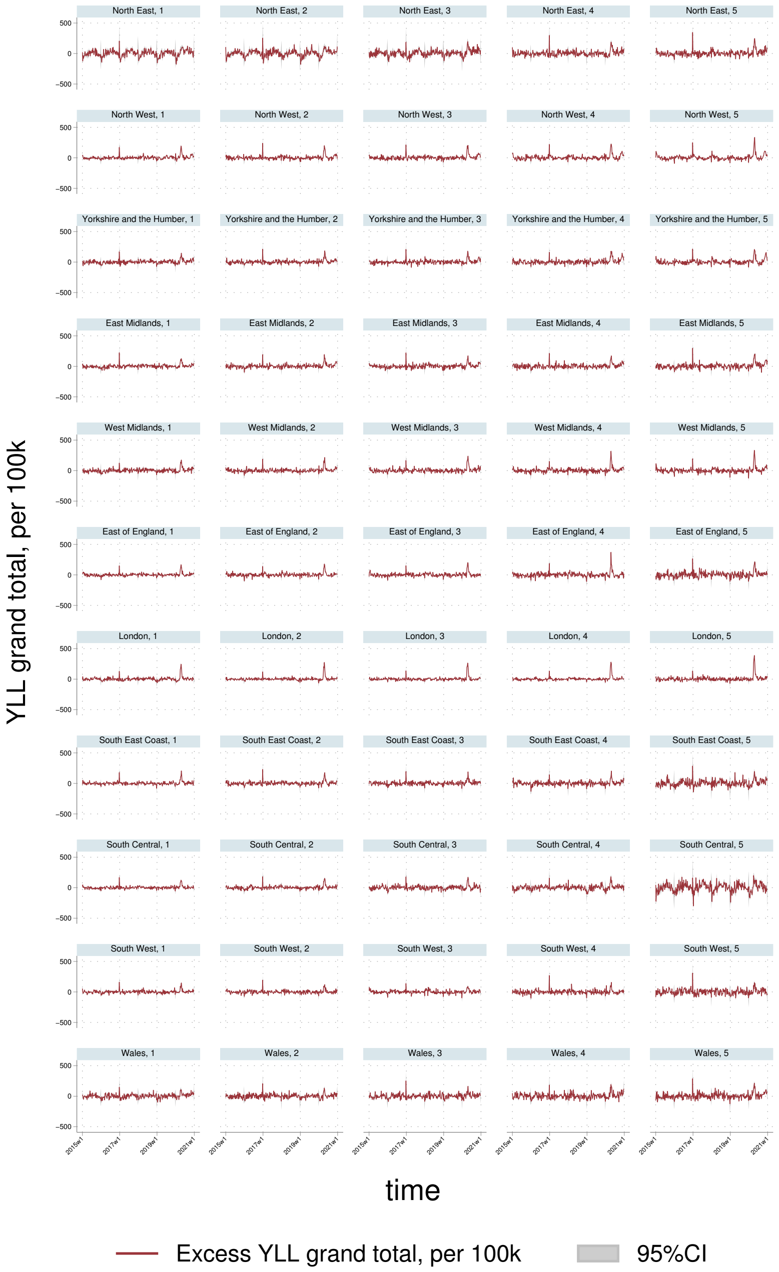

Figure 64: Excess Years of Life Lost trend per 100,000 population, total deaths by regionXdeprivation, 2019-2020

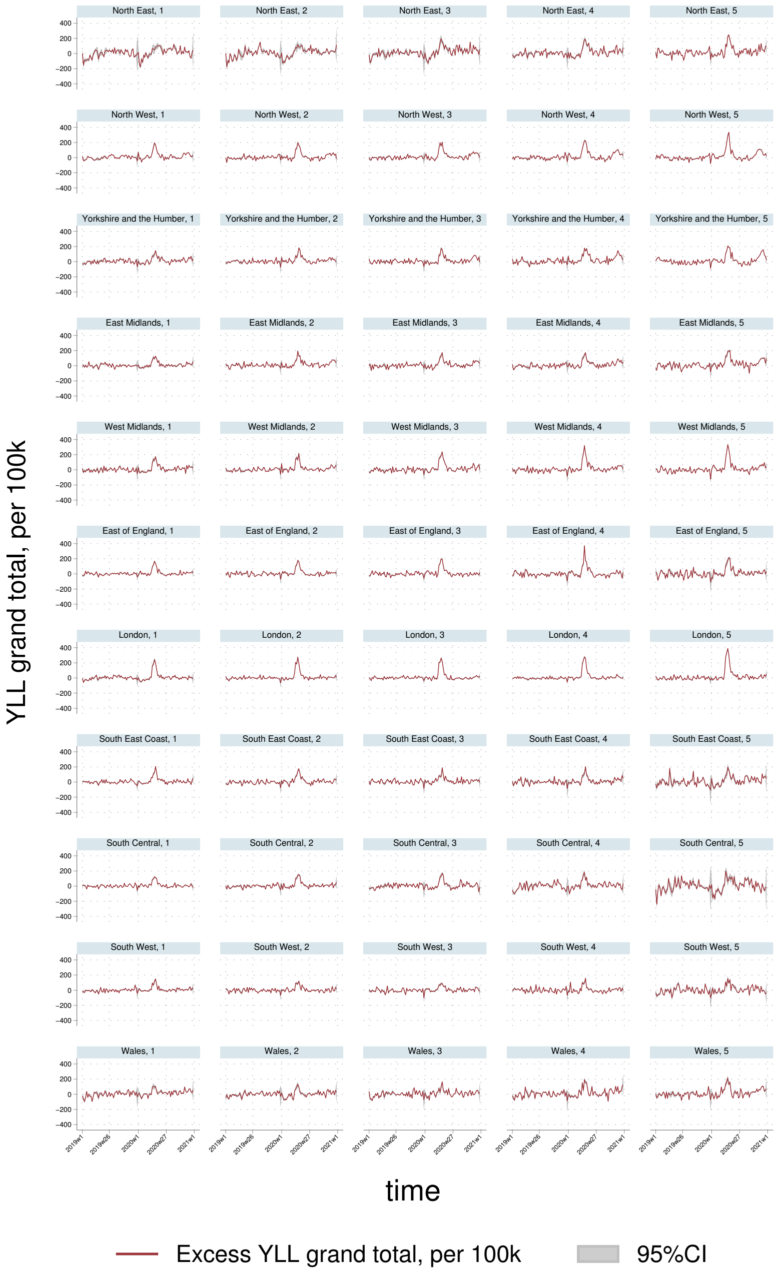

Figure 65: Observed vs Predicted Years of Life Lost trends per 100,000 population, total deaths by regionXdeprivation, 2015-2020

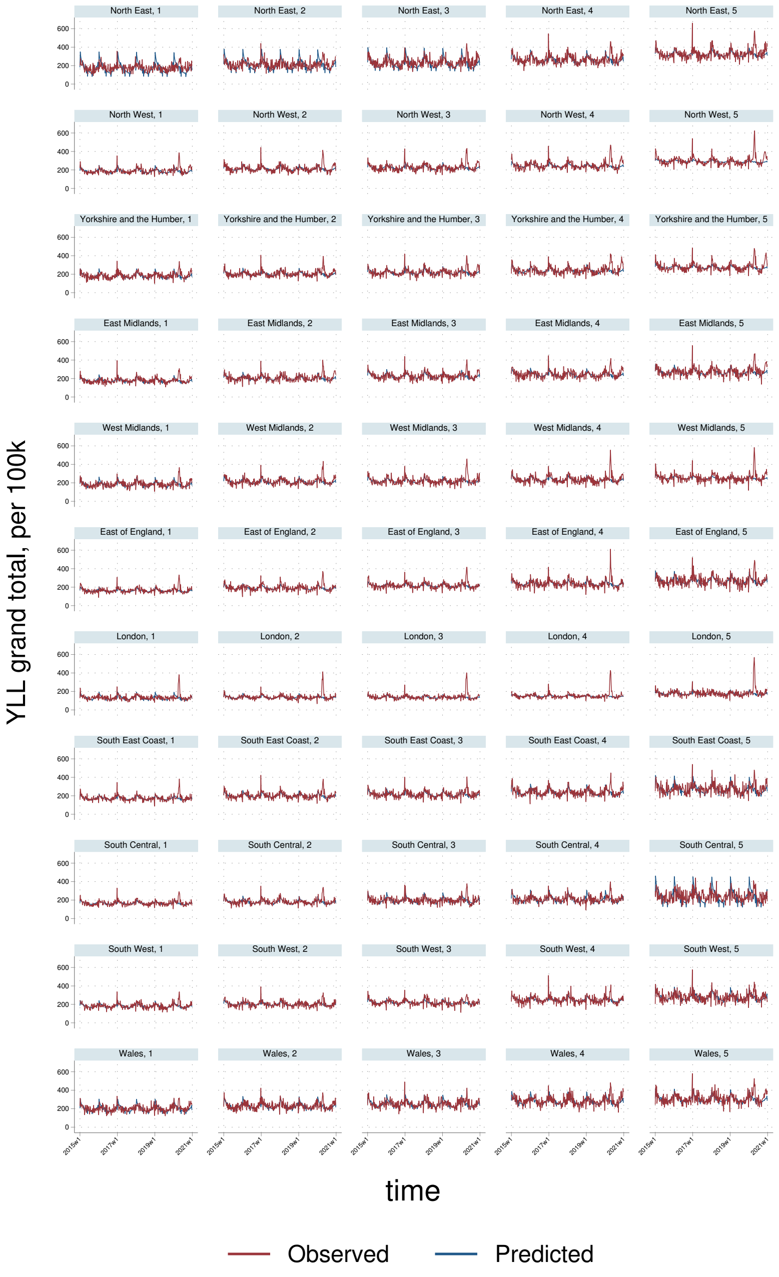

Figure 66: Observed vs Predicted Years of Life Lost trends per 100,000 population, total deaths by regionXdeprivation, 2019-2020

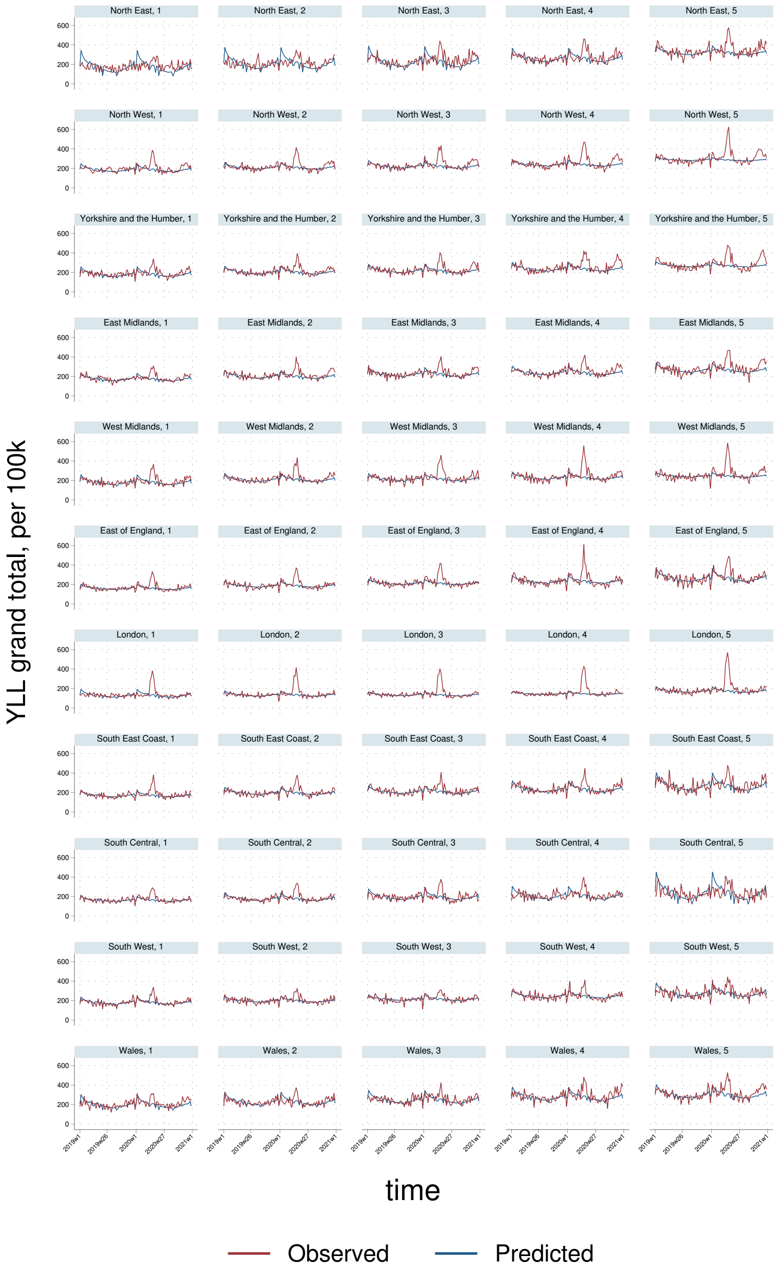

6 Direct

6.1 AASMRs

Figure 67: Age-standardised mortality trend, direct deaths, 2015-2020

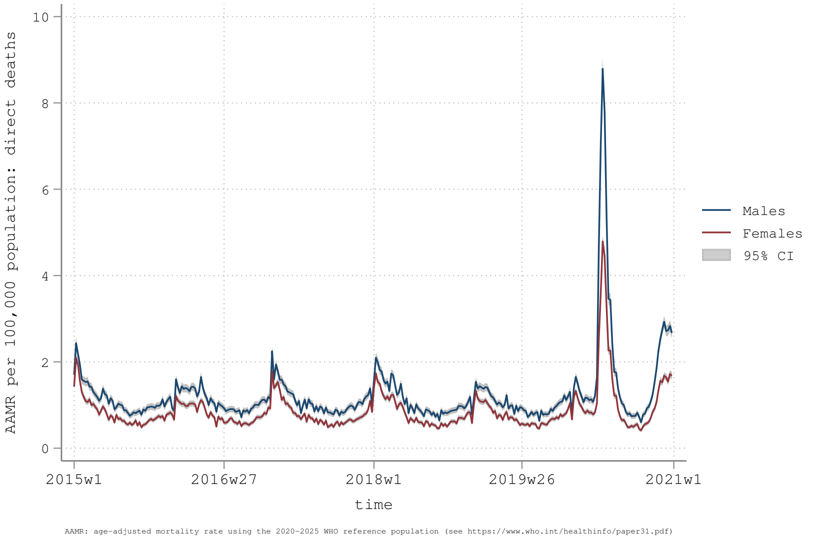

Figure 68: Age-standardised mortality trend, direct deaths, 2019-2020

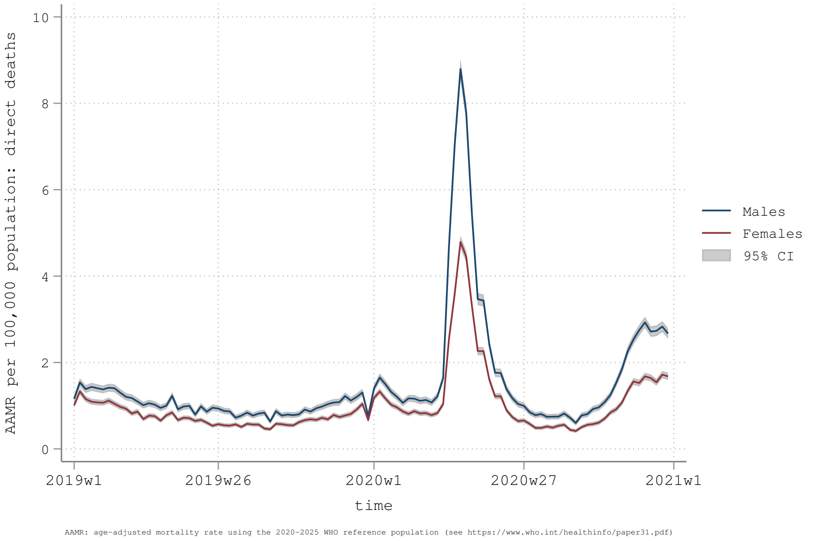

Figure 69: Age-standardised mortality trend, direct deaths by deprivation quintile, 2015-2020

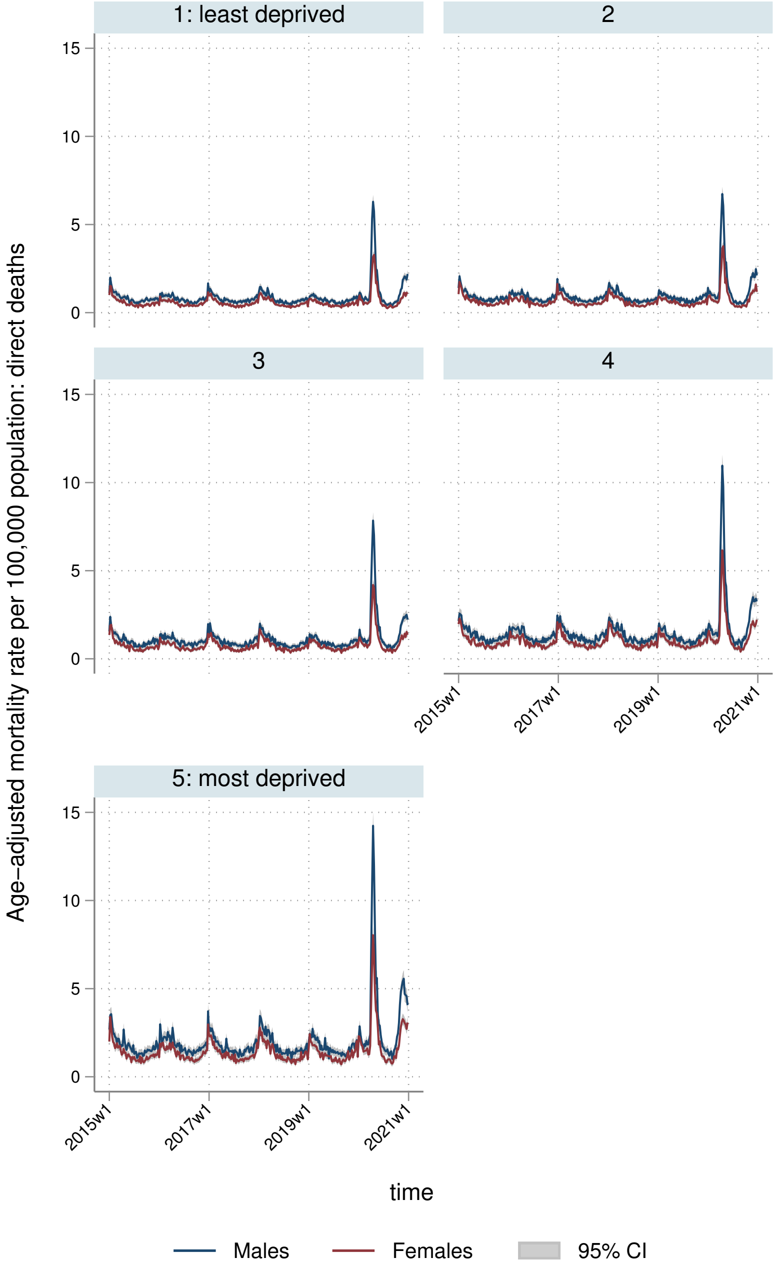

Figure 70: Age-standardised mortality trend, direct deaths by deprivation quintile, 2019-2020

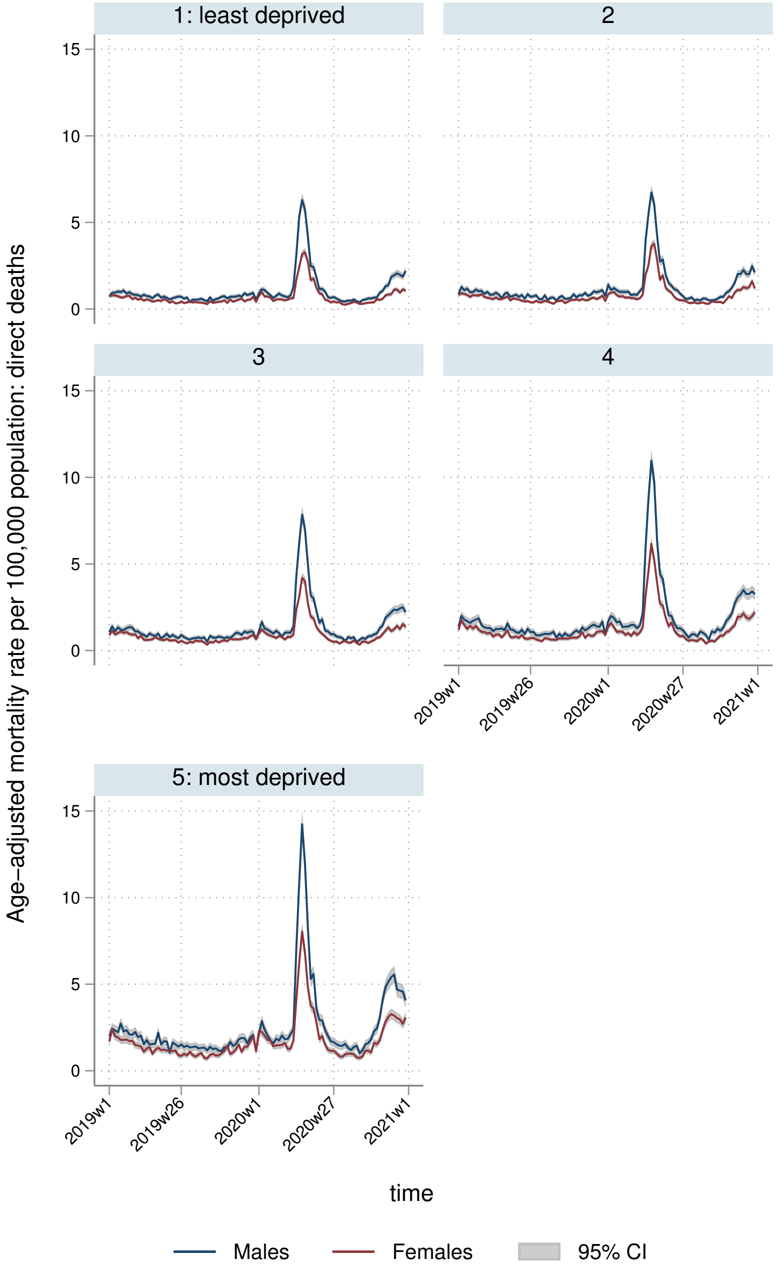

Figure 71: Age-standardised mortality trend, direct deaths by region, 2015-2020

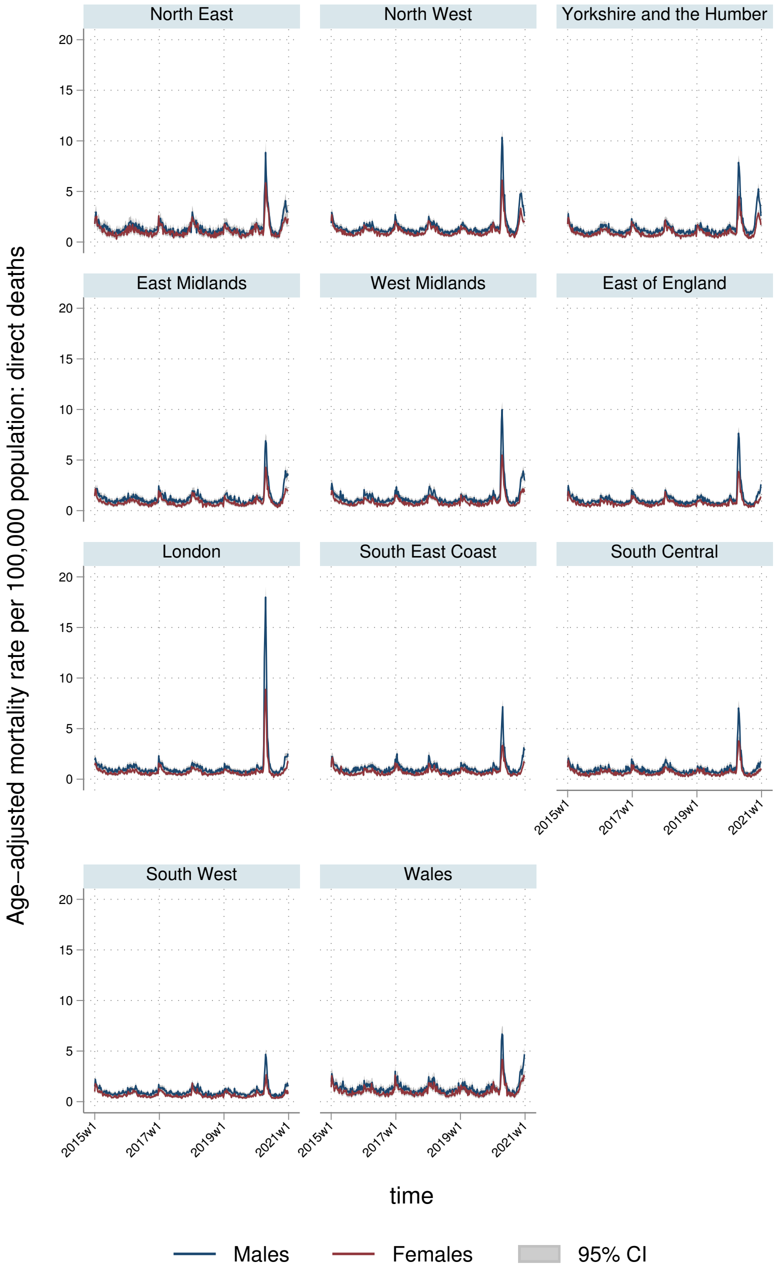

Figure 72: Age-standardised mortality trend, direct deaths by region, 2019-2020

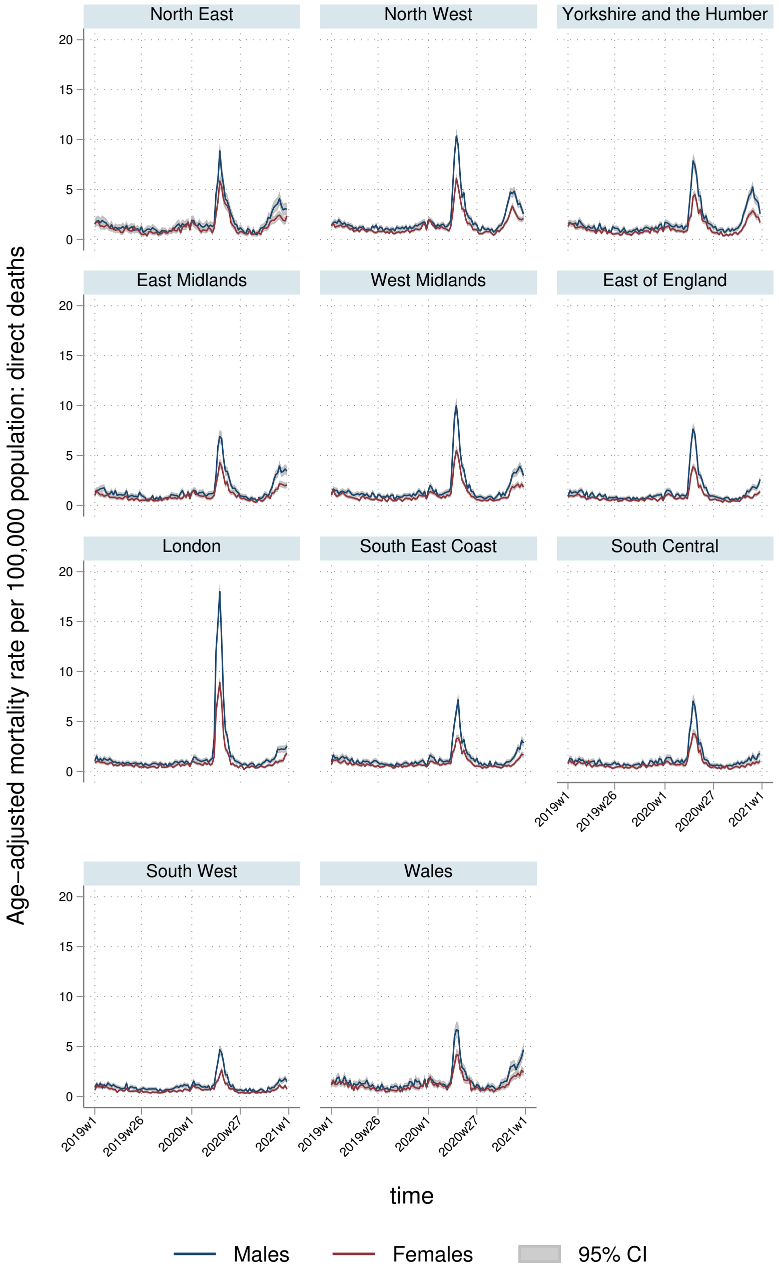

6.2 YLLs

6.2.1 England-Wales aggregate

Figure 73: Years of Life Lost trend, direct deaths, 2015-2020

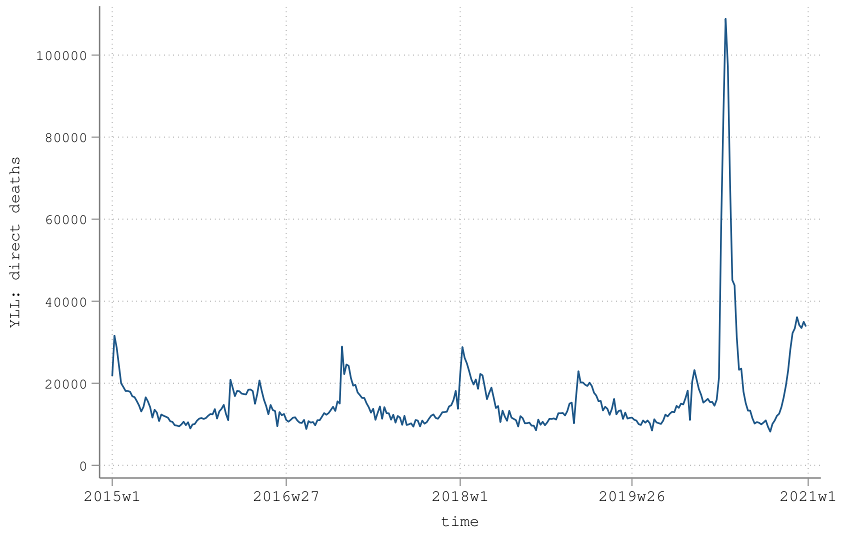

Figure 74: Years of Life Lost trend, direct deaths, 2019-2020

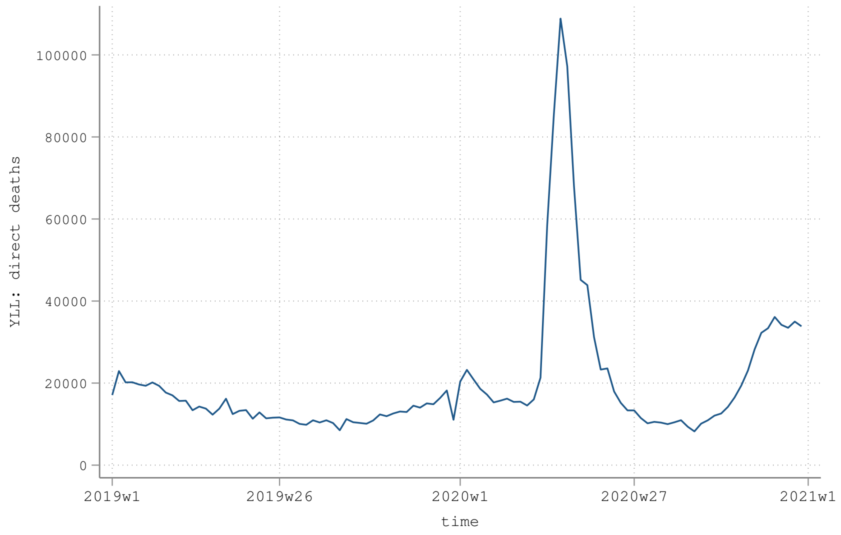

Figure 75: Excess Years of Life Lost trend, direct deaths, 2015-2020

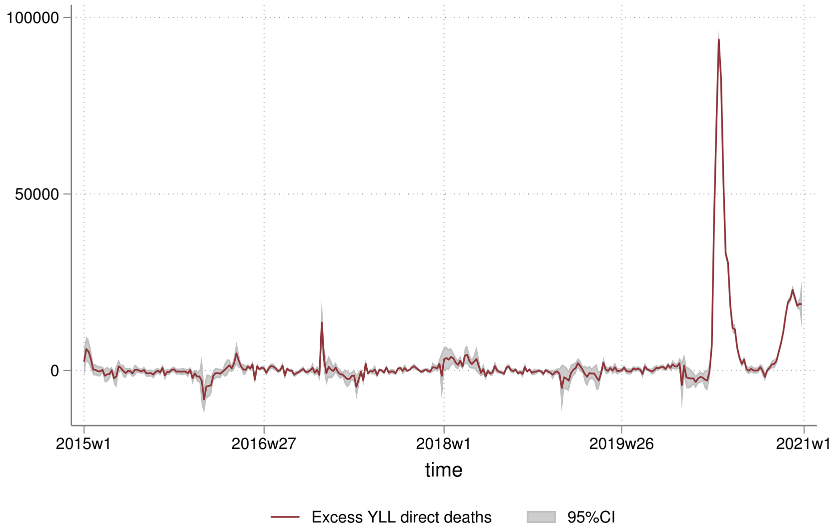

Figure 76: Excess Years of Life Lost trend, direct deaths, 2019-2020

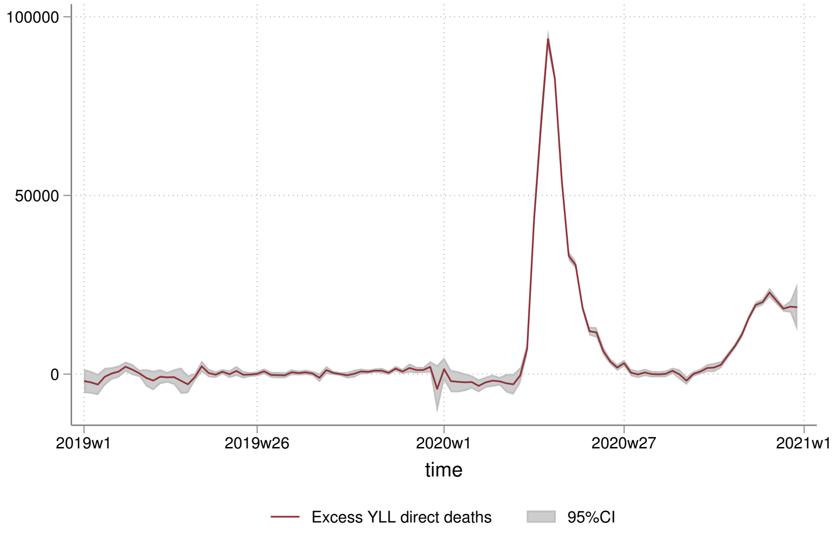

Figure 77: Observed vs Predicted Years of Life Lost trends, direct deaths, 2015-2020

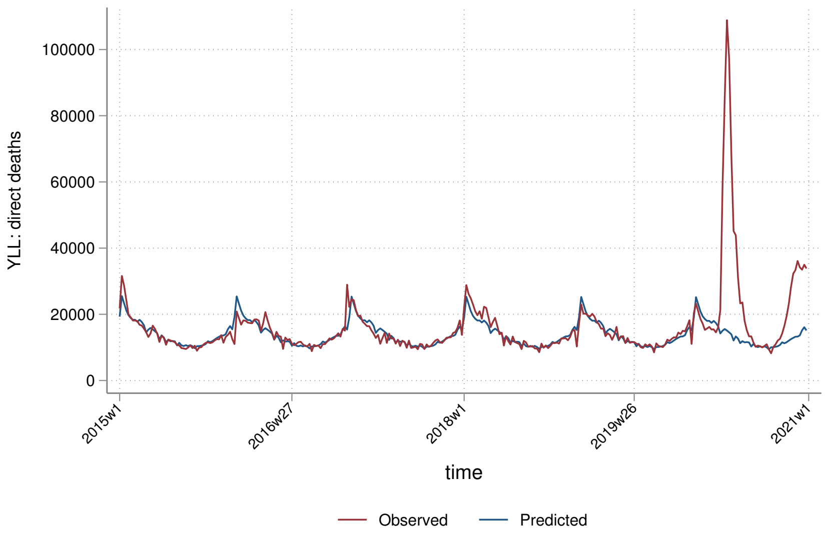

Figure 78: Observed vs Predicted Years of Life Lost trends, direct deaths, 2019-2020

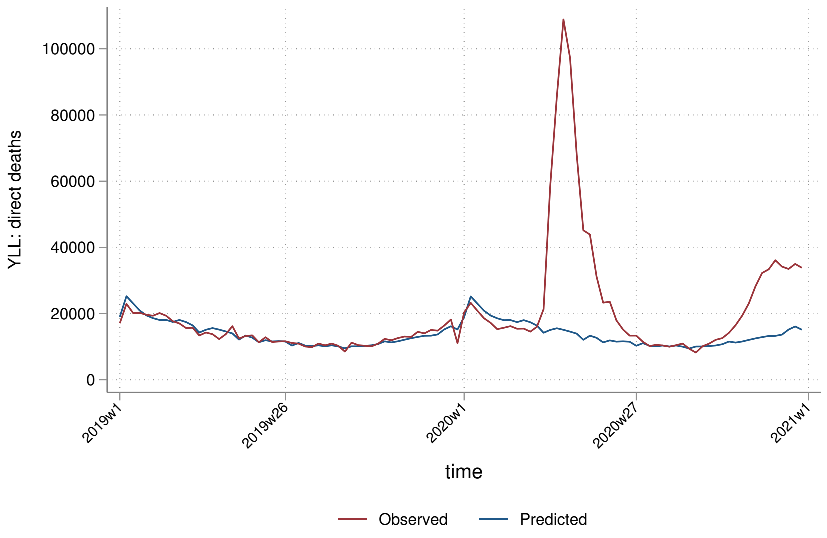

6.2.2 By sex

Figure 79: Years of Life Lost trend, direct deaths by sex, 2015-2020

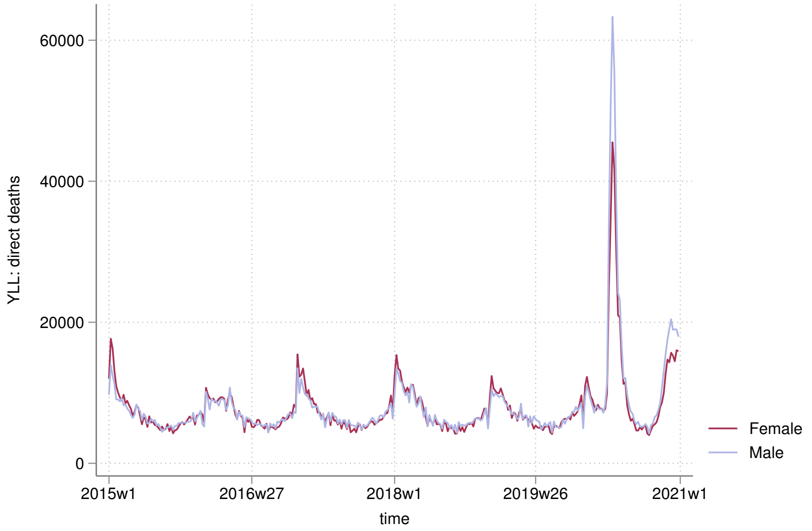

Figure 80: Years of Life Lost trend, direct deaths by sex, 2019-2020

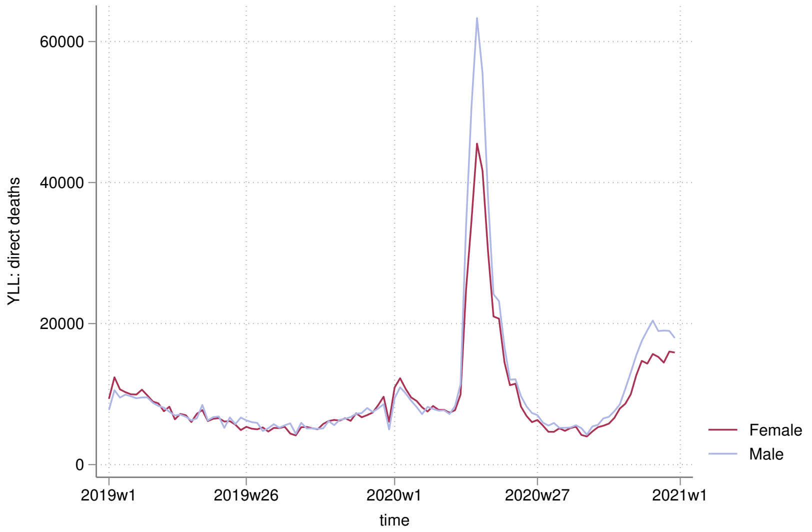

Figure 81: Excess Years of Life Lost trend, direct deaths by sex, 2015-2020

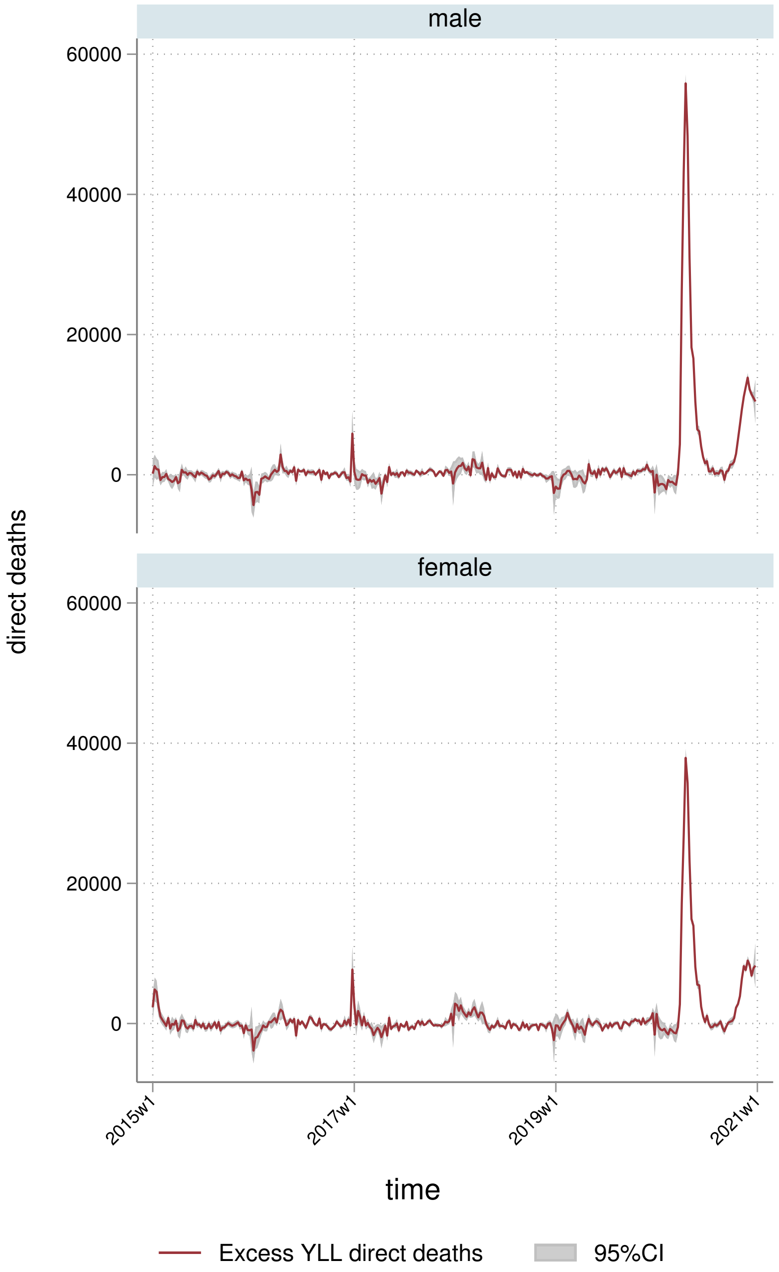

Figure 82: Excess Years of Life Lost trend, direct deaths by sex, 2019-2020

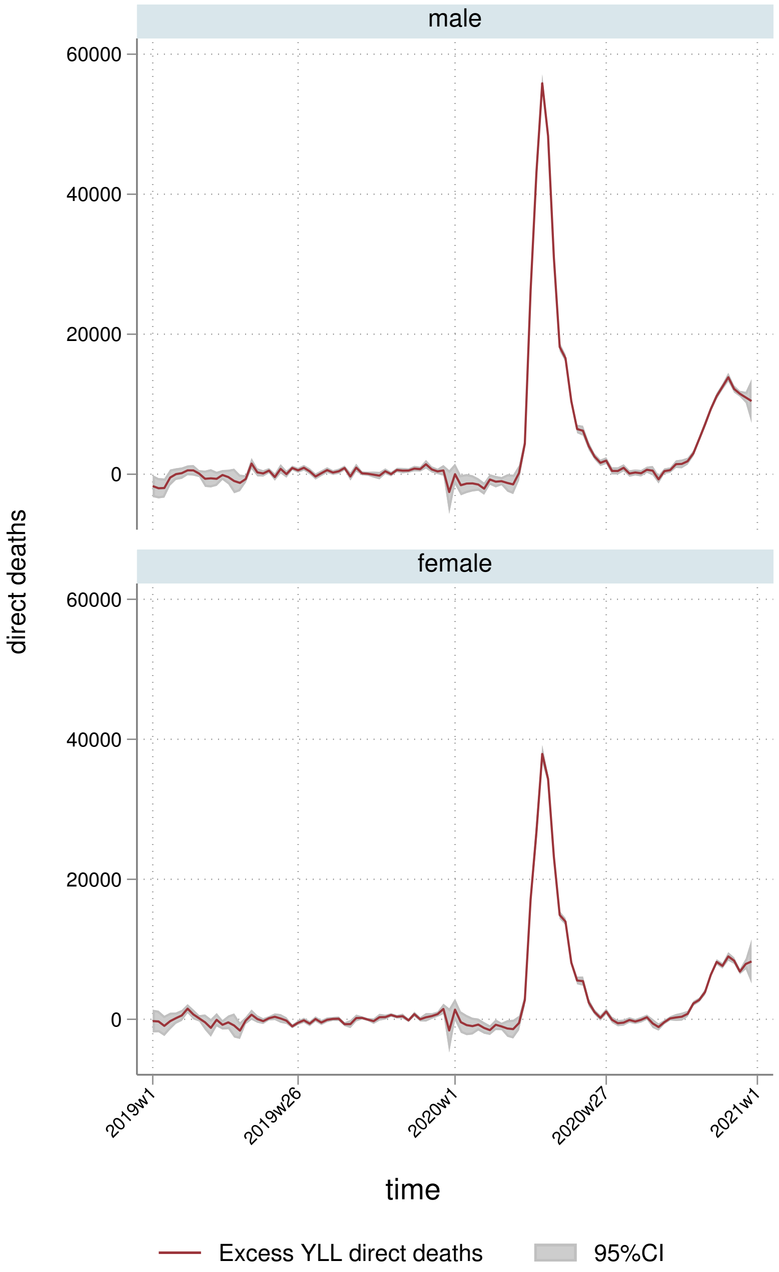

Figure 83: Observed vs Predicted Years of Life Lost trends, direct deaths by sex, 2015-2020

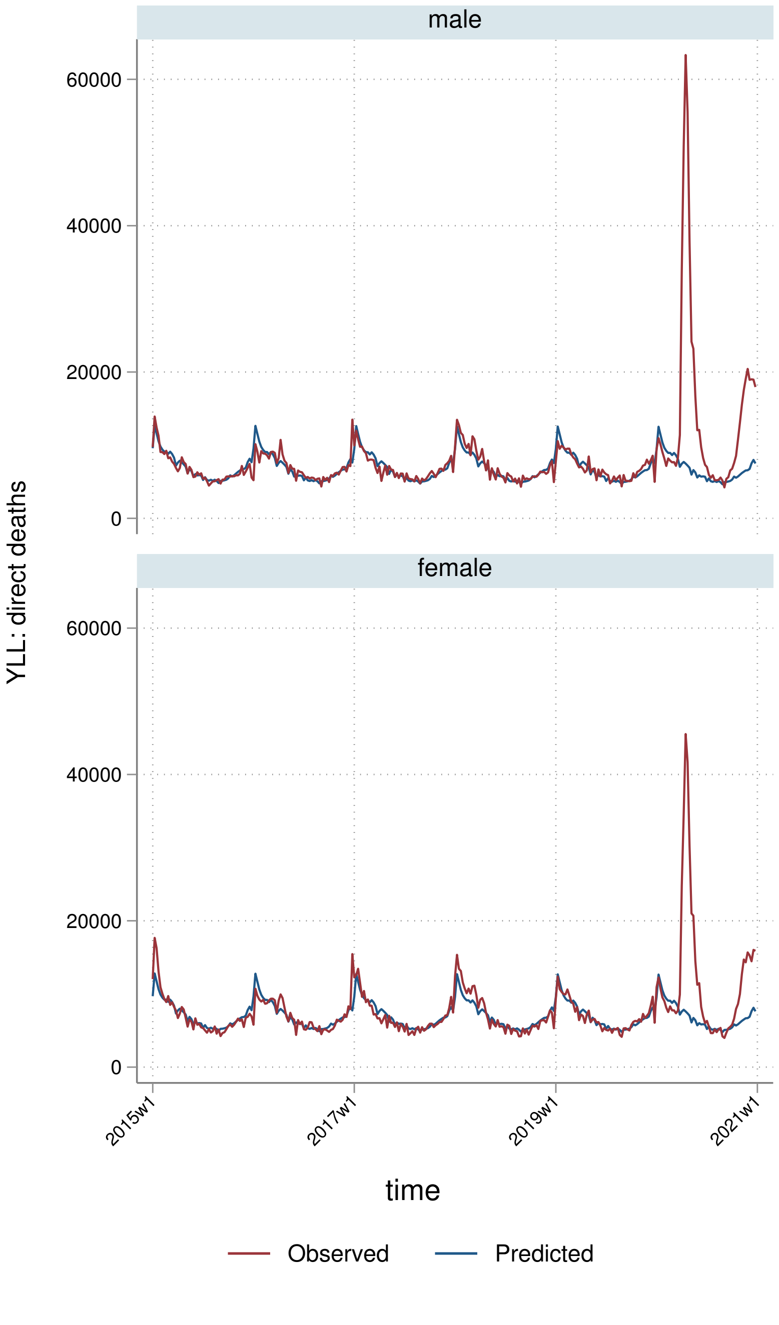

Figure 84: Observed vs Predicted Years of Life Lost trends, direct deaths by sex, 2019-2020

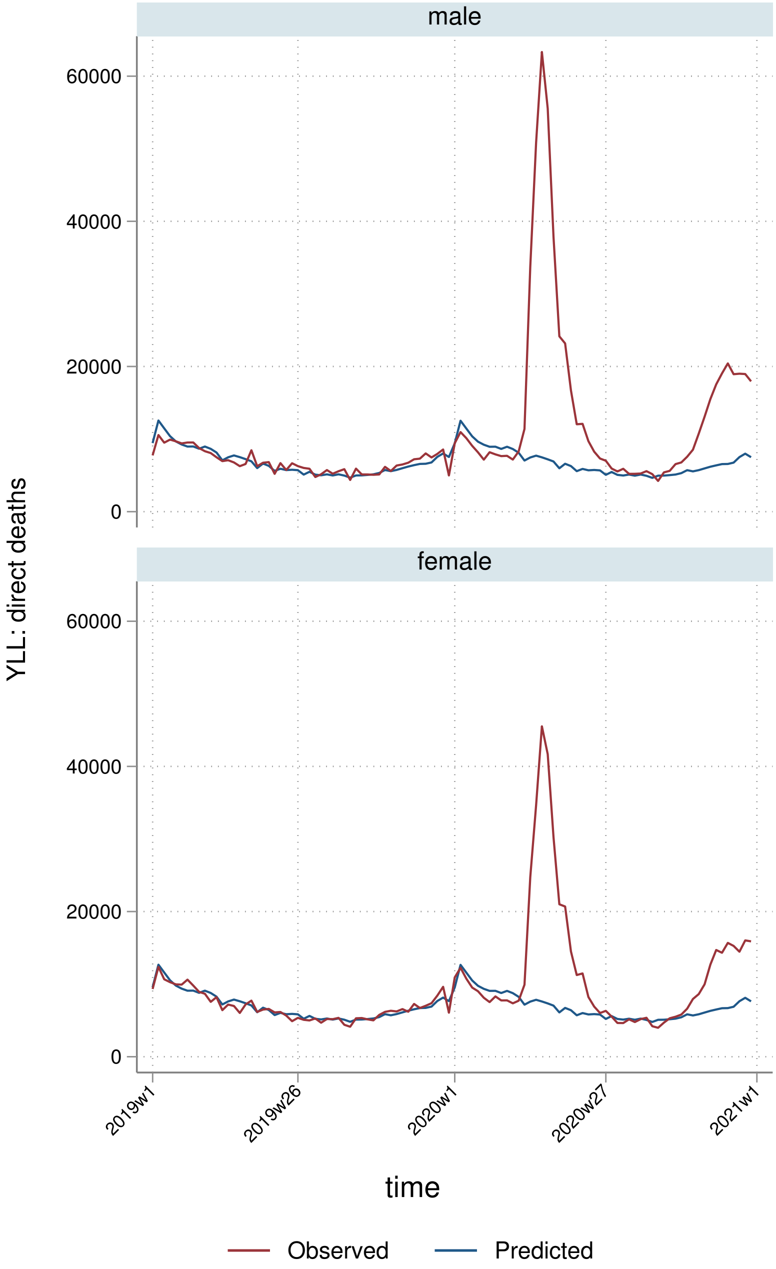

6.2.3 By deprivation quintile

Figure 85: Years of Life Lost trend, direct deaths by deprivation quintile, 2015-2020

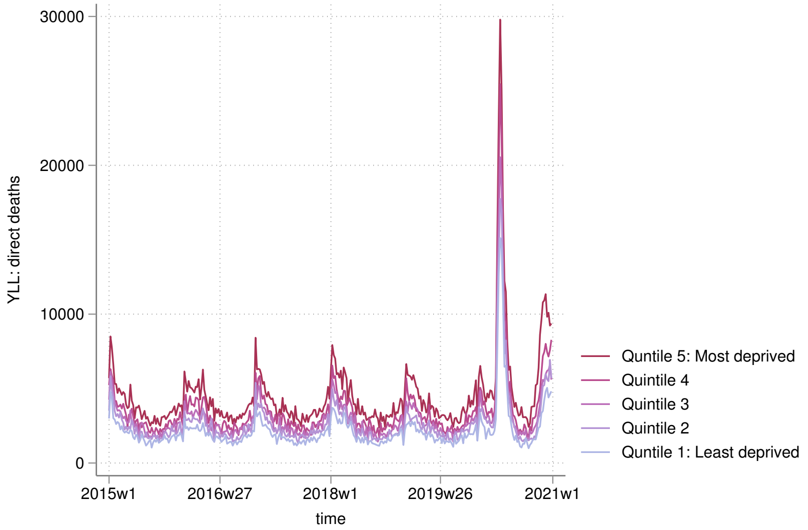

Figure 86: Years of Life Lost trend, direct deaths by deprivation quintile, 2019-2020

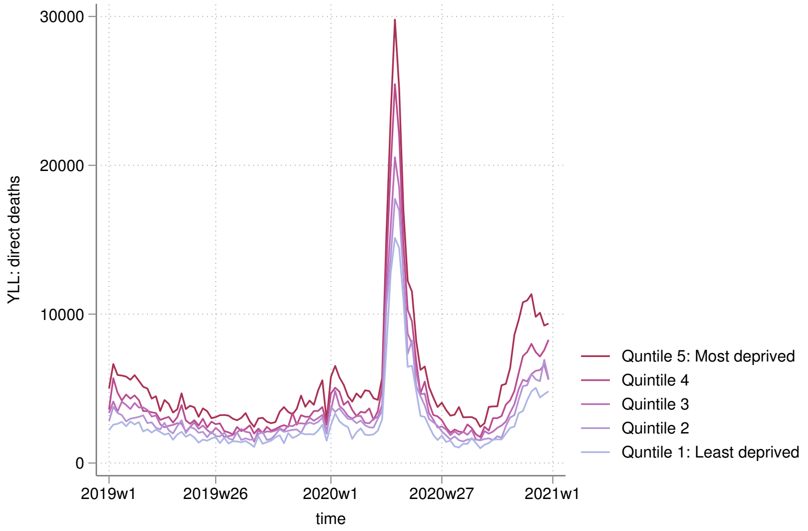

Figure 87: Excess Years of Life Lost trend, direct deaths by deprivation quintile, 2015-2020

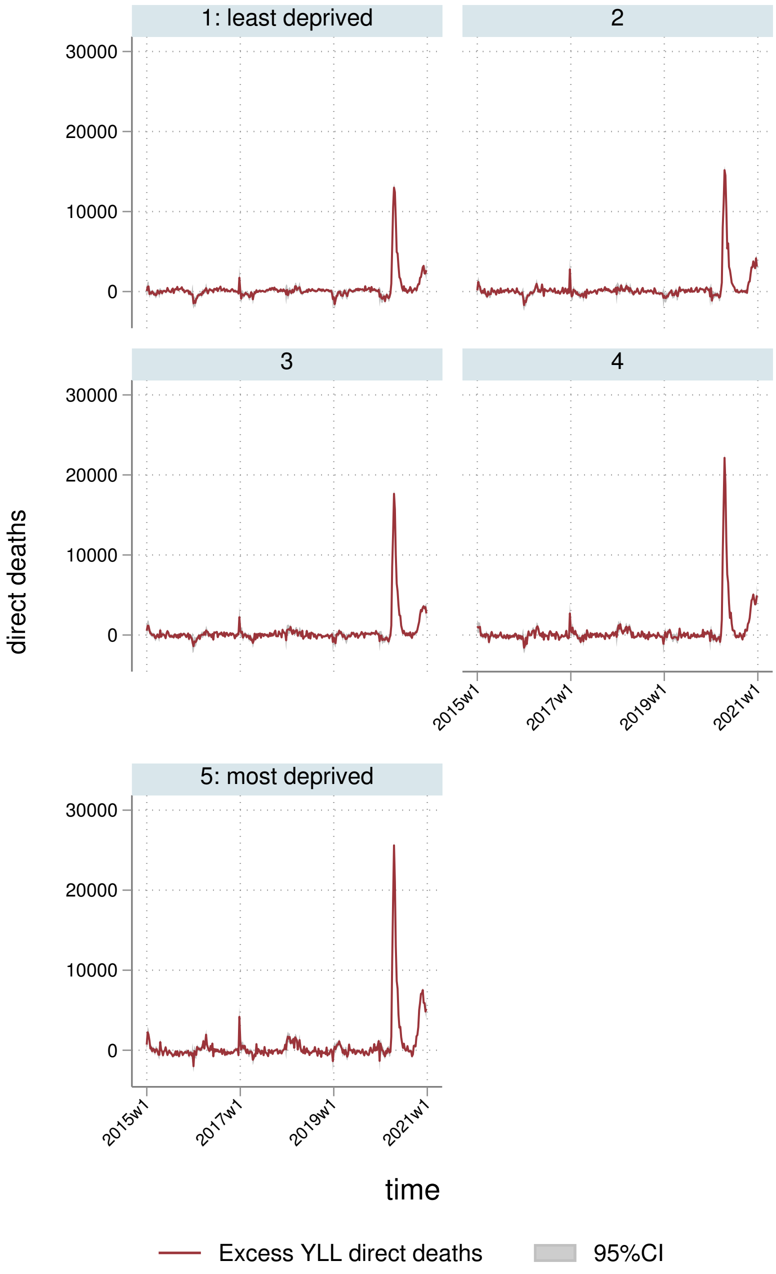

Figure 88: Excess Years of Life Lost trend, direct deaths by deprivation quintile, 2019-2020

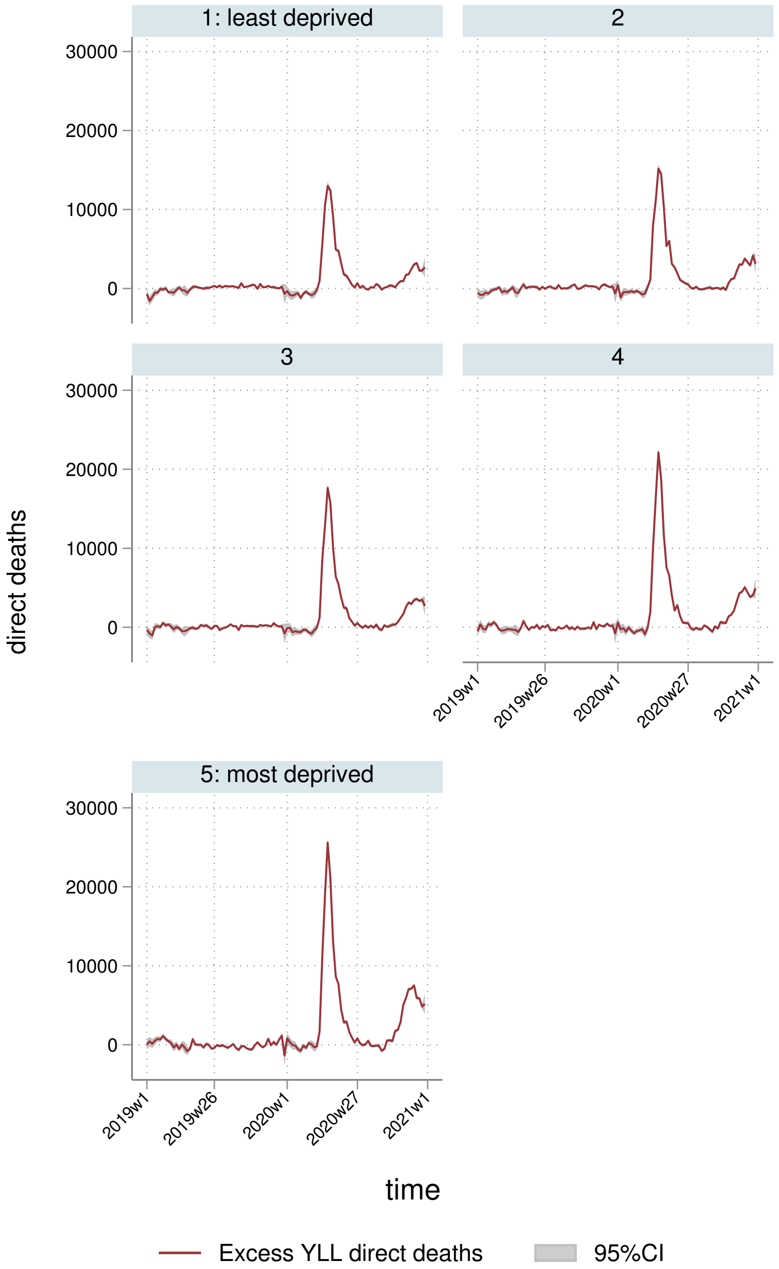

Figure 89: Observed vs Predicted Years of Life Lost trends, direct deaths by deprivation quintile, 2015-2020

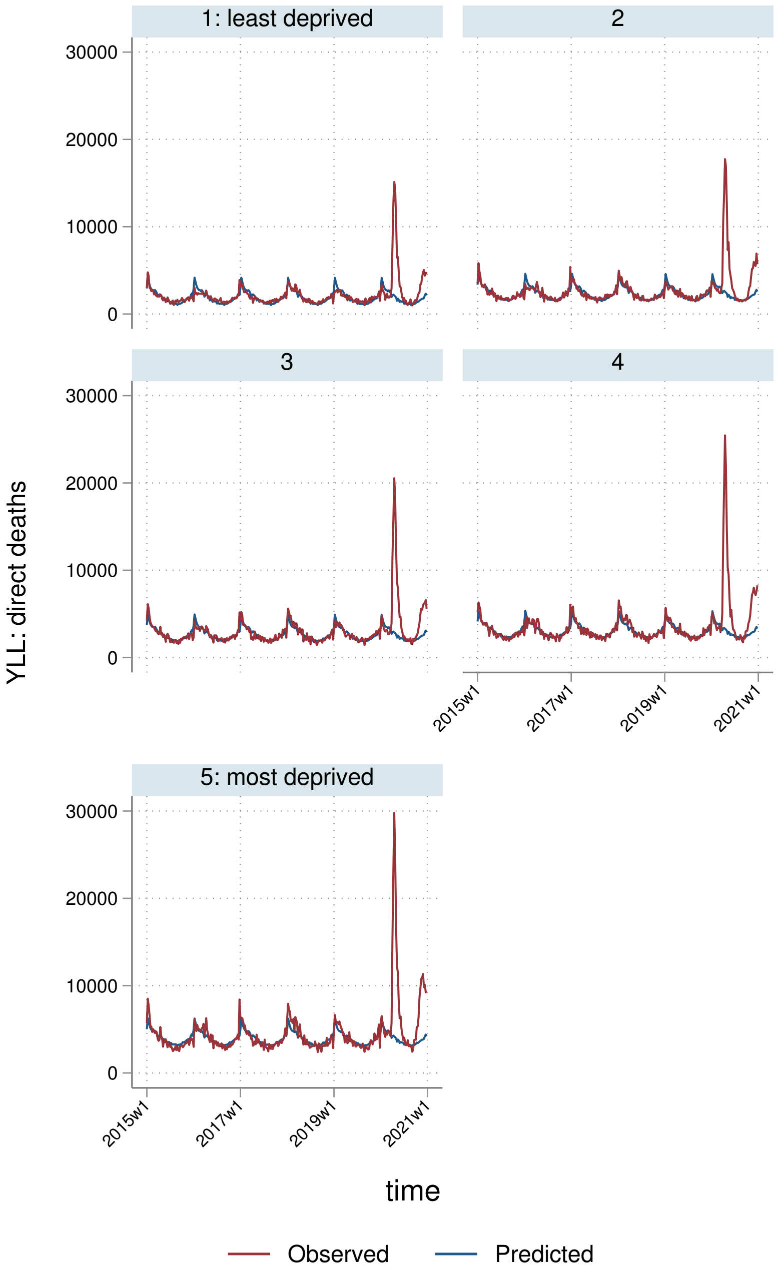

Figure 90: Observed vs Predicted Years of Life Lost trends, direct deaths by deprivation quintile, 2019-2020

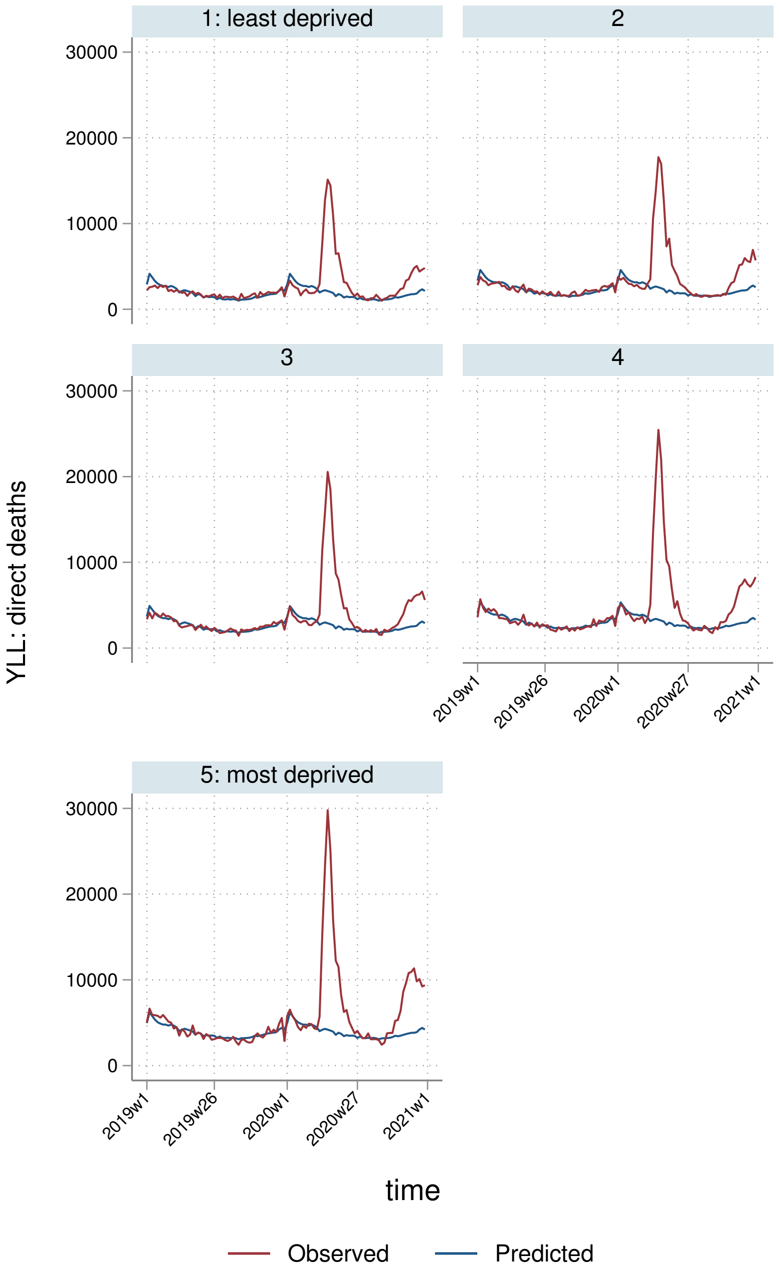

6.2.4 By Strategic Health Authority

Figure 91: Years of Life Lost trend, direct deaths by region, 2015-2020

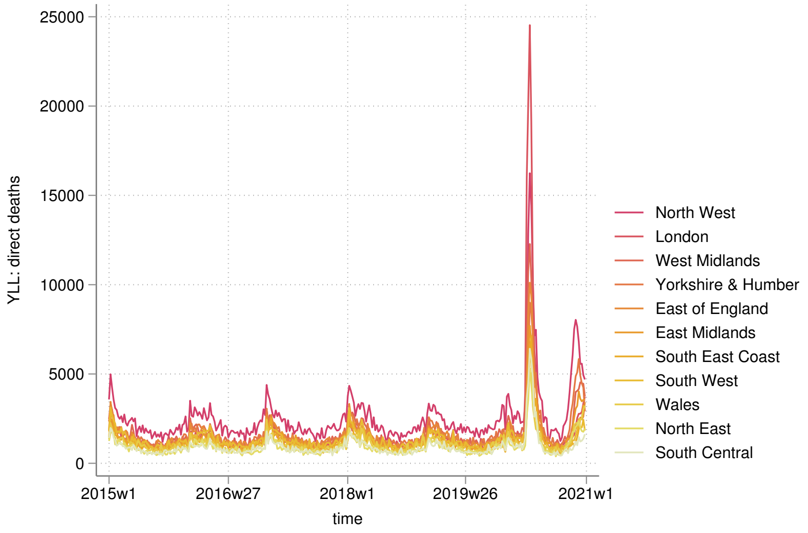

Figure 92: Years of Life Lost trend, direct deaths by region, 2019-2020

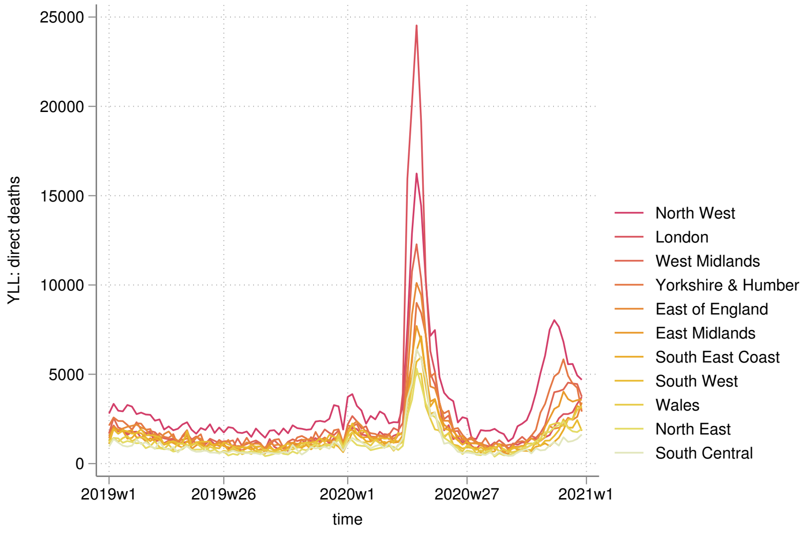

Figure 93: Excess Years of Life Lost trend, direct deaths by deprivation quintile, 2015-2020

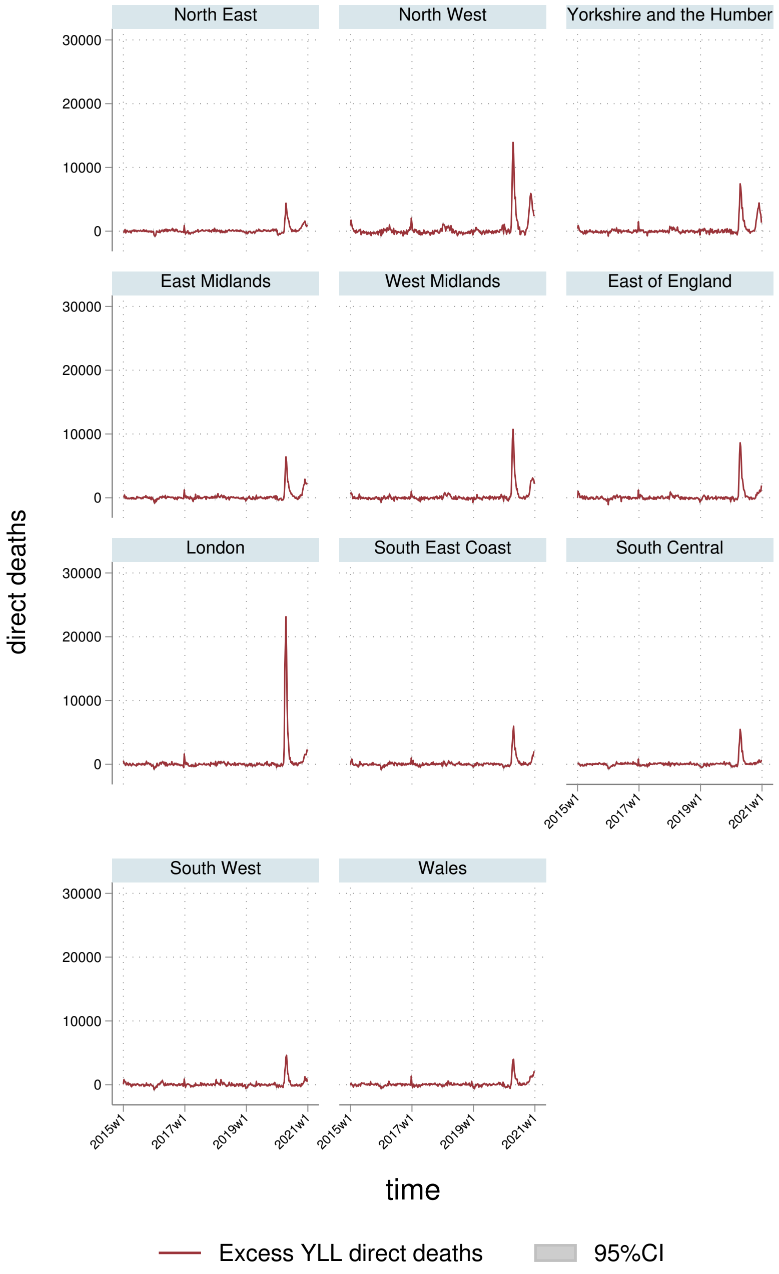

Figure 94: Excess Years of Life Lost trend, direct deaths by deprivation quintile, 2019-2020

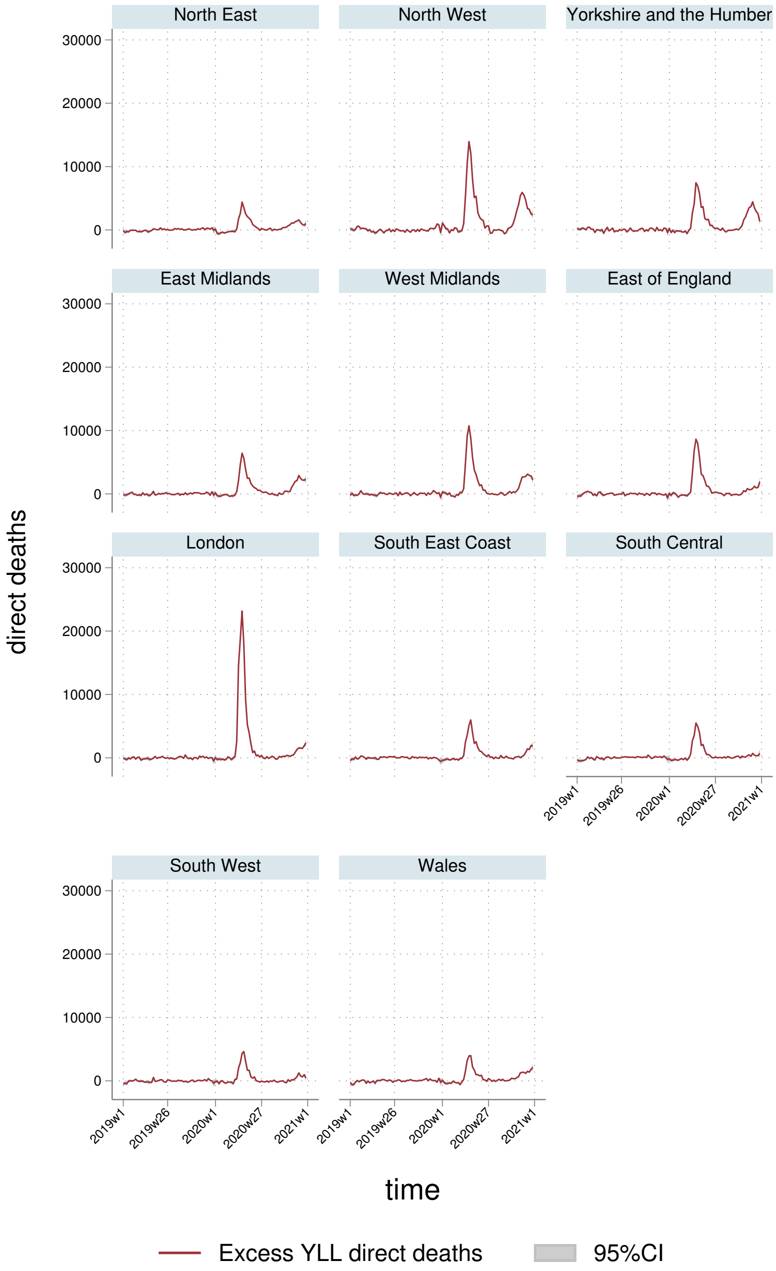

Figure 95: Observed vs Predicted Years of Life Lost trends, direct deaths by deprivation quintile, 2015-2020

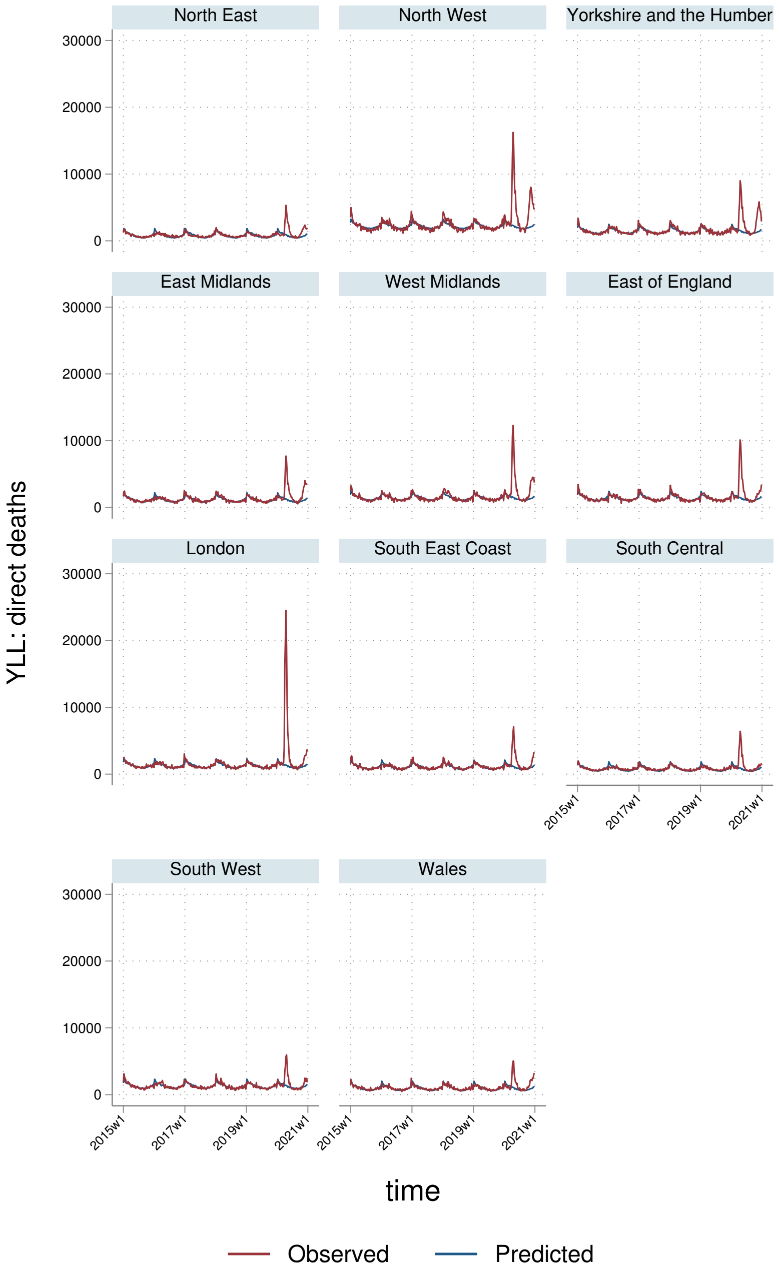

Figure 96: Observed vs Predicted Years of Life Lost trends, direct deaths by deprivation quintile, 2019-2020

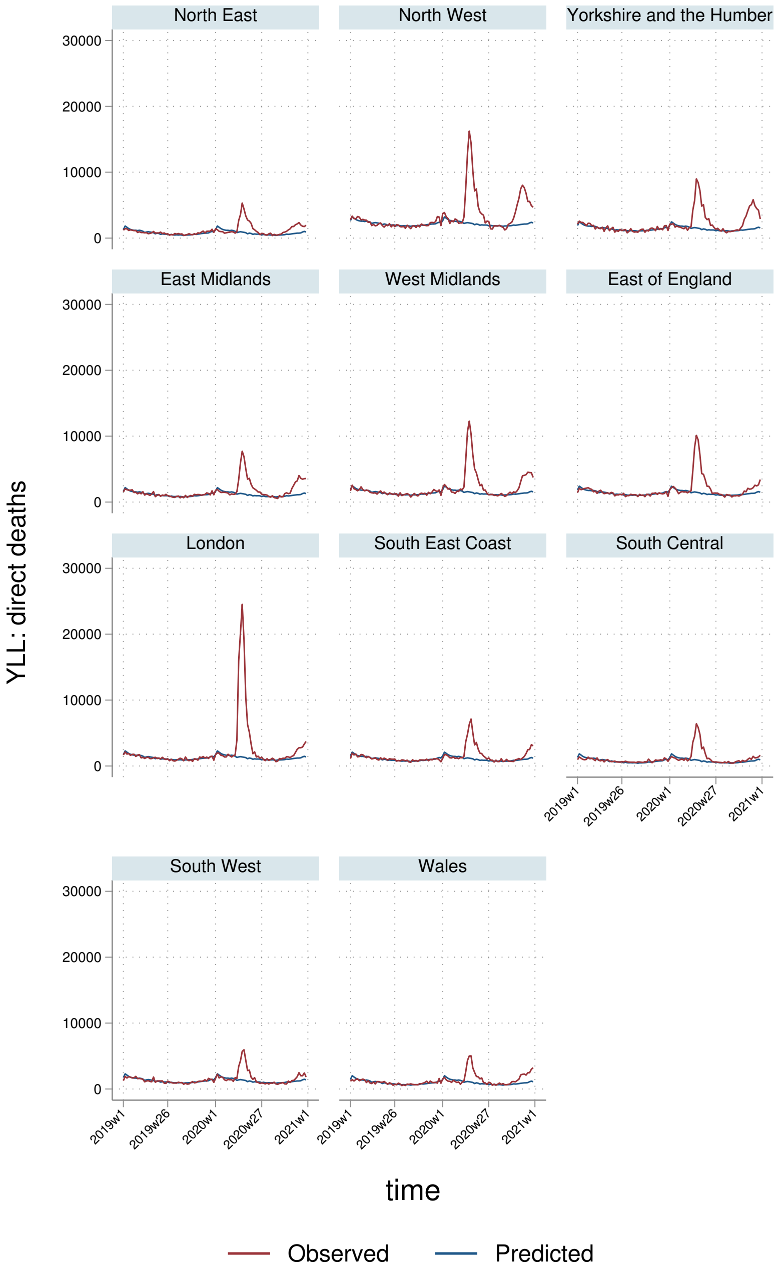

6.2.5 By deprivation quintile & Strategic Health Authority

Figure 97: Years of Life Lost trend, direct deaths by regionXdeprivation, 2015-2020

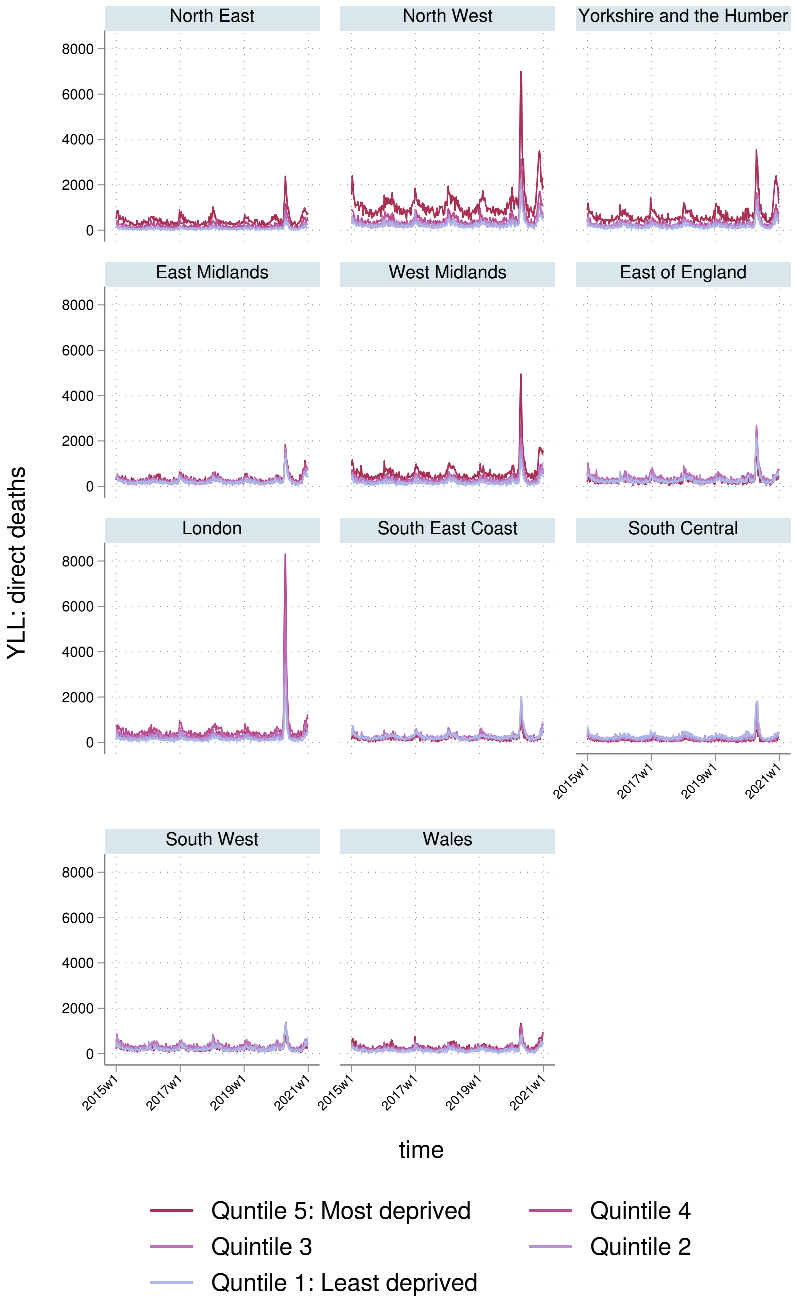

Figure 98: Years of Life Lost trend, direct deaths by region, 2019-2020

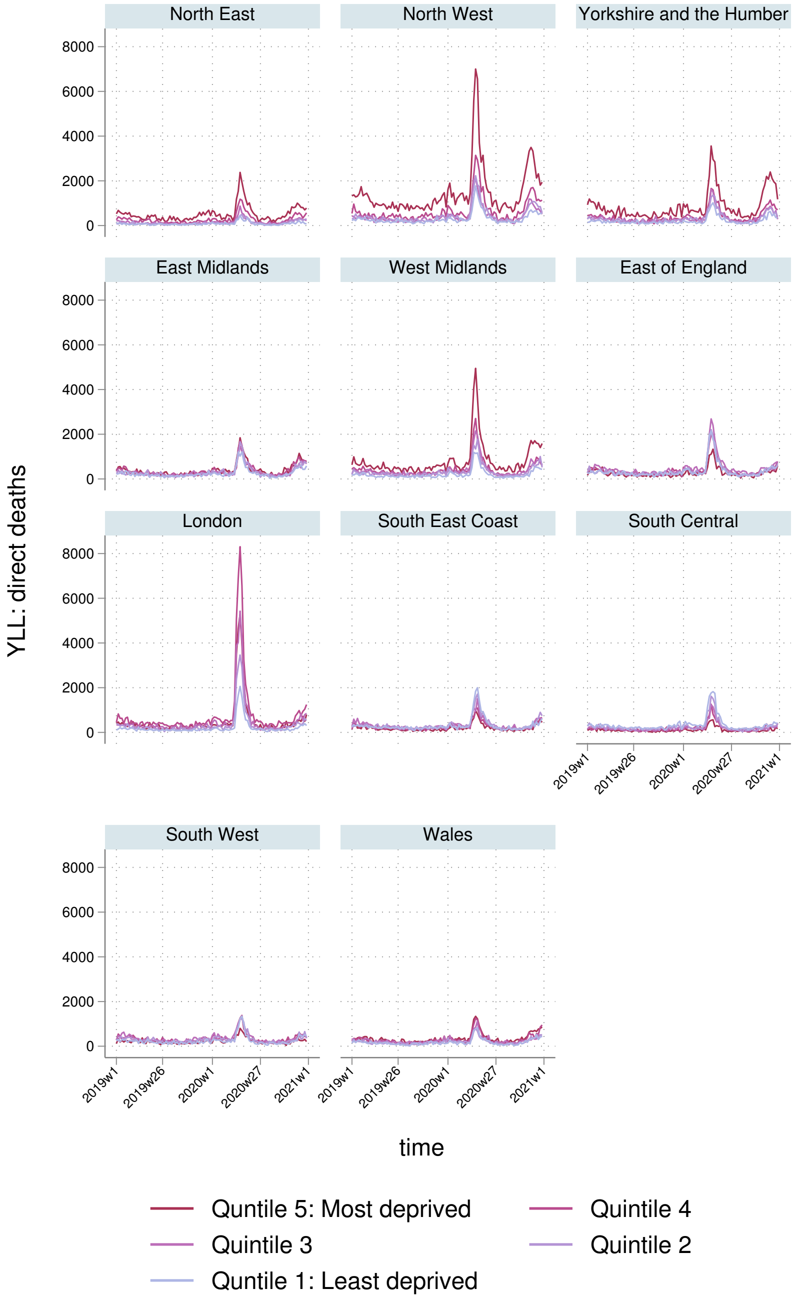

Figure 99: Excess Years of Life Lost trend, direct deaths by regionXdeprivation, 2015-2020

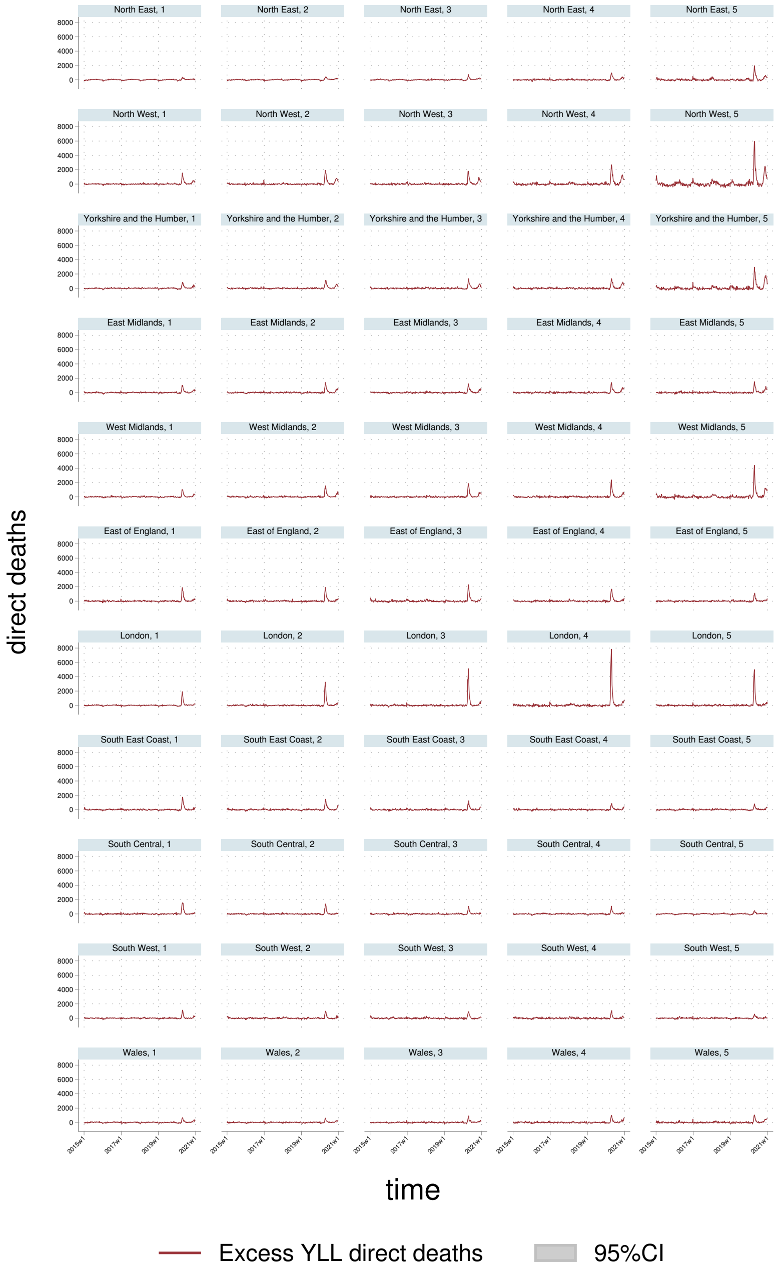

Figure 100: Excess Years of Life Lost trend, direct deaths by regionXdeprivation, 2019-2020

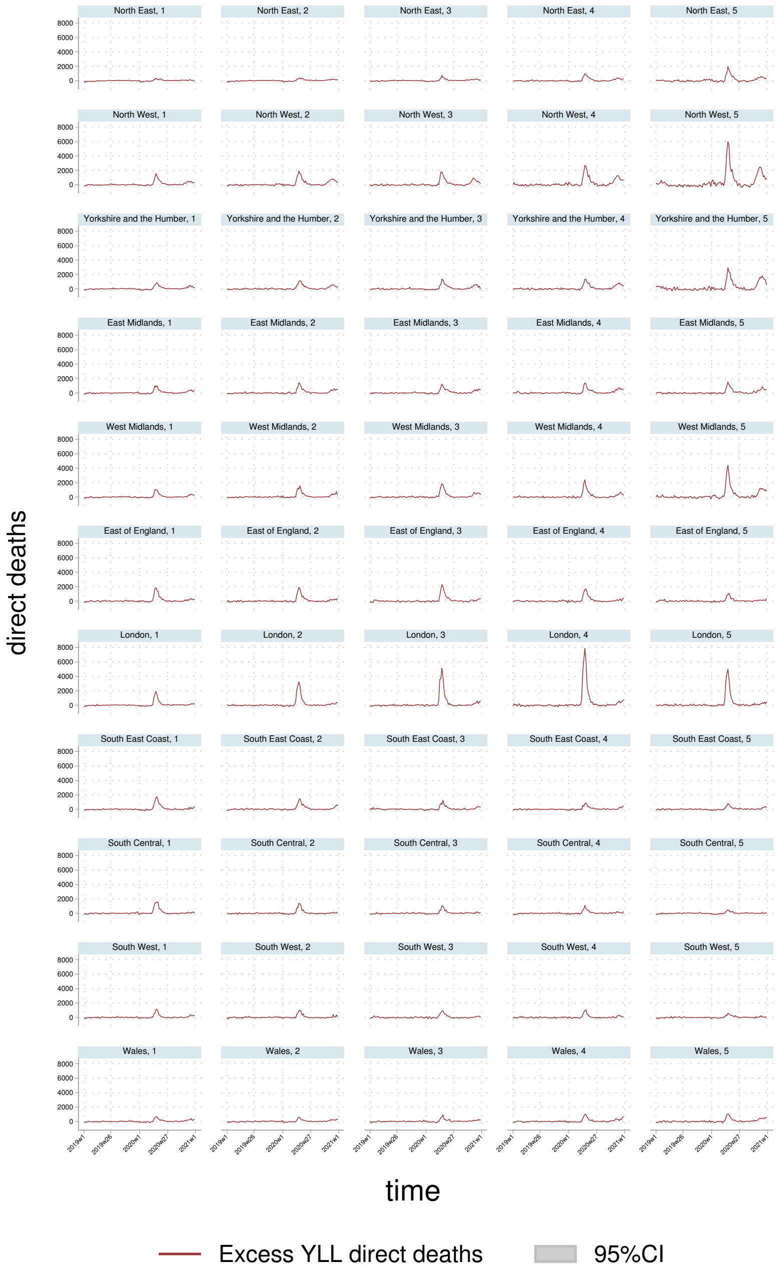

Figure 101: Observed vs Predicted Years of Life Lost trends, direct deaths by regionXdeprivation, 2015-2020

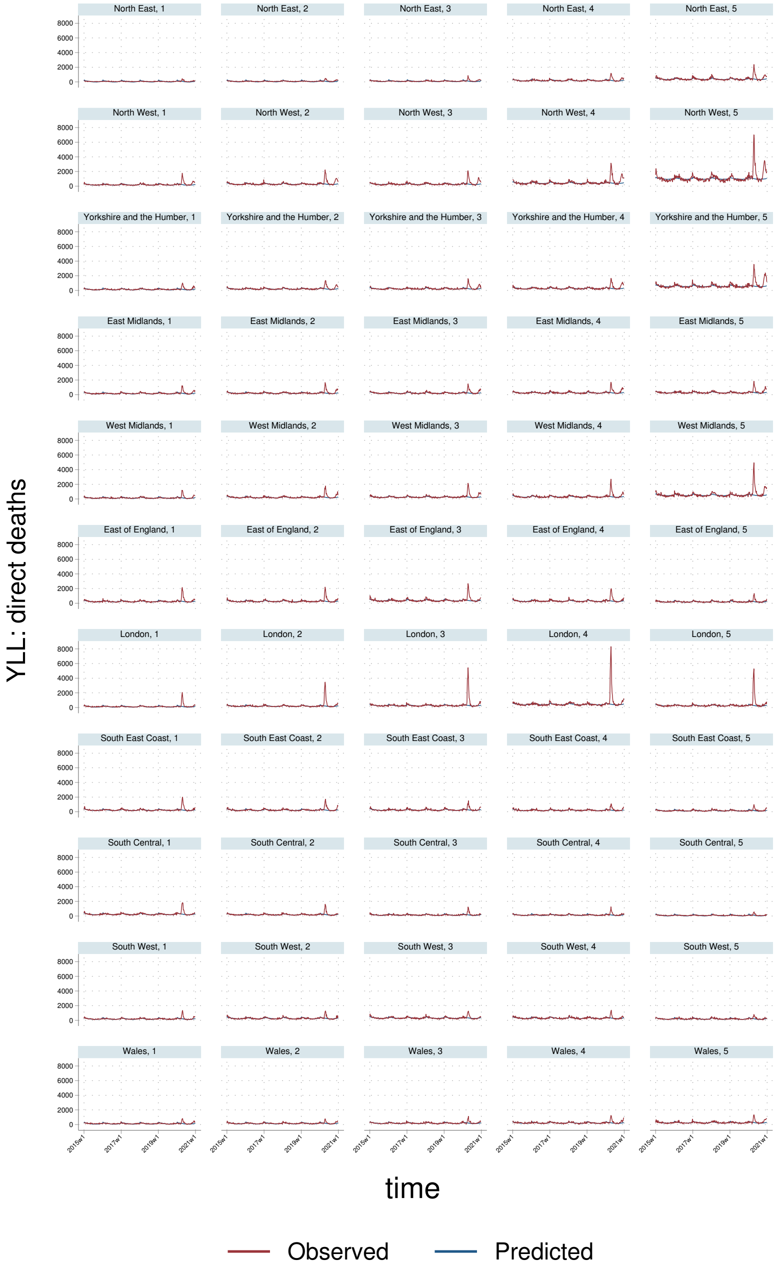

Figure 102: Observed vs Predicted Years of Life Lost trends, direct deaths by regionXdeprivation, 2019-2020

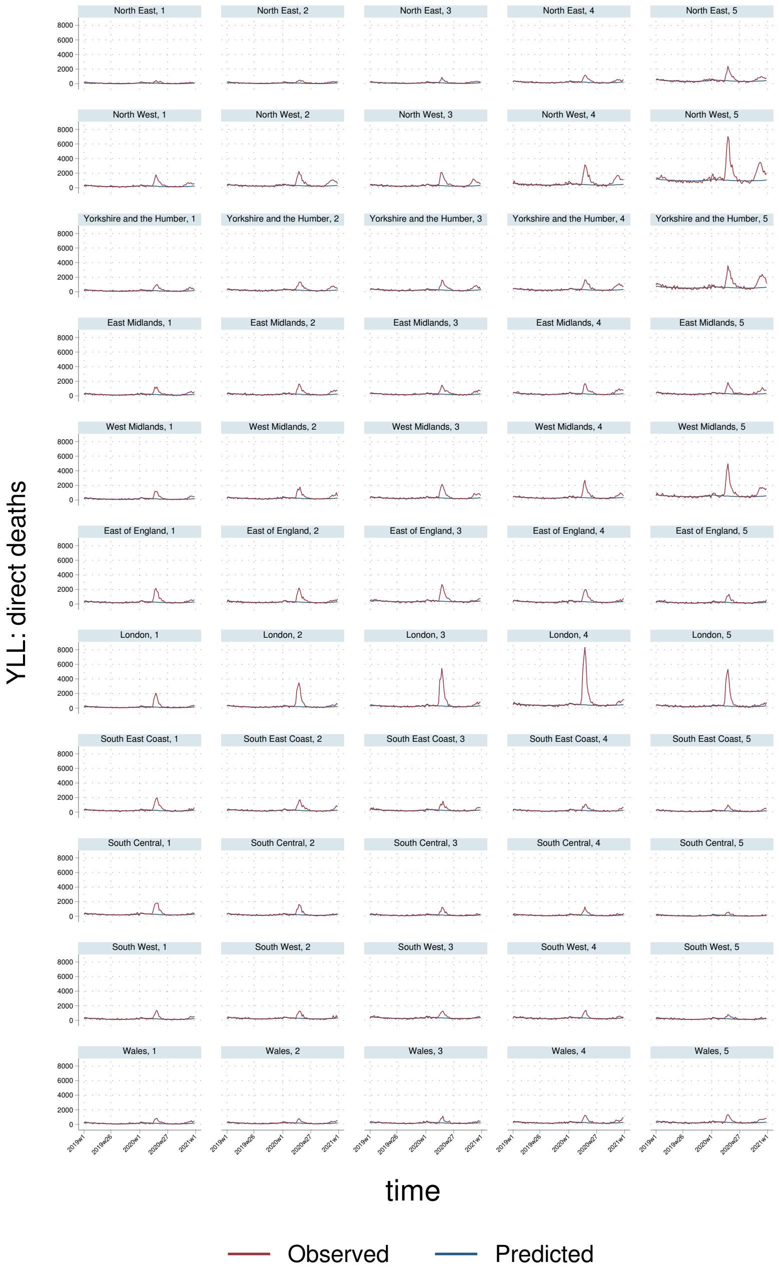

6.3 YLLs per $100,000$ population

6.3.1 England-Wales aggregate

Figure 103: Years of Life Lost trend per $100,000$ population, direct deaths, 2015-2020

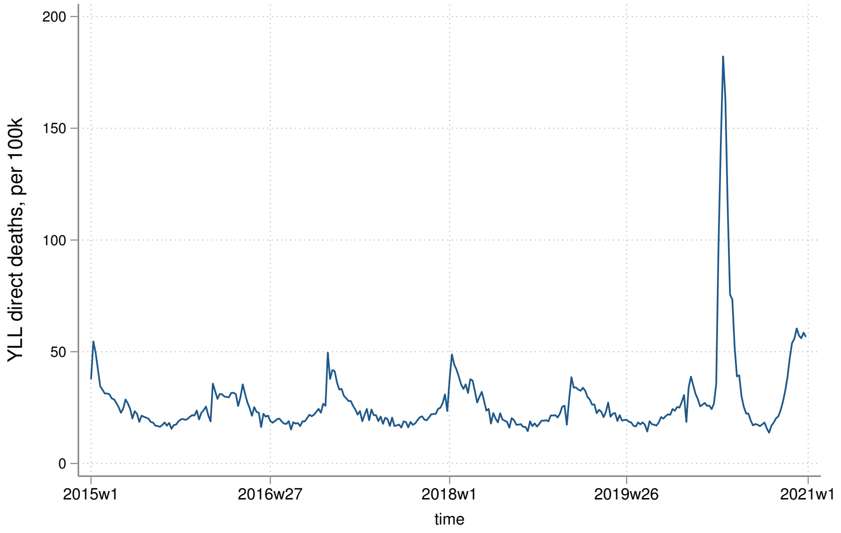

Figure 104: Years of Life Lost trend per $100,000$ population, direct deaths, 2019-2020

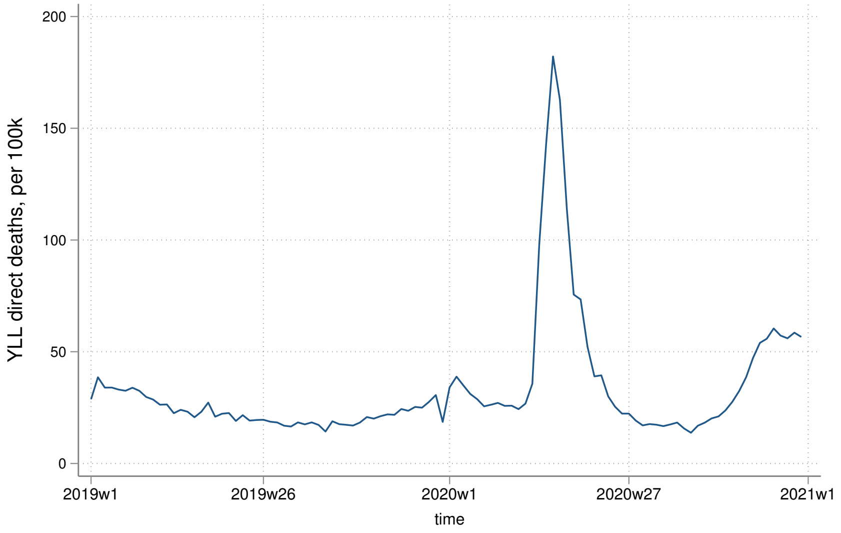

Figure 105: Excess Years of Life Lost trend per 100,000 population, direct deaths, 2015-2020

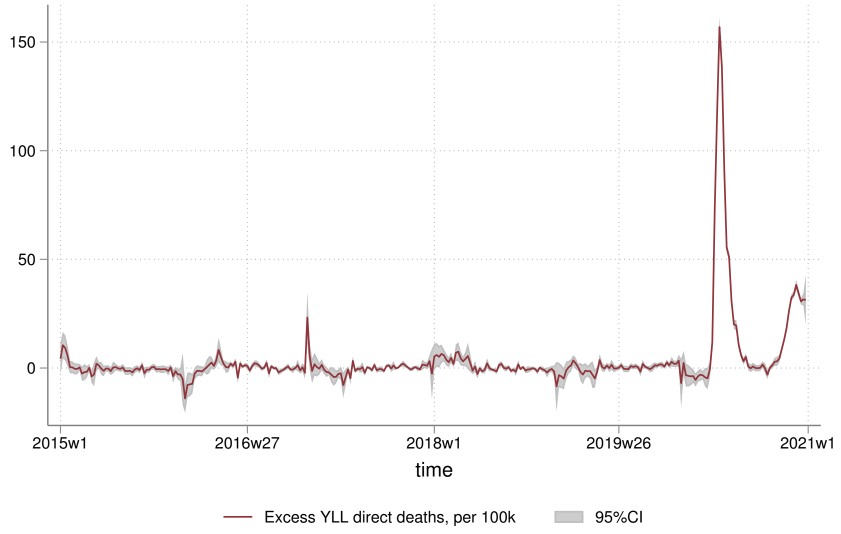

Figure 106: Excess Years of Life Lost trend per 100,000 population, direct deaths, 2019-2020

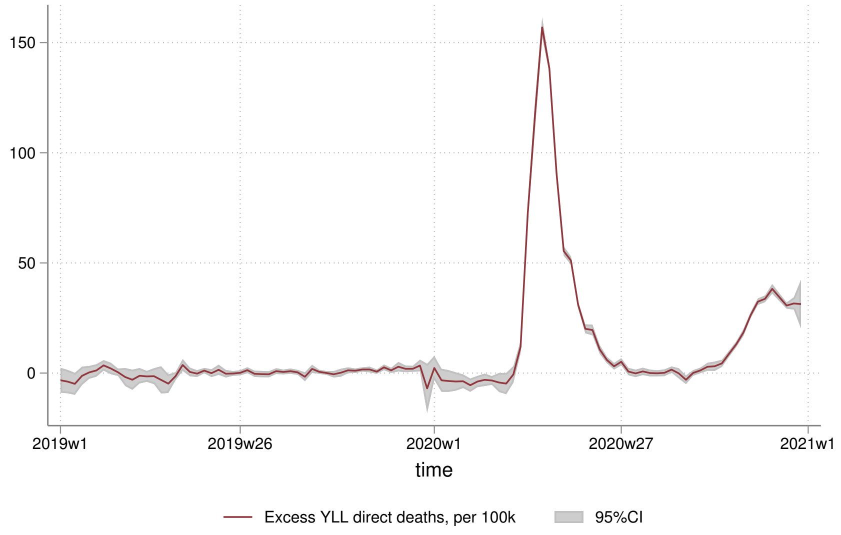

Figure 107: Observed vs Predicted Years of Life Lost trends per 100,000 population, direct deaths, 2015-2020

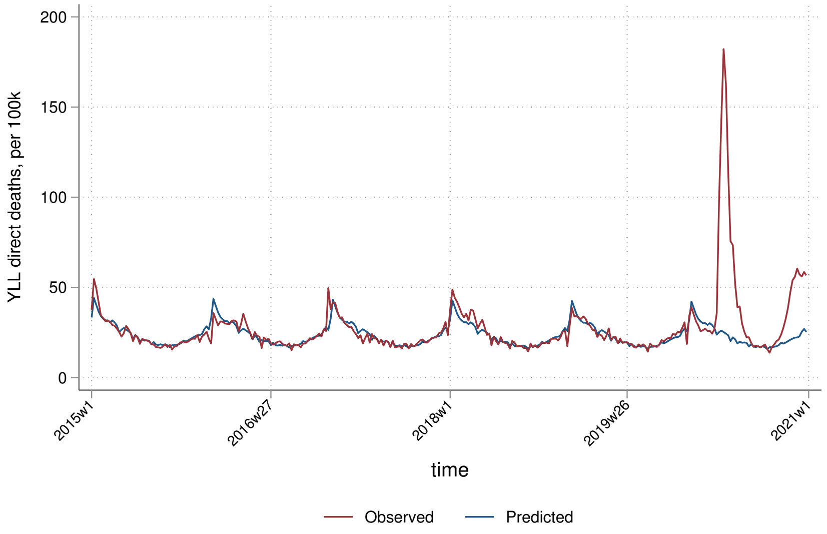

Figure 108: Observed vs Predicted Years of Life Lost trends per 100,000 population, direct deaths, 2019-2020

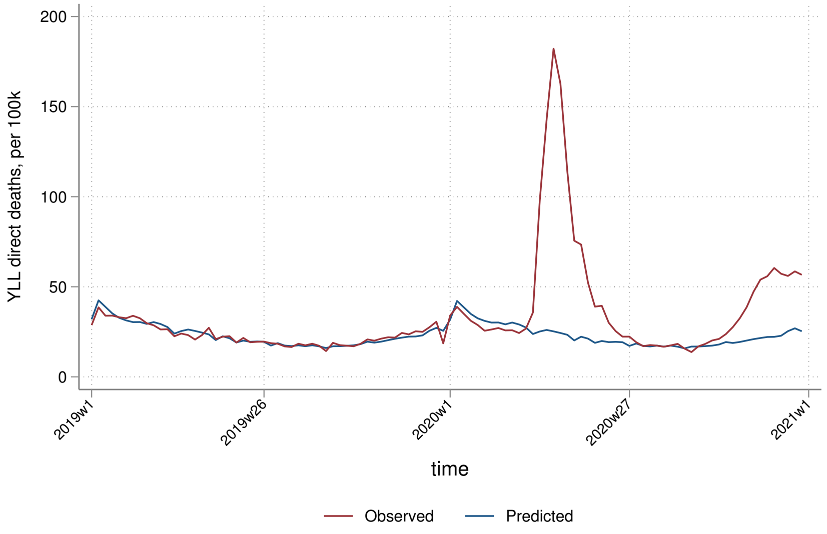

6.3.2 By sex

Figure 109: Years of Life Lost trend per 100,000 population, direct deaths by sex, 2015-2020

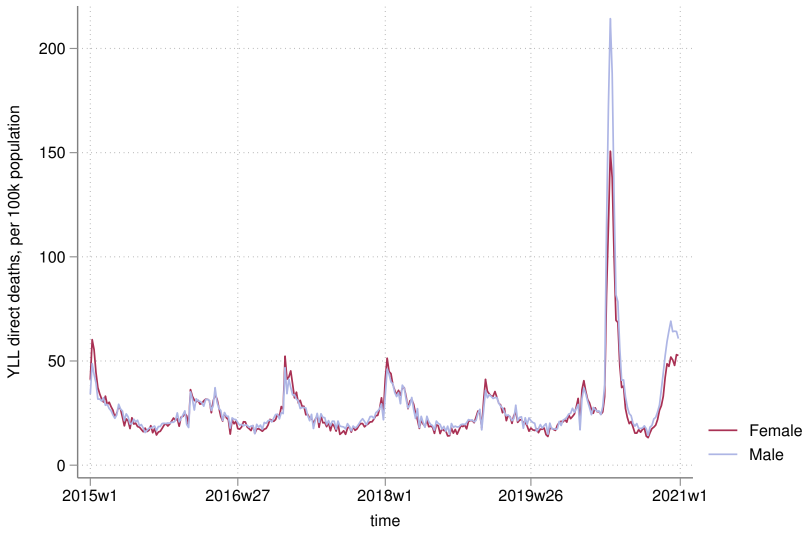

Figure 110: Years of Life Lost trend per 100,000 population, direct deaths by sex, 2019-2020

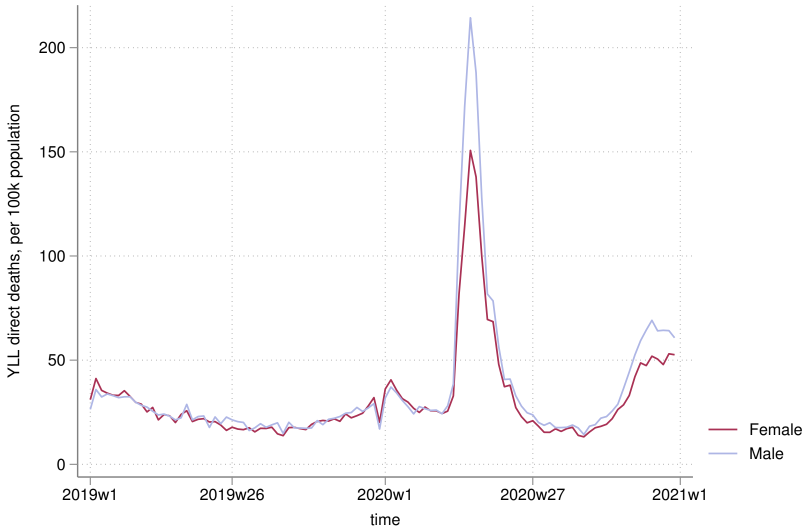

Figure 111: Excess Years of Life Lost trend per 100,000 population, direct deaths by sex, 2015-2020

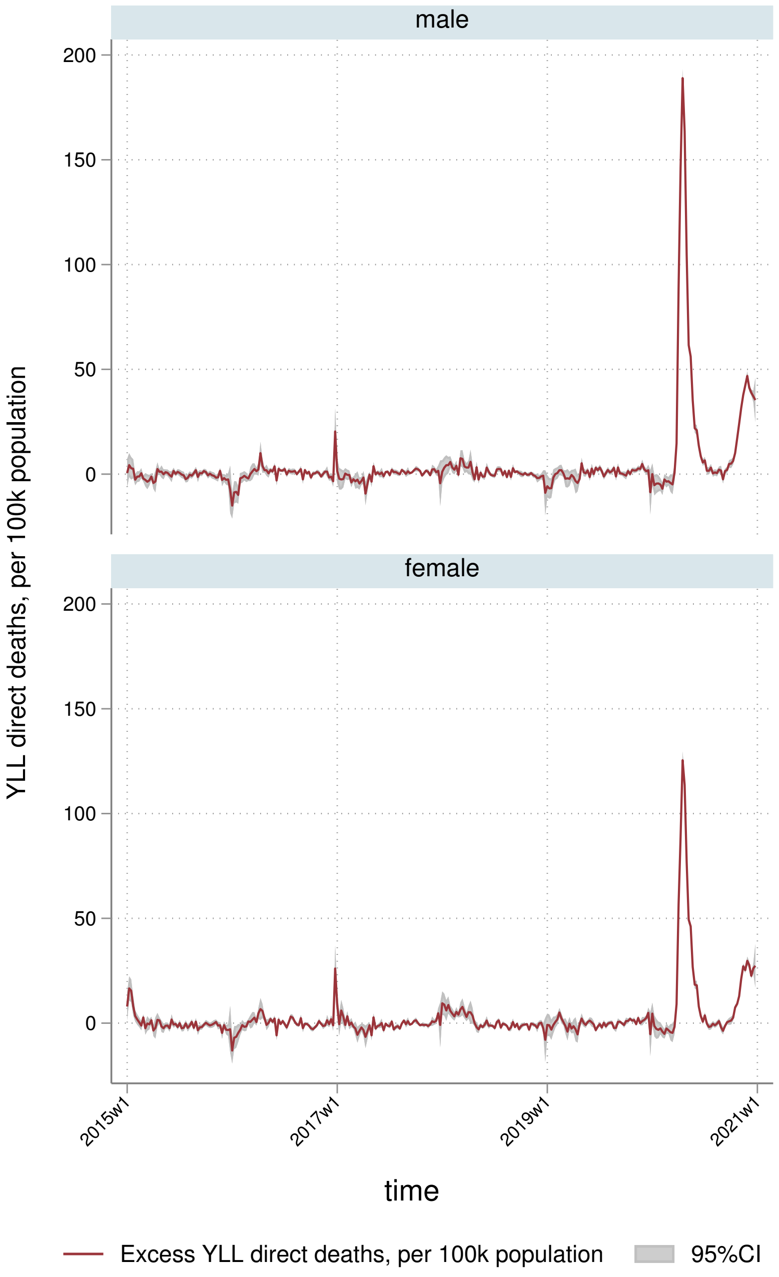

Figure 112: Excess Years of Life Lost trend per 100,000 population, direct deaths by sex, 2019-2020

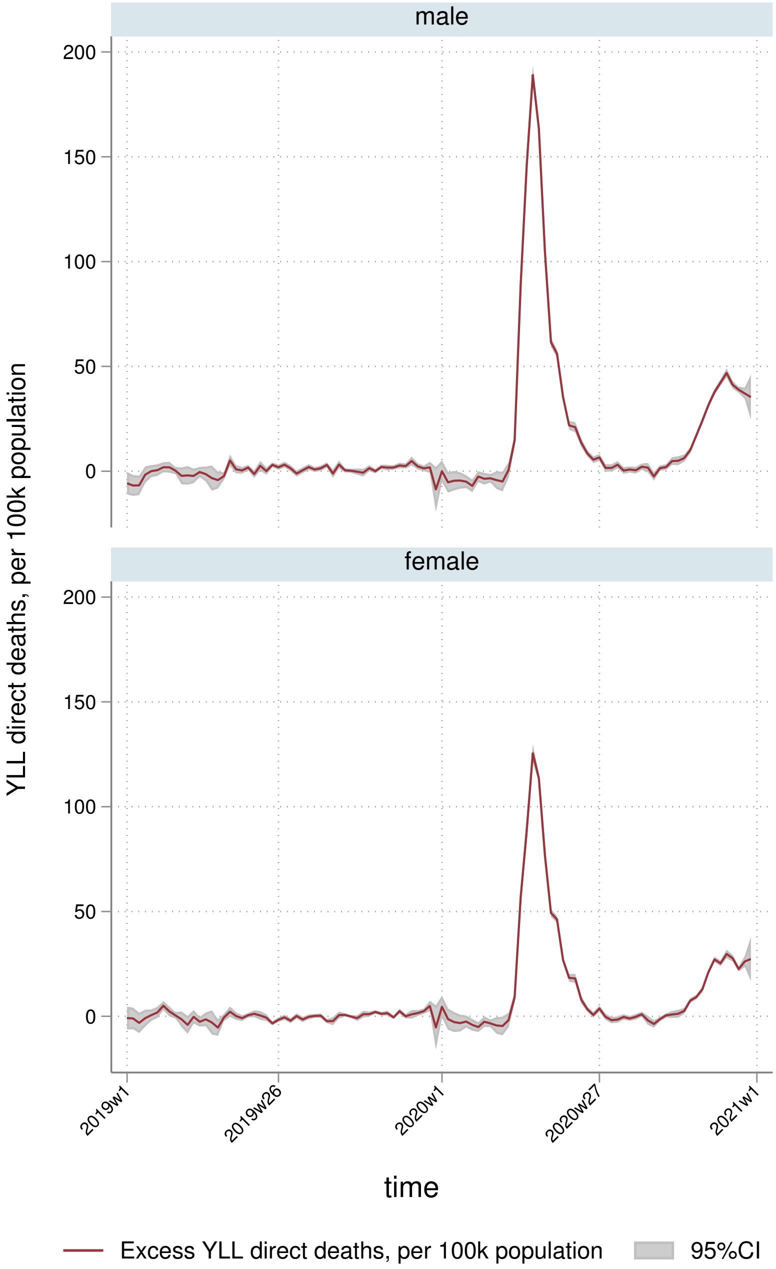

Figure 113: Observed vs Predicted Years of Life Lost trends per 100,000 population, direct deaths by sex, 2015-2020

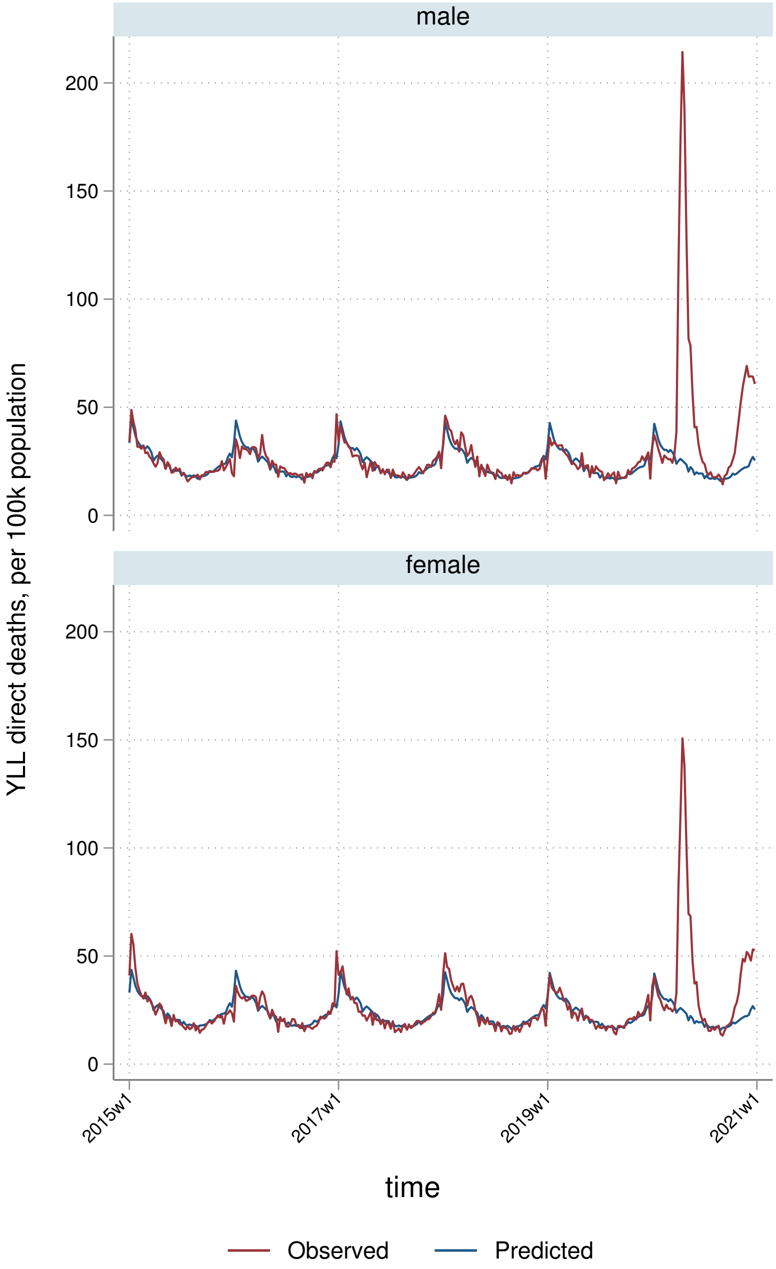

Figure 114: Observed vs Predicted Years of Life Lost trends per 100, 000 population, direct deaths by sex, 2019-2020

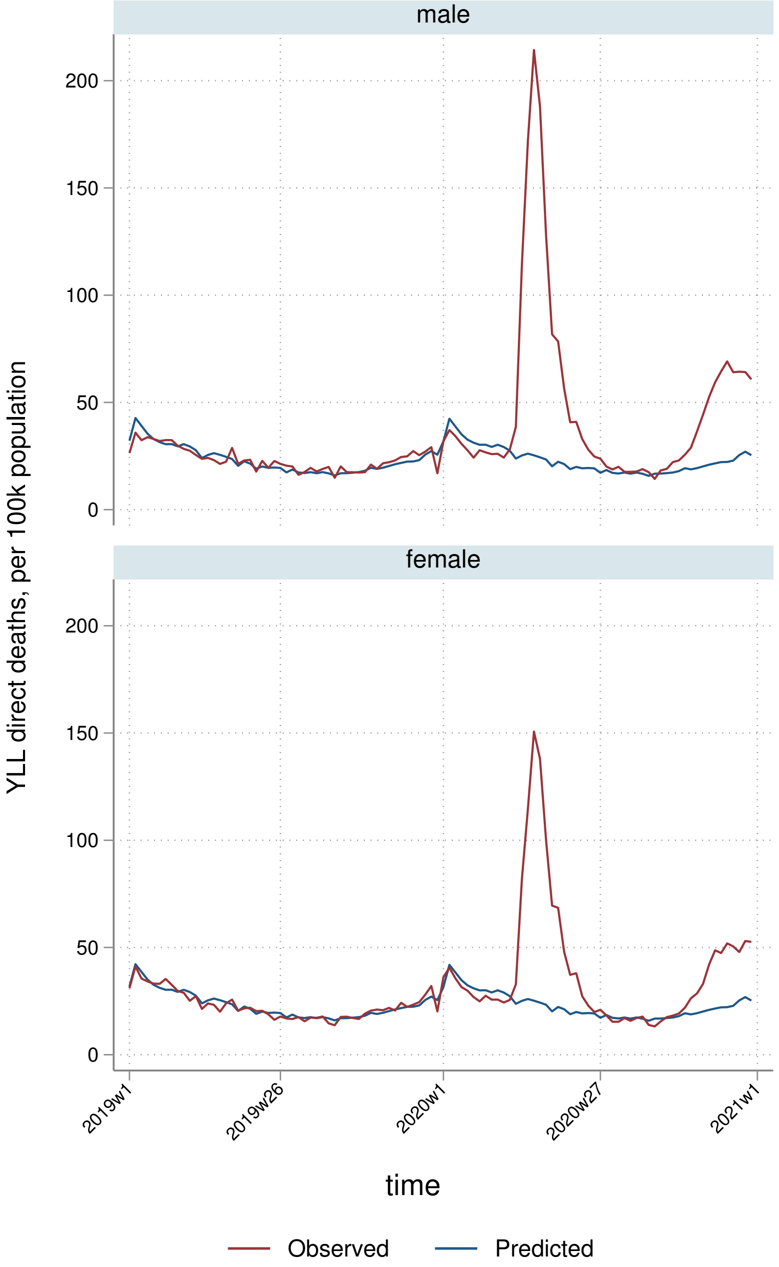

6.3.3 By deprivation quintile

Figure 115: Years of Life Lost trend per 100,000 population, direct deaths by deprivation quintile, 2015-2020

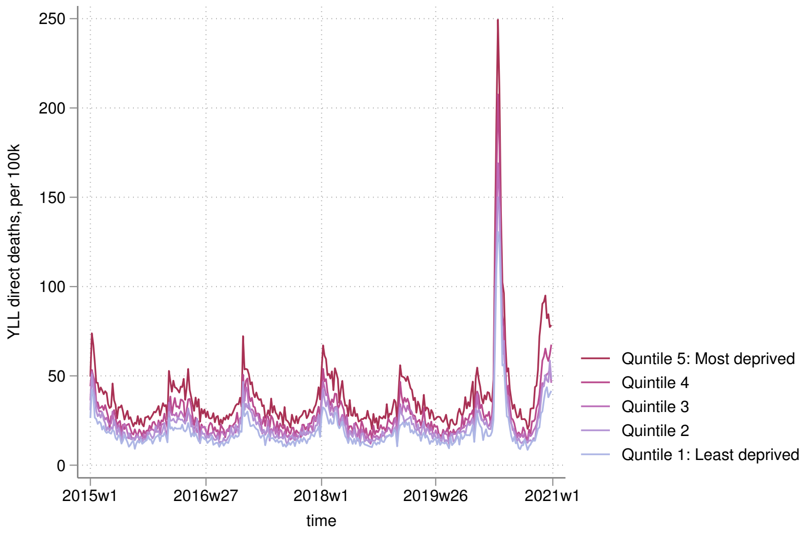

Figure 116: Years of Life Lost trend per 100,000 population, direct deaths by deprivation quintile, 2019-2020

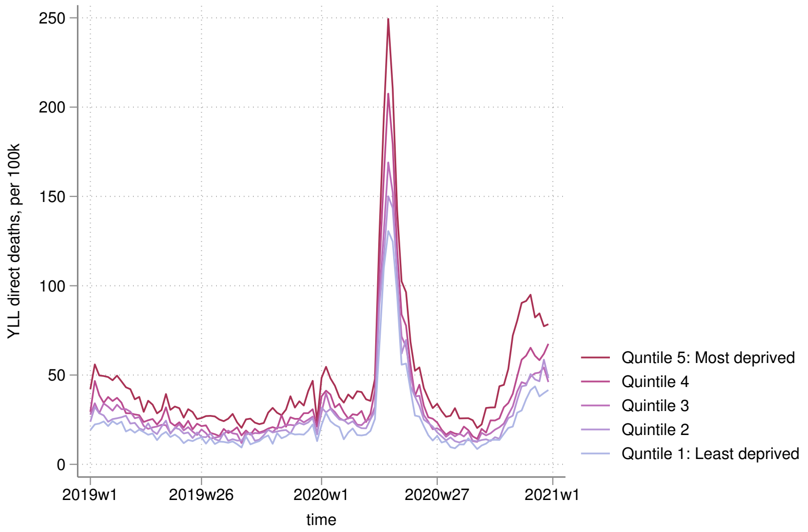

Figure 117: Excess Years of Life Lost trend per 100,000 population, direct deaths by deprivation quintile, 2015-2020

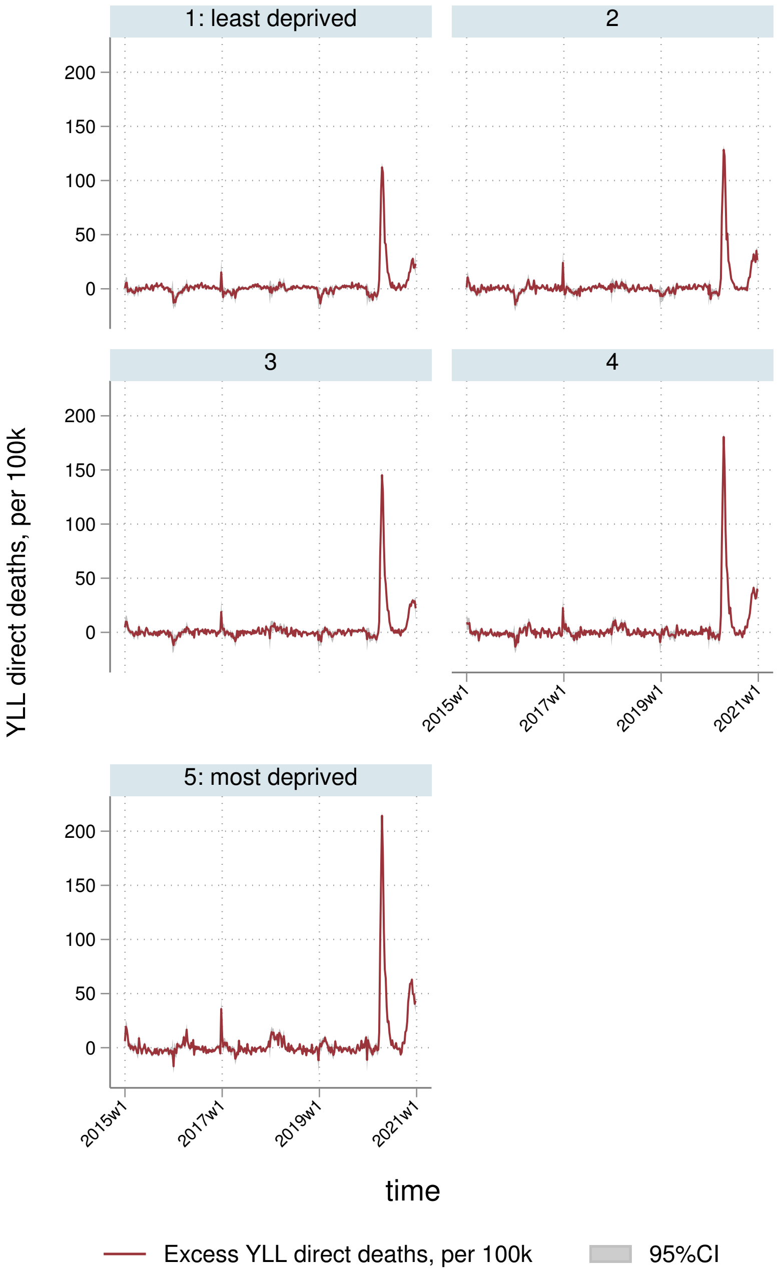

Figure 118: Excess Years of Life Lost trend per 100,000 population, direct deaths by deprivation quintile, 2019-2020

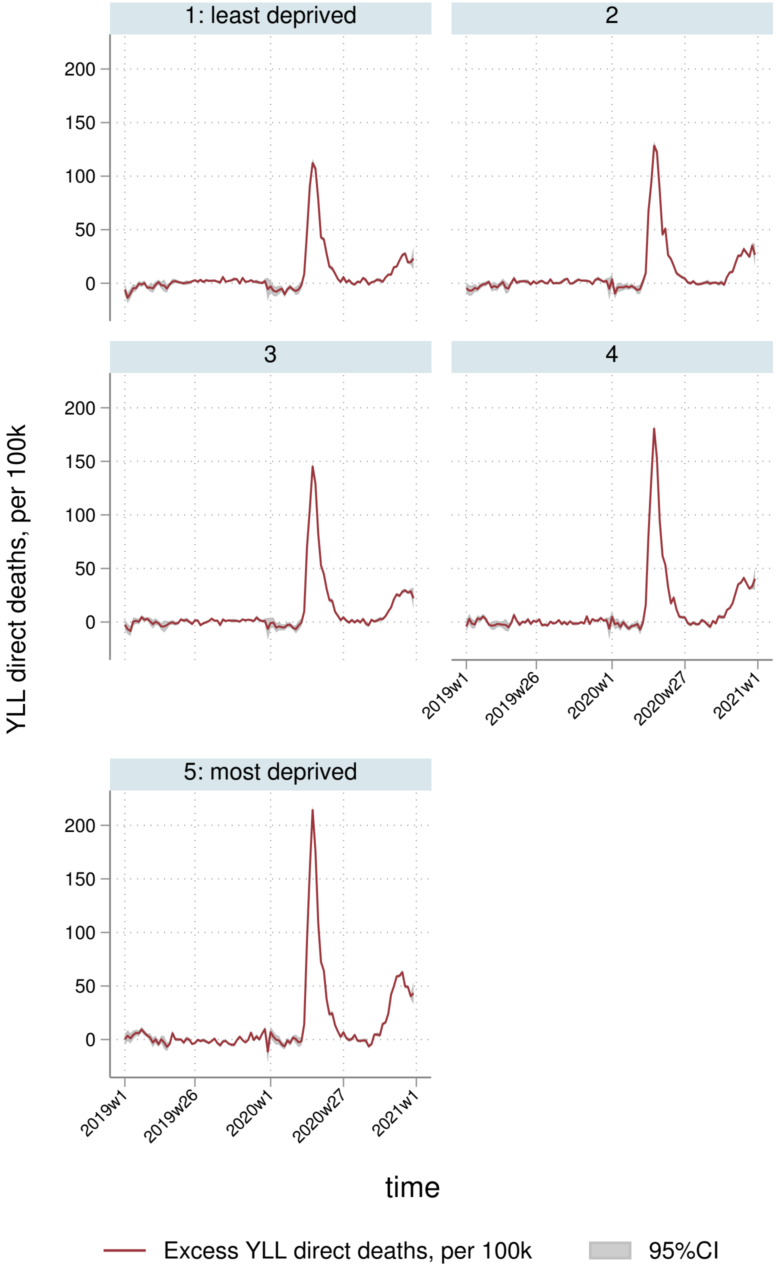

Figure 119: Observed vs Predicted Years of Life Lost trends per 100,000 population, direct deaths by deprivation quintile, 2015-2020

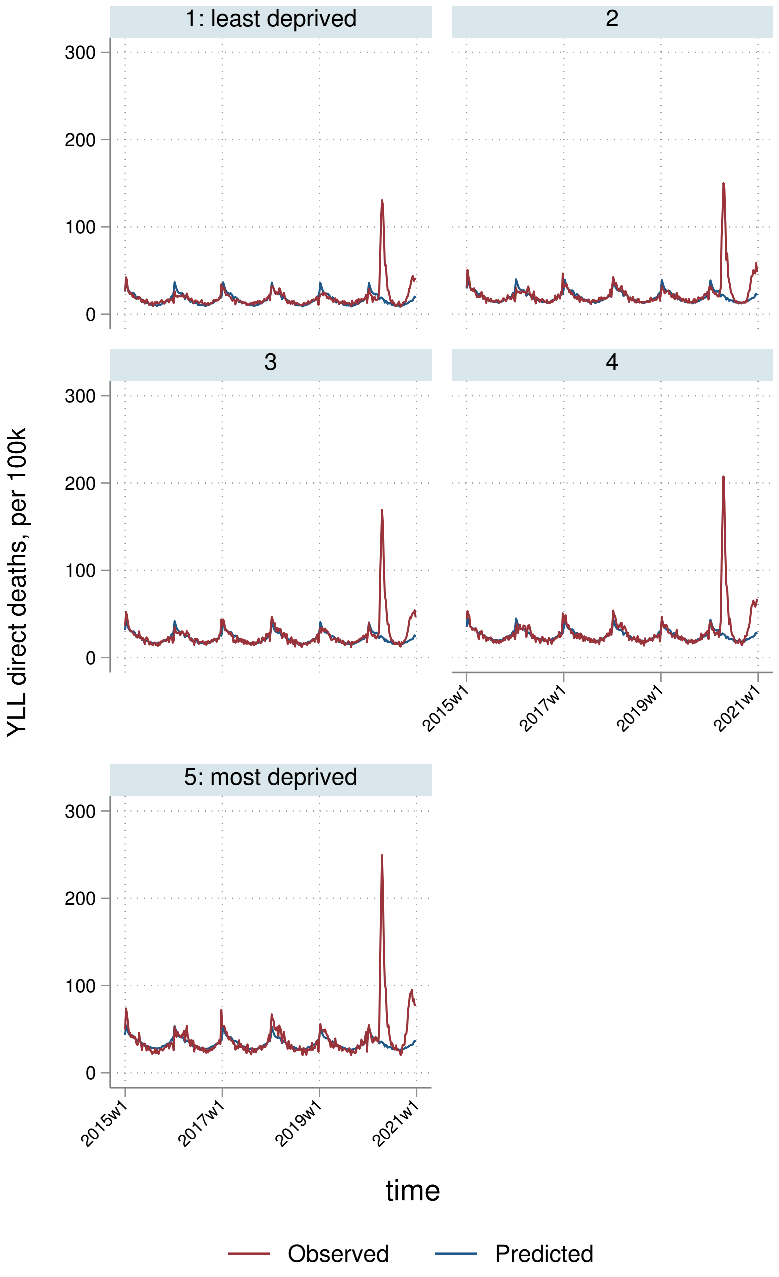

Figure 120: Observed vs Predicted Years of Life Lost trends per 100,000 population, direct deaths by deprivation quintile, 2019-2020

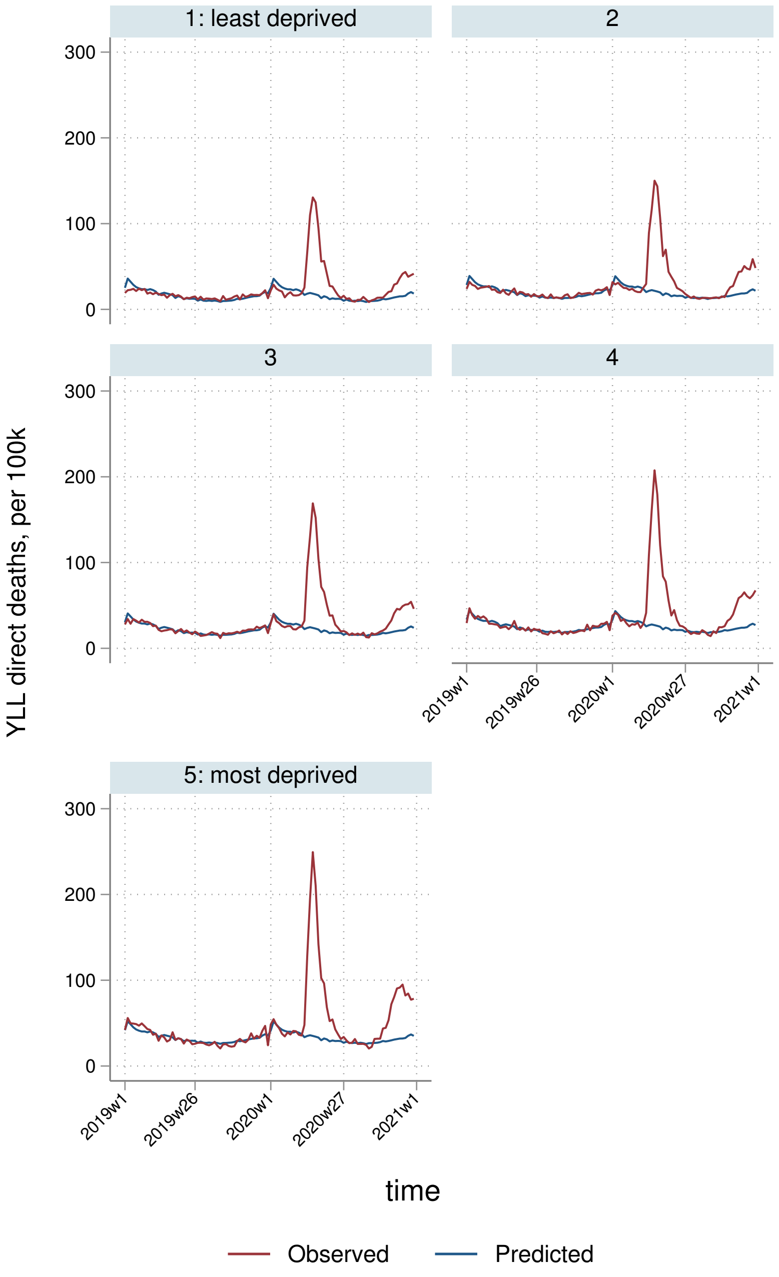

6.3.4 By Strategic Health Authority

Figure 121: Years of Life Lost trend per 100,000 population, direct deaths by region, 2015-2020

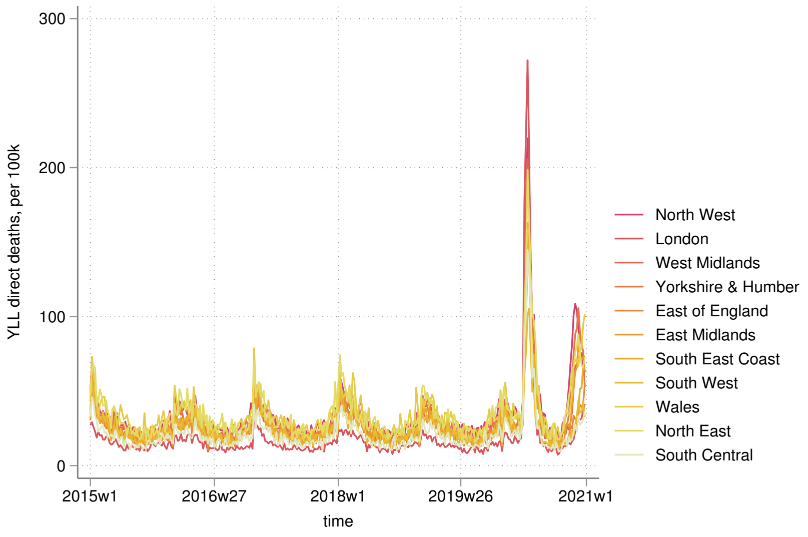

Figure 122: Years of Life Lost trend per 100,000 population, direct deaths by region, 2019-2020

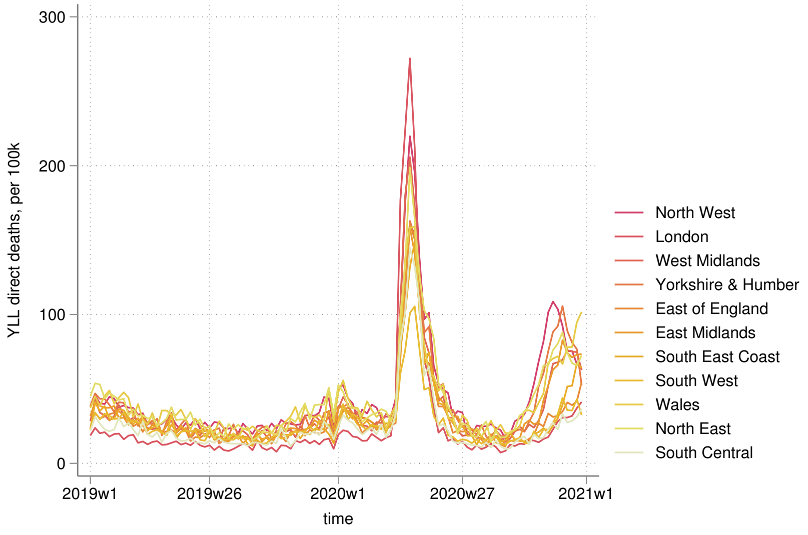

Figure 123: Excess Years of Life Lost trend per 100,000 population, direct deaths by region, 2015-2020

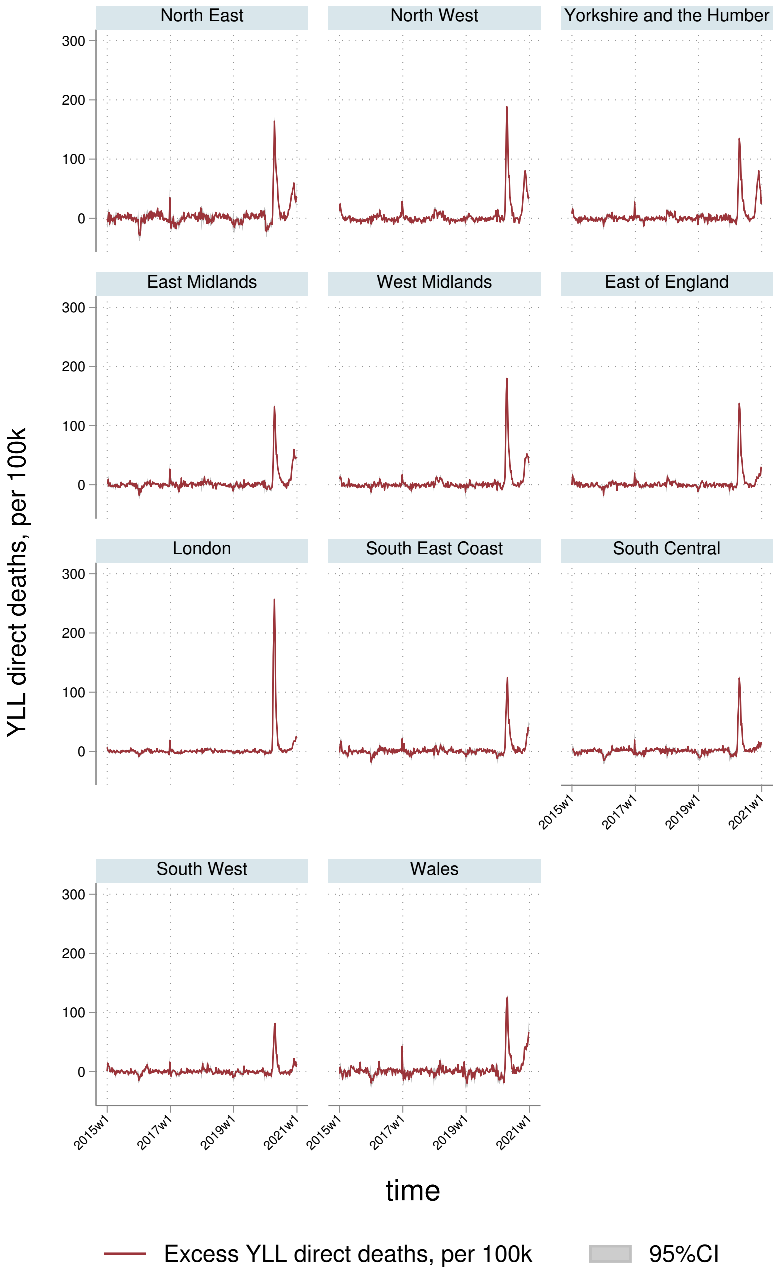

Figure 124: Excess Years of Life Lost trend per 100,000 population per 100,000 population, direct deaths by region, 2019-2020

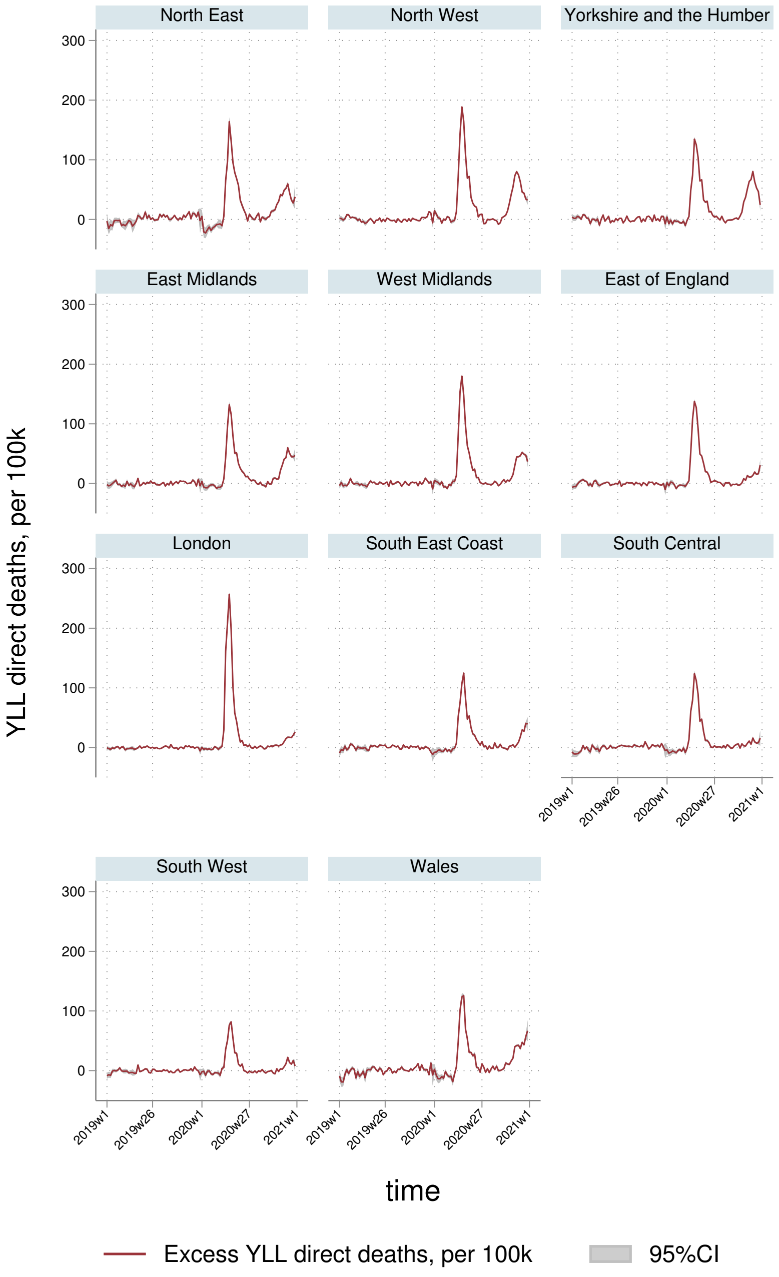

Figure 125: Observed vs Predicted Years of Life Lost trends per $100,000$ population, direct deaths by region, 2015-2020

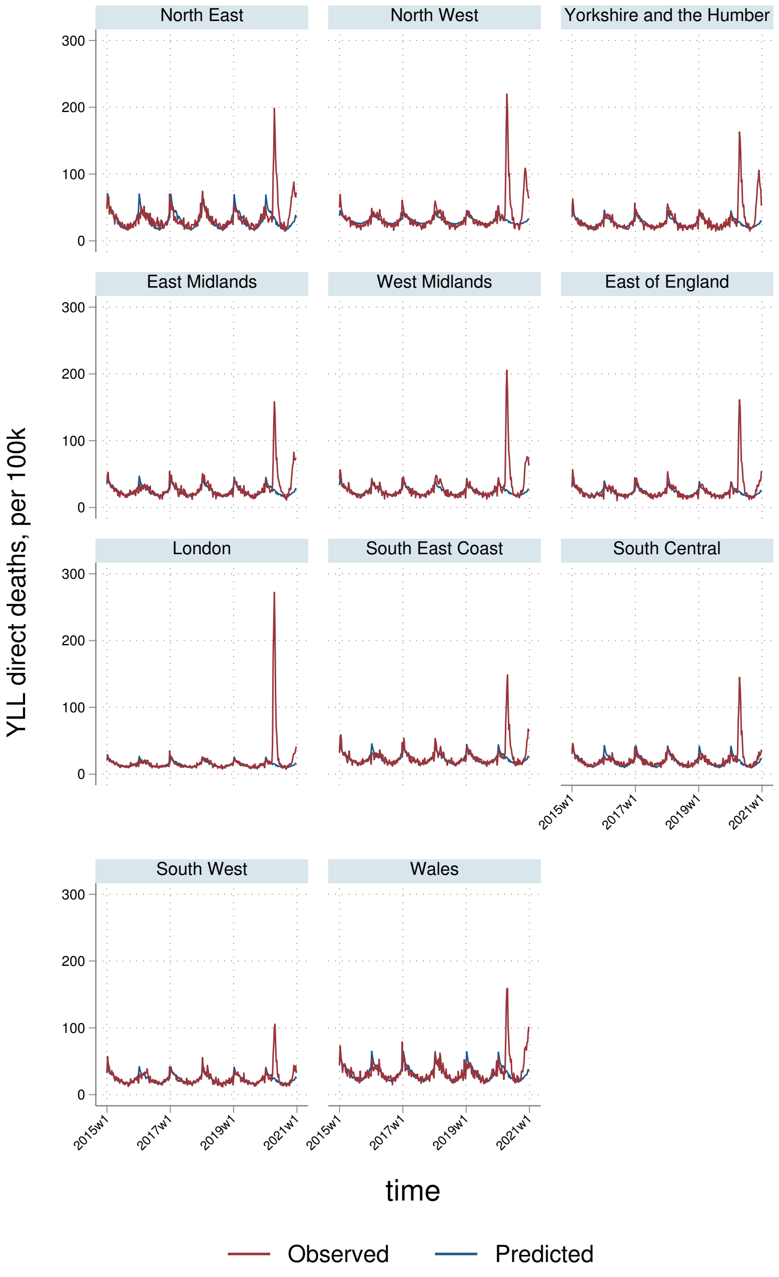

Figure 126: Observed vs Predicted Years of Life Lost trends per 100,000 population, direct deaths by region, 2019-2020

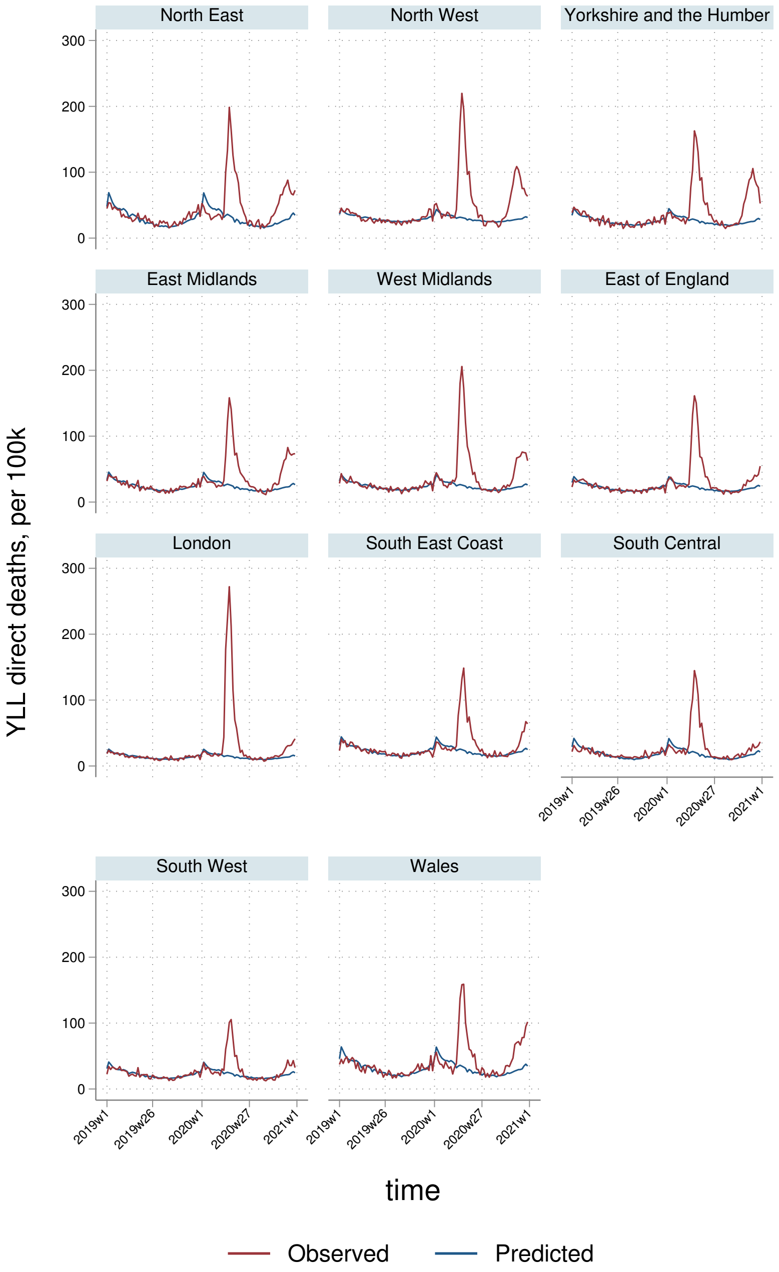

6.3.5 By deprivation quintile & Strategic Health Authority

Figure 127: Years of Life Lost trend per 100,000 population, direct deaths by regionXdeprivation, 2015-2020

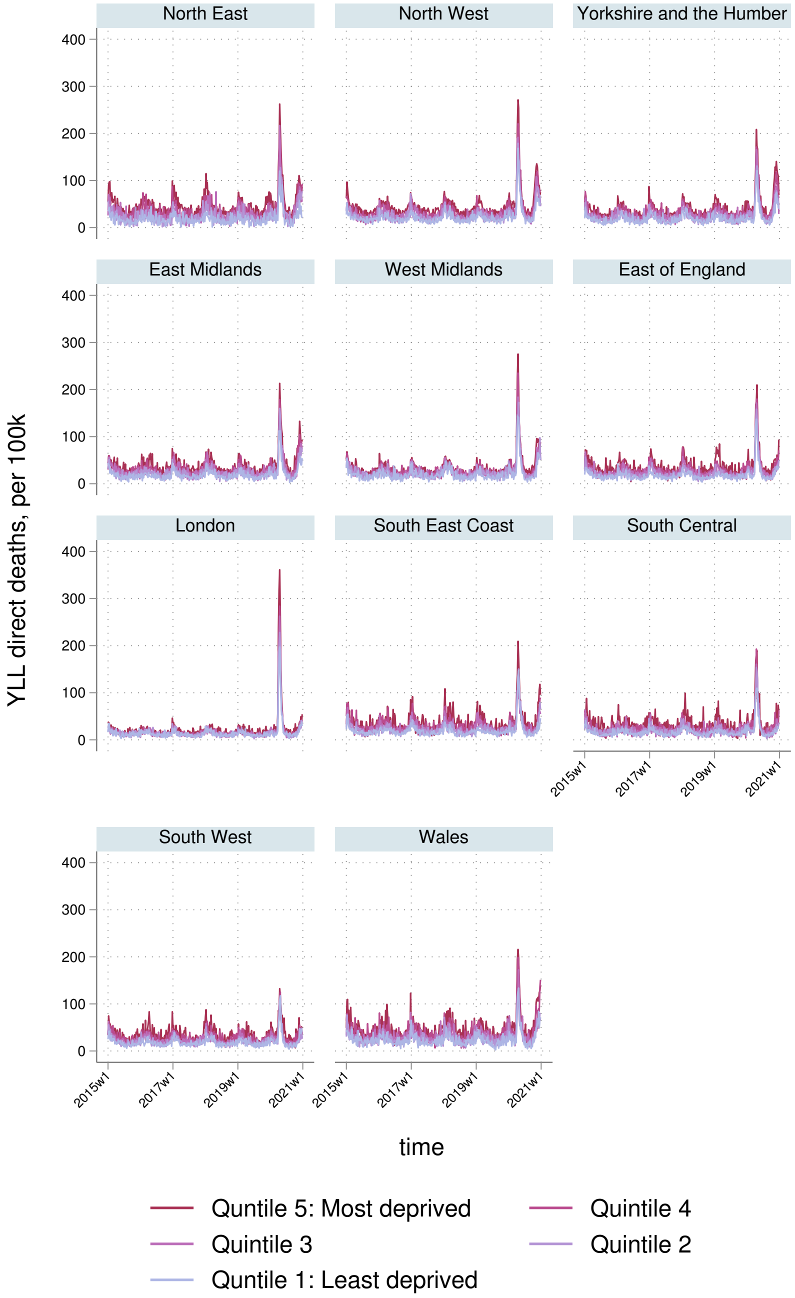

Figure 128: Years of Life Lost trend per 100,000 population, direct deaths by region, 2019-2020

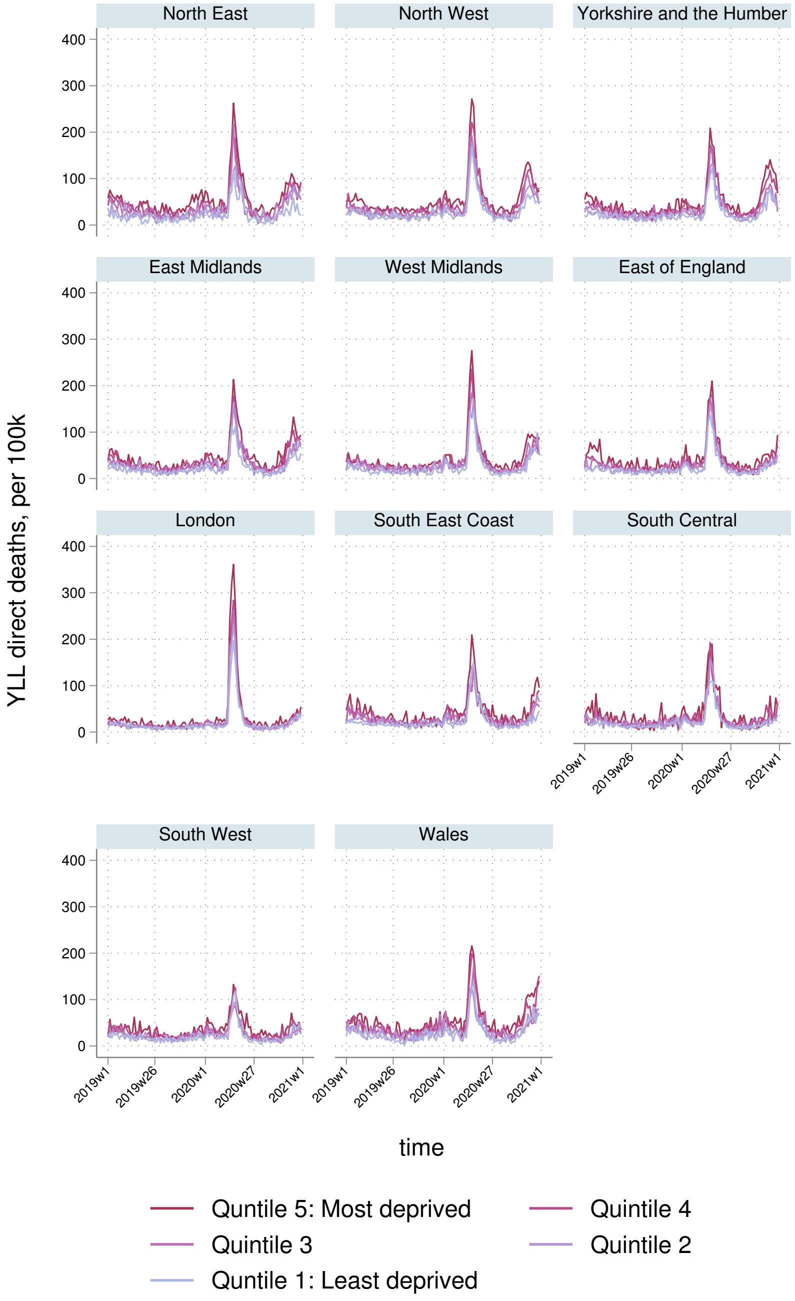

Figure 129: Excess Years of Life Lost trend per 100,000 population, direct deaths by regionXdeprivation, 2015-2020

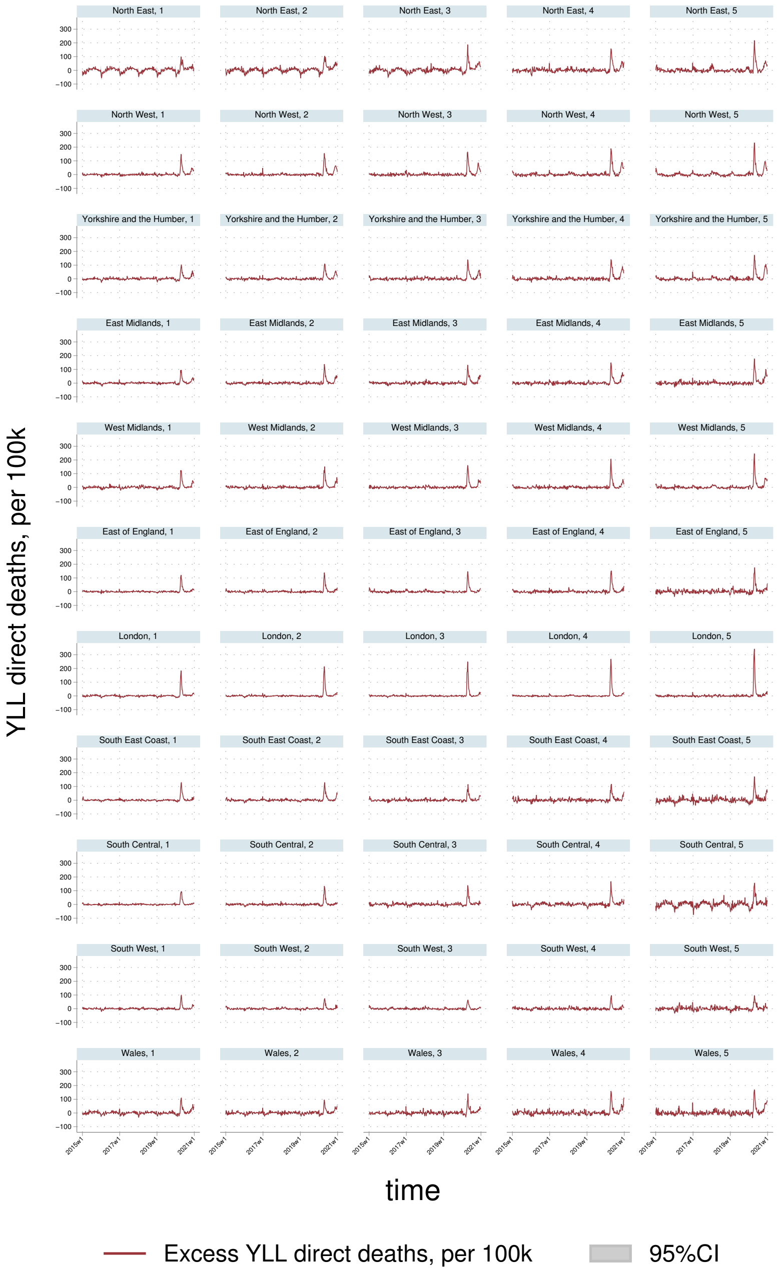

Figure 130: Excess Years of Life Lost trend per 100,000 population, direct deaths by regionXdeprivation, 2019-2020

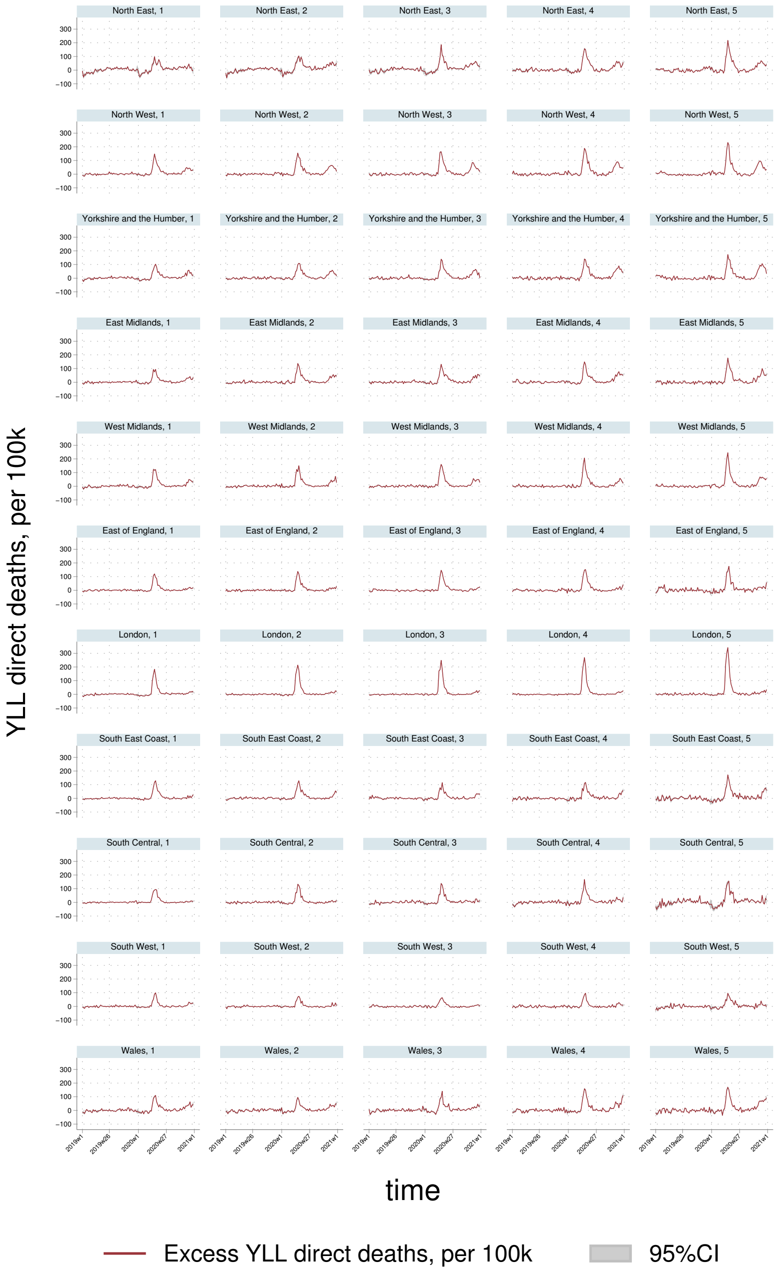

Figure 131: Observed vs Predicted Years of Life Lost trends per 100,000 population, direct deaths by regionXdeprivation, 2015-2020

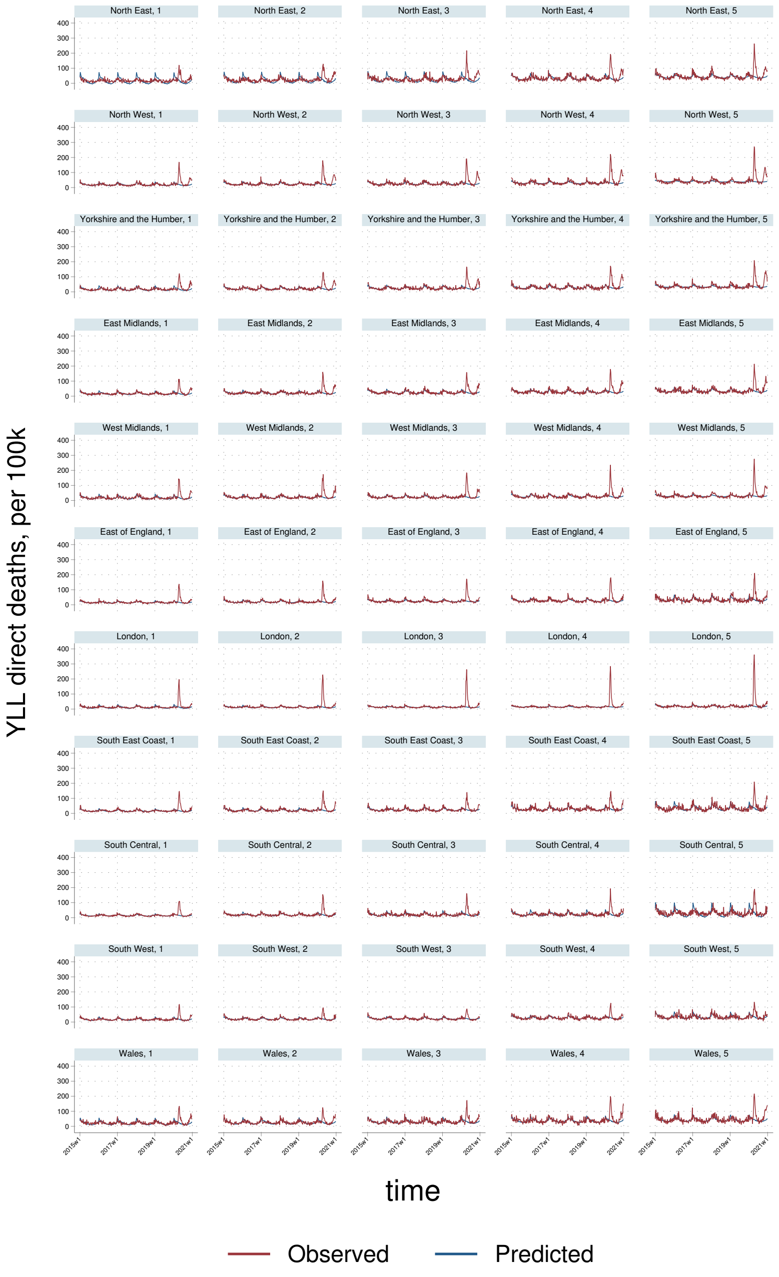

Figure 132: Observed vs Predicted Years of Life Lost trends per 100,000 population, direct deaths by regionXdeprivation, 2019-2020

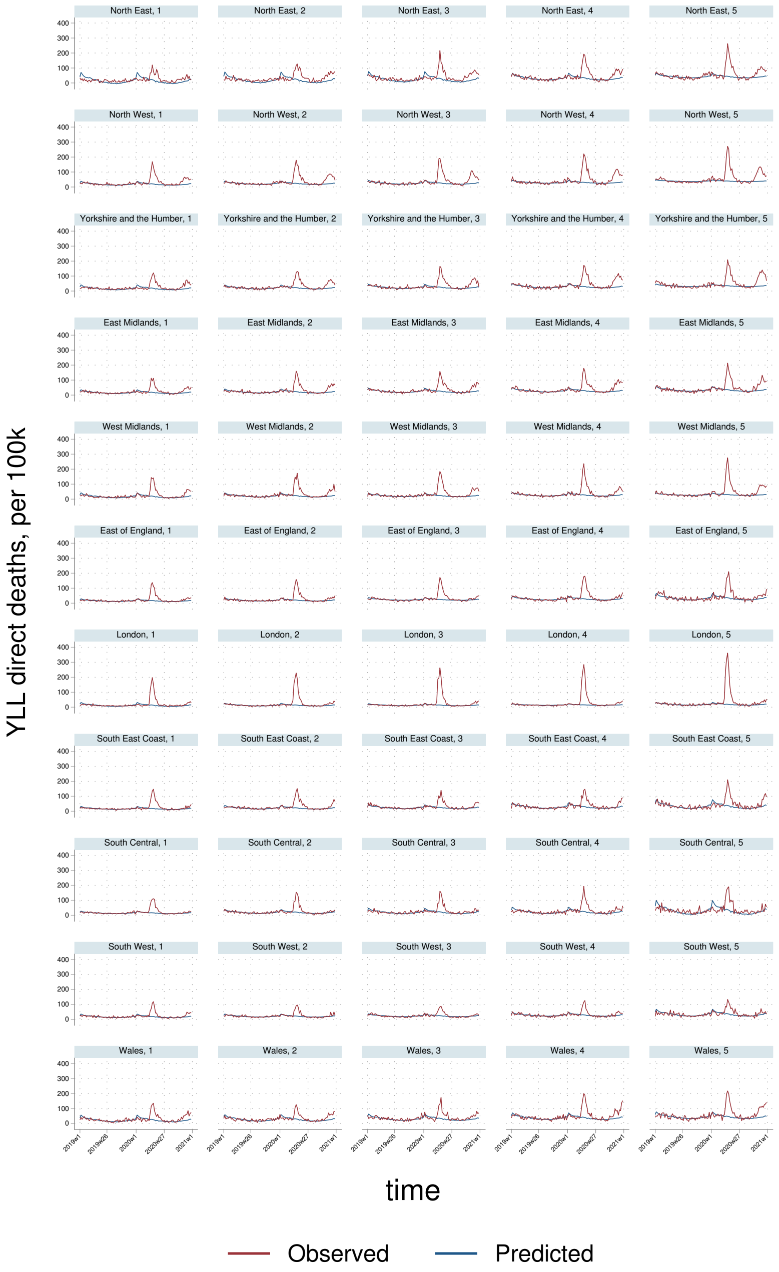

7 Cardiovascular & diabetes

7.1 AASMRs

Figure 133: Age-standardised mortality trend, cardiovascular & diabetes deaths, 2015-2020

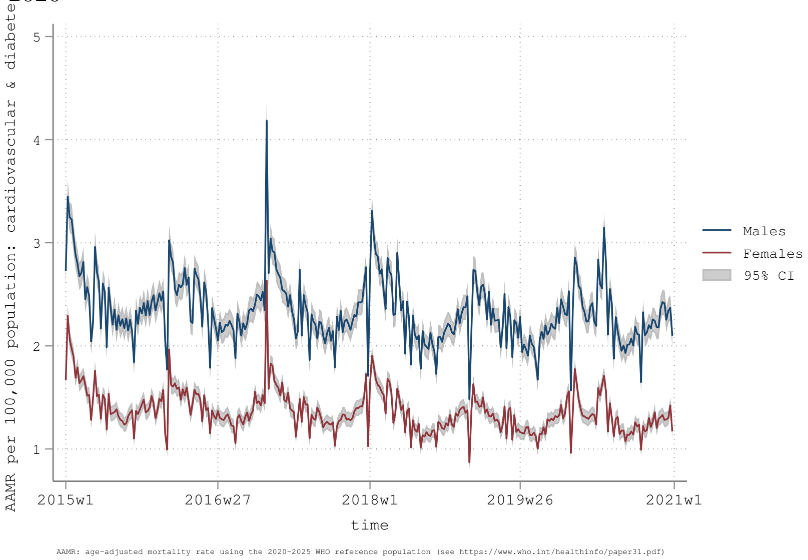

Figure 134: Age-standardised mortality trend, cardiovascular & diabetes deaths, 2019-2020

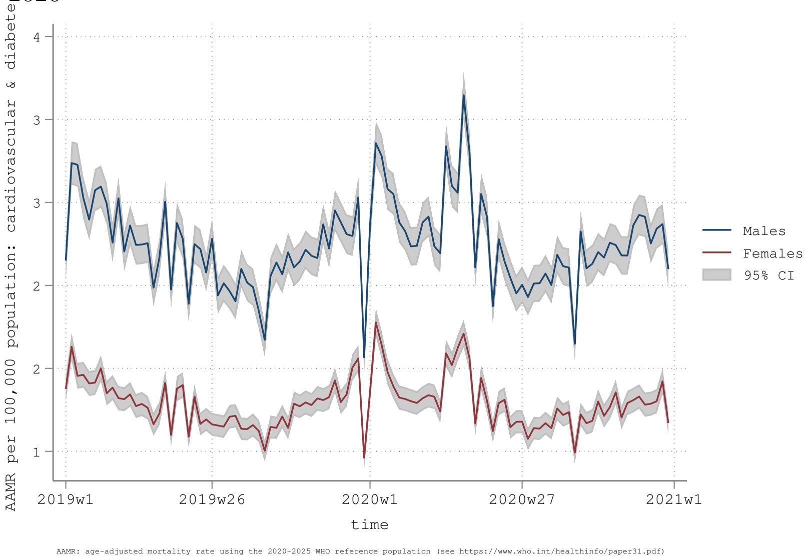

Figure 135: Age-standardised mortality trend, cardiovascular & diabetes deaths by deprivation quintile, 2015-2020

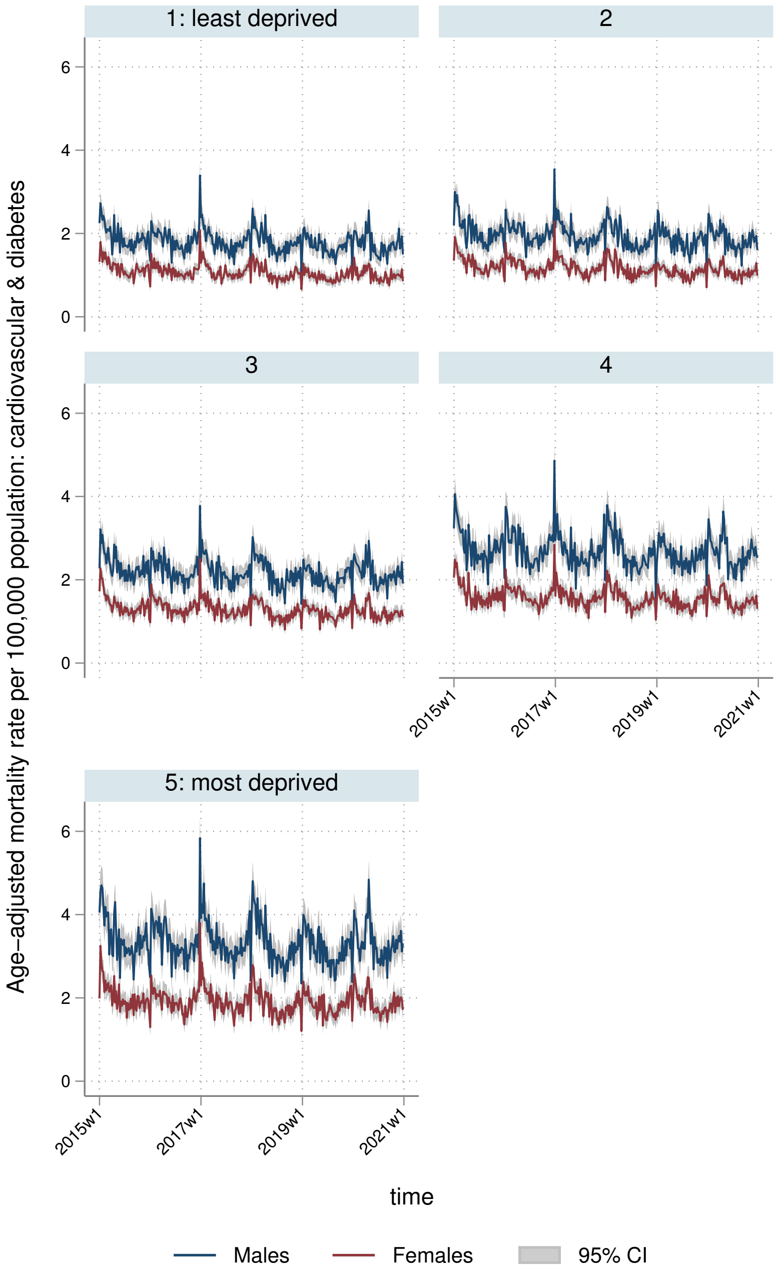

Figure 136: Age-standardised mortality trend, cardiovascular & diabetes deaths by deprivation quintile, 2019-2020

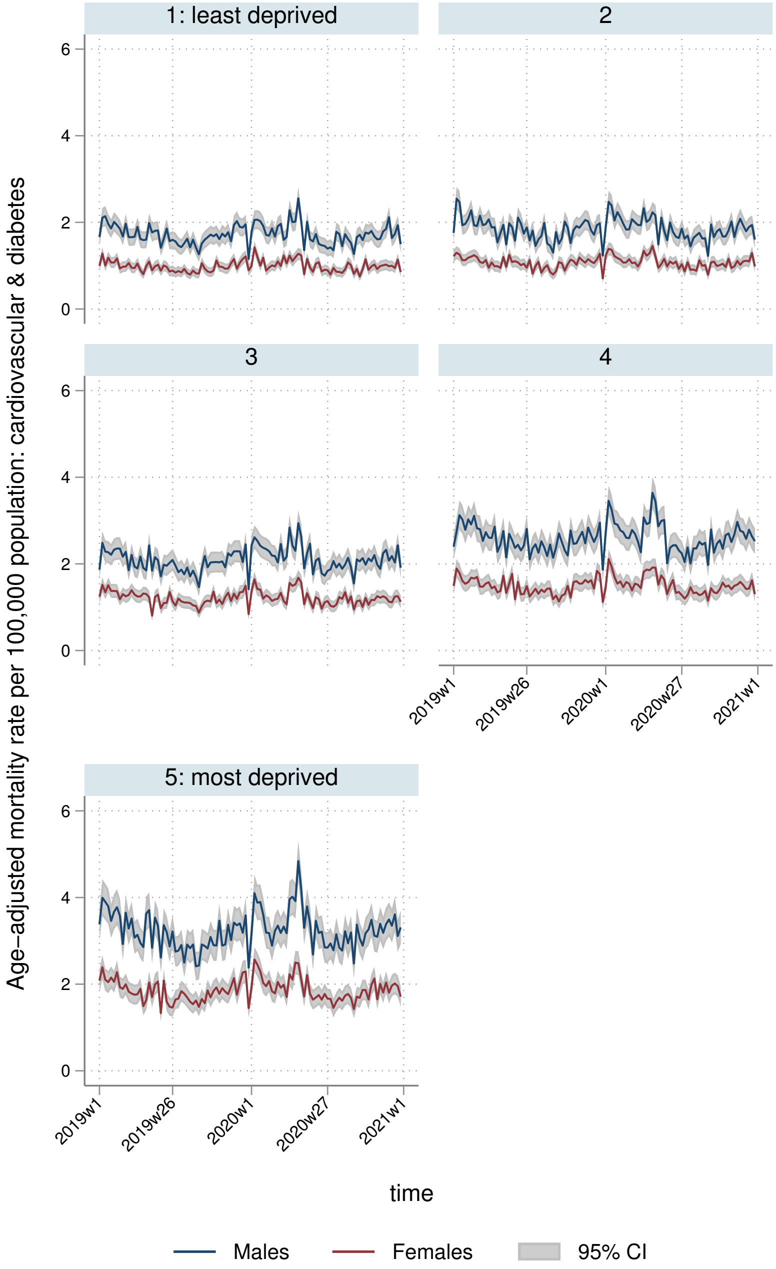

Figure 137: Age-standardised mortality trend, cardiovascular & diabetes deaths by region, 2015-2020

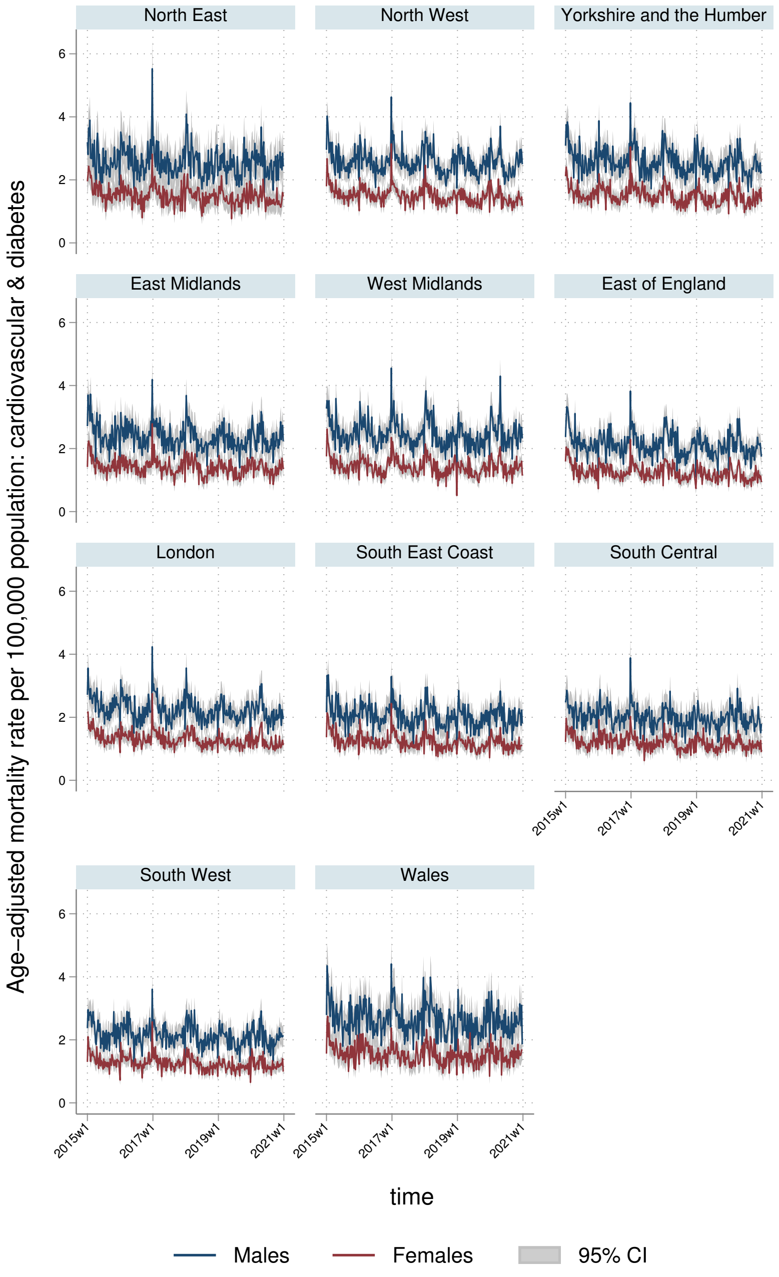

7.2 YLLs

7.2.1 England-Wales aggregate

Figure 138: Age-standardised mortality trend, cardiovascular & diabetes deaths by region, 2019-2020

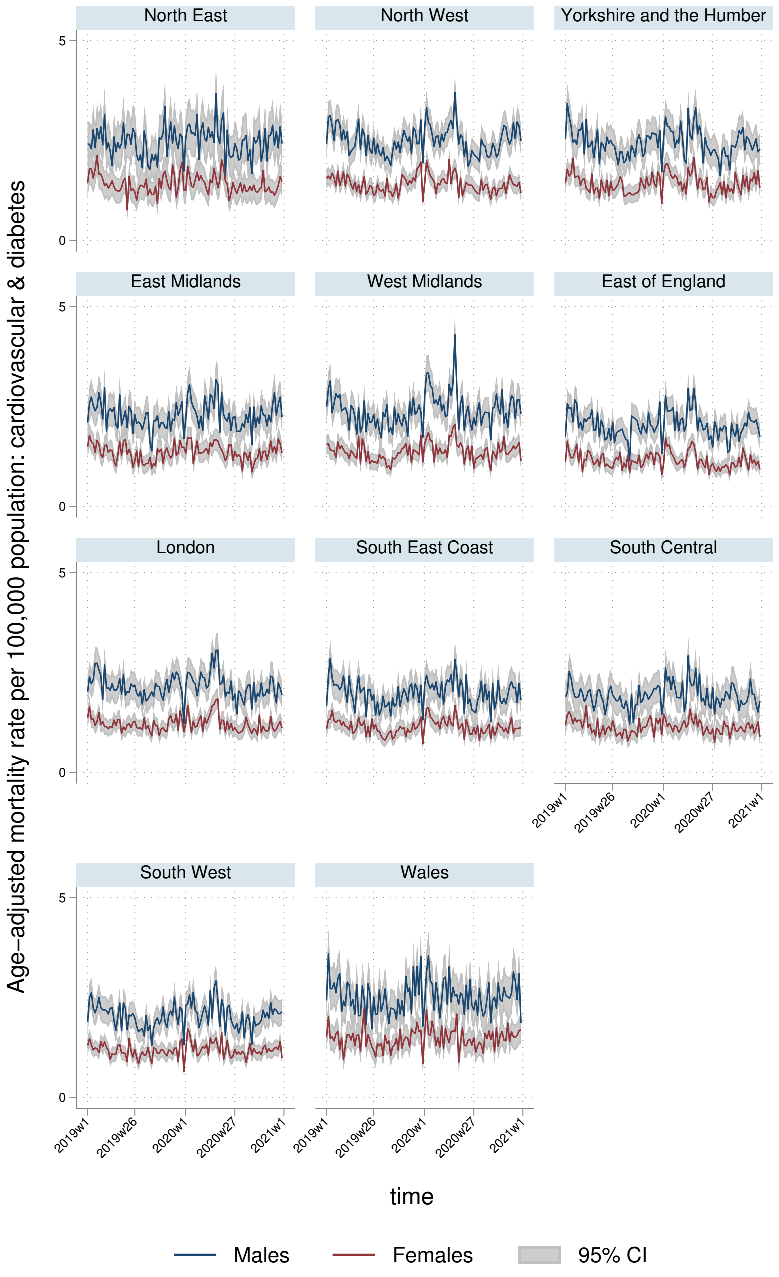

Figure 139: Years of Life Lost trend, cardiovascular & diabetes deaths, 2015-2020

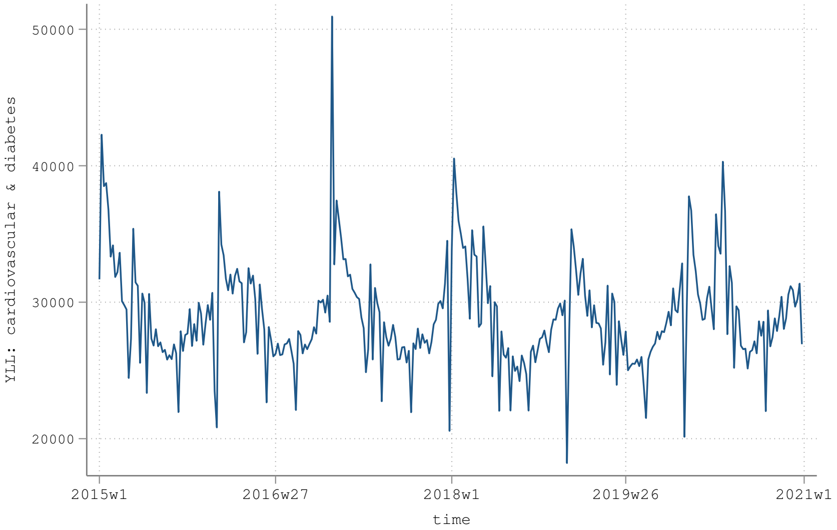

Figure 140: Years of Life Lost trend, cardiovascular & diabetes deaths, 2019-2020

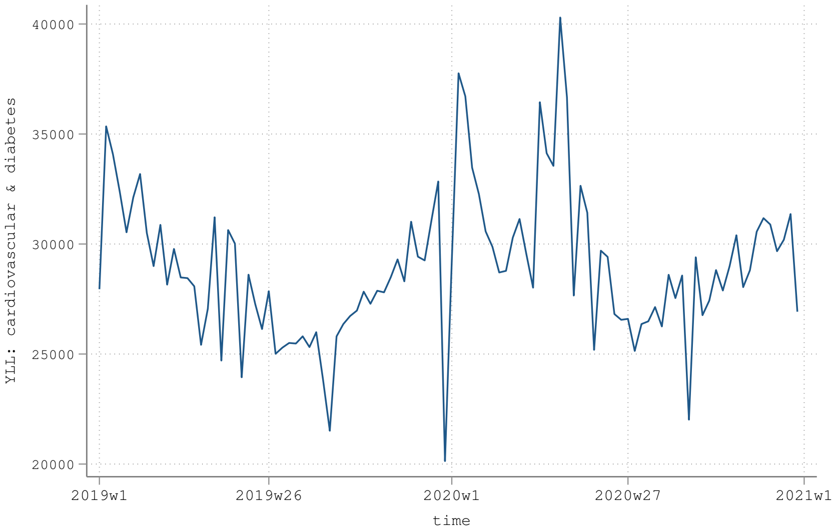

Figure 141: Excess Years of Life Lost trend, cardiovascular & diabetes deaths, 2015-2020

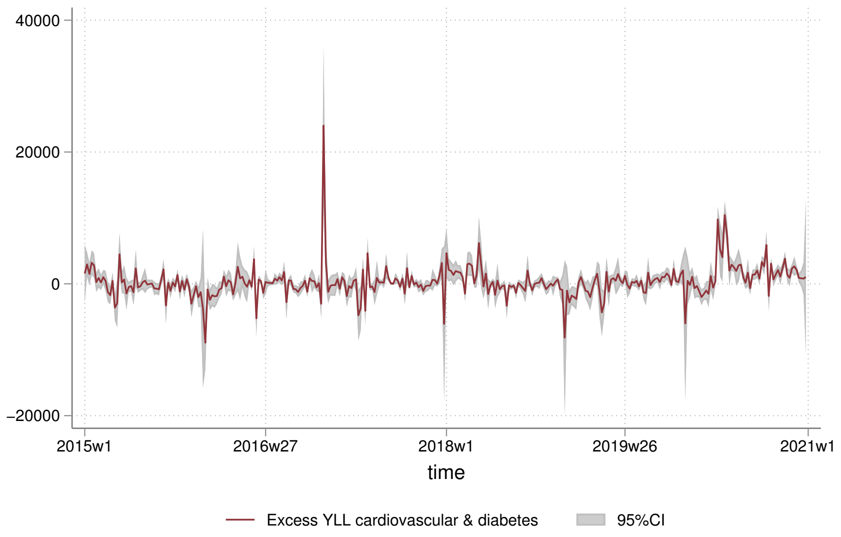

Figure 142: Excess Years of Life Lost trend, cardiovascular & diabetes deaths, 2019-2020

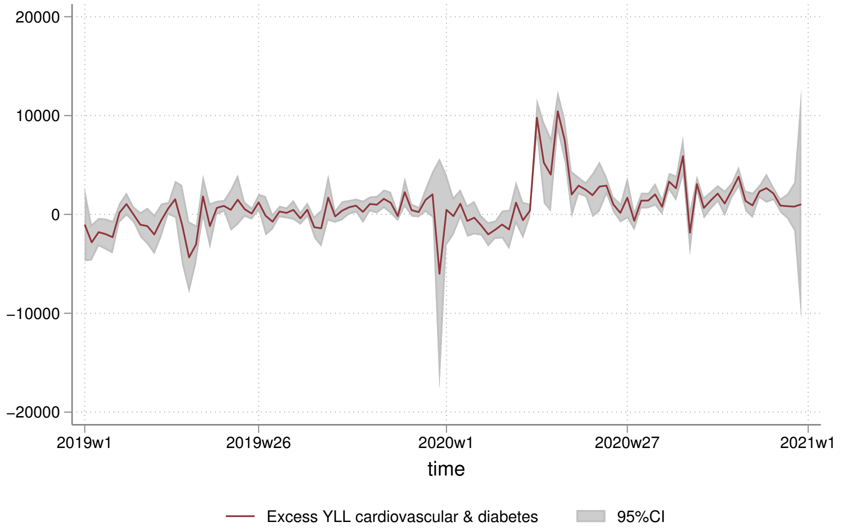

Figure 143: Observed vs Predicted Years of Life Lost trends, cardiovascular & diabetes deaths, 2015-2020

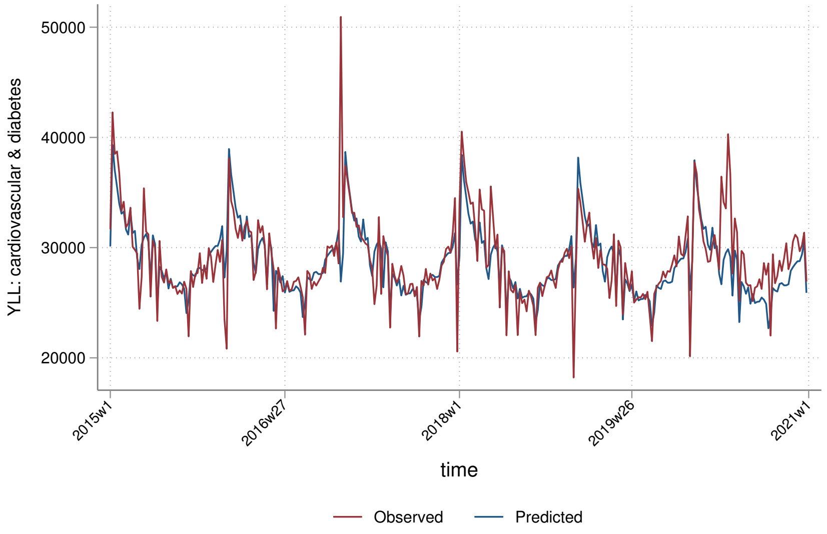

Figure 144: Observed vs Predicted Years of Life Lost trends, cardiovascular & diabetes deaths, 2019-2020

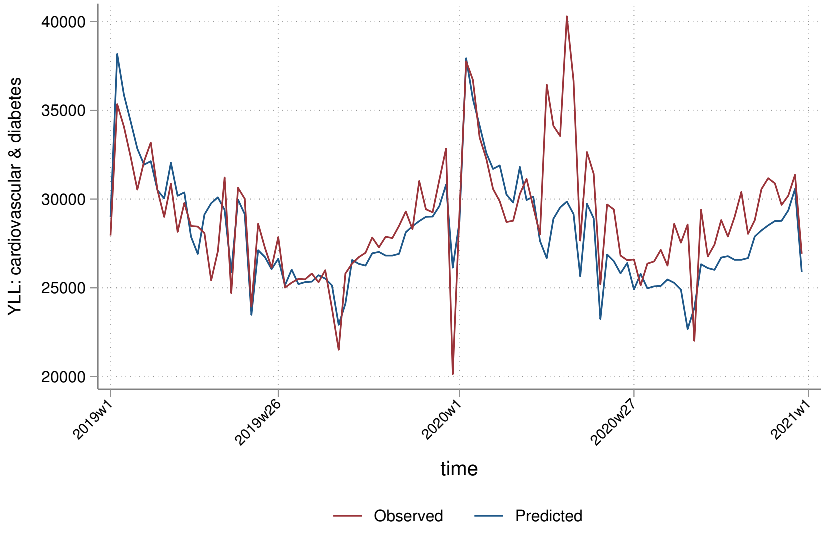

7.2.2 By sex

Figure 145: Years of Life Lost trend, cardiovascular & diabetes deaths by sex, 2015-2020

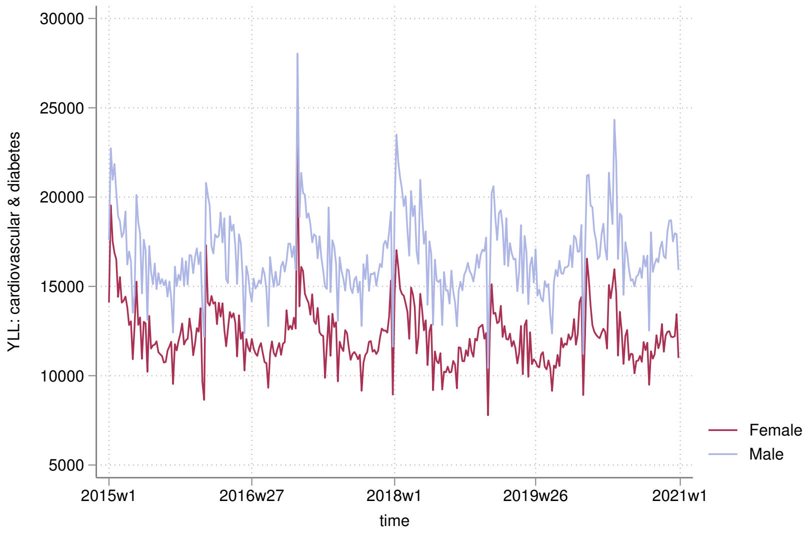

Figure 146: Years of Life Lost trend, cardiovascular & diabetes deaths by sex, 2019-2020

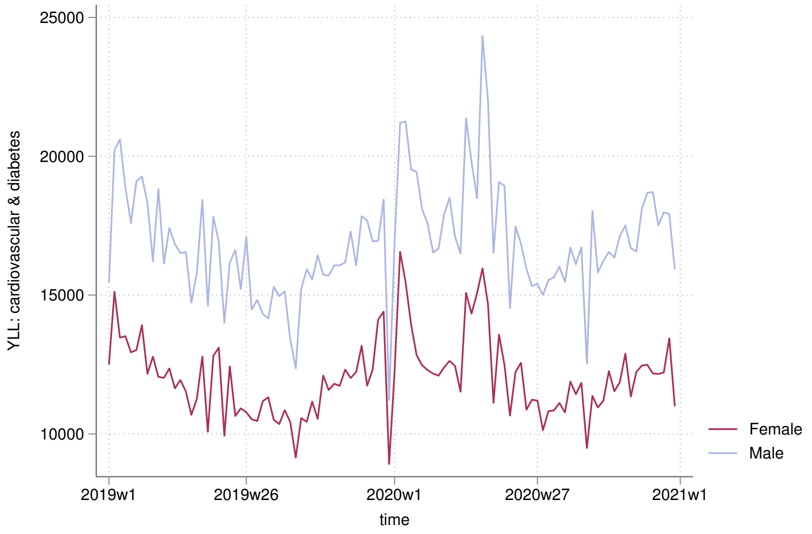

Figure 147: Excess Years of Life Lost trend, cardiovascular & diabetes deaths by sex, 2015-2020

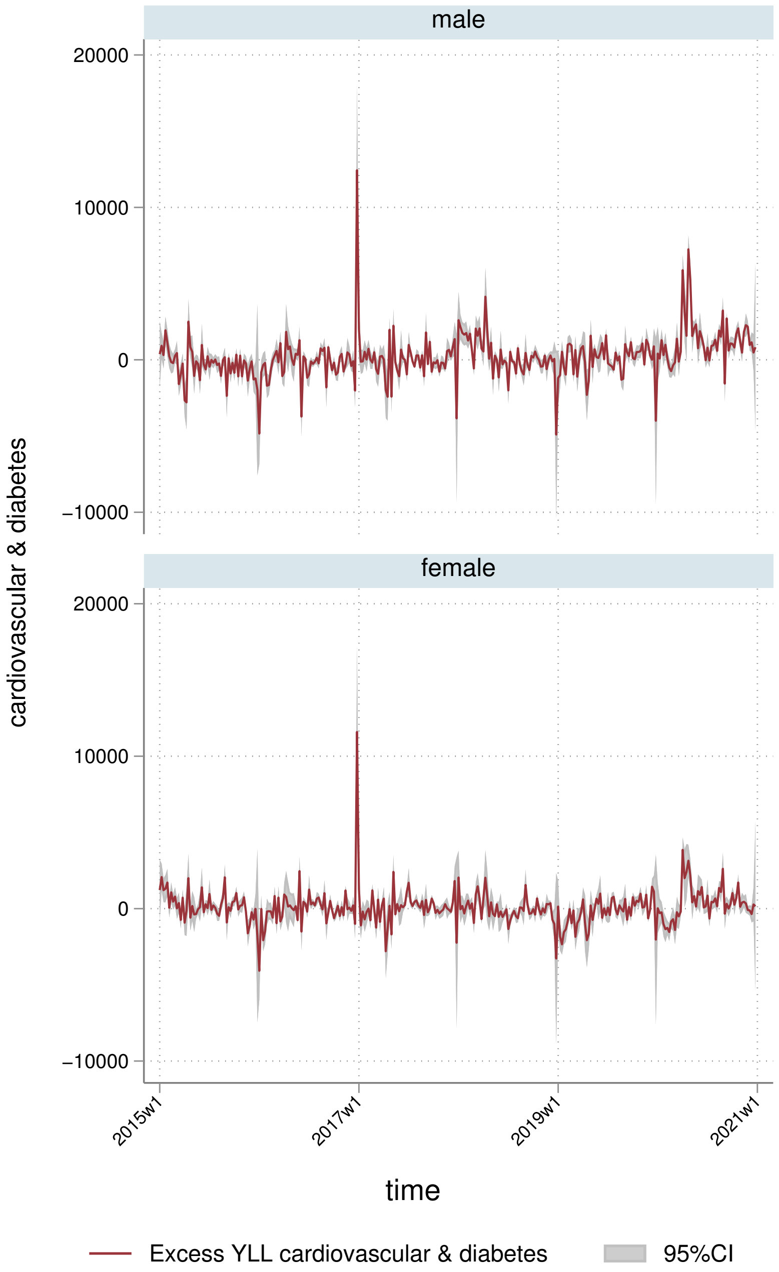

Figure 148: Excess Years of Life Lost trend, cardiovascular & diabetes deaths by sex, 2019-2020

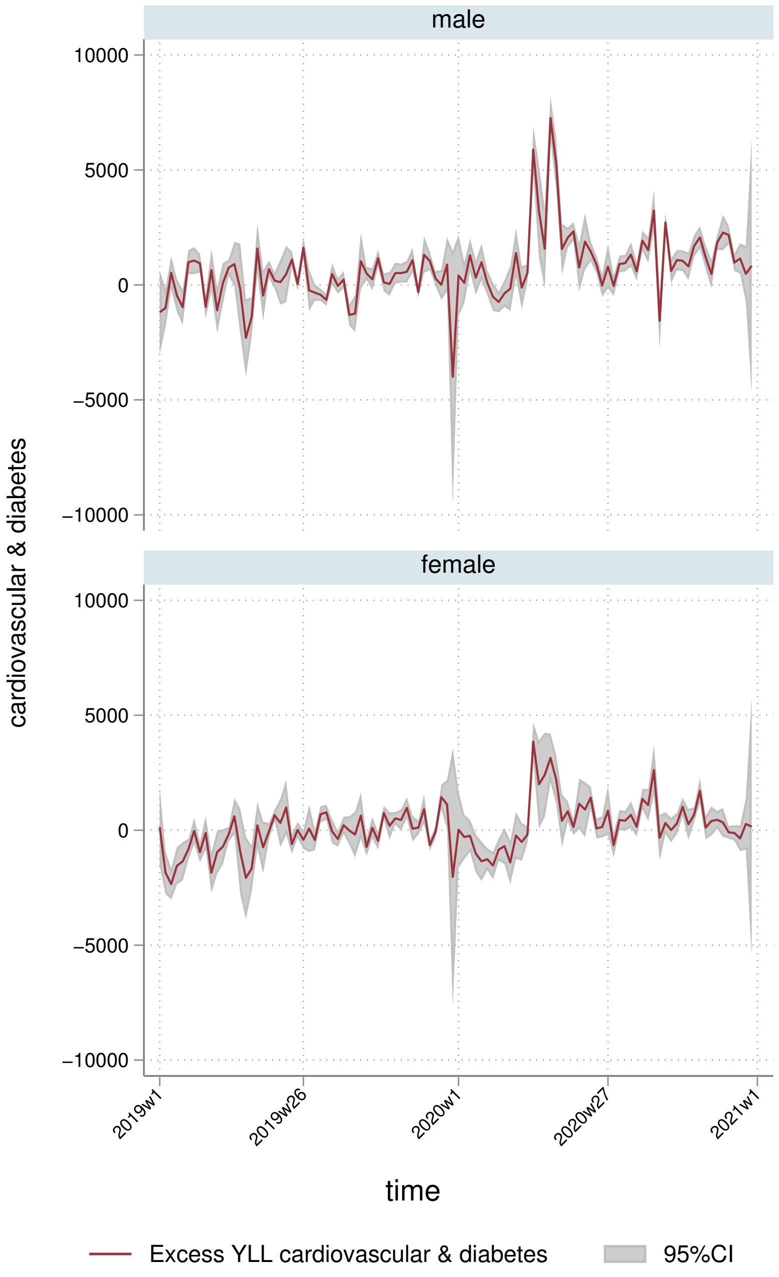

Figure 149: Observed vs Predicted Years of Life Lost trends, cardiovascular & diabetes deaths by sex, 2015-2020

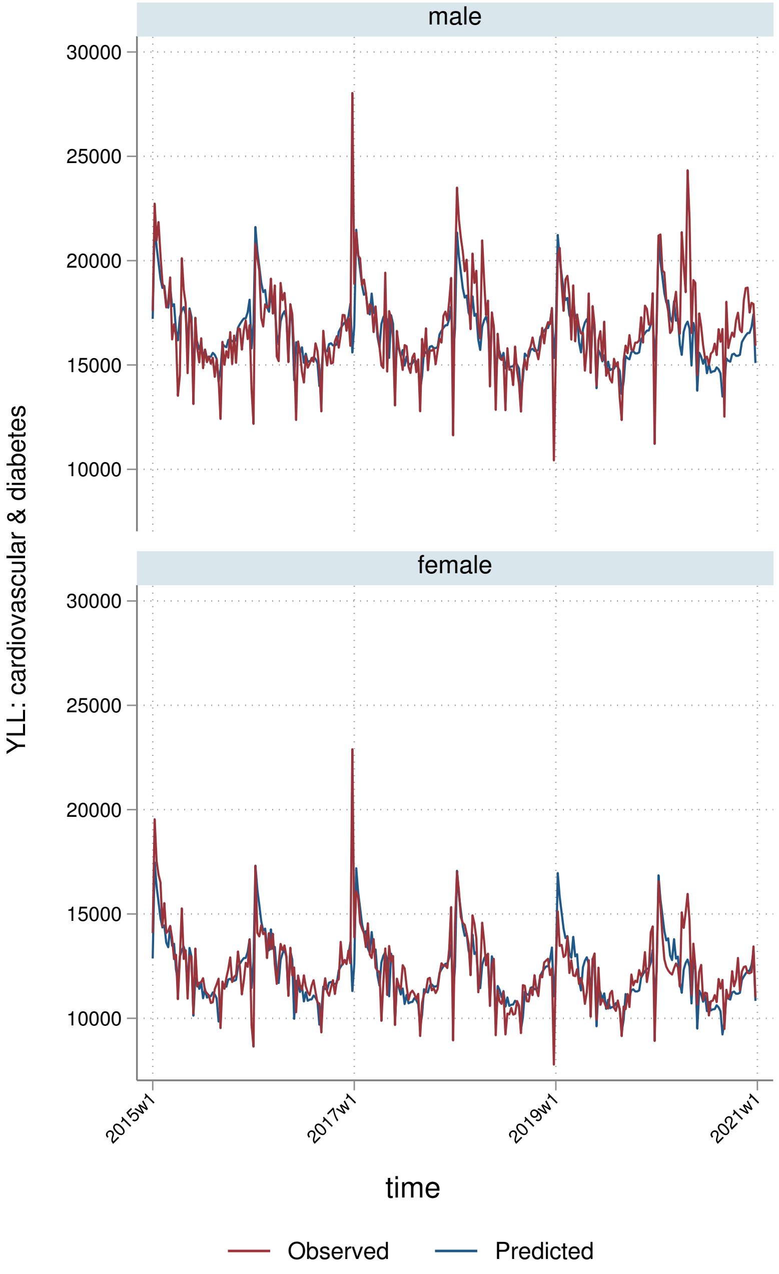

Figure 150: Observed vs Predicted Years of Life Lost trends, cardiovascular & diabetes deaths by sex, 2019-2020

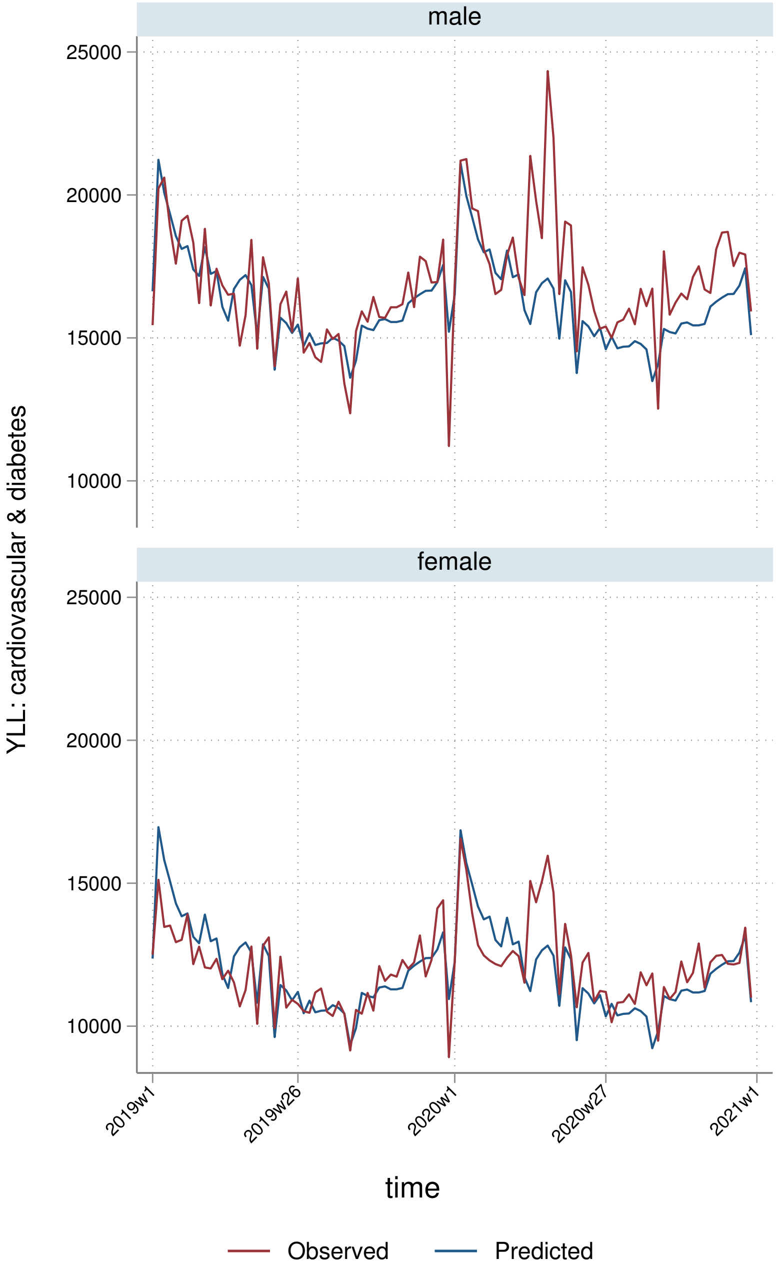

7.2.3 By deprivation quintile

Figure 151: Years of Life Lost trend, cardiovascular & diabetes deaths by deprivation quintile, 2015-2020

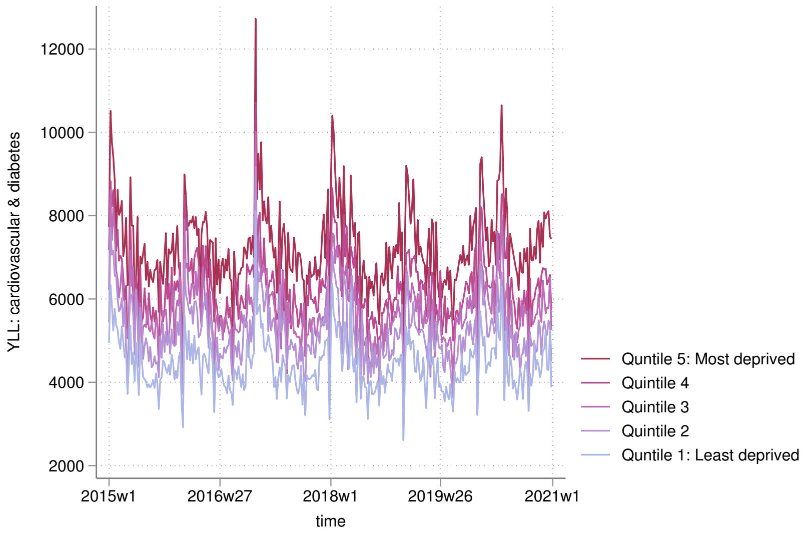

Figure 152: Years of Life Lost trend, cardiovascular & diabetes deaths by deprivation quintile, 2019-2020

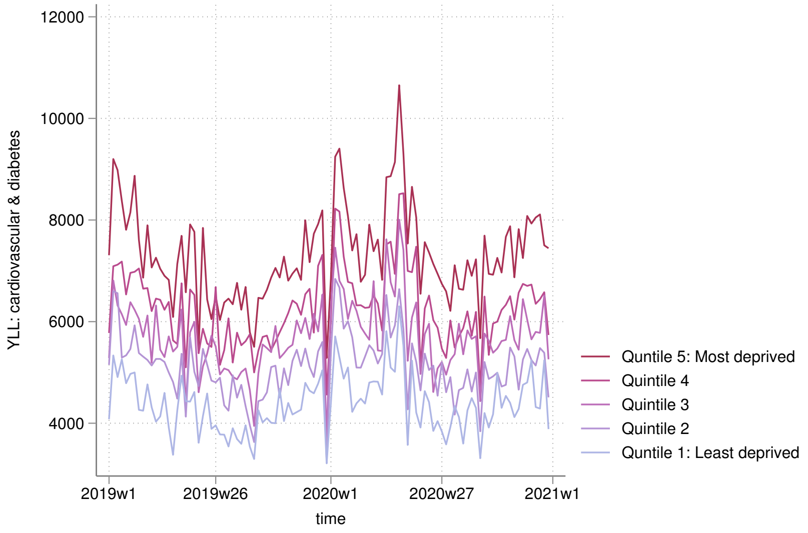

Figure 153: Excess Years of Life Lost trend, cardiovascular & diabetes deaths by deprivation quintile, 2015-2020

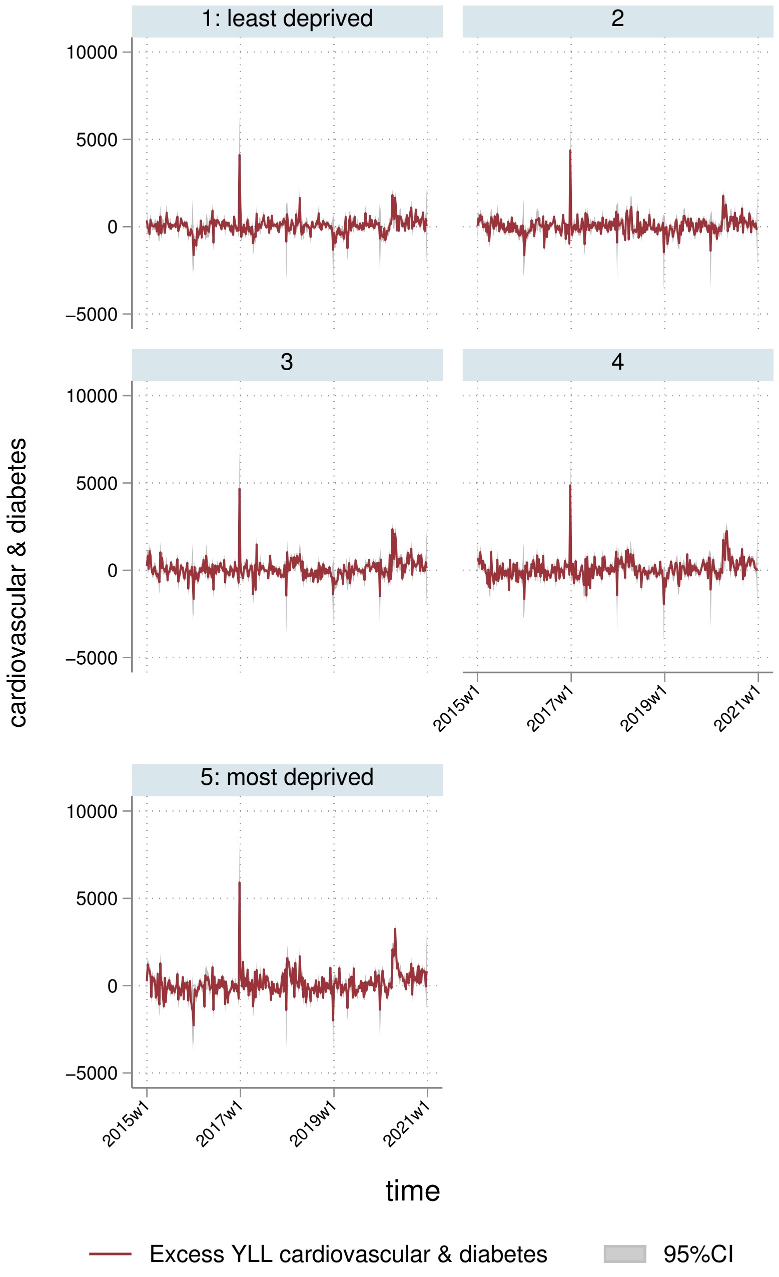

Figure 154: Excess Years of Life Lost trend, cardiovascular & diabetes deaths by deprivation quintile, 2019-2020

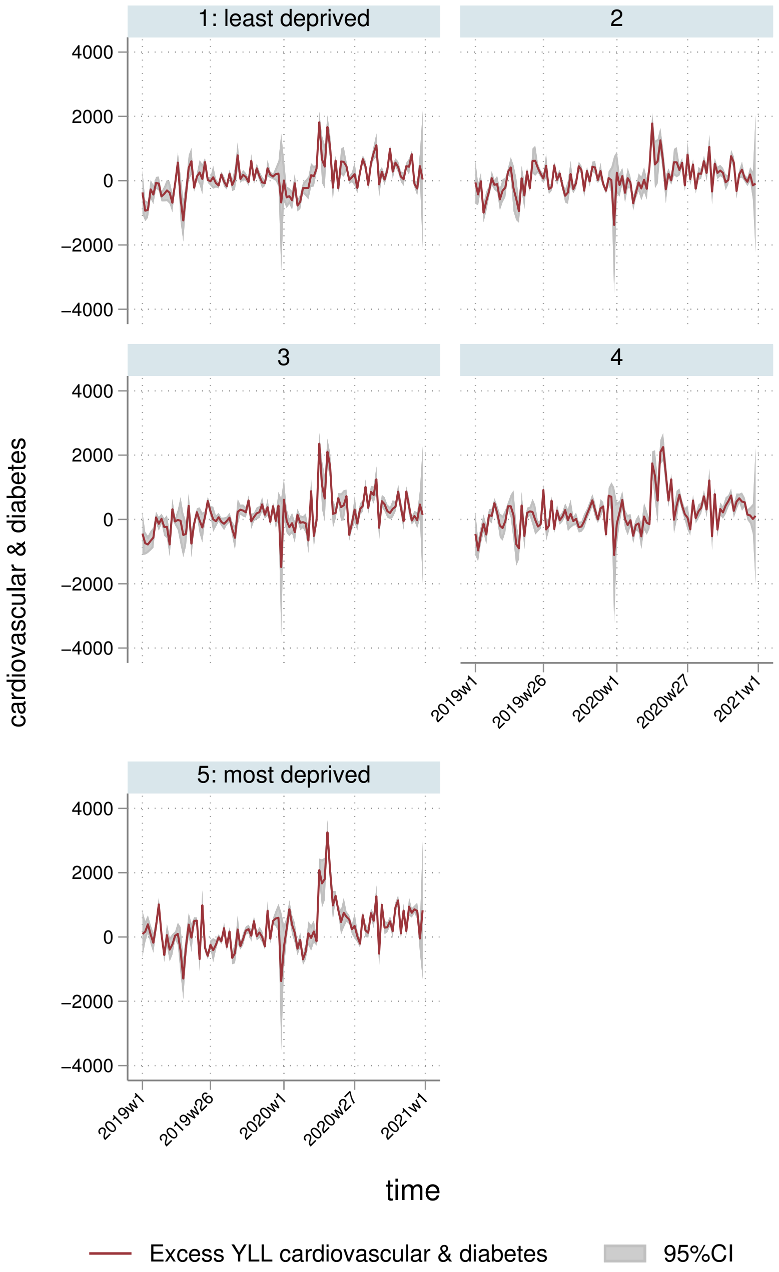

Figure 155: Observed vs Predicted Years of Life Lost trends, cardiovascular & diabetes deaths by deprivation quintile, 2015-2020

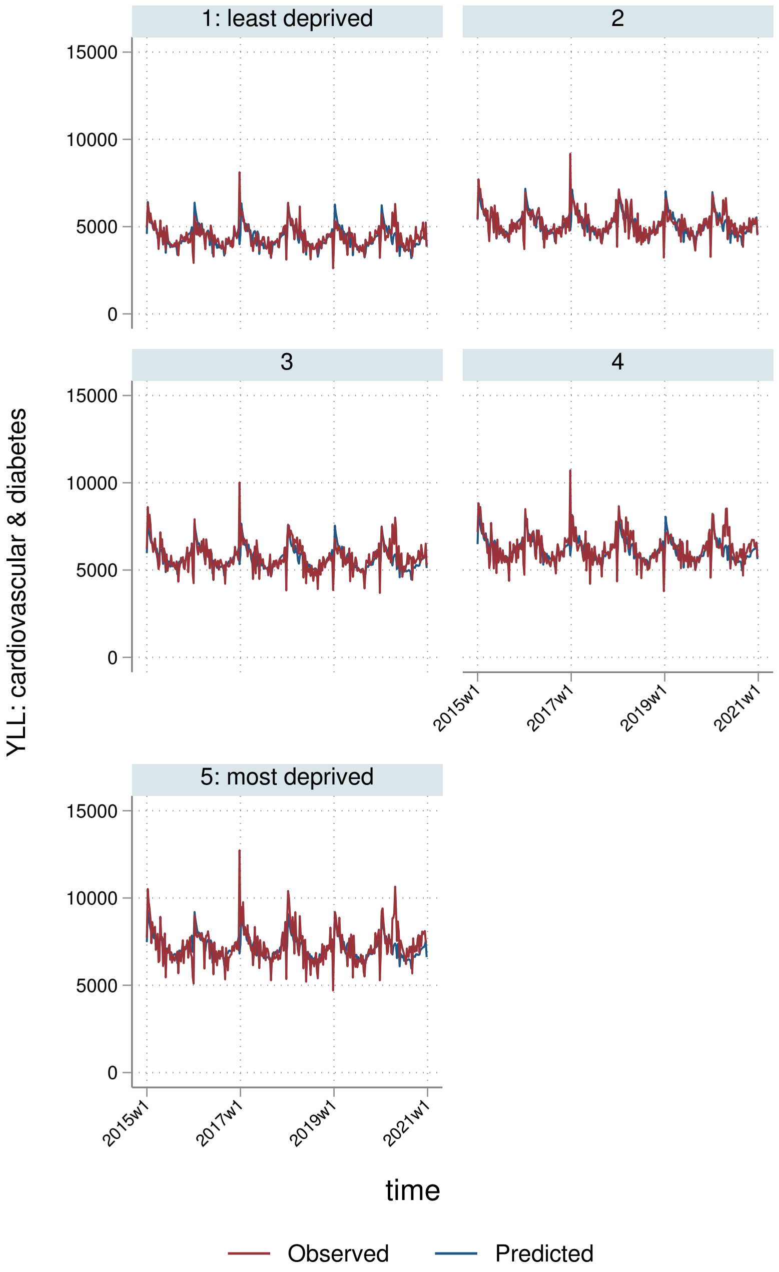

Figure 156: Observed vs Predicted Years of Life Lost trends, cardiovascular & diabetes deaths by deprivation quintile, 2019-2020

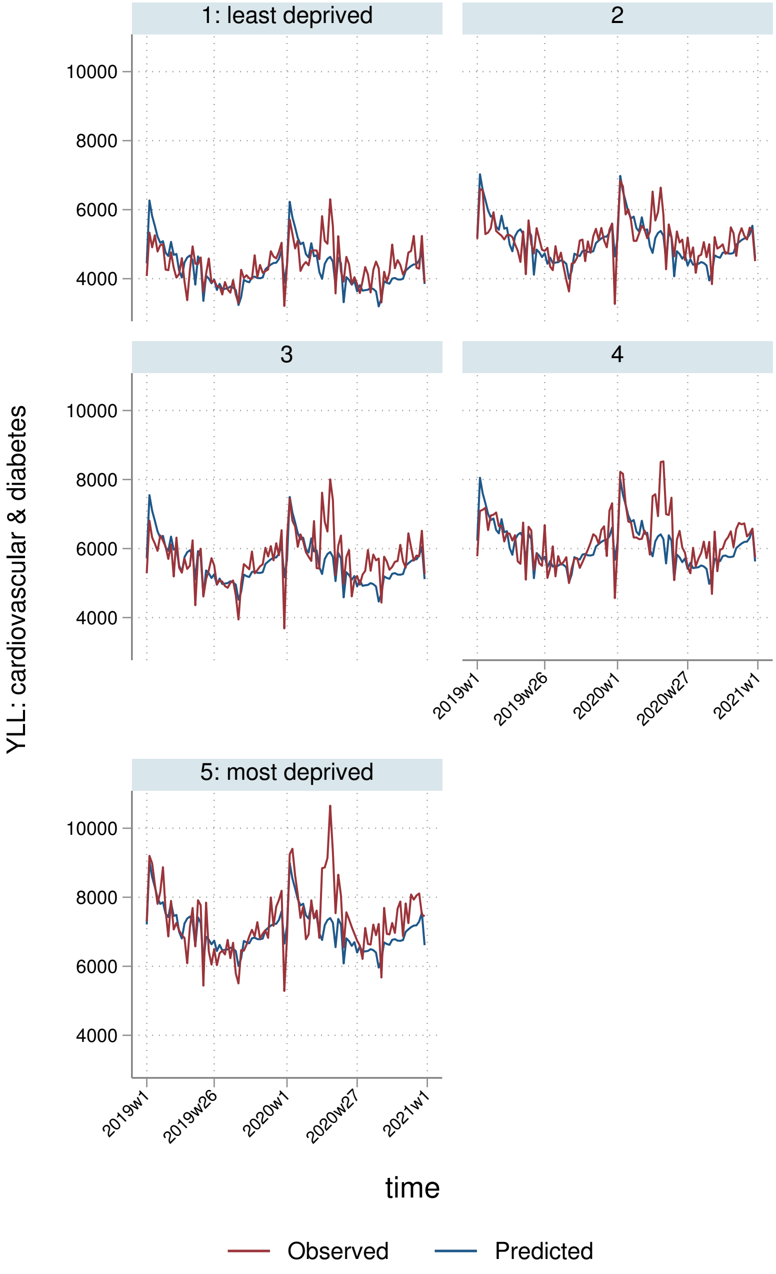

7.2.4 By Strategic Health Authority

Figure 157: Years of Life Lost trend, cardiovascular & diabetes deaths by region, 2015-2020

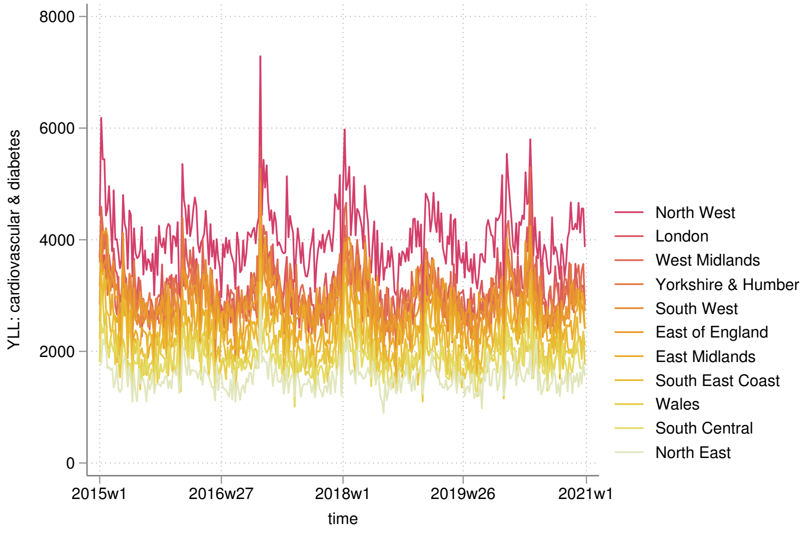

Figure 158: Years of Life Lost trend, cardiovascular & diabetes deaths by region, 2019-2020

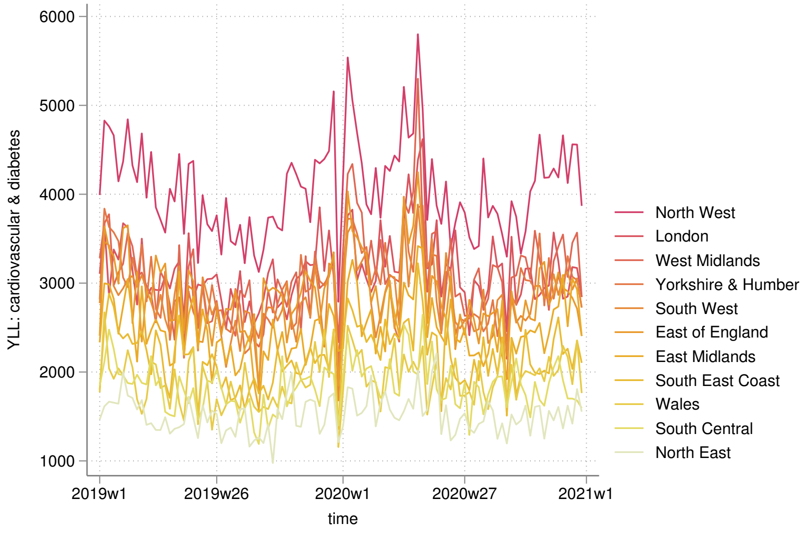

Figure 159: Excess Years of Life Lost trend, cardiovascular & diabetes deaths by region, 2015-2020

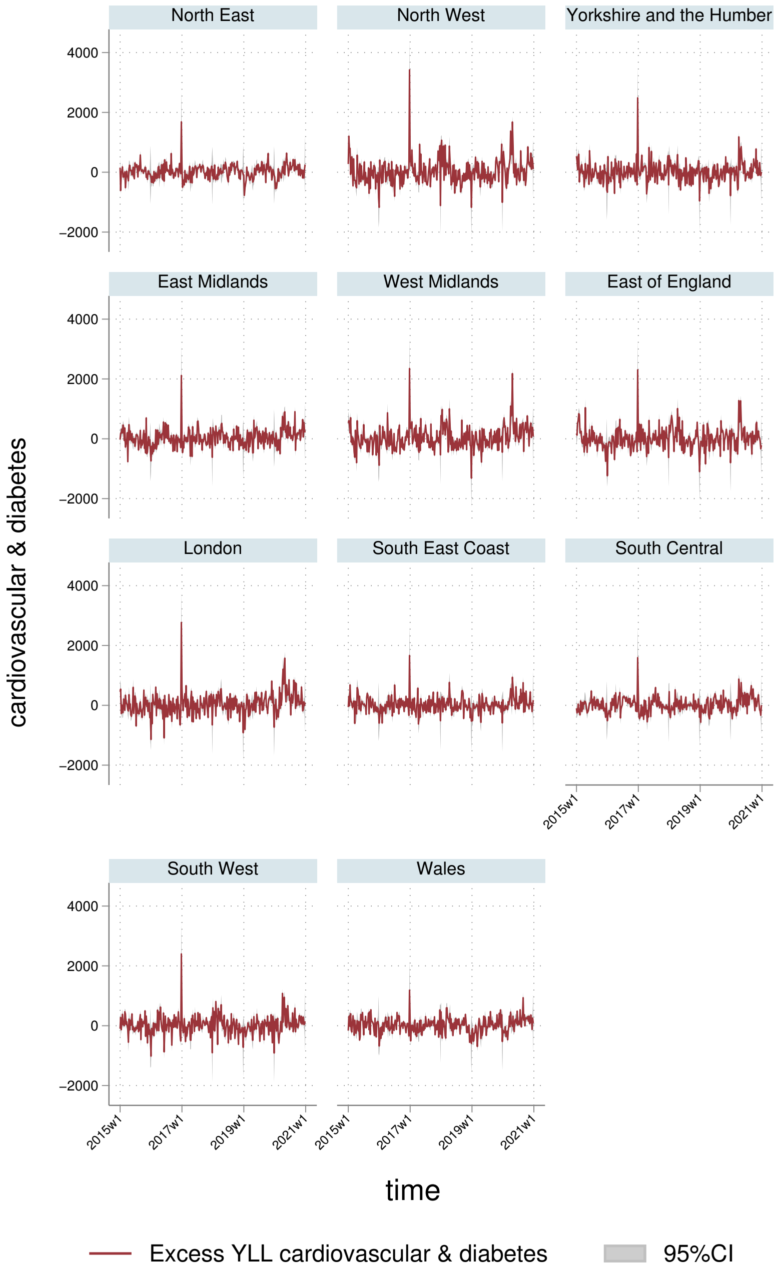

Figure 160: Excess Years of Life Lost trend, cardiovascular & diabetes deaths by region, 2019-2020

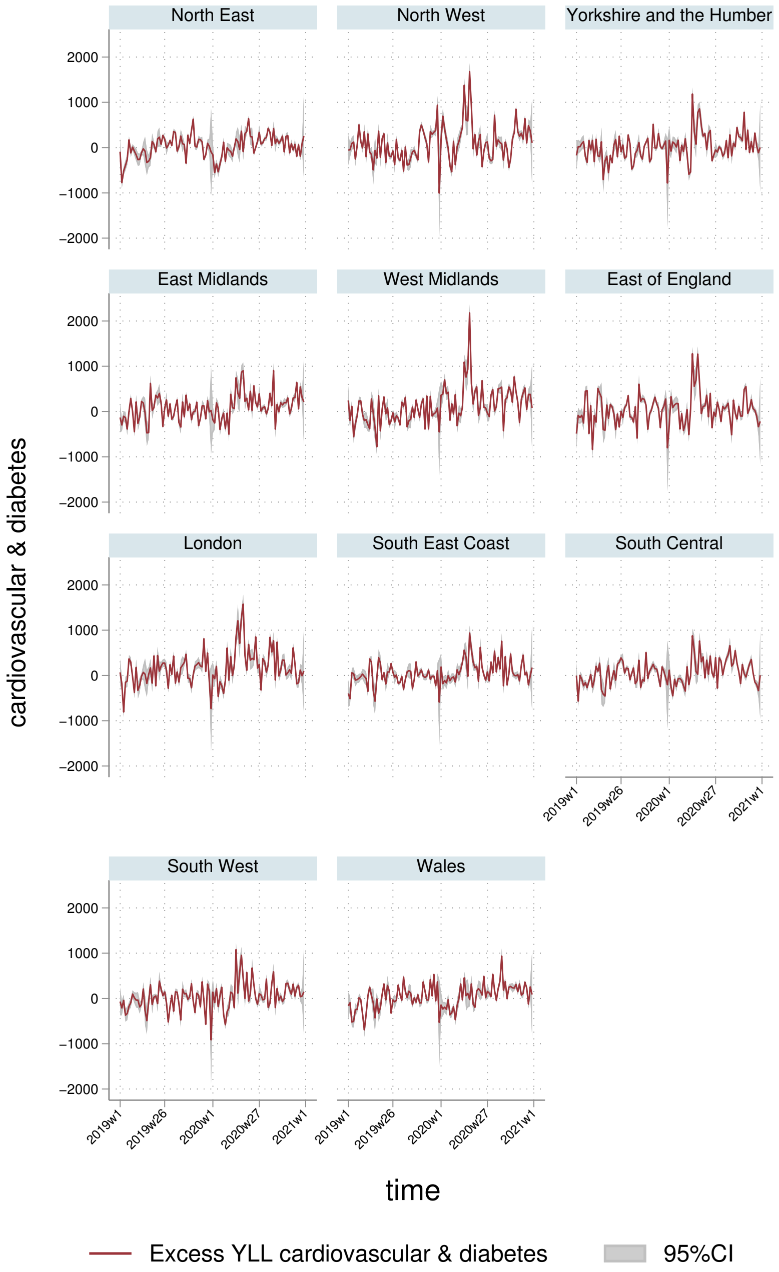

Figure 161: Observed vs Predicted Years of Life Lost trends, cardiovascular & diabetes deaths by region, 2015-2020

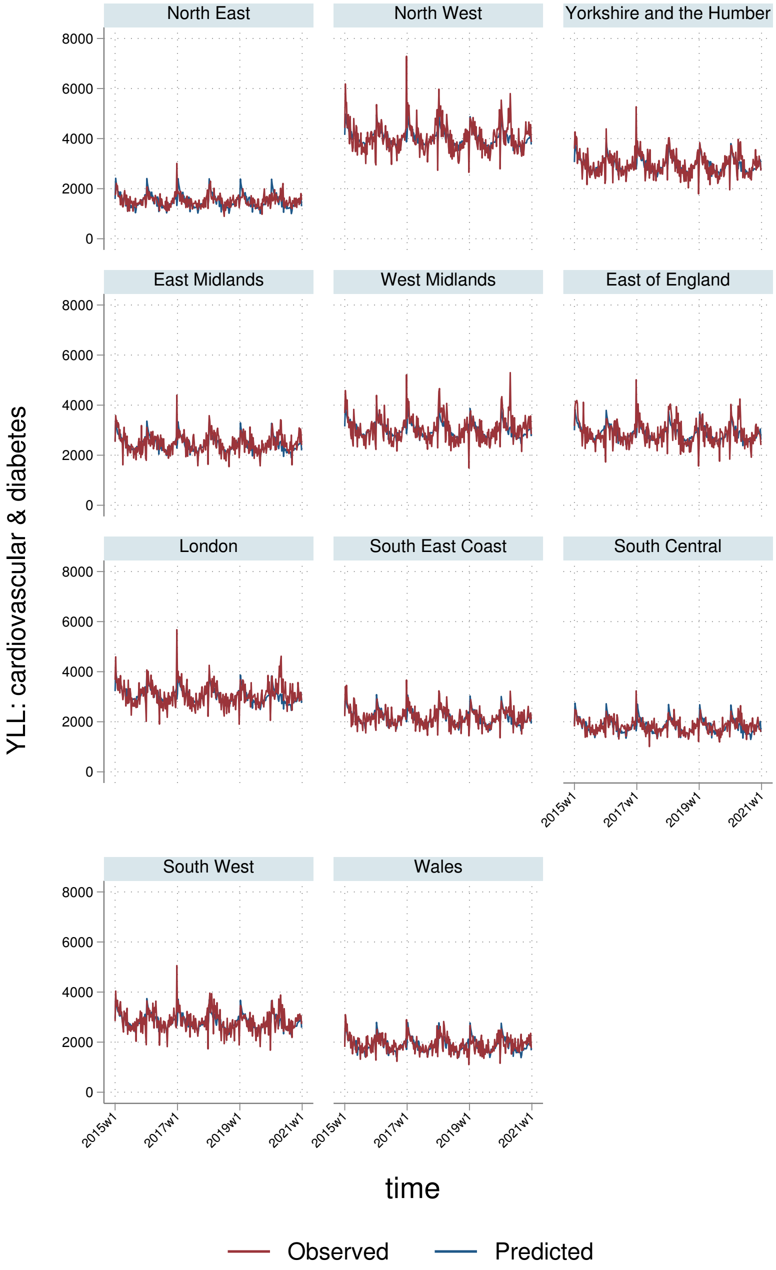

7.3 YLLs per $100,000$ population

7.3.1 England-Wales aggregate

Figure 162: Observed vs Predicted Years of Life Lost trends, cardiovascular & diabetes deaths by region, 2019-2020

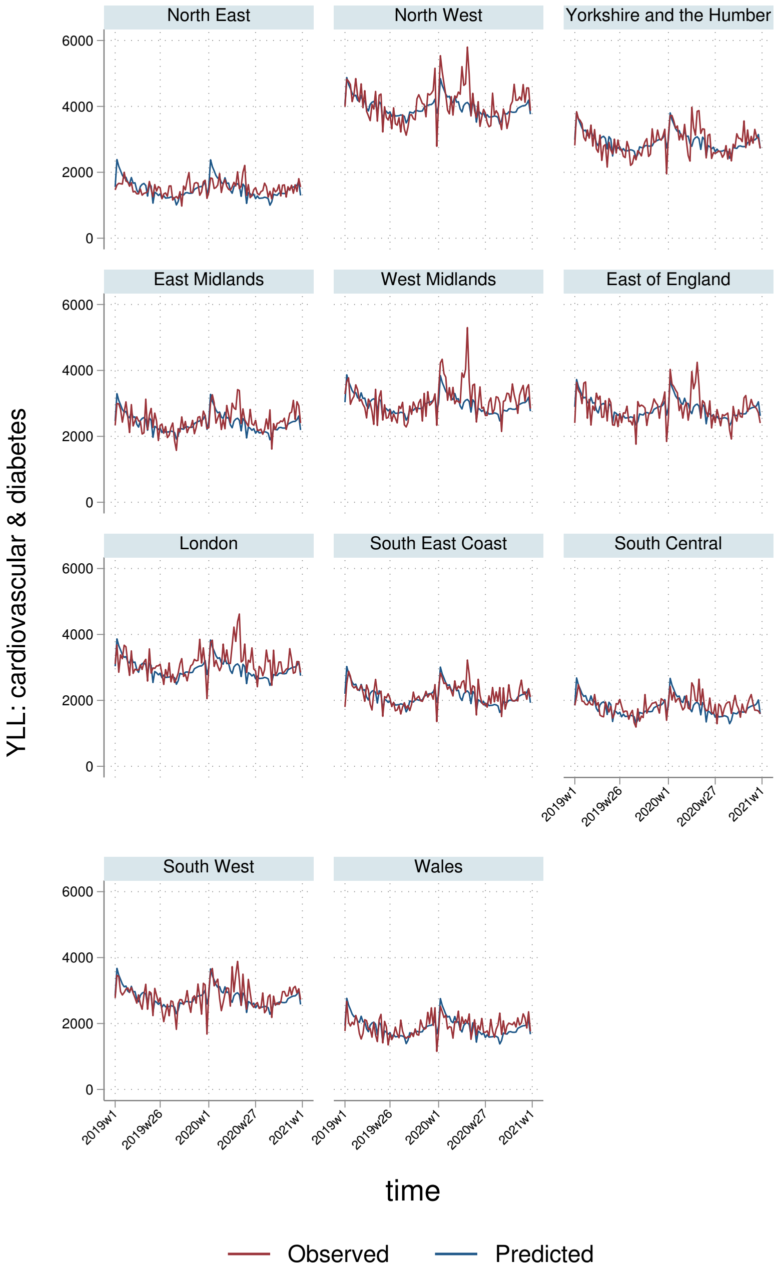

Figure 163: Years of Life Lost trend per 100,000 population, cardiovascular & diabetes deaths, 2015-2020

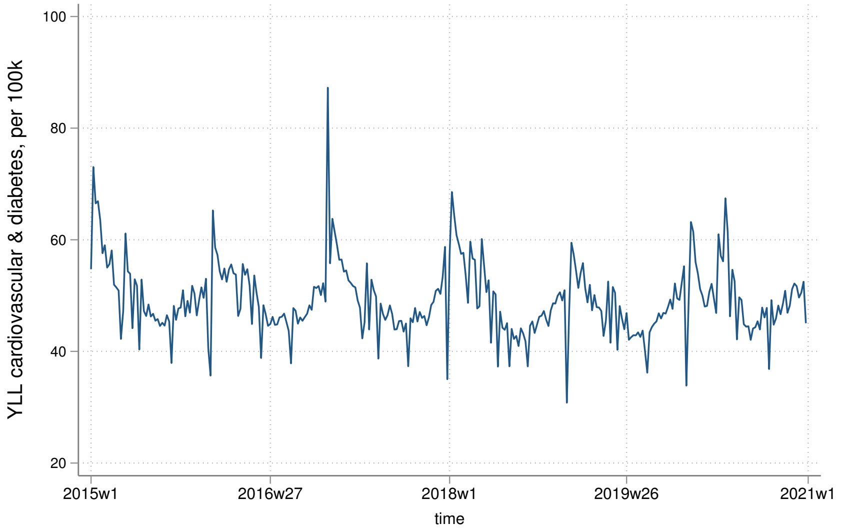

Figure 164: Years of Life Lost trend per 100,000 population, cardiovascular & diabetes deaths, 2019-2020

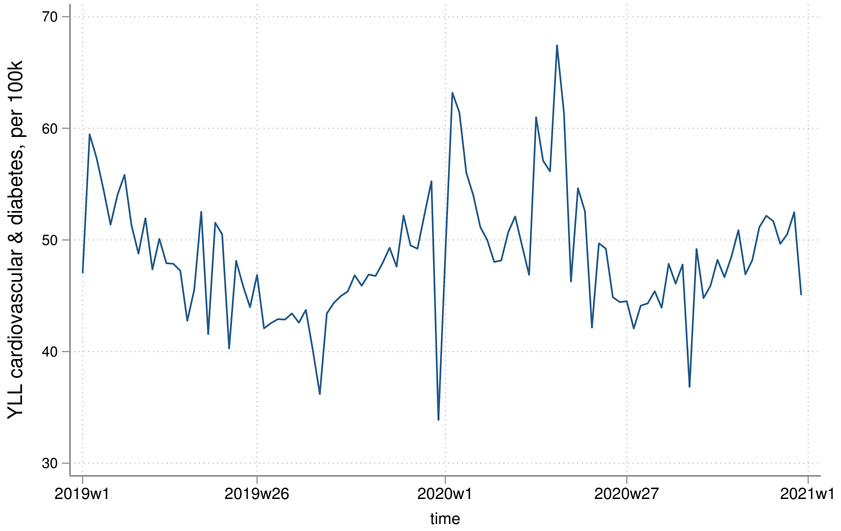

Figure 165: Excess Years of Life Lost trend per 100,000 population, cardiovascular & diabetes deaths, 2015-2020

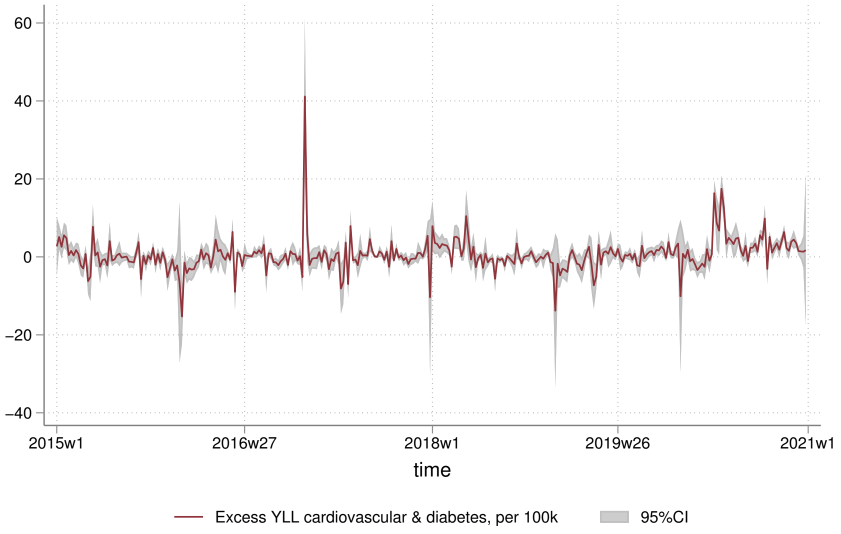

Figure 166: Excess Years of Life Lost trend per 100,000 population, cardiovascular & diabetes deaths, 2019-2020

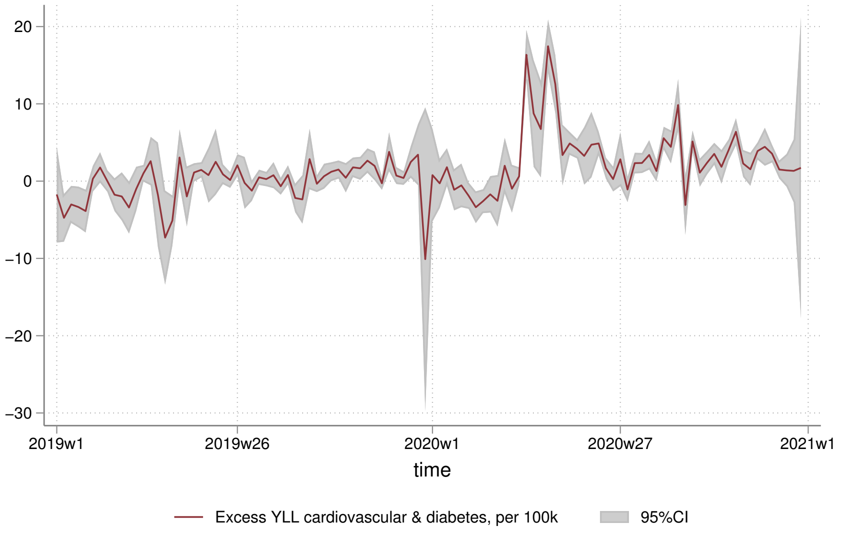

Figure 167: Observed vs Predicted Years of Life Lost trends per 100, 000 population, cardiovascular & diabetes deaths, 2015-2020

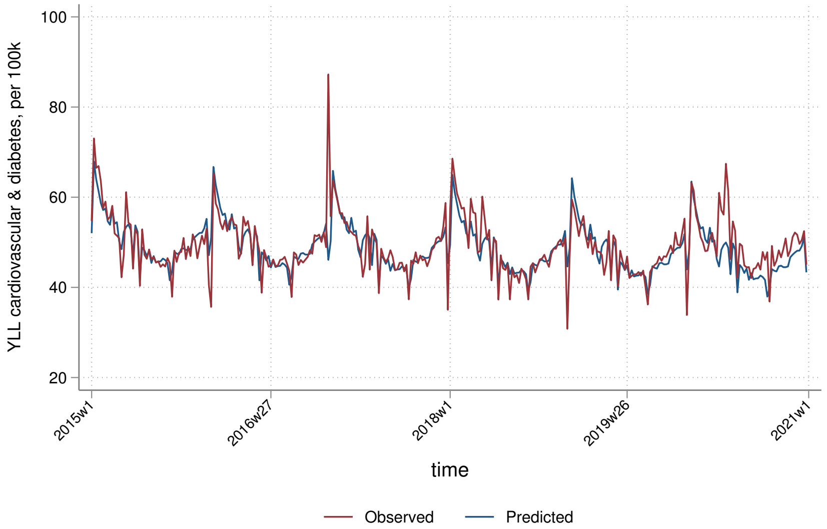

Figure 168: Observed vs Predicted Years of Life Lost trends per 100, 000 population, cardiovascular & diabetes deaths, 2019-2020

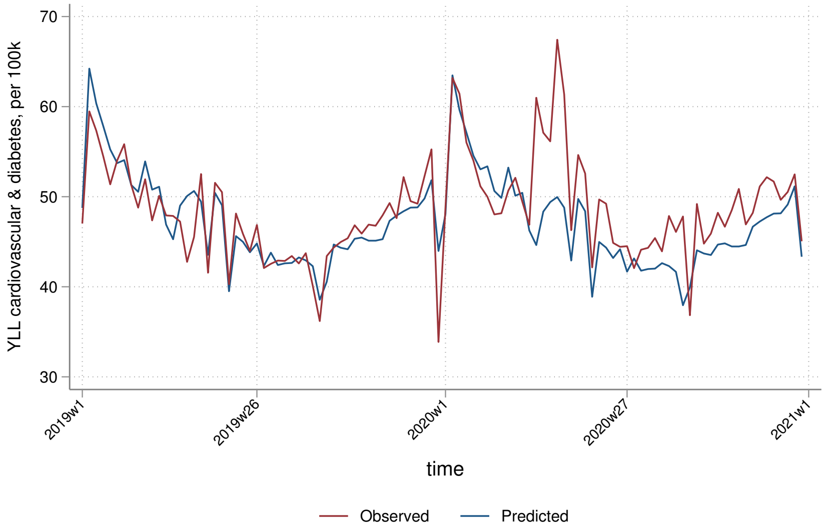

7.3.2 By sex

Figure 169: Years of Life Lost trend per 100,000 population, cardiovascular & diabetes deaths by sex, 2015-2020

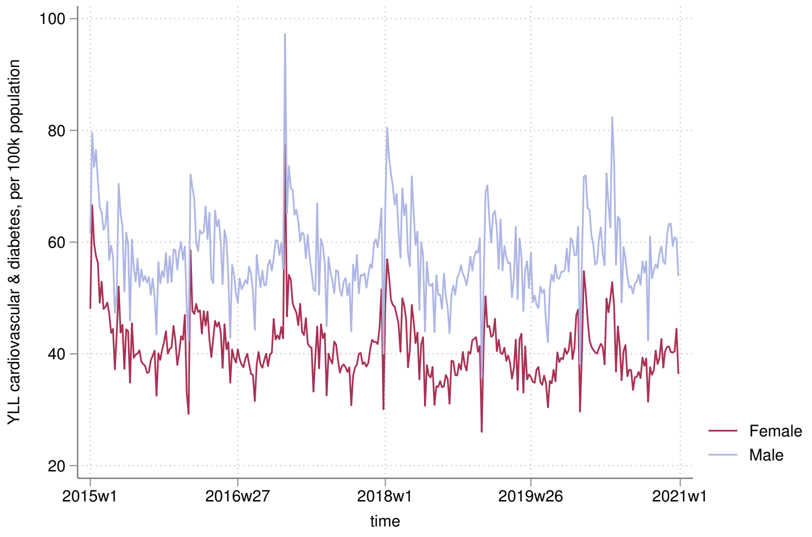

Figure 170: Years of Life Lost trend per 100,000 population, cardiovascular & diabetes deaths by sex, 2019-2020

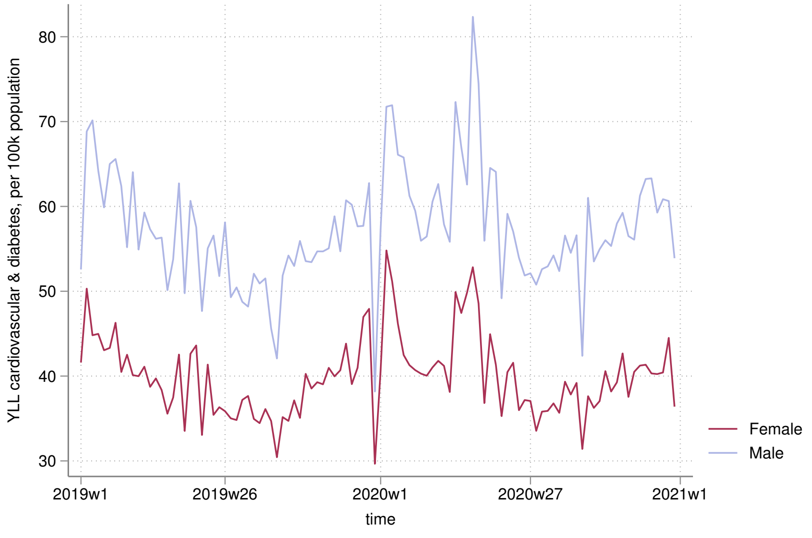

Figure 171: Excess Years of Life Lost trend per 100, 000 population, cardiovascular & diabetes deaths by sex, 2015-2020

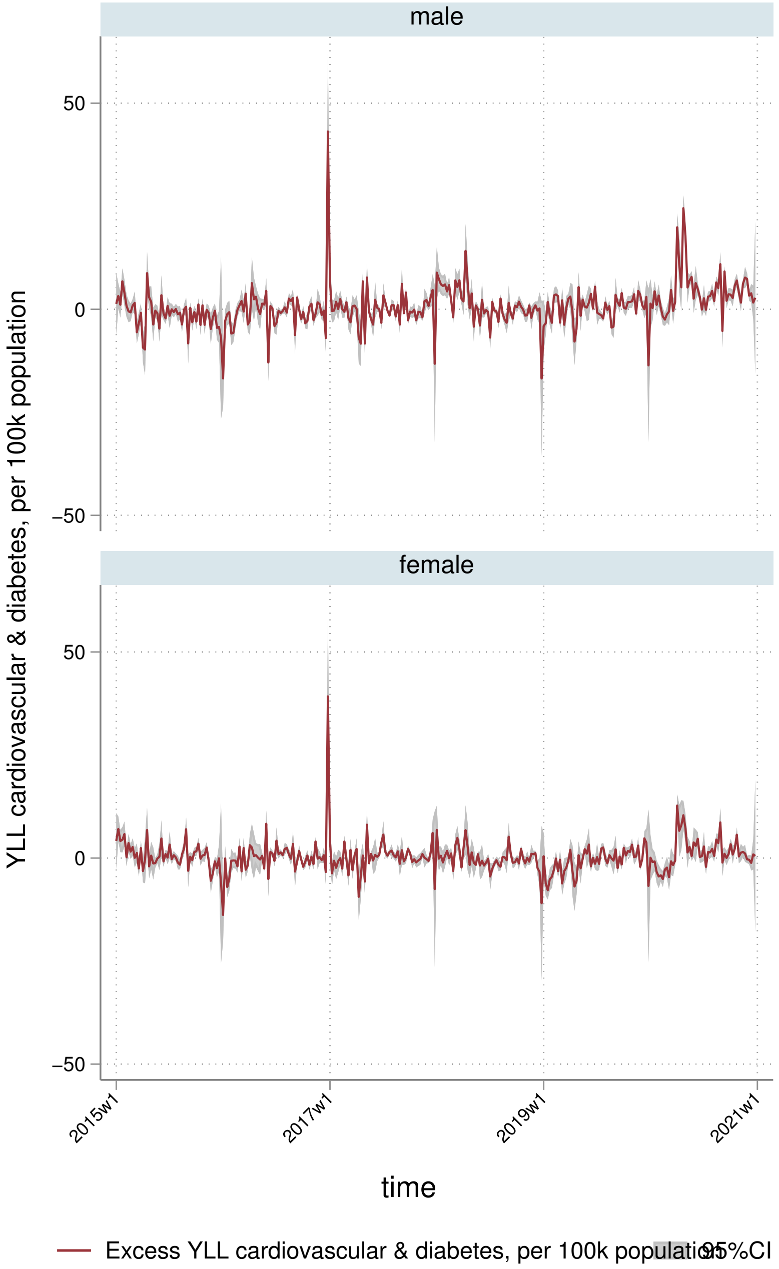

Figure 172: Excess Years of Life Lost trend per 100, 000 population, cardiovascular & diabetes deaths by sex, 2015-2020

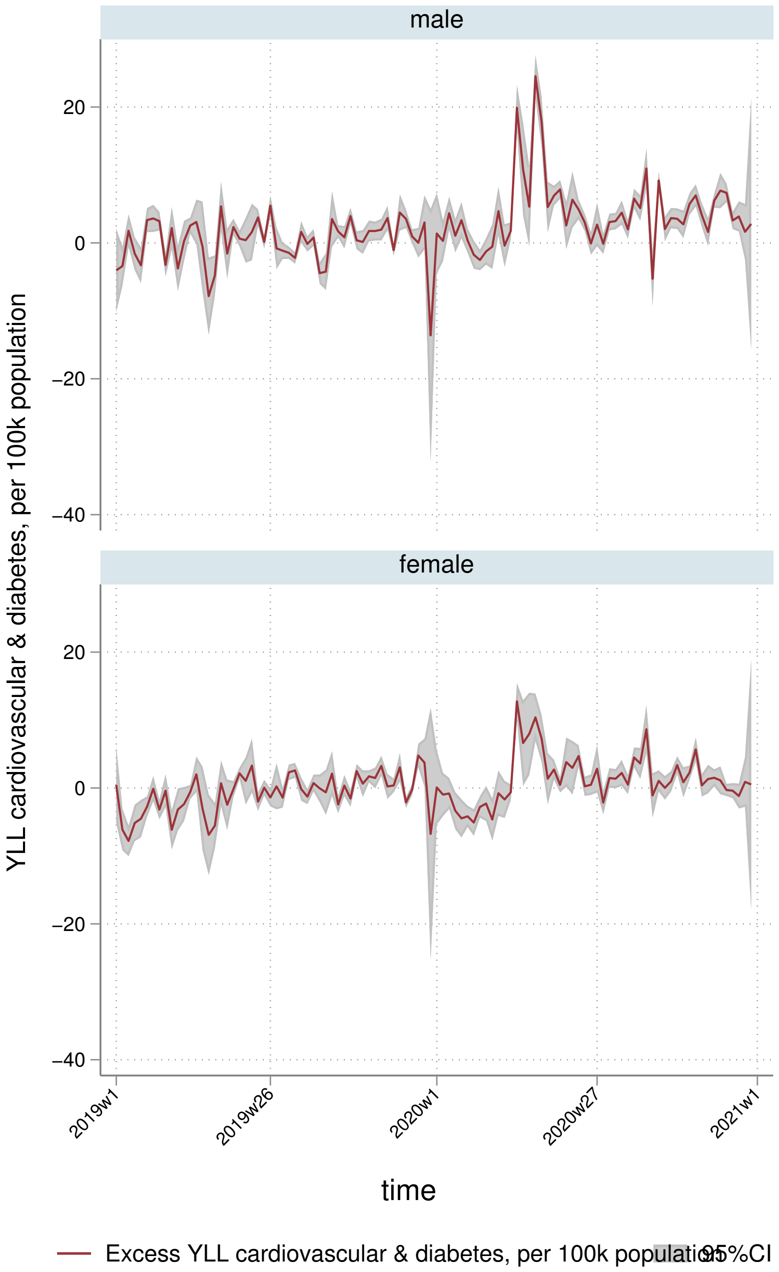

Figure 173: Observed vs Predicted Years of Life Lost trends per 100,000 population, cardiovascular & diabetes deaths by sex, 2015-2020

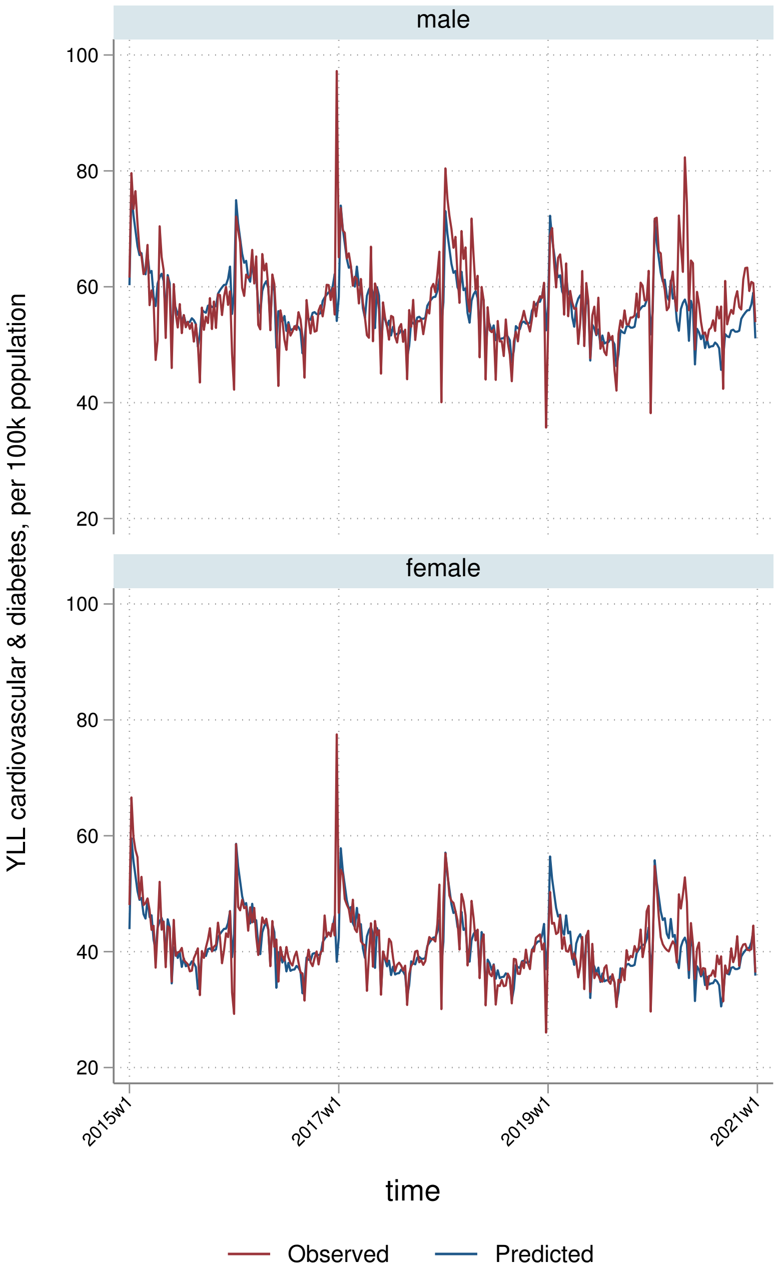

Figure 174: Observed vs Predicted Years of Life Lost trends per 100, 000 population, cardiovascular & diabetes deaths by sex, 2015-2020

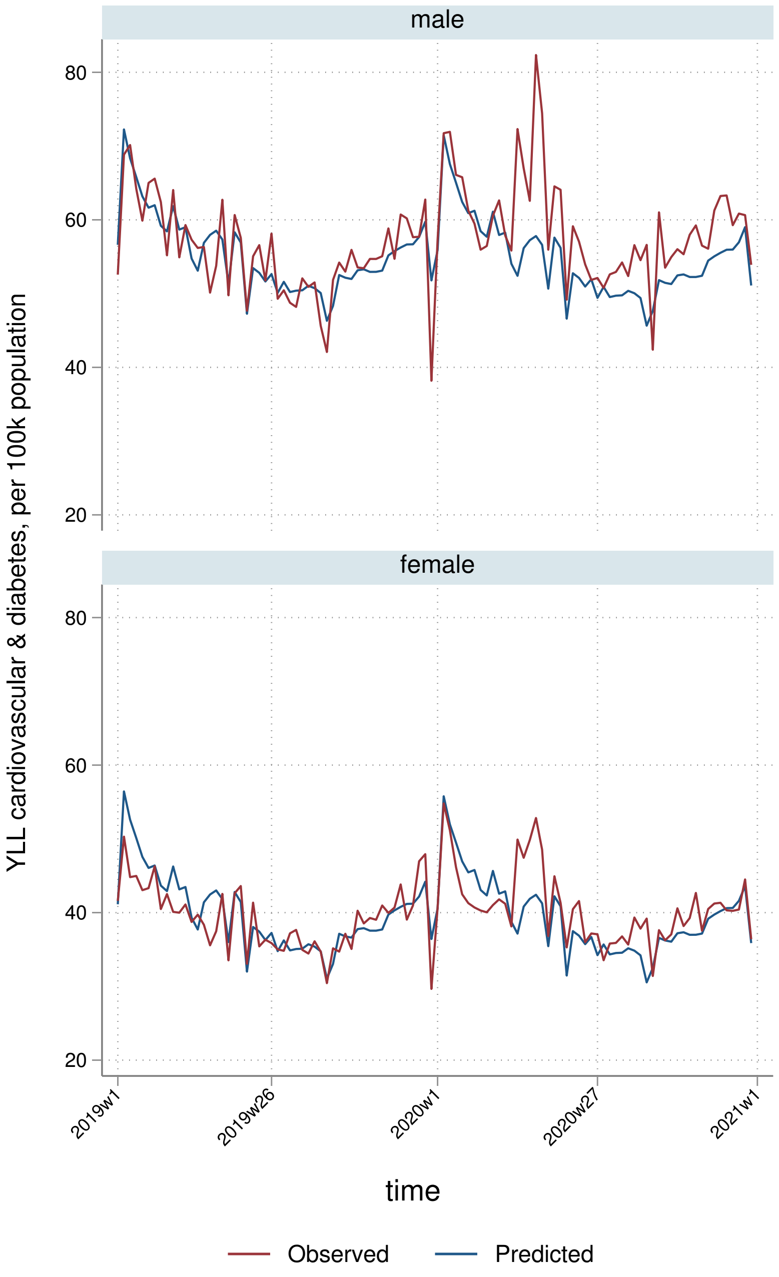

7.3.3 By deprivation quintile

Figure 175: Years of Life Lost trend per 100,000 population, cardiovascular & diabetes deaths by deprivation quintile, 2015-2020

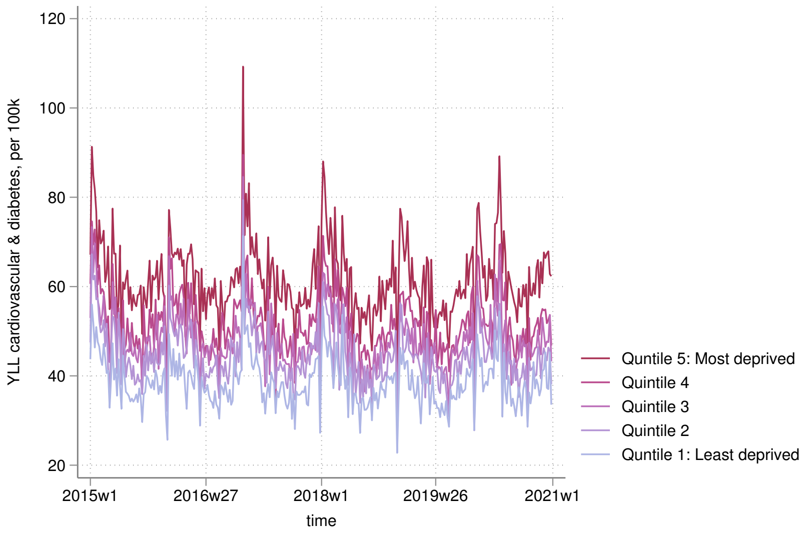

Figure 176: Years of Life Lost trend per 100,000 population, cardiovascular & diabetes deaths by deprivation quintile, 2019-2020

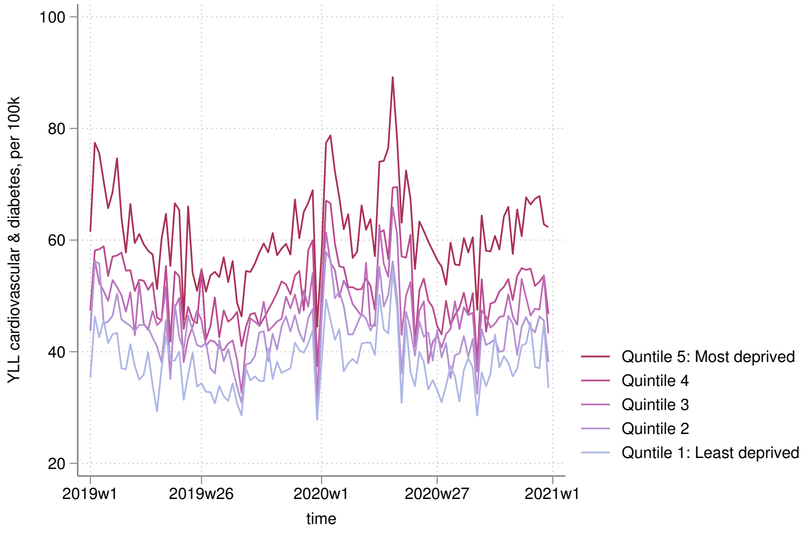

Figure 177: Excess Years of Life Lost trend per 100, 000 population, cardiovascular & diabetes deaths by deprivation quintile, 2015-2020

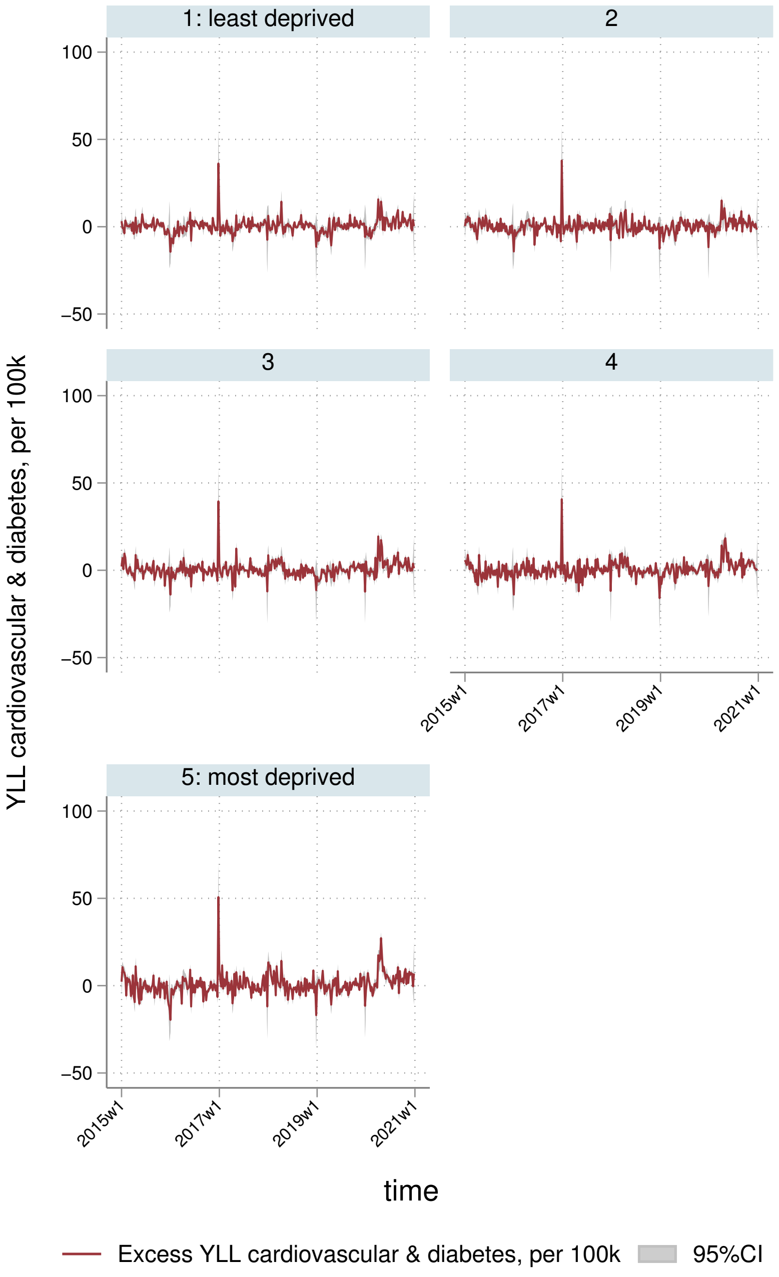

Figure 178: Excess Years of Life Lost trend per 100, 000 population, cardiovascular & diabetes deaths by deprivation quintile, 2019-2020

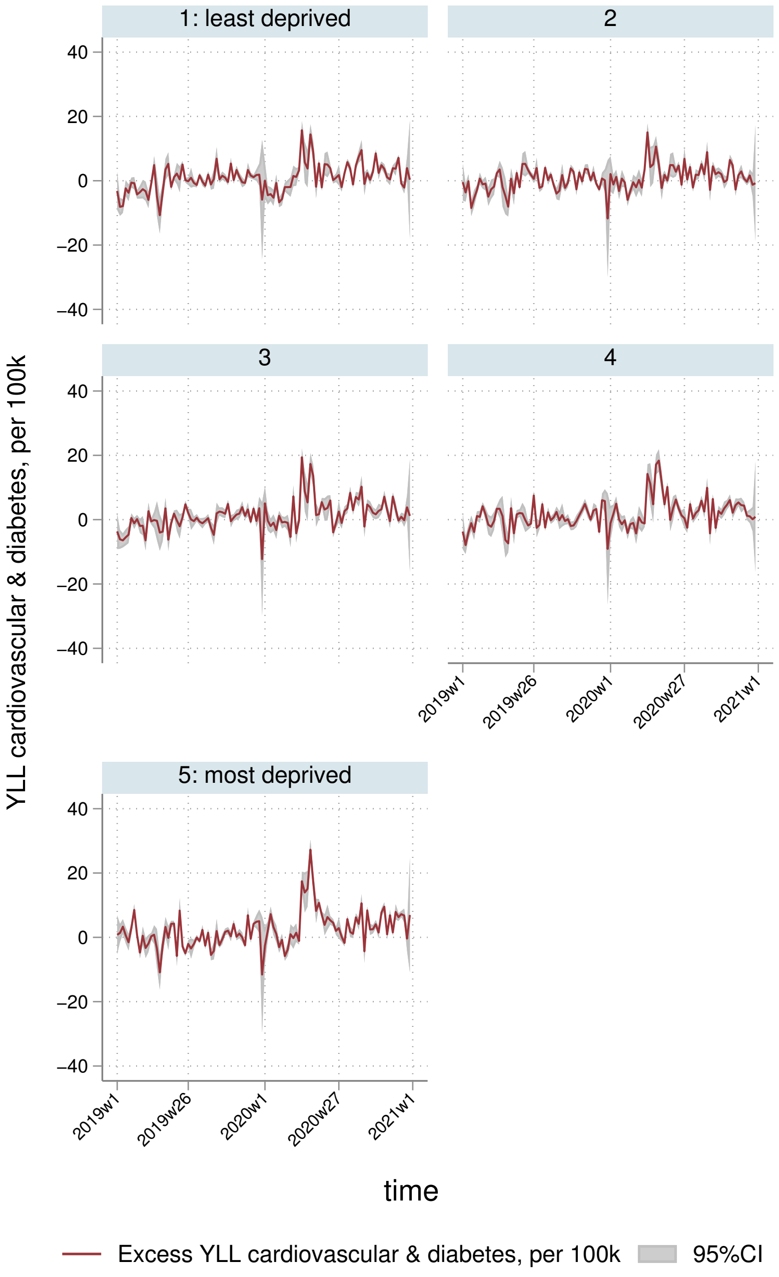

Figure 179: Observed vs Predicted Years of Life Lost trends per 100,000 population, cardiovascular & diabetes deaths by deprivation quintile, 2015-2020

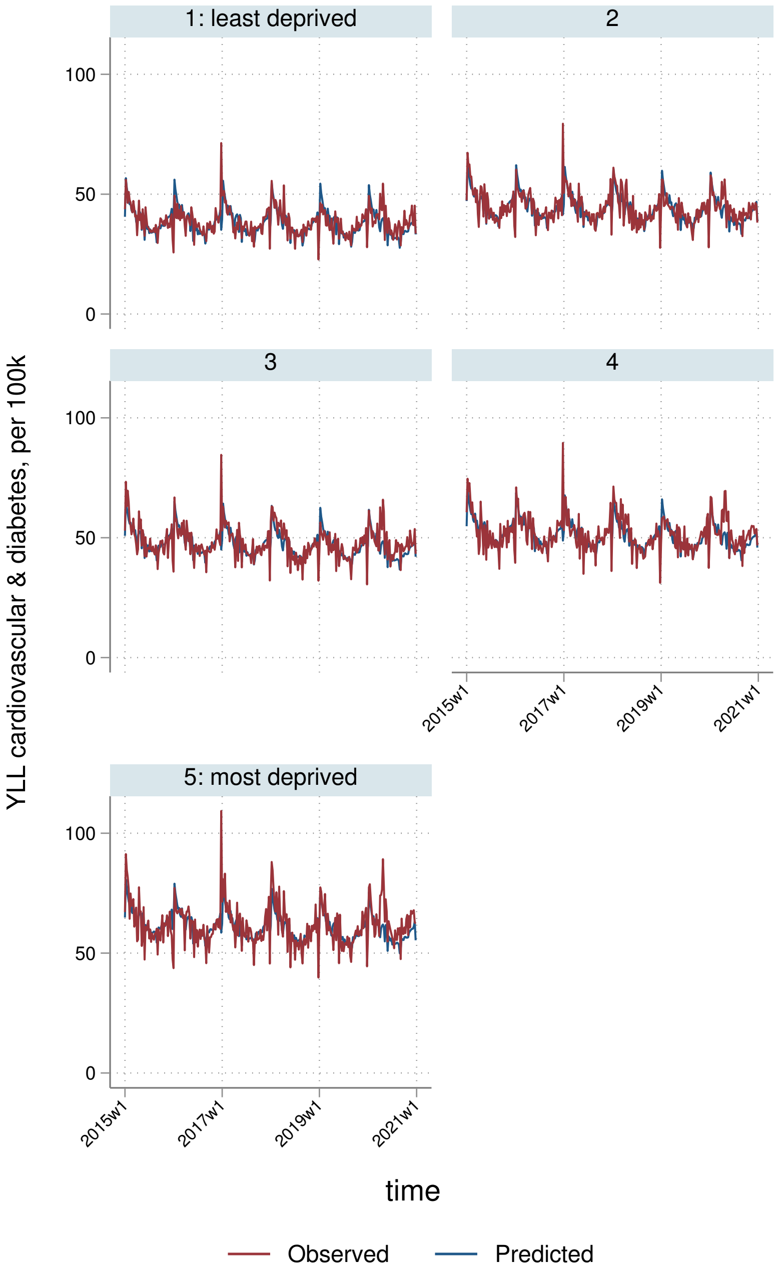

Figure 180: Observed vs Predicted Years of Life Lost trends per 100,000 population, cardiovascular & diabetes deaths by deprivation quintile, 2019-2020

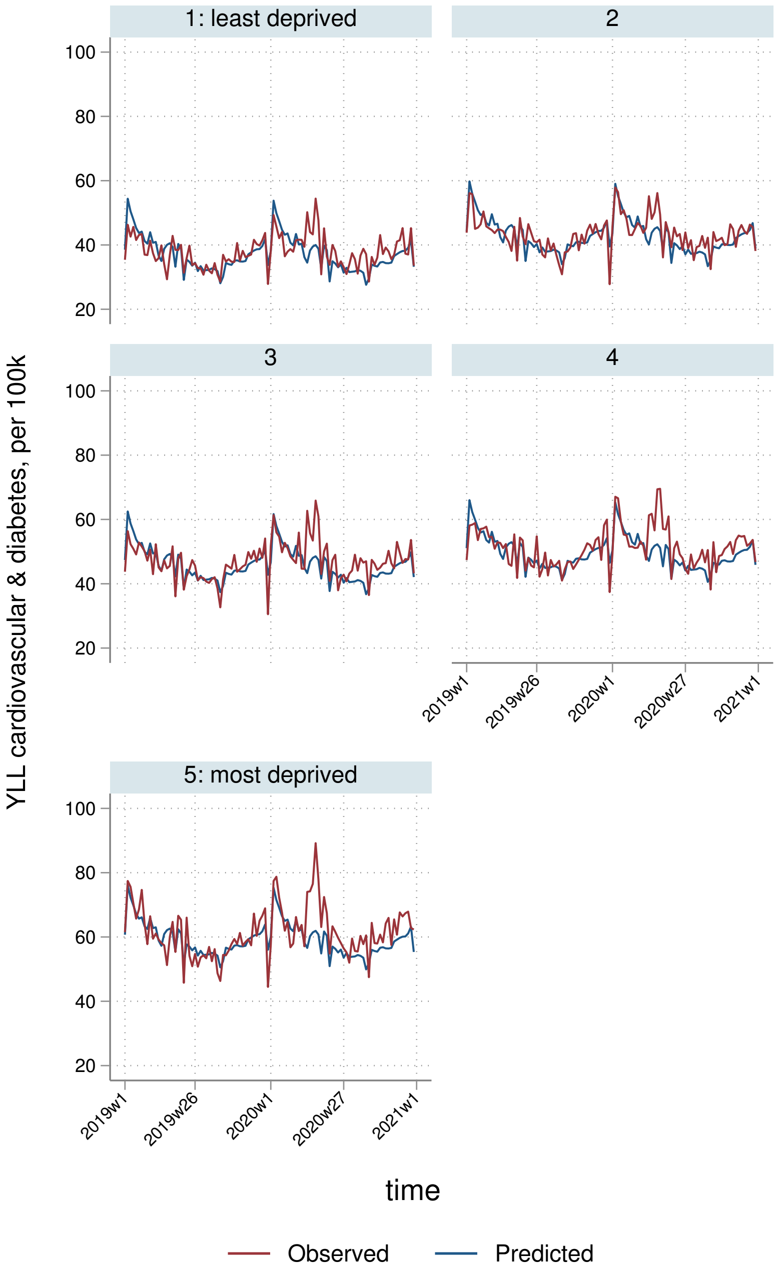

7.3.4 By Strategic Health Authority

Figure 181: Years of Life Lost trend per 100,000 population, cardiovascular & diabetes deaths by region, 2015-2020

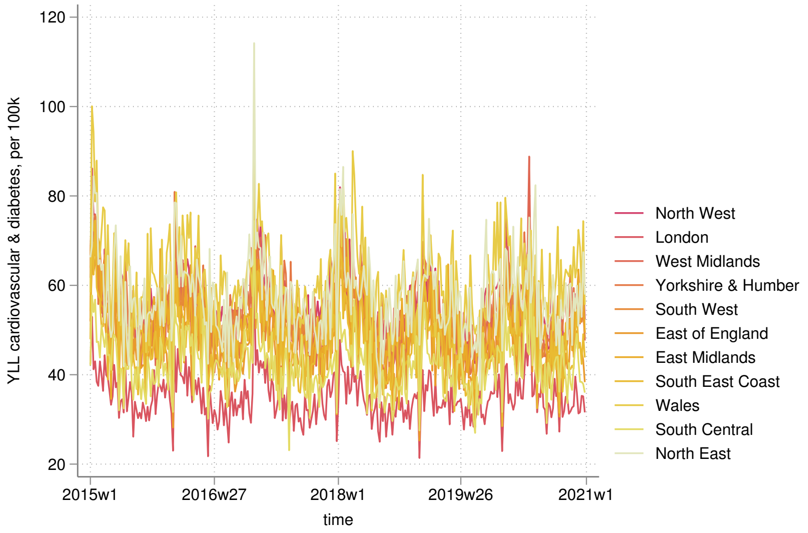

Figure 182: Years of Life Lost trend per 100,000 population, cardiovascular & diabetes deaths by region, 2019-2020

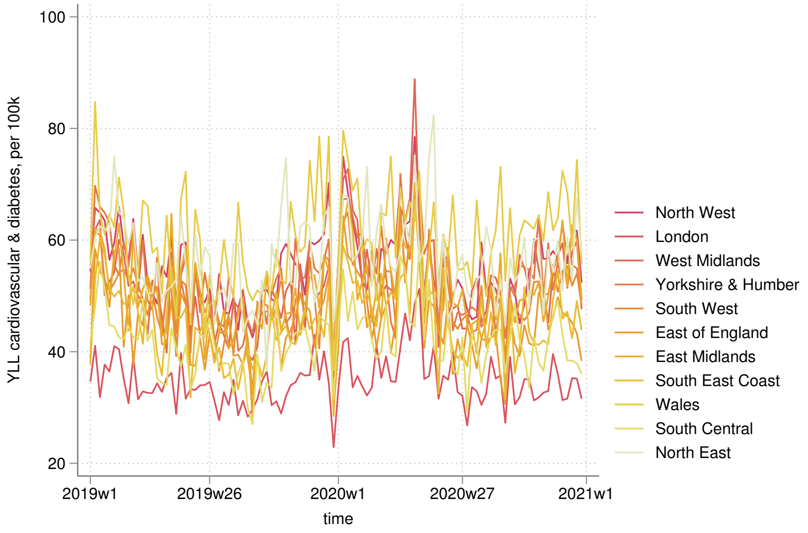

Figure 183: Excess Years of Life Lost trend per 100,000 population, cardiovascular & diabetes deaths by region, 2015-2020

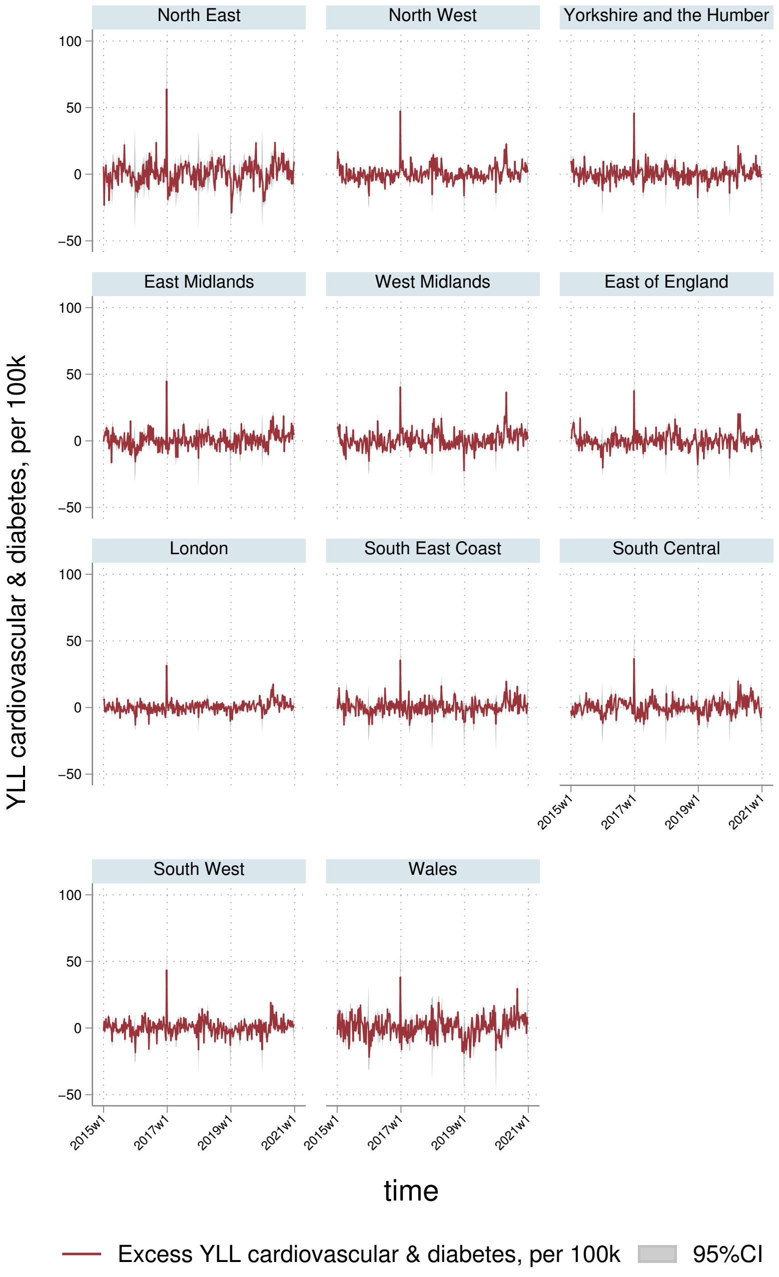

Figure 184: Excess Years of Life Lost trend per 100, 000 population, cardiovascular & diabetes deaths by region, 2019-2020

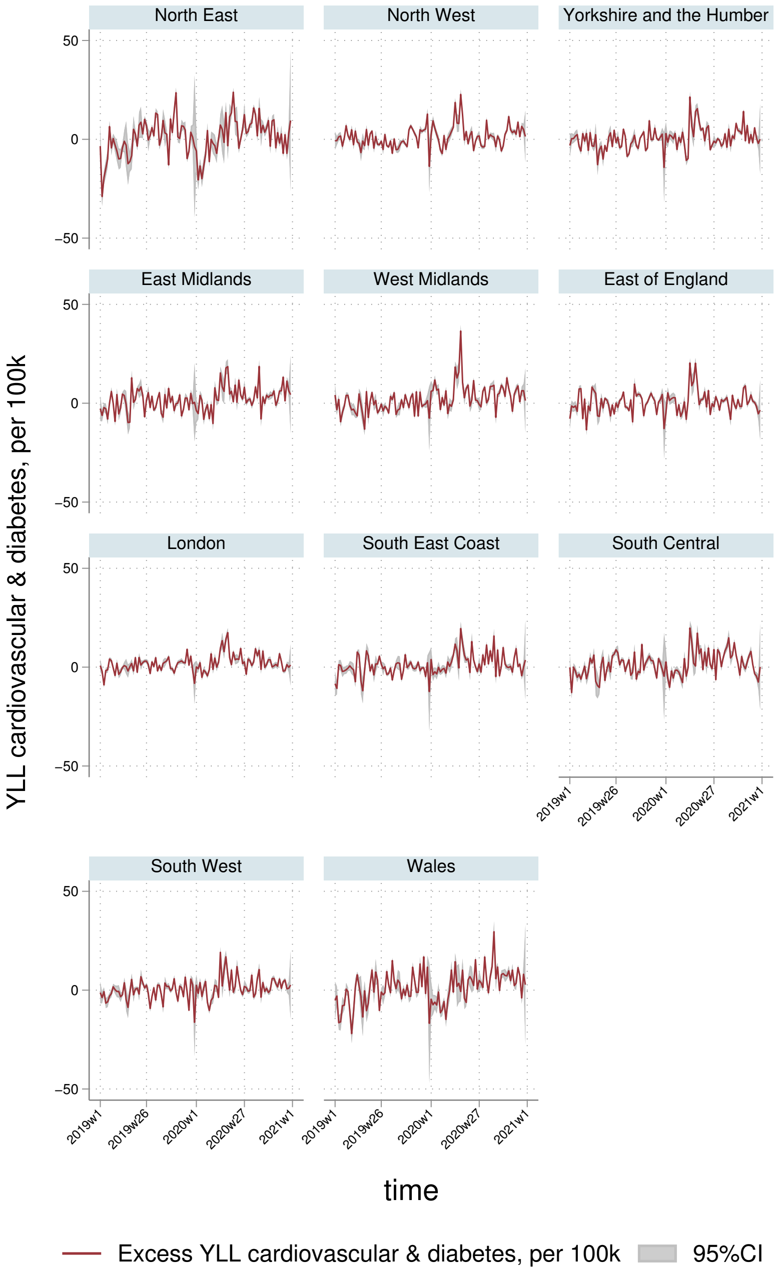

Figure 185: Observed vs Predicted Years of Life Lost trends per 100,000 population, cardiovascular & diabetes deaths by region, 2015-2020

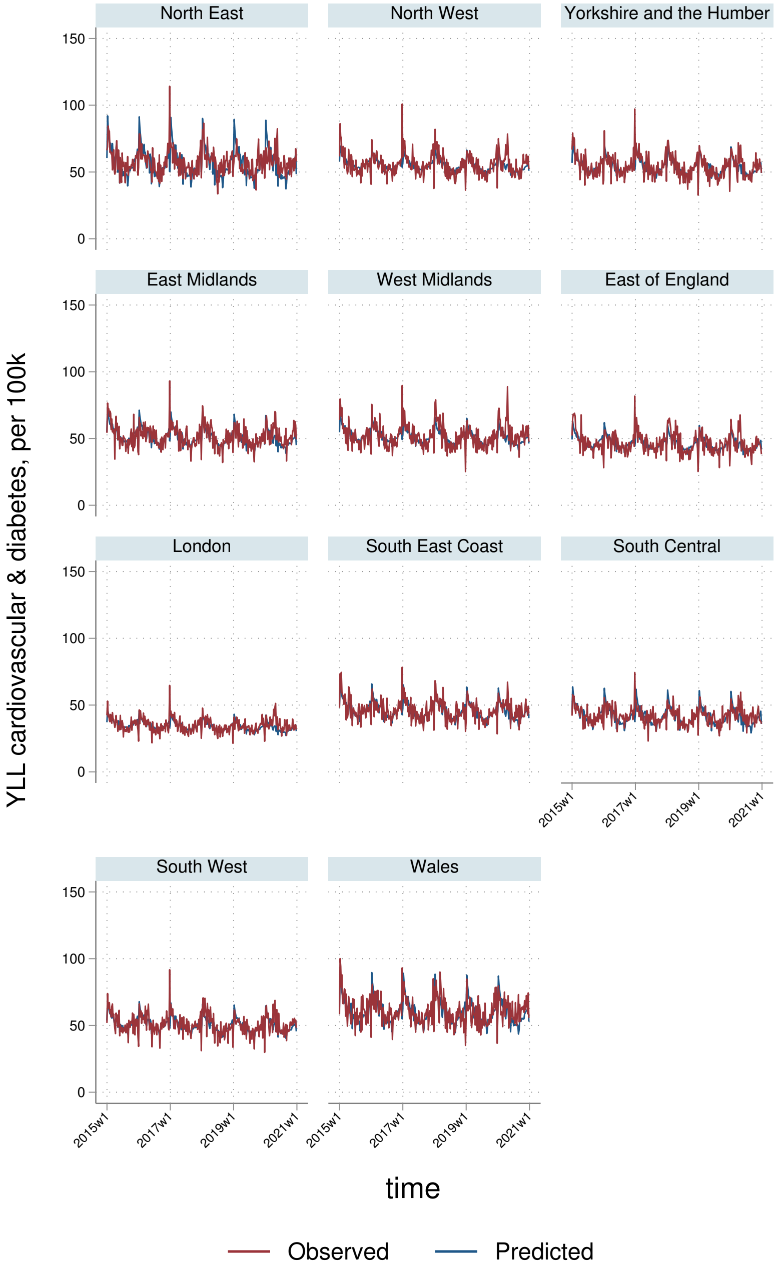

Figure 186: Observed vs Predicted Years of Life Lost trends per 100, 000 population, cardiovascular & diabetes deaths by region, 2019-2020

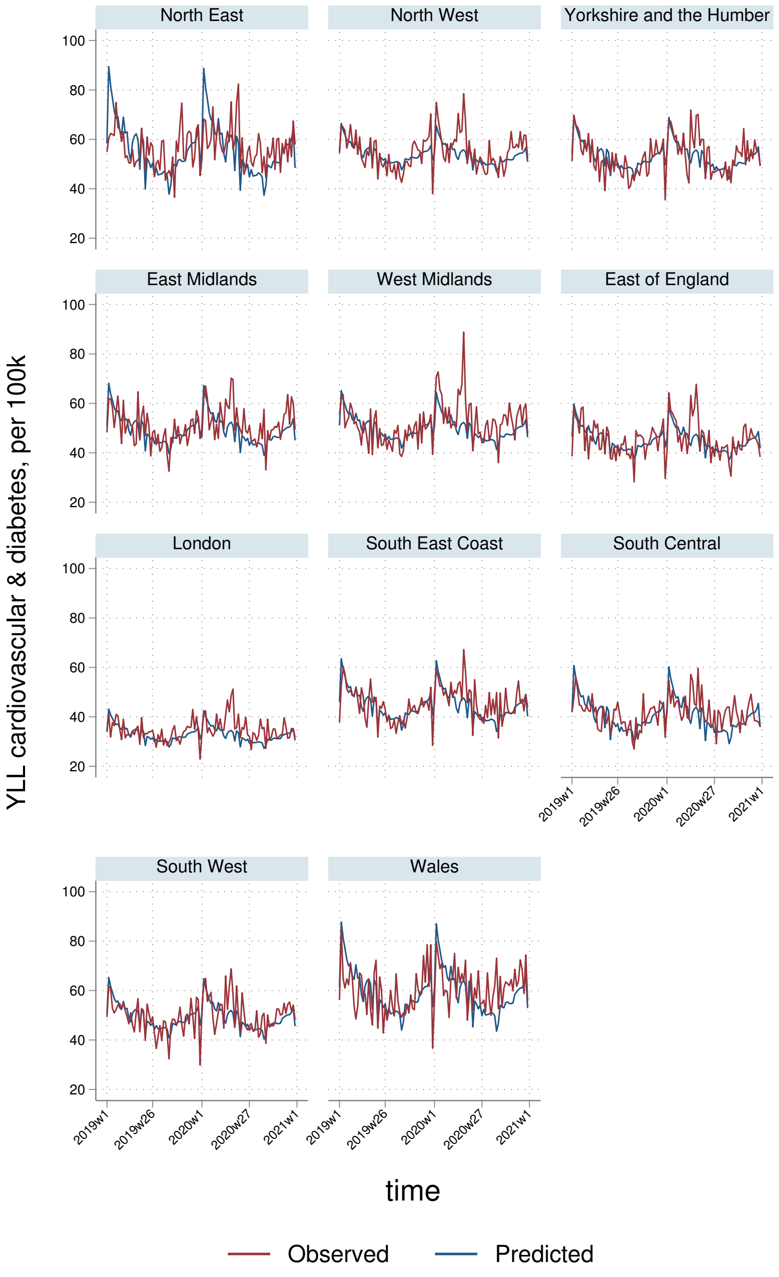

8 Cancer

8.1 AASMRs

Figure 187: Age-standardised mortality trend, cancer deaths, 2015-2020

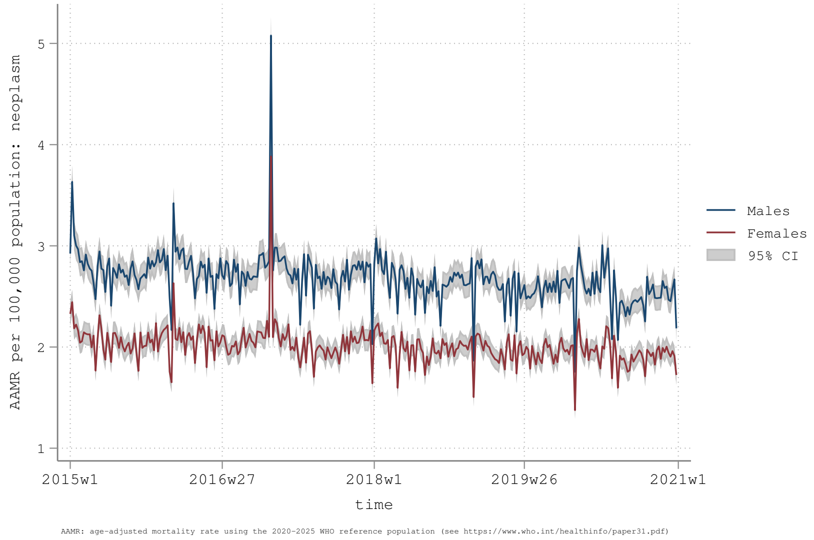

Figure 188: Age-standardised mortality trend, cancer deaths, 2019-2020

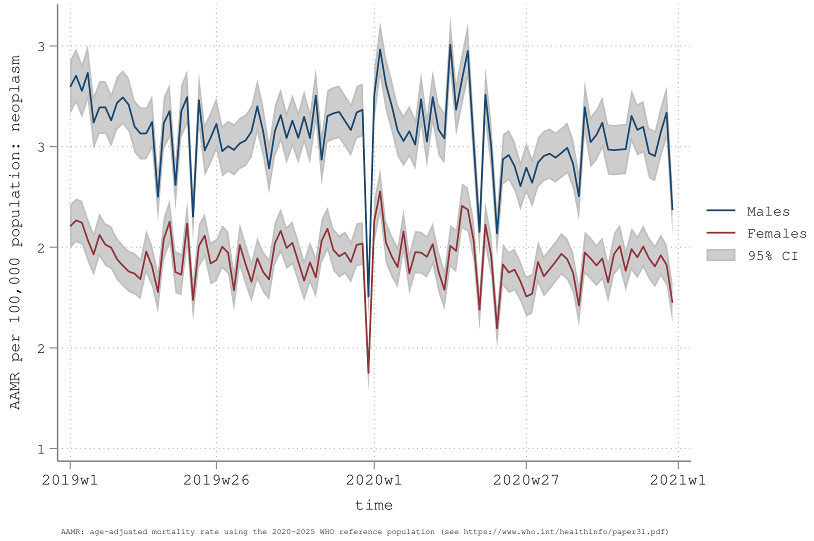

Figure 189: Age-standardised mortality trend, cancer deaths by deprivation quintile, 2015-2020

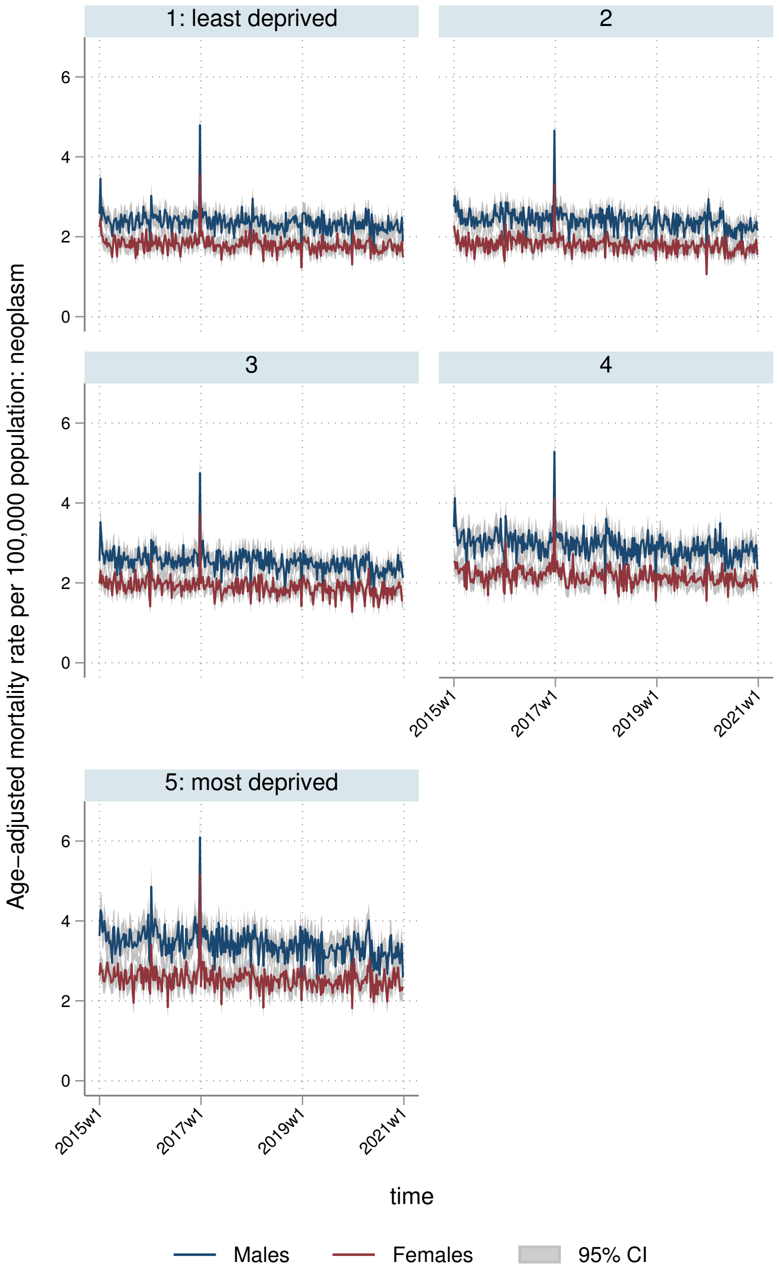

Figure 190: Age-standardised mortality trend, cancer deaths by deprivation quintile, 2019-2020

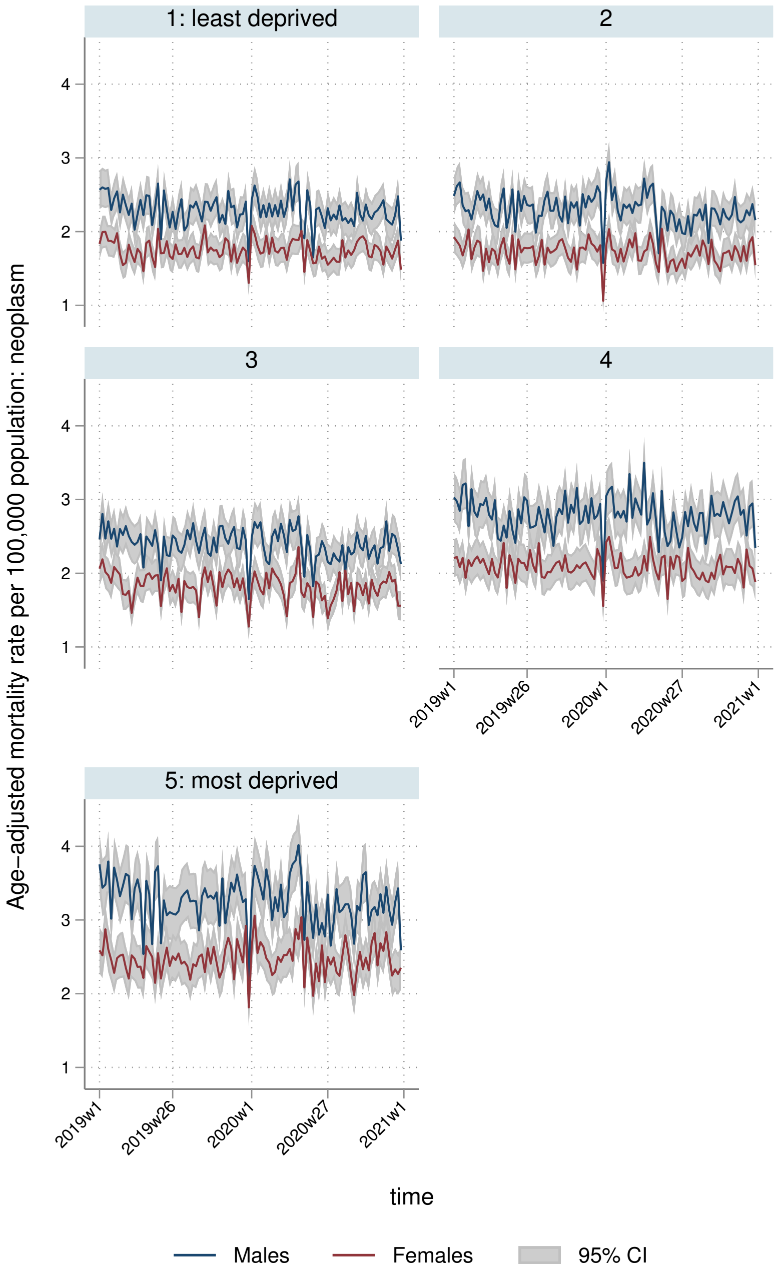

Figure 191: Age-standardised mortality trend, cancer deaths by region, 2015-2020

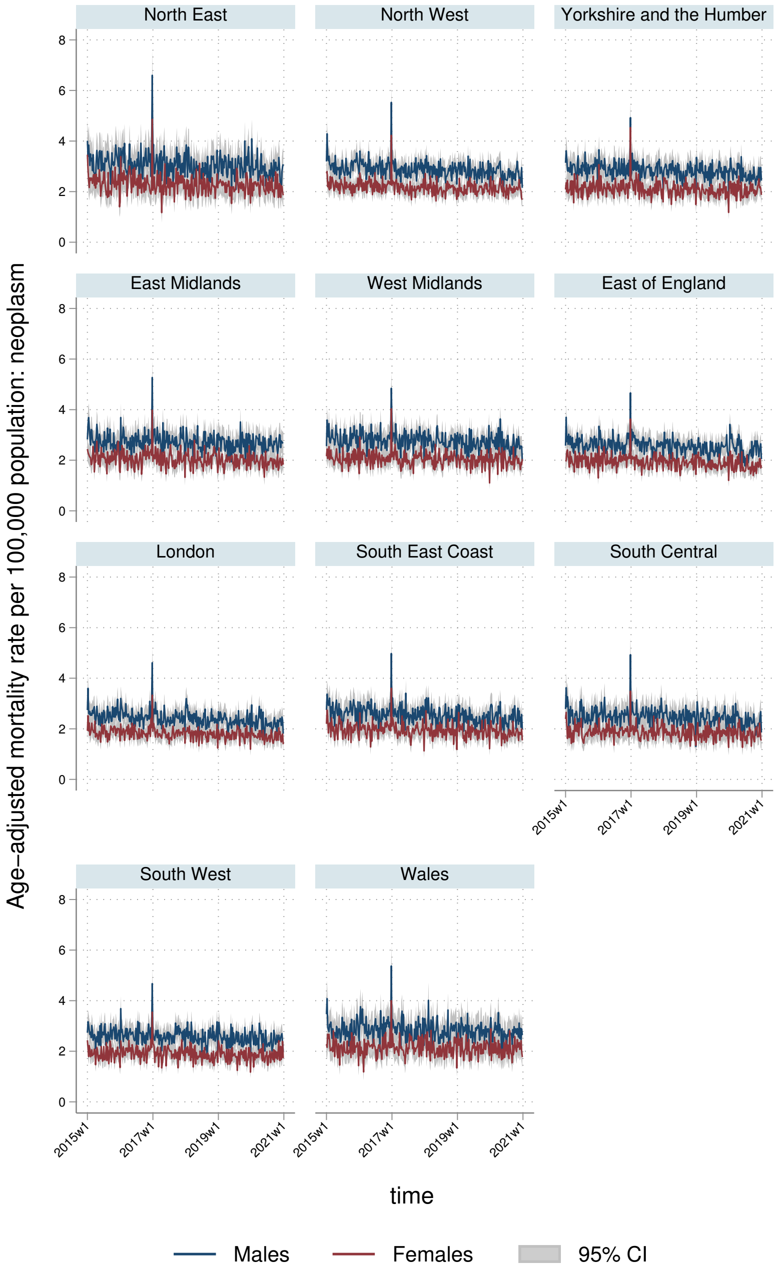

8.2 YLLs

8.2.1 England-Wales aggregate

Figure 192: Age-standardised mortality trend, cancer deaths by region, 2019-2020

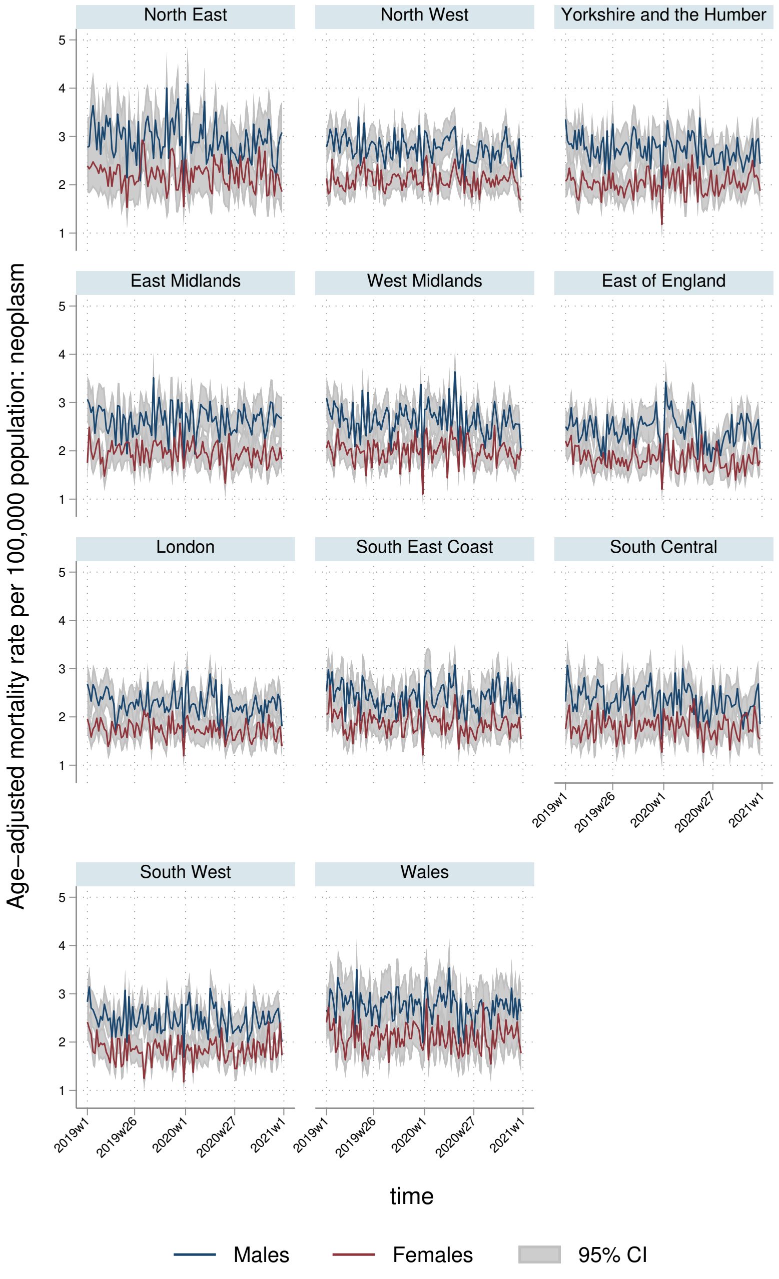

Figure 193: Years of Life Lost trend, cancer deaths, 2015-2020

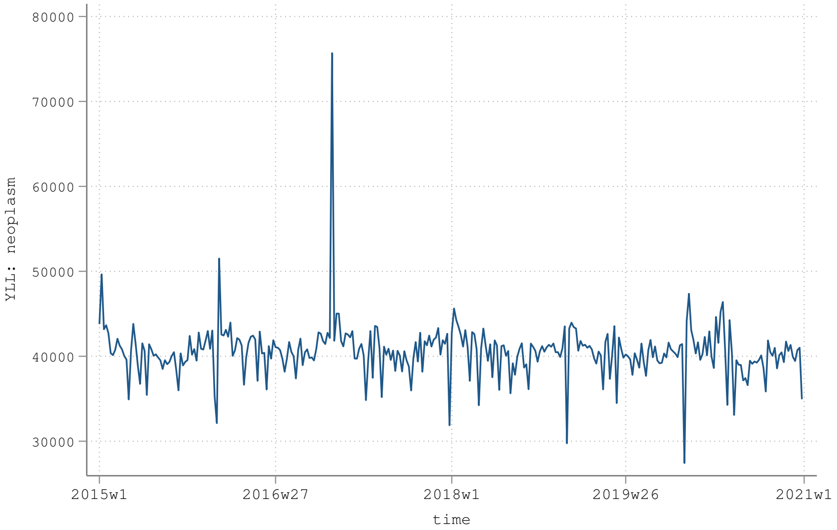

Figure 194: Years of Life Lost trend, cancer deaths, 2019-2020

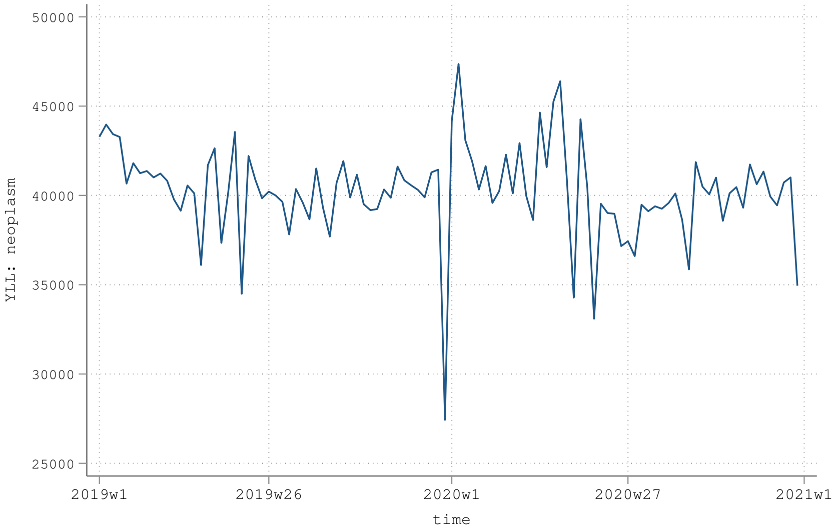

Figure 195: Excess Years of Life Lost trend, cancer deaths, 2015-2020

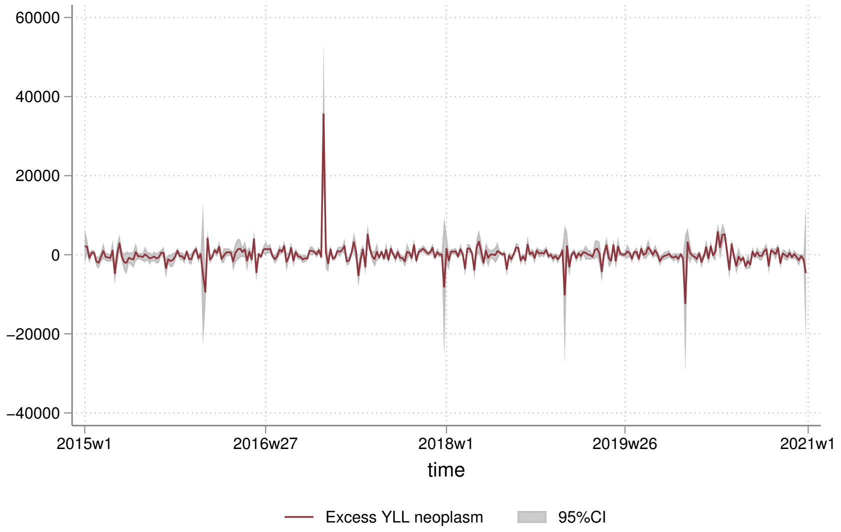

Figure 196: Excess Years of Life Lost trend, cancer deaths, 2019-2020

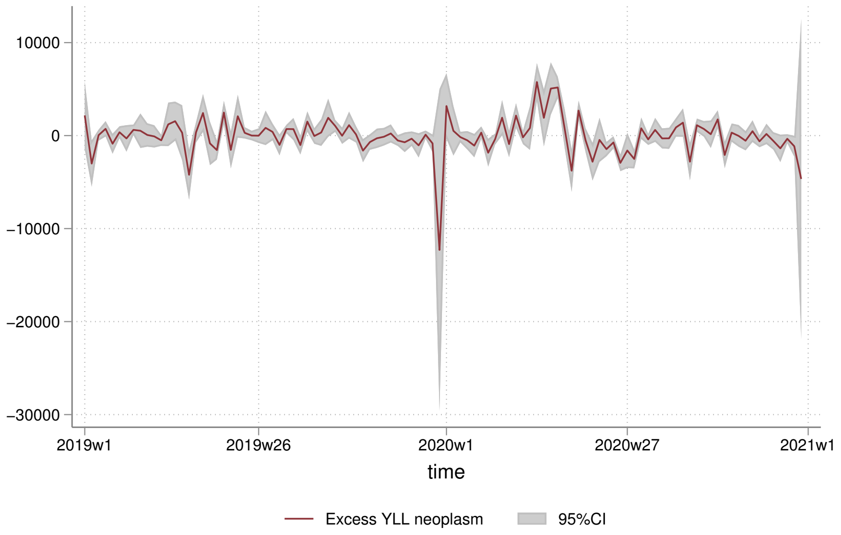

Figure 197: Observed vs Predicted Years of Life Lost trends, cancer deaths, 2015-2020

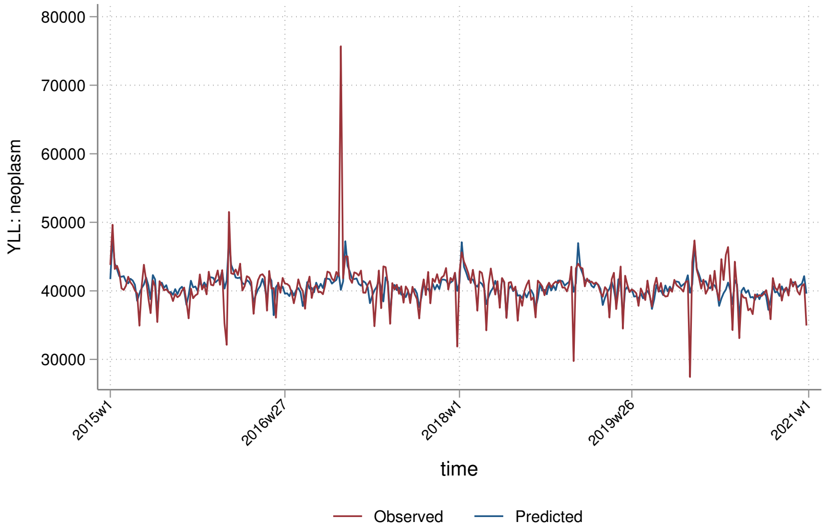

Figure 198: Observed vs Predicted Years of Life Lost trends, cancer deaths, 2019-2020

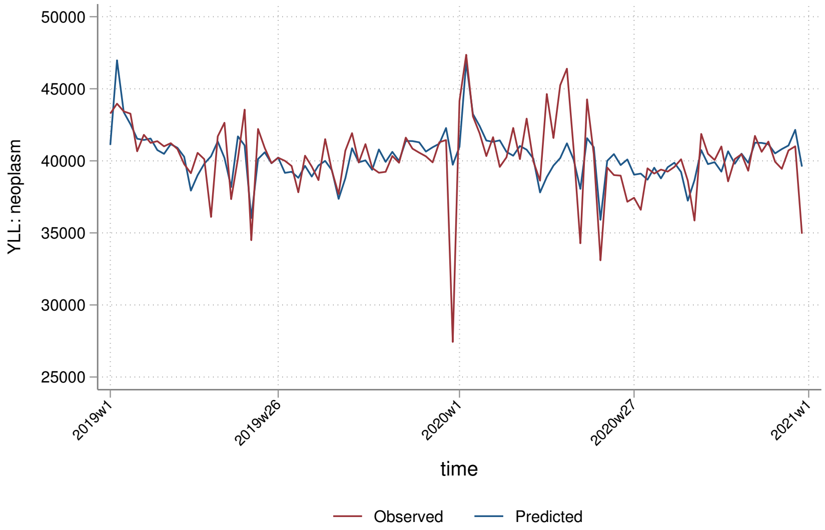

8.2.2 By sex

Figure 199: Years of Life Lost trend, cancer deaths by sex, 2015-2020

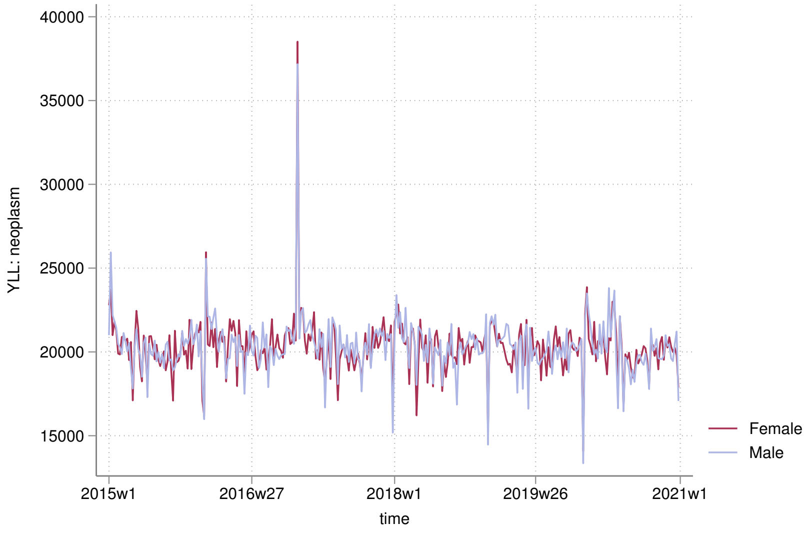

Figure 200: Years of Life Lost trend, cancer deaths by sex, 2019-2020

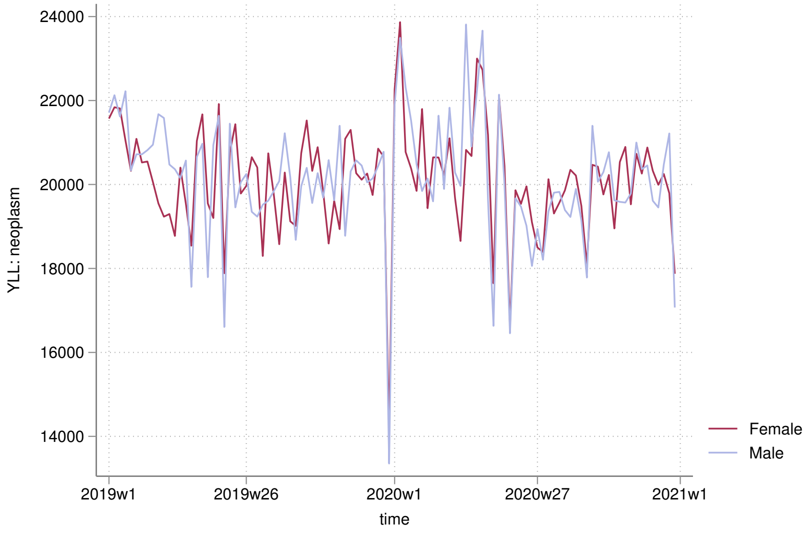

Figure 201: Excess Years of Life Lost trend, cancer deaths by sex, 2015-2020

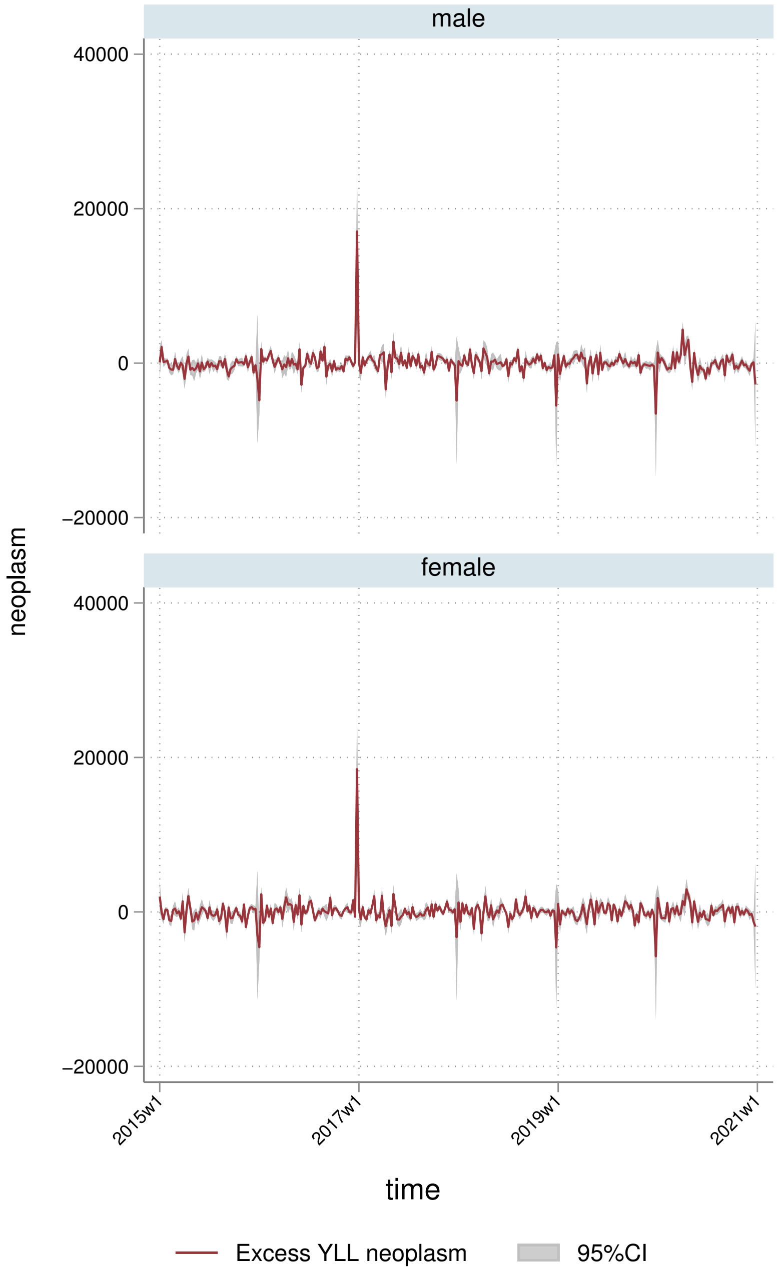

Figure 202: Excess Years of Life Lost trend, cancer deaths by sex, 2019-2020

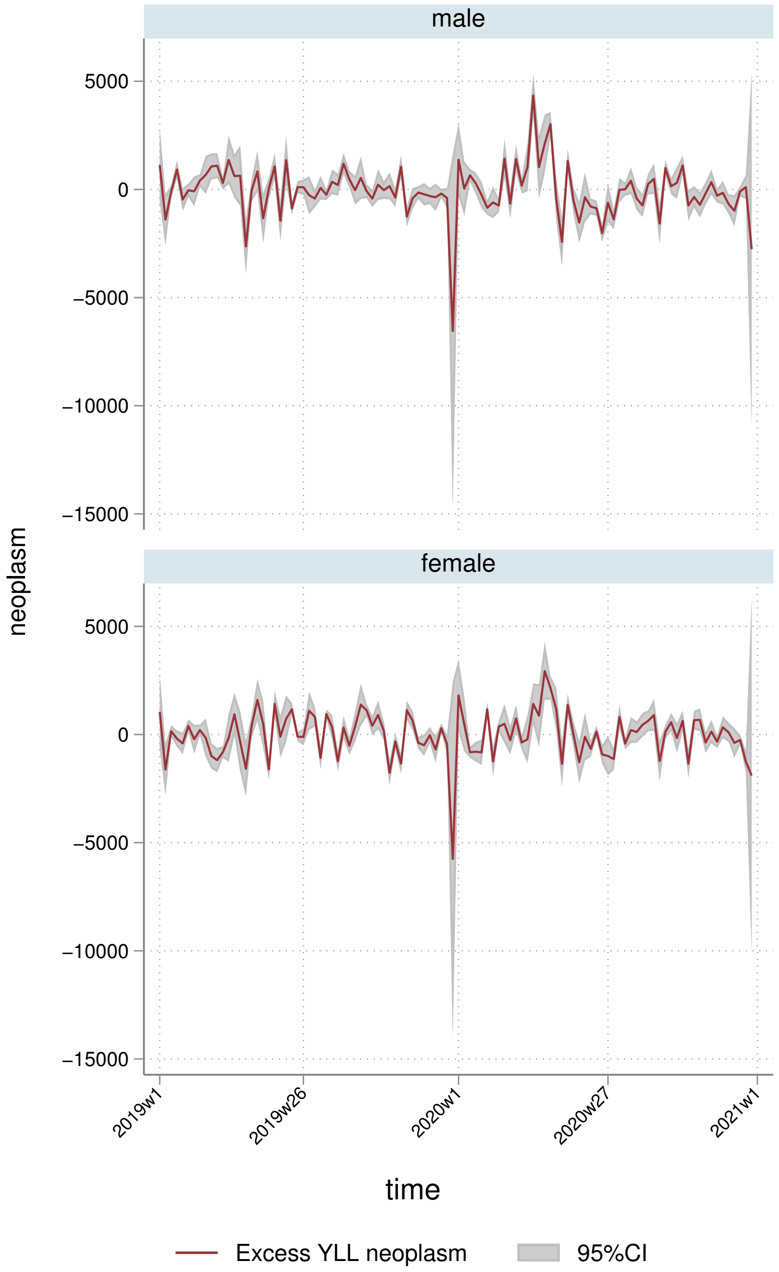

Figure 203: Observed vs Predicted Years of Life Lost trends, cancer deaths by sex, 2015-2020

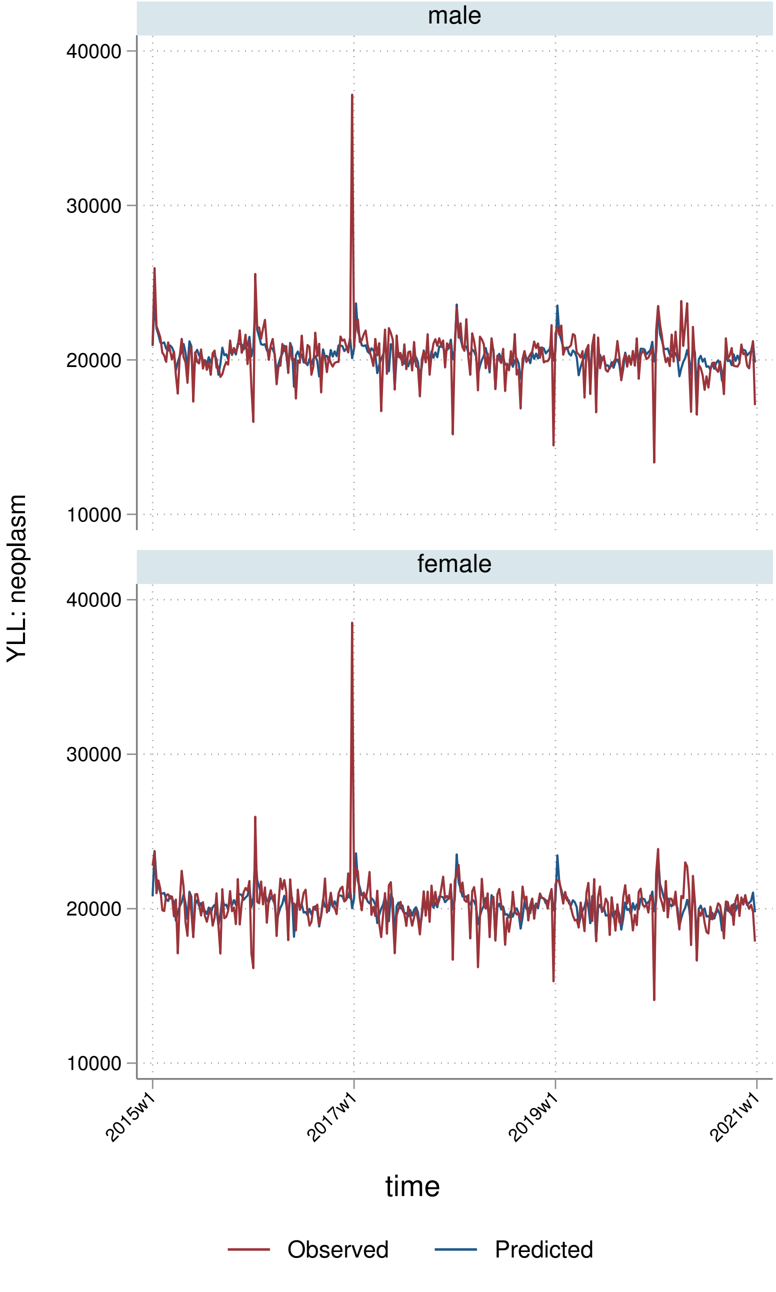

Figure 204: Observed vs Predicted Years of Life Lost trends, cancer deaths by sex, 2019-2020

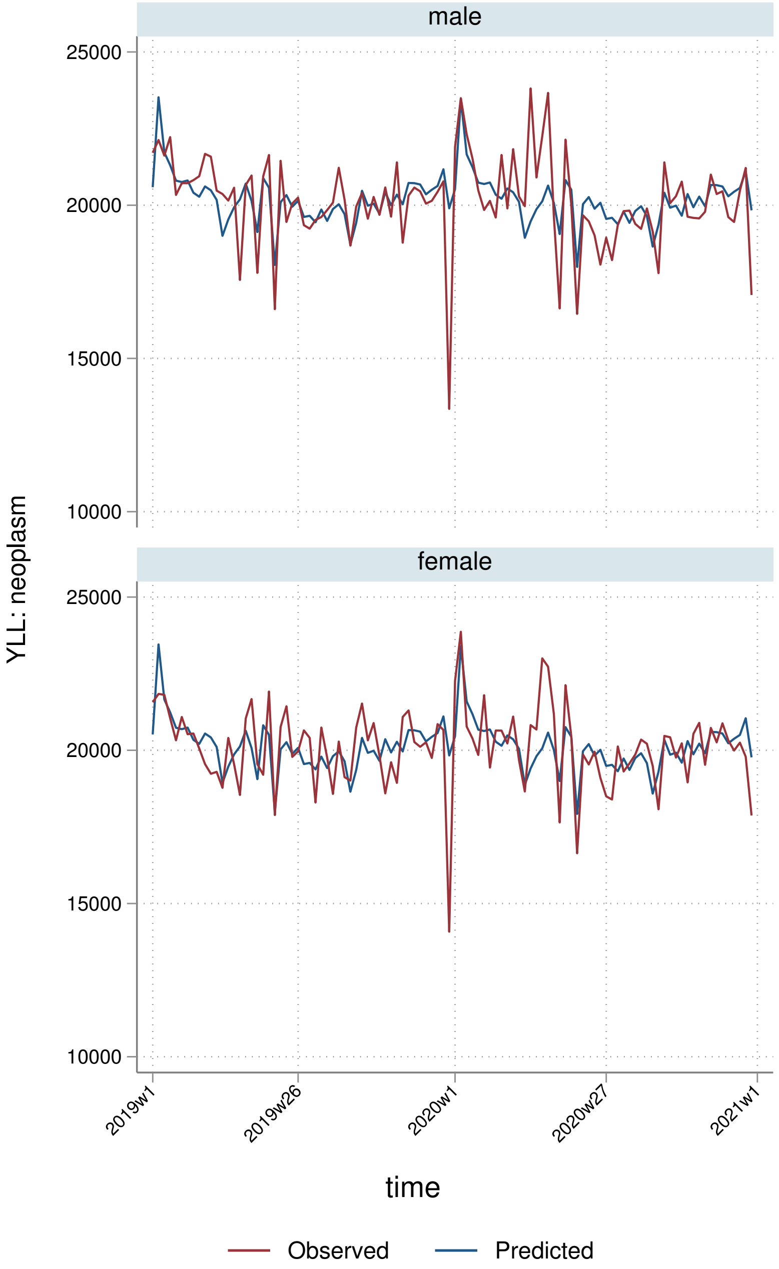

8.2.3 By deprivation quintile

Figure 205: Years of Life Lost trend, cancer deaths by deprivation quintile, 2015-2020

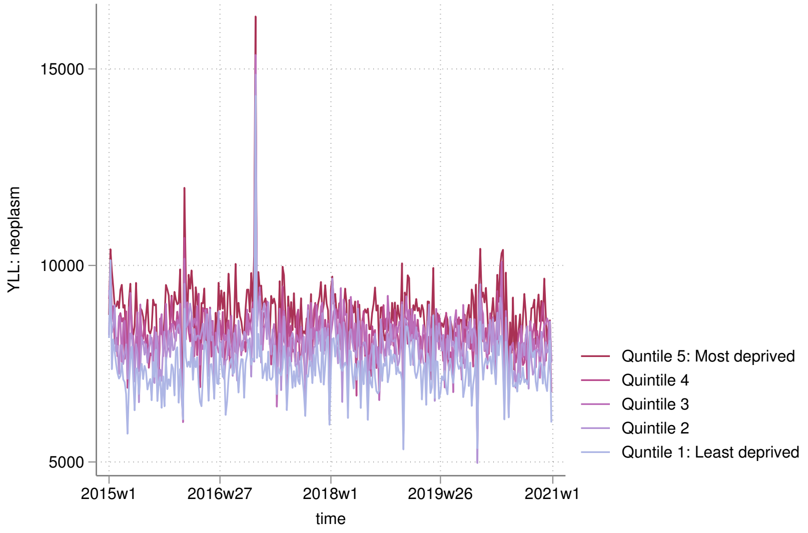

Figure 206: Years of Life Lost trend, cancer deaths by deprivation quintile, 2019-2020

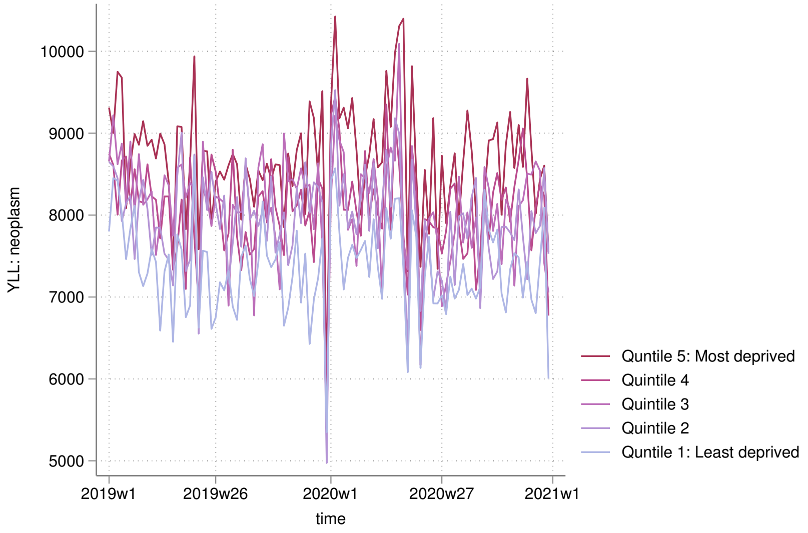

Figure 207: Excess Years of Life Lost trend, cancer deaths by deprivation quintile, 2015-2020

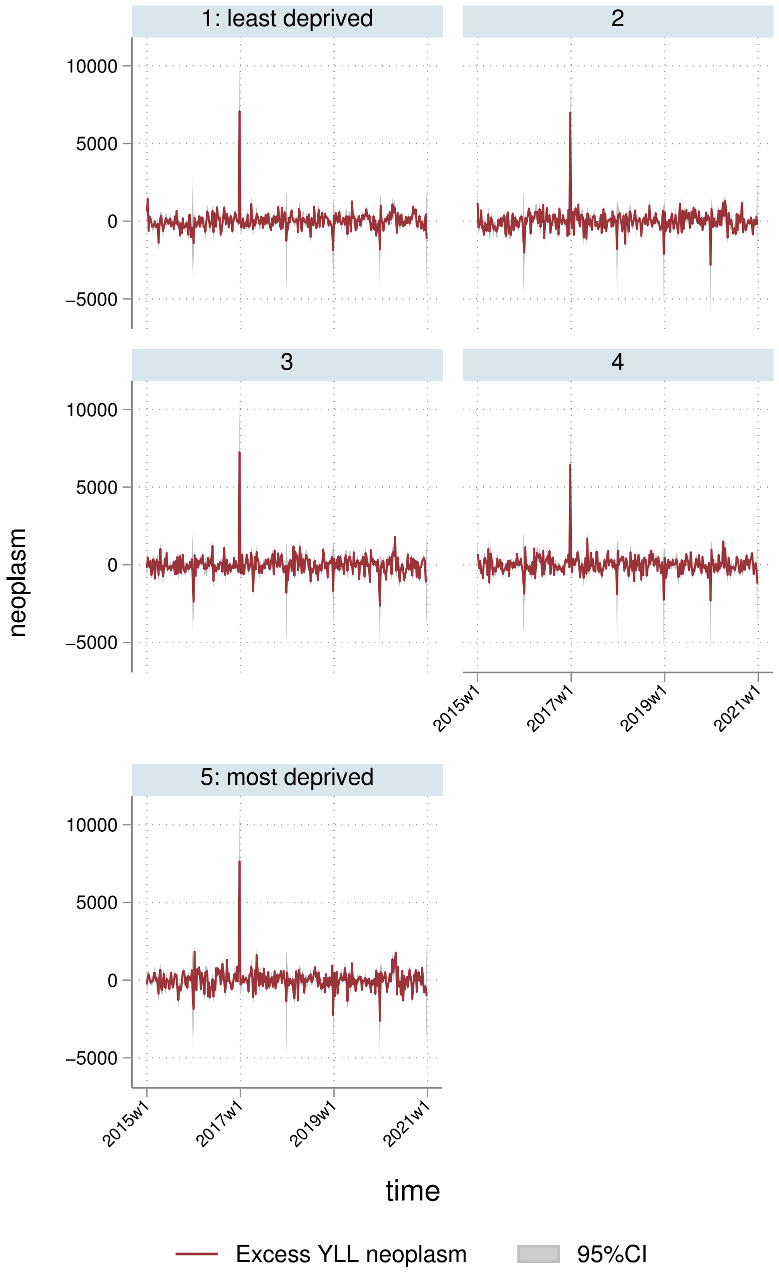

Figure 208: Excess Years of Life Lost trend, cancer deaths by deprivation quintile, 2019-2020

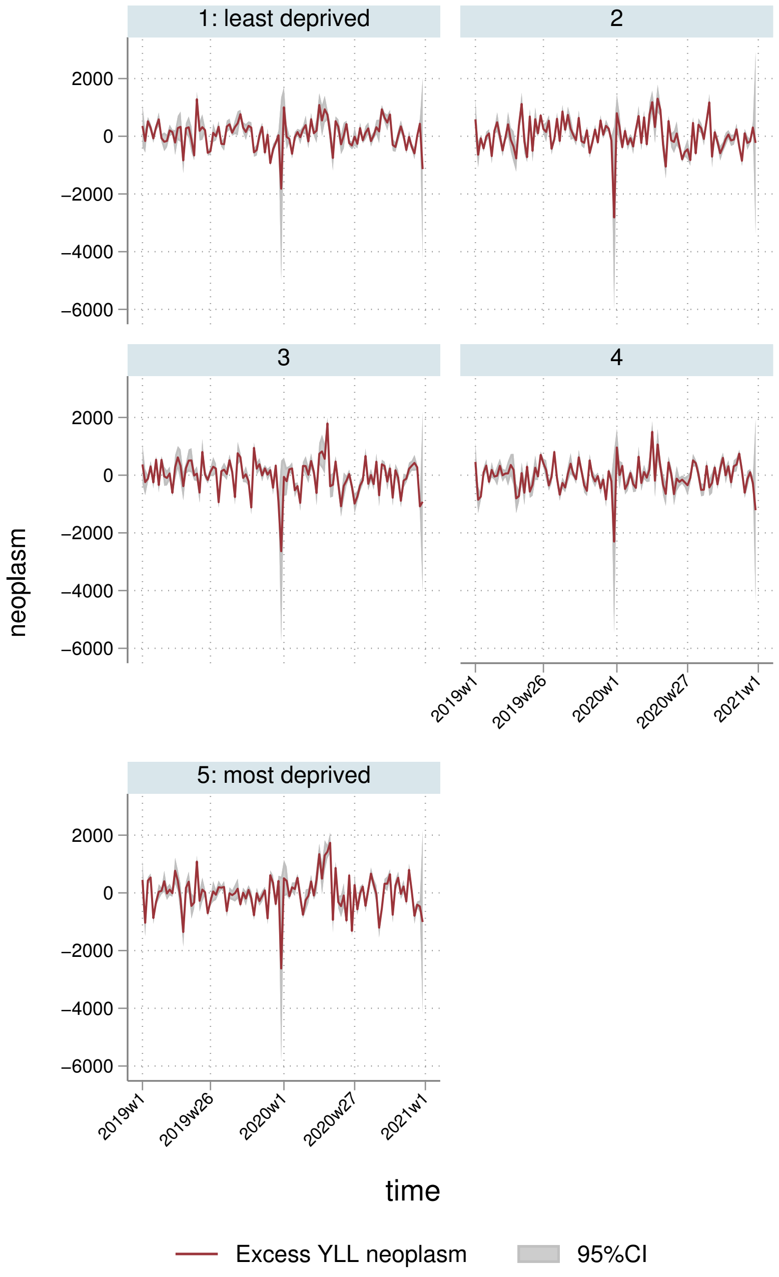

Figure 209: Observed vs Predicted Years of Life Lost trends, cancer deaths by deprivation quintile, 2015-2020

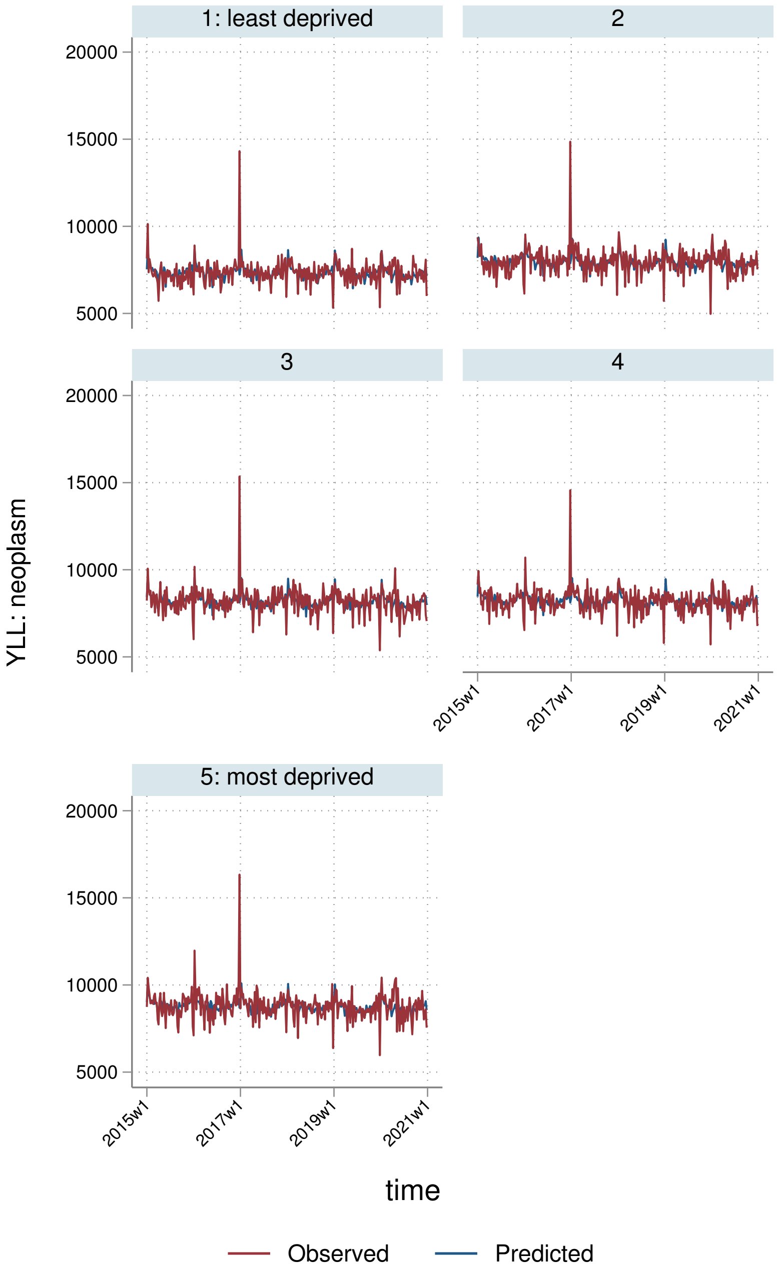

Figure 210: Observed vs Predicted Years of Life Lost trends, cancer deaths by deprivation quintile, 2019-2020

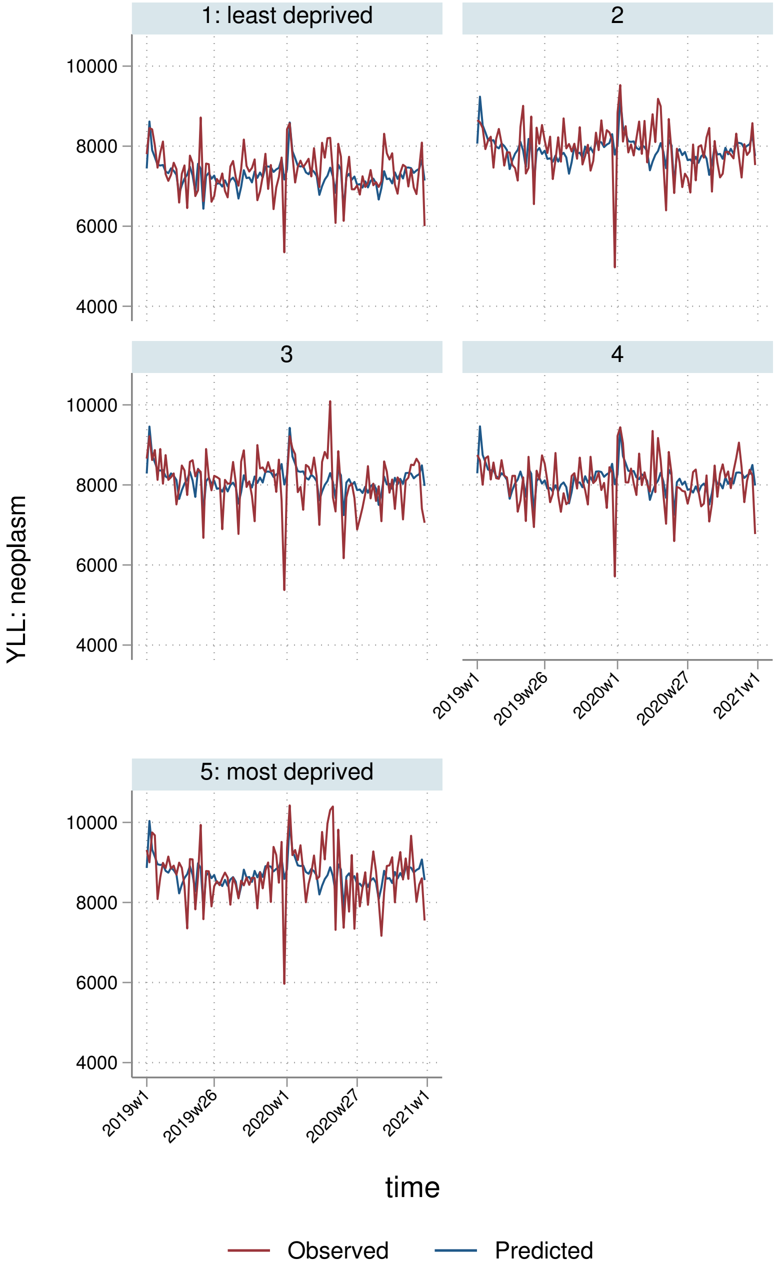

8.2.4 By Strategic Health Authority

Figure 211: Years of Life Lost trend, cancer deaths by region, 2015-2020

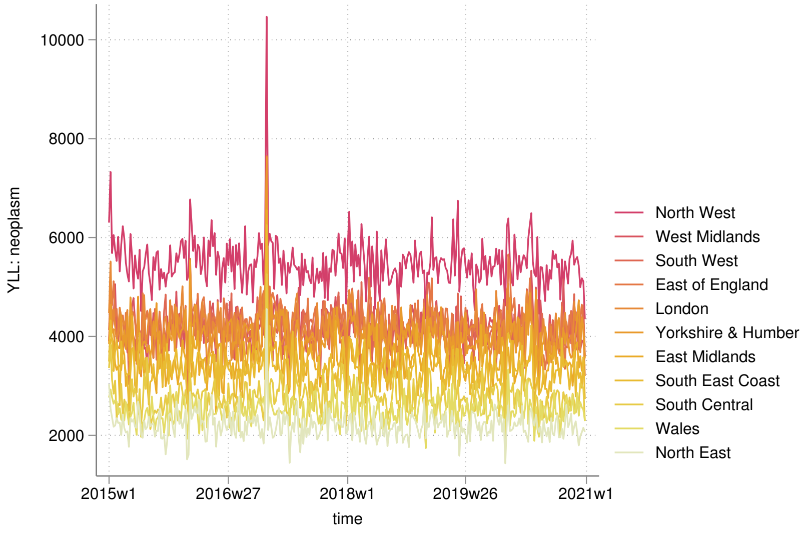

Figure 212: Years of Life Lost trend, cancer deaths by region, 2019-2020

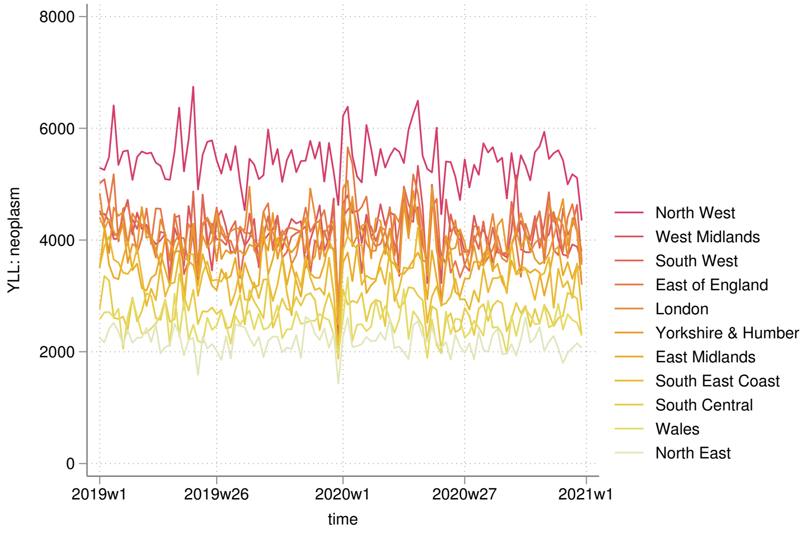

Figure 213: Excess Years of Life Lost trend, cancer deaths by region, 2015-2020

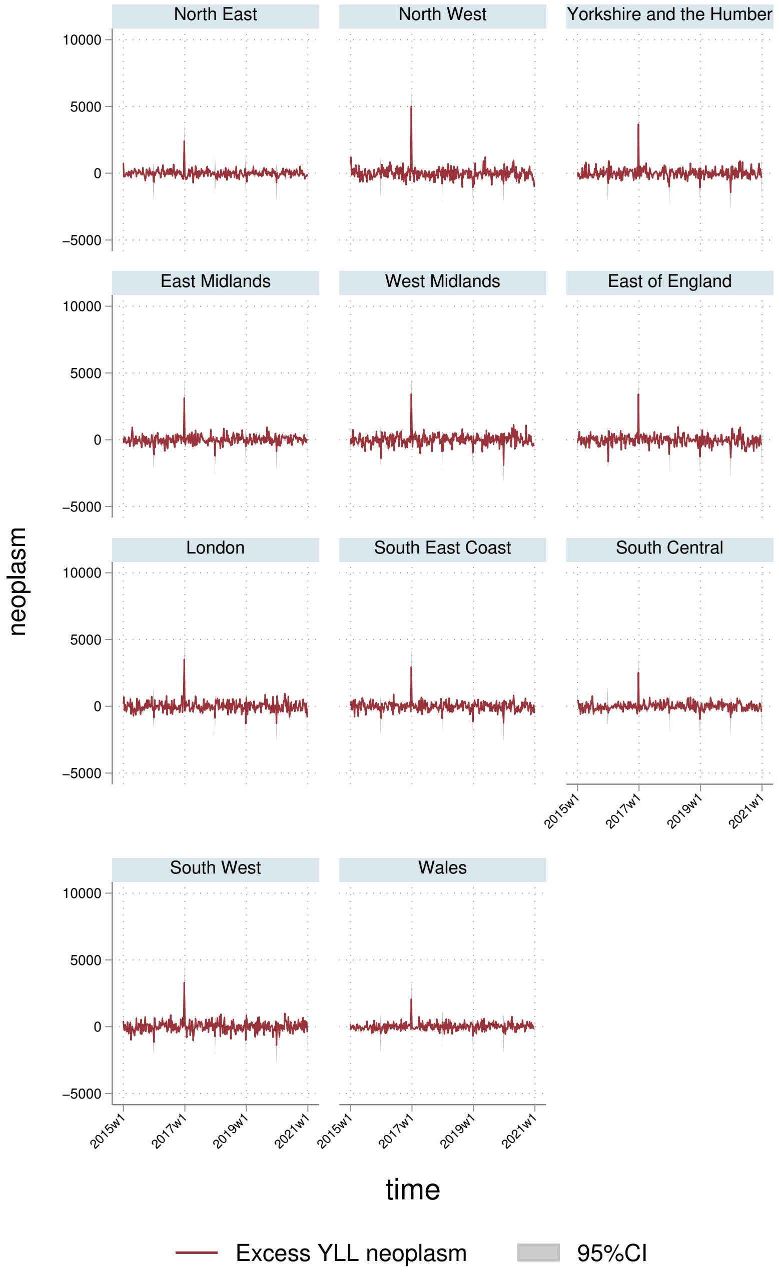

Figure 214: Excess Years of Life Lost trend, cancer deaths by region, 2019-2020

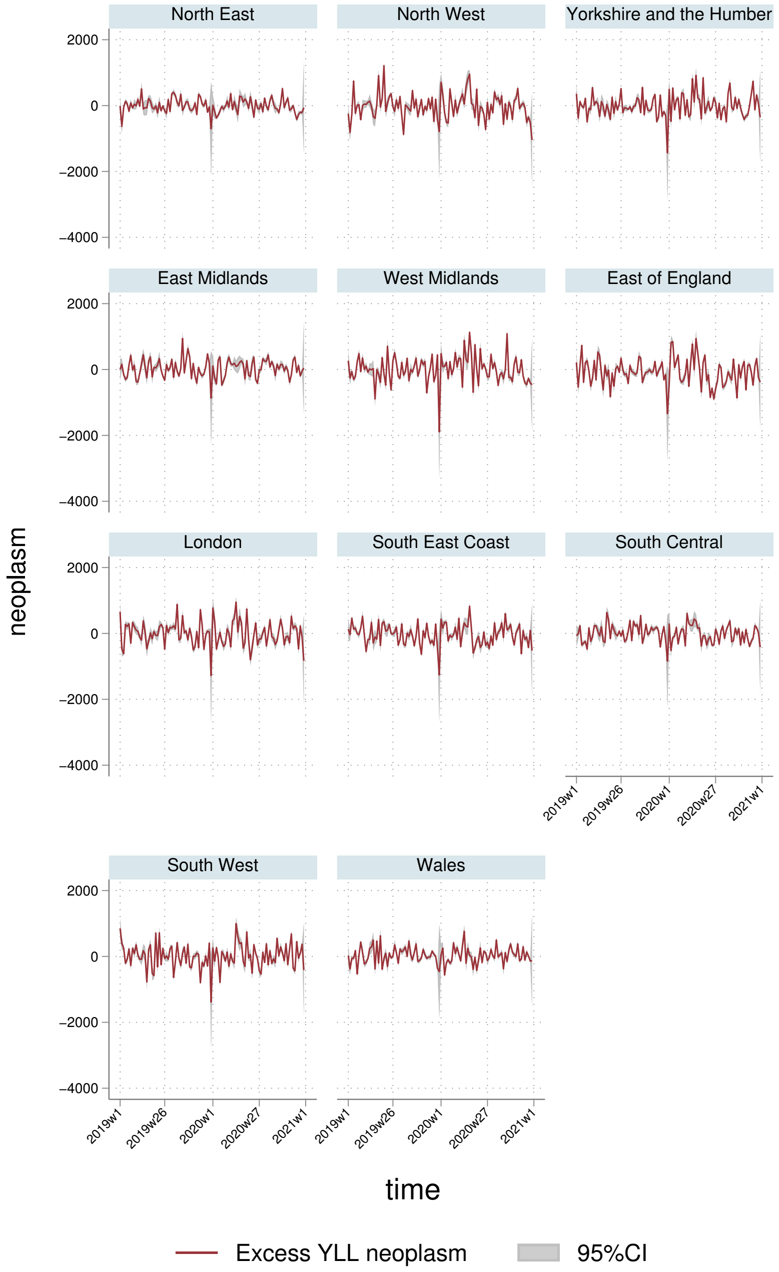

Figure 215: Observed vs Predicted Years of Life Lost trends, cancer deaths by region, 2015-2020

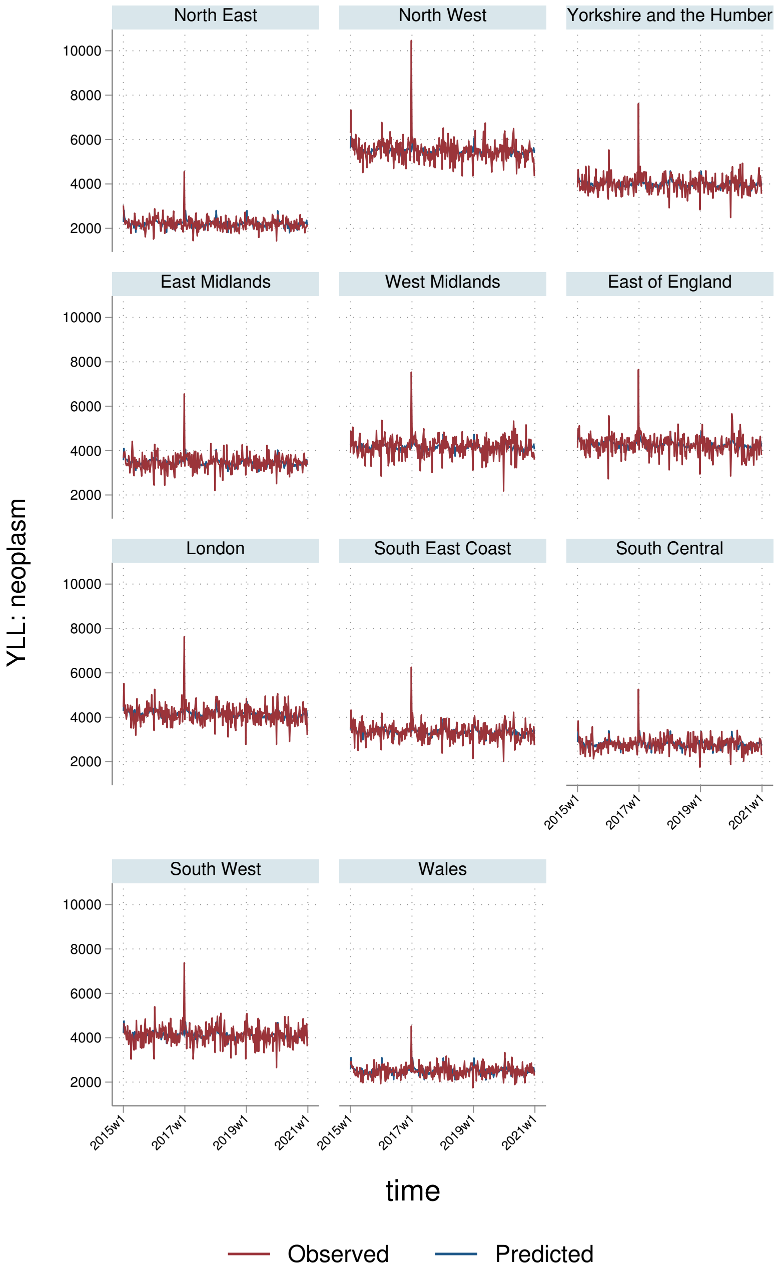

8.3 YLLs per $100,000$ population

8.3.1 England-Wales aggregate

Figure 216: Observed vs Predicted Years of Life Lost trends, cancer deaths by region, 2019-2020

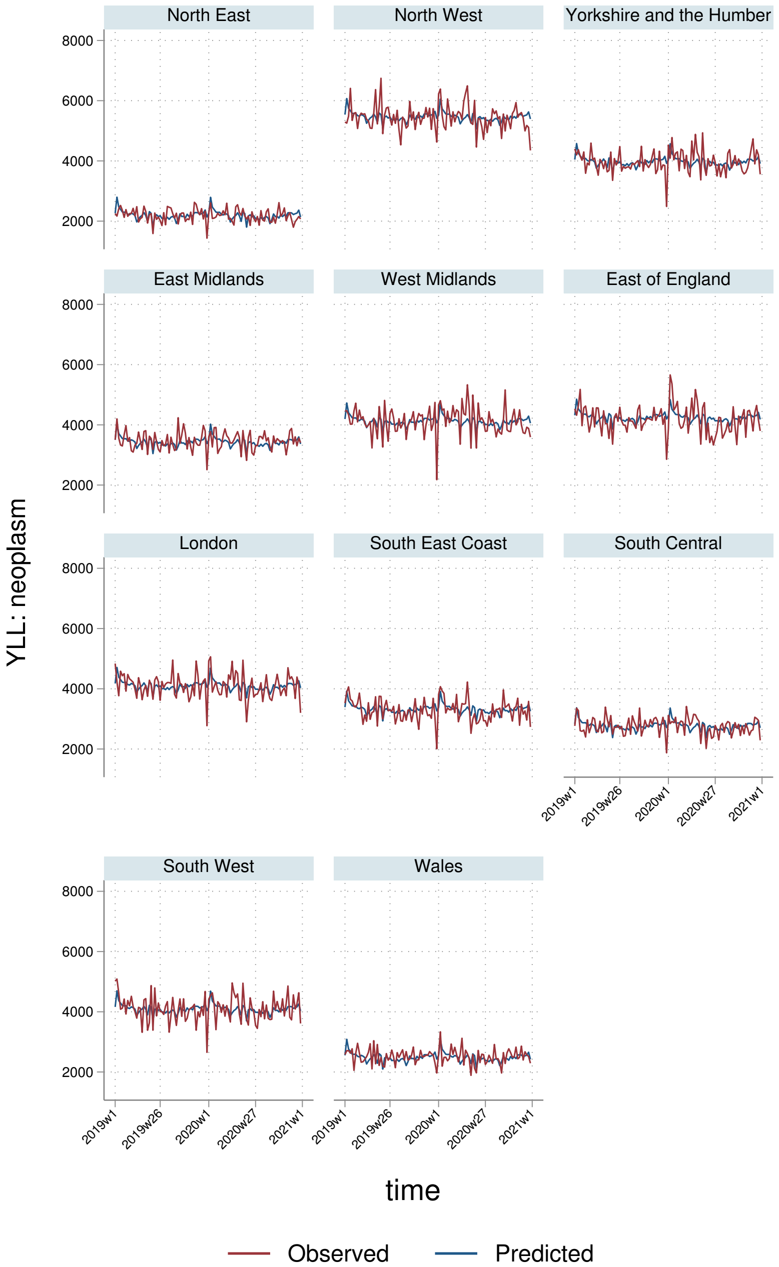

Figure 217: Years of Life Lost trend per 100, 000 population, cancer deaths, 2015-2020

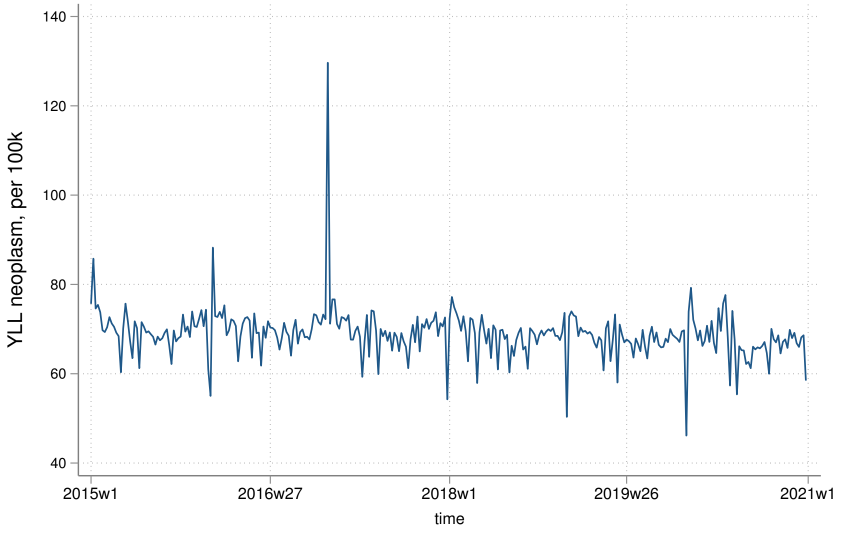

Figure 218: Years of Life Lost trend per 100, 000 population, cancer deaths, 2019-2020

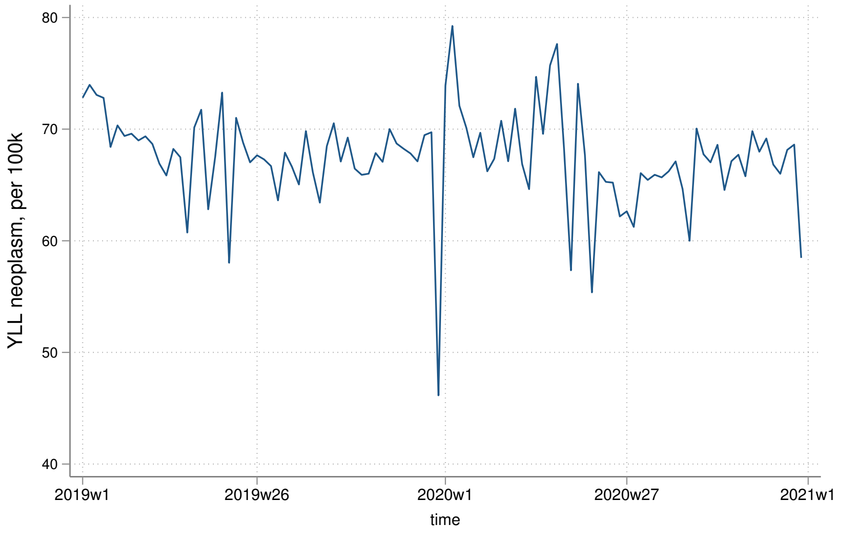

Figure 219: Excess Years of Life Lost trend per 100,000 population, cancer deaths, 2015-2020

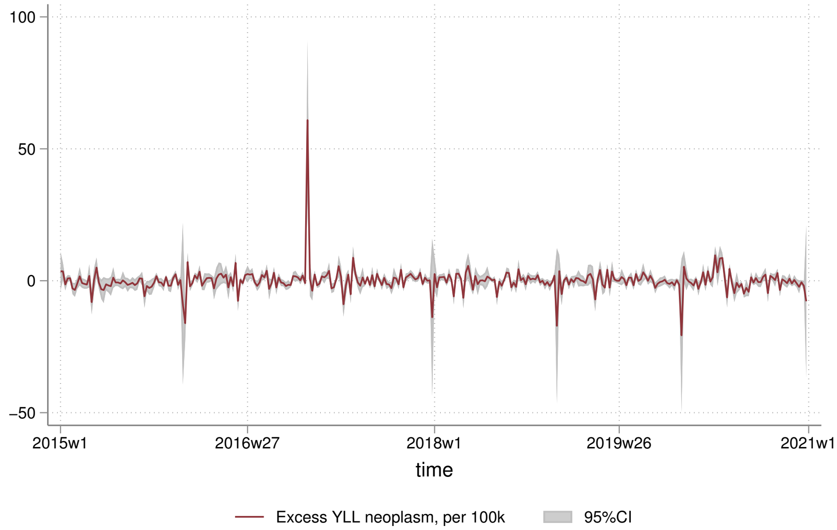

Figure 220: Excess Years of Life Lost trend per 100,000 population, cancer deaths, 2019-2020

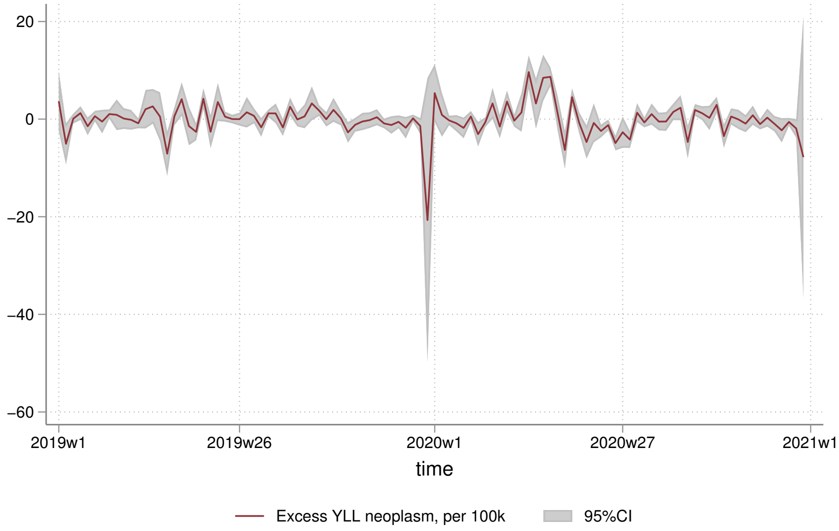

Figure 221: Observed vs Predicted Years of Life Lost trends per 100,000 population, cancer deaths, 2015-2020

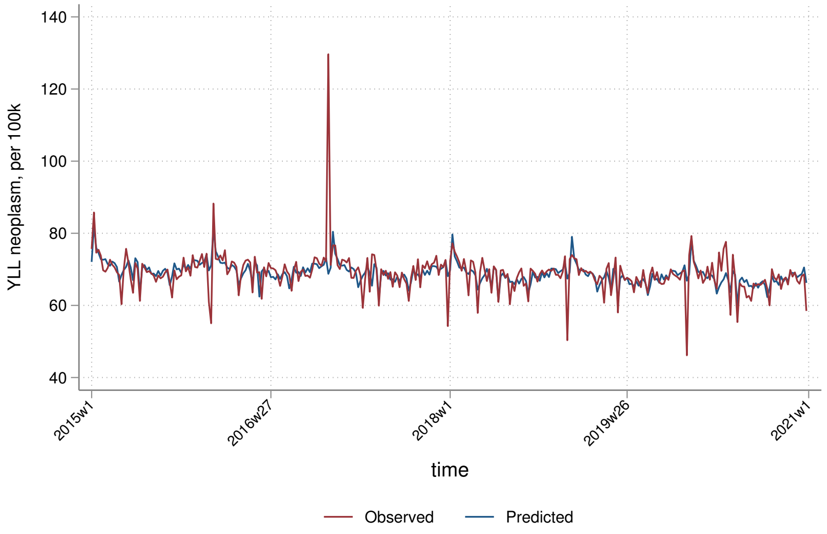

Figure 222: Observed vs Predicted Years of Life Lost trends per 100,000 population, cancer deaths, 2019-2020

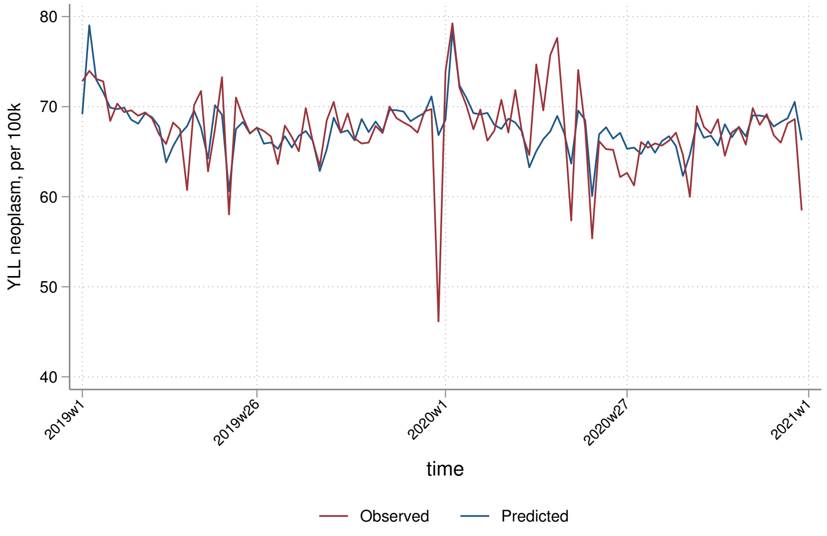

8.3.2 By sex

Figure 223: Years of Life Lost trend per 100,000 population, cancer deaths by sex, 2015-2020

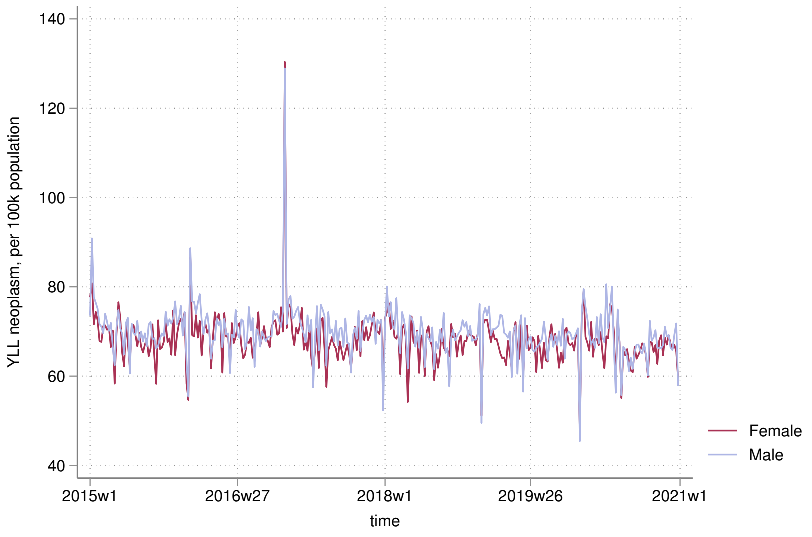

Figure 224: Years of Life Lost trend per 100,000 population, cancer deaths by sex, 2019-2020

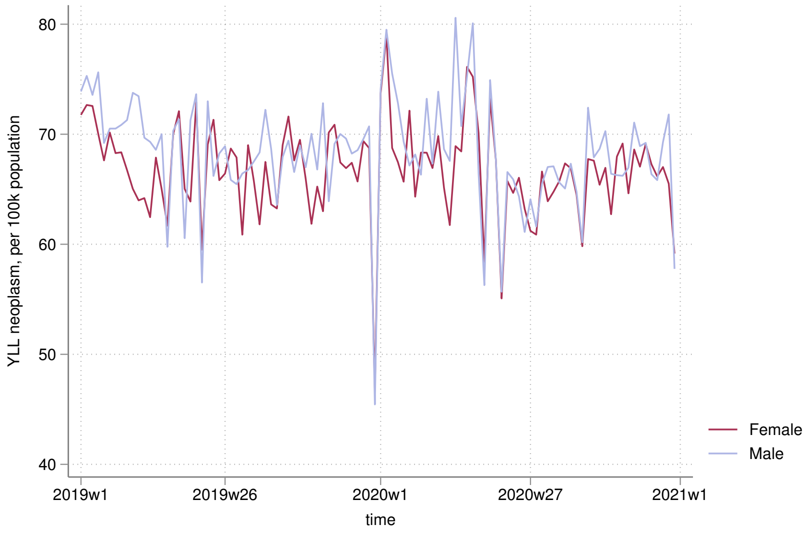

Figure 225: Excess Years of Life Lost trend per 100,000 population, cancer deaths by sex, 2015-2020

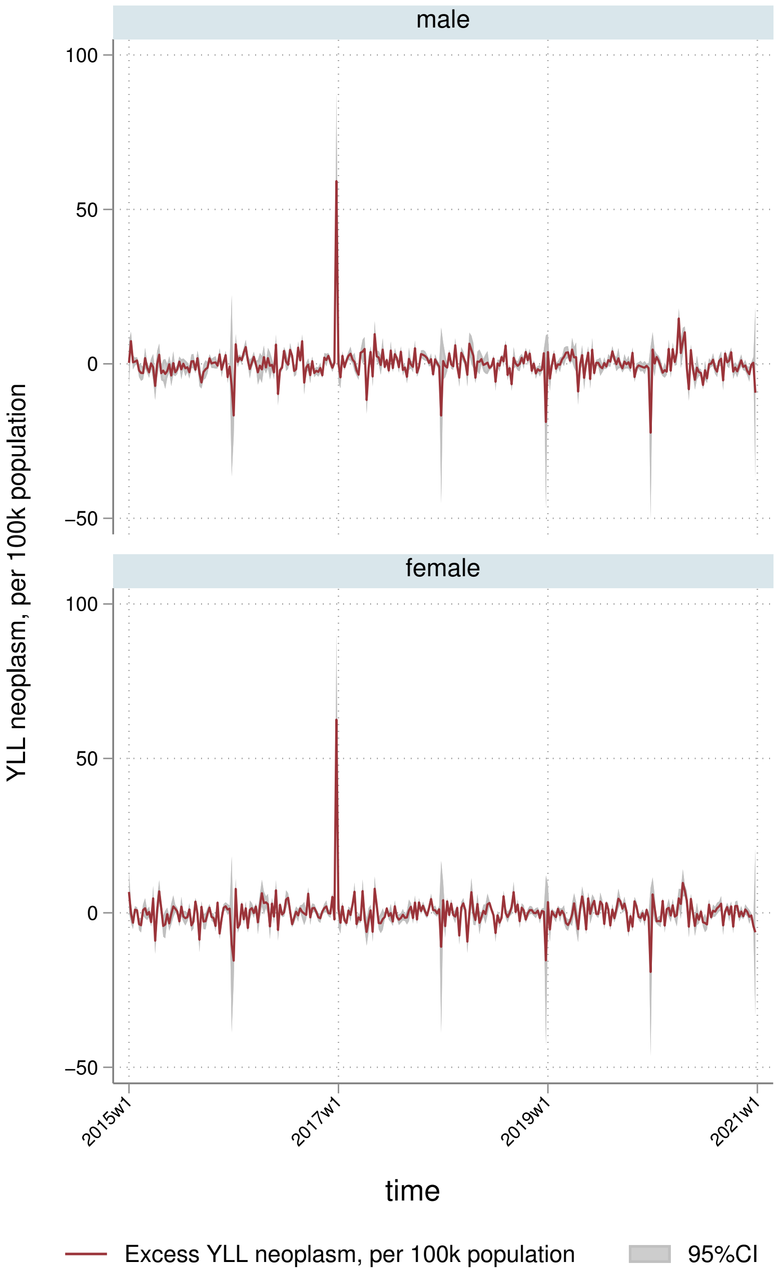

Figure 226: Excess Years of Life Lost trend per 100,000 population, cancer deaths by sex, 2019-2020

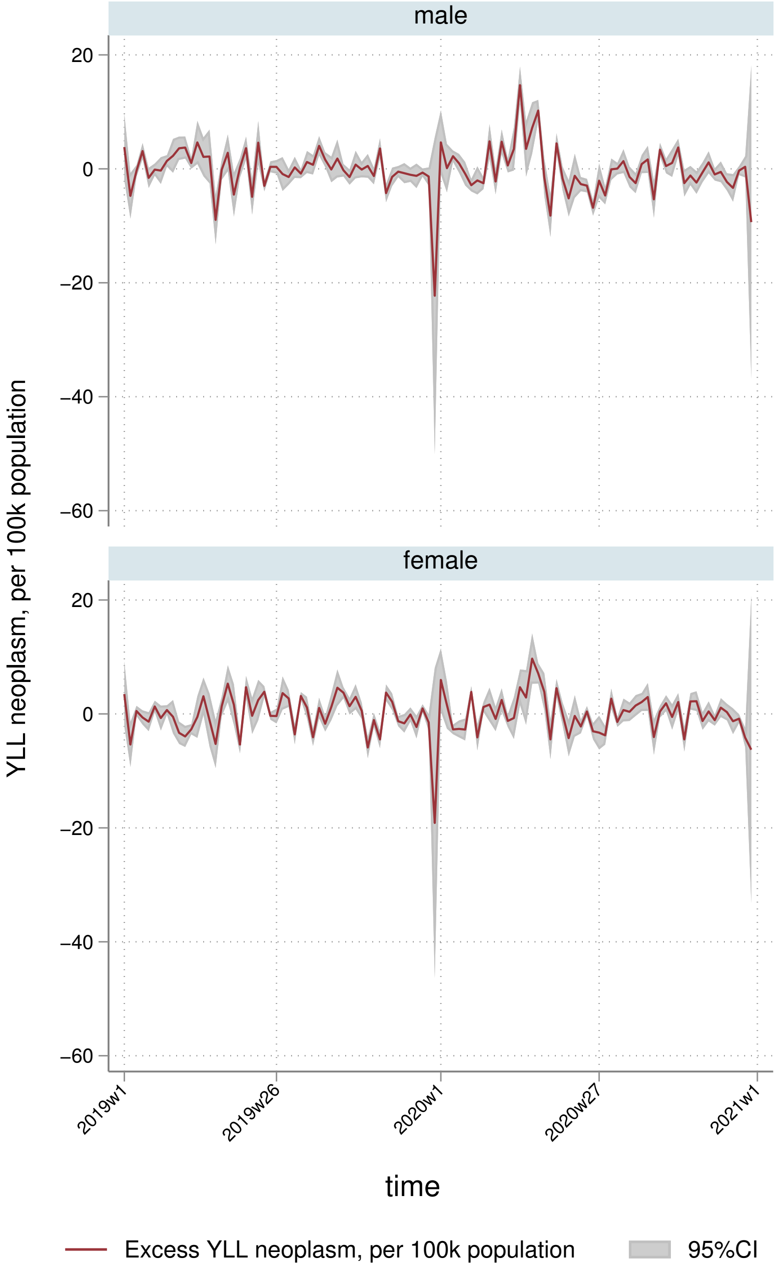

Figure 227: Observed vs Predicted Years of Life Lost trends per 100,000 population, cancer deaths by sex, 2015-2020

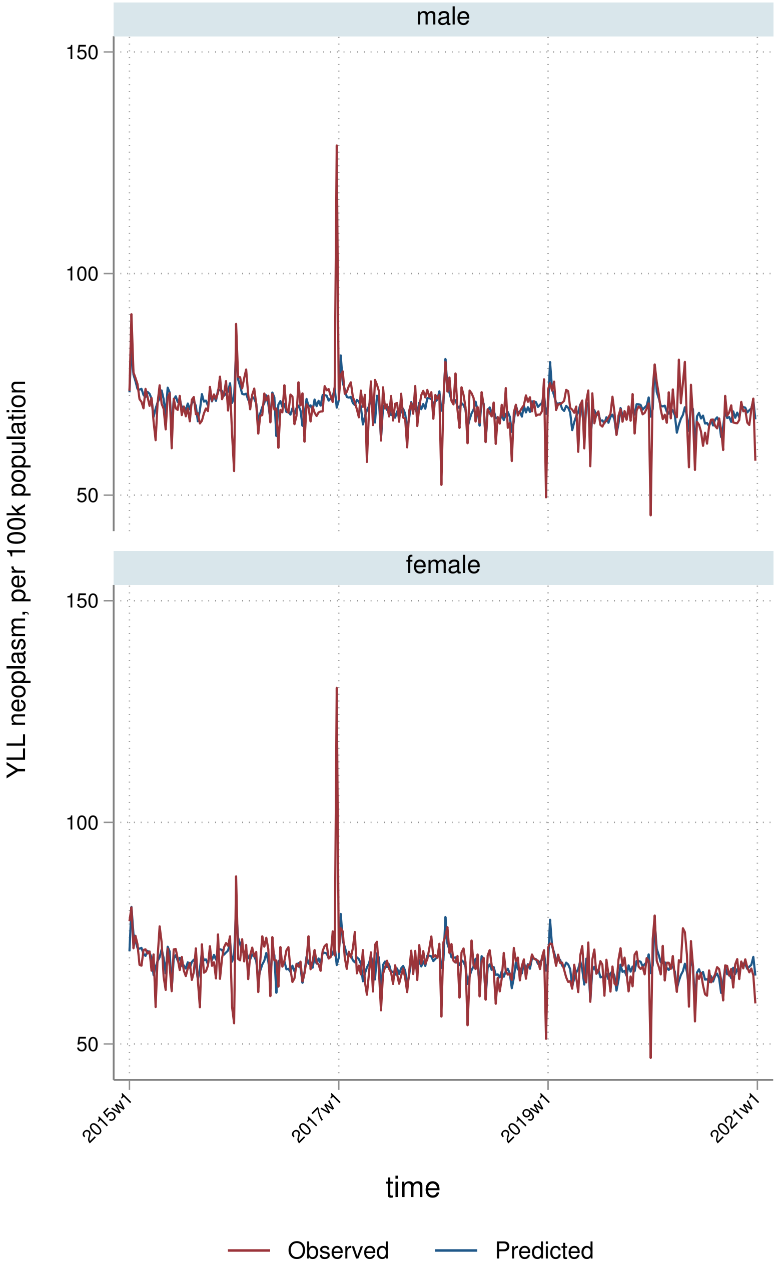

Figure 228: Observed vs Predicted Years of Life Lost trends per 100, 000 population, cancer deaths by sex, 2019-2020

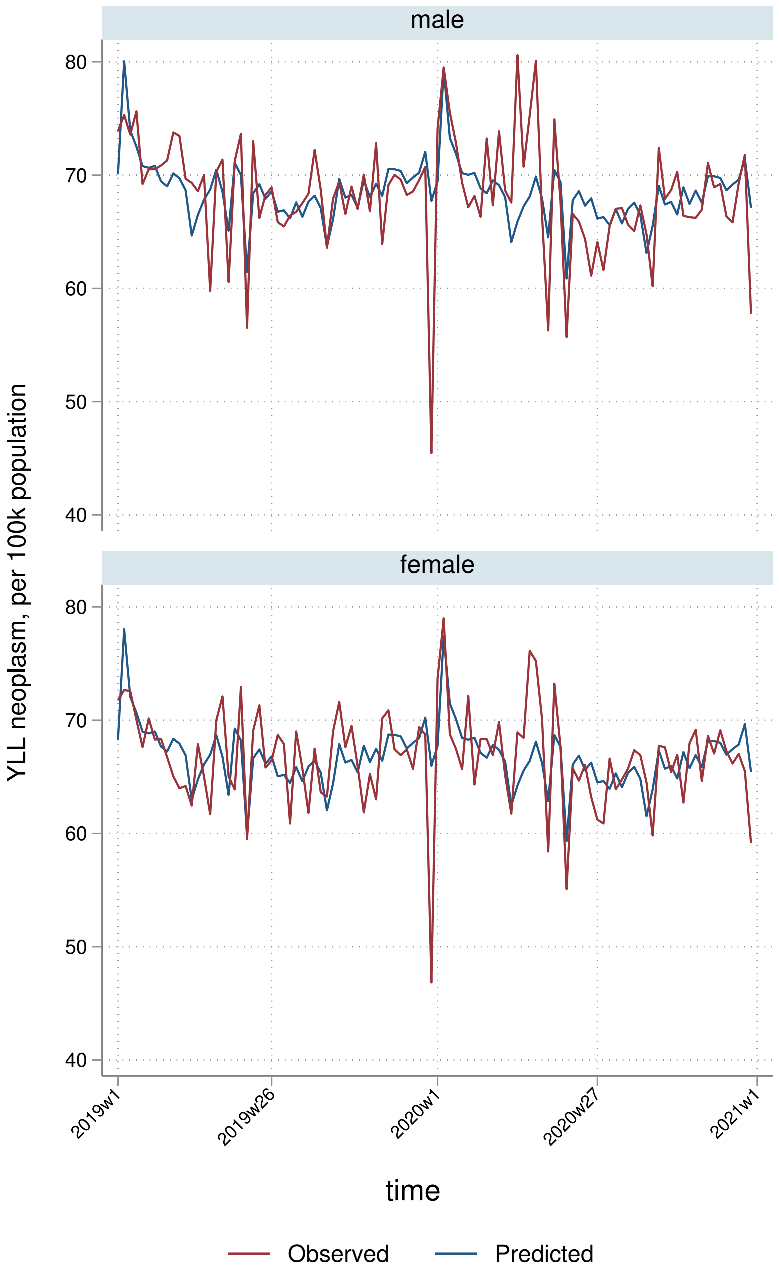

8.3.3 By deprivation quintile

Figure 229: Years of Life Lost trend per 100,000 population, cancer deaths by deprivation quintile, 2015-2020

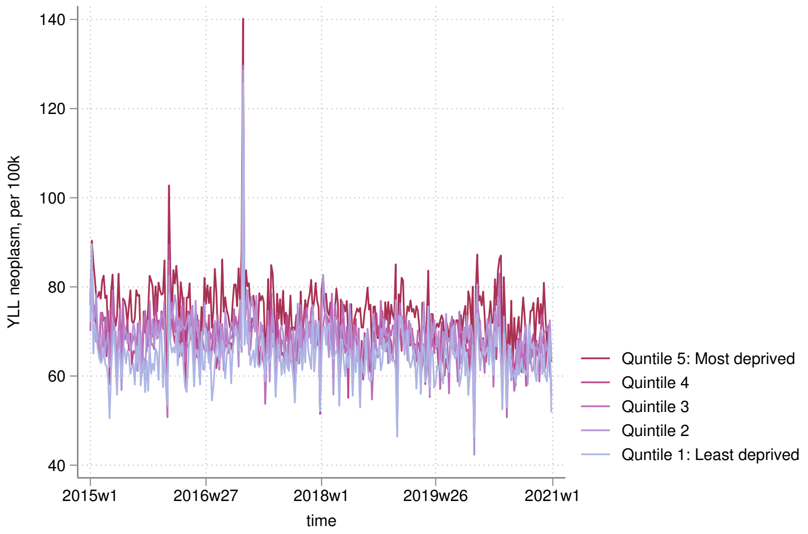

Figure 230: Years of Life Lost trend per 100,000 population, cancer deaths by deprivation quintile, 2019-2020

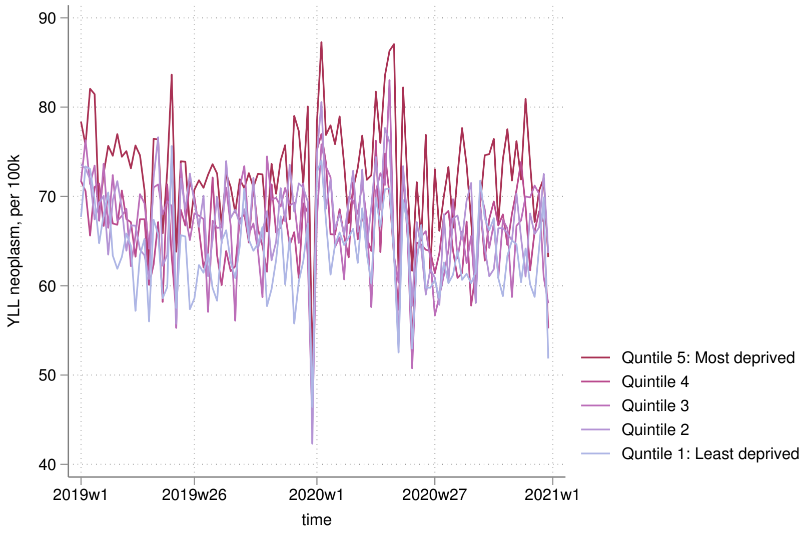

Figure 231: Excess Years of Life Lost trend per 100,000 population, cancer deaths by deprivation quintile, 2015-2020

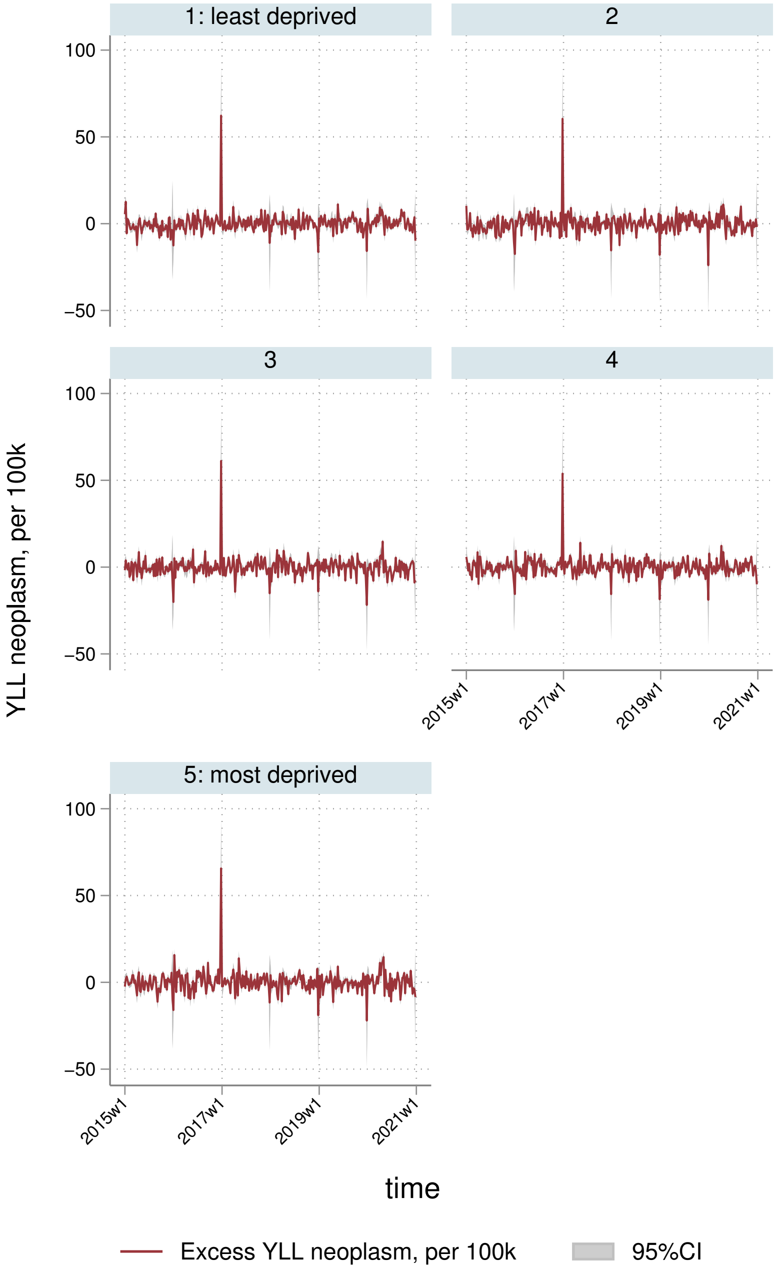

Figure 232: Excess Years of Life Lost trend per 100,000 population, cancer deaths by deprivation quintile, 2019-2020

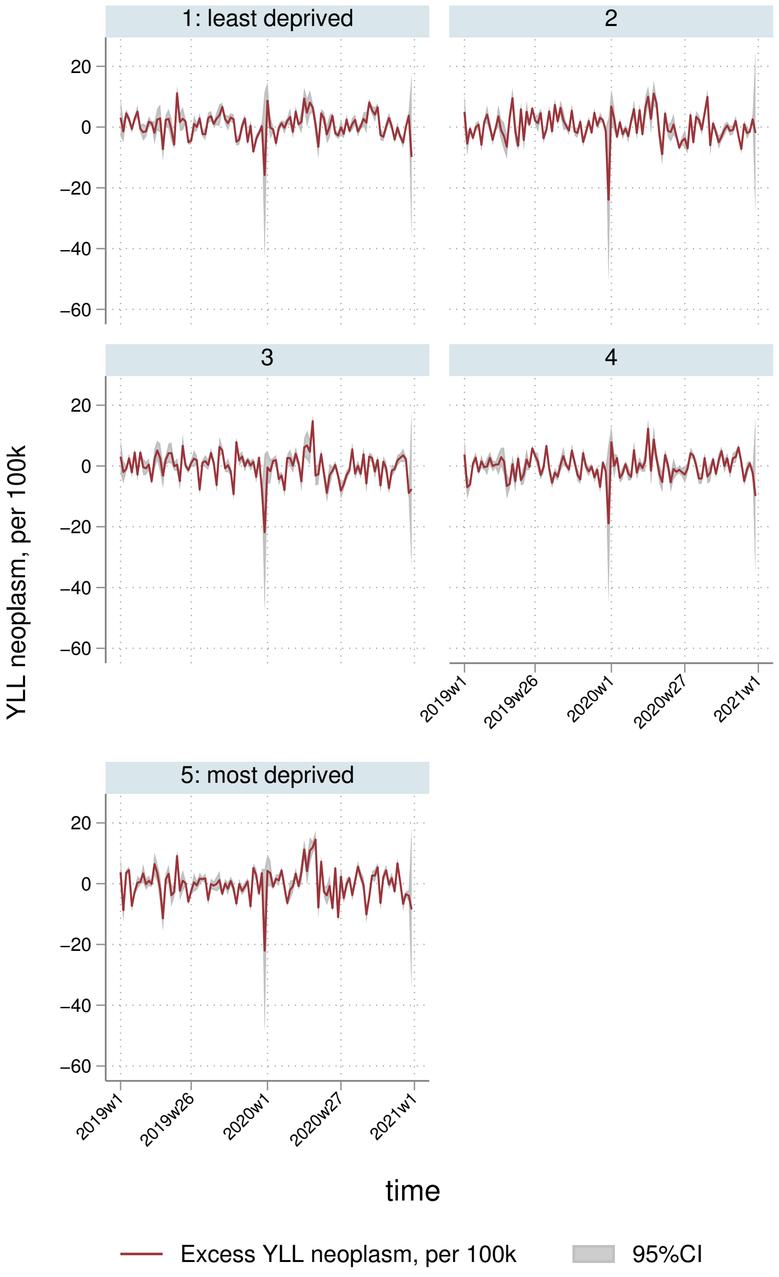

Figure 233: Observed vs Predicted Years of Life Lost trends per 100,000 population, cancer deaths by deprivation quintile, 2015-2020

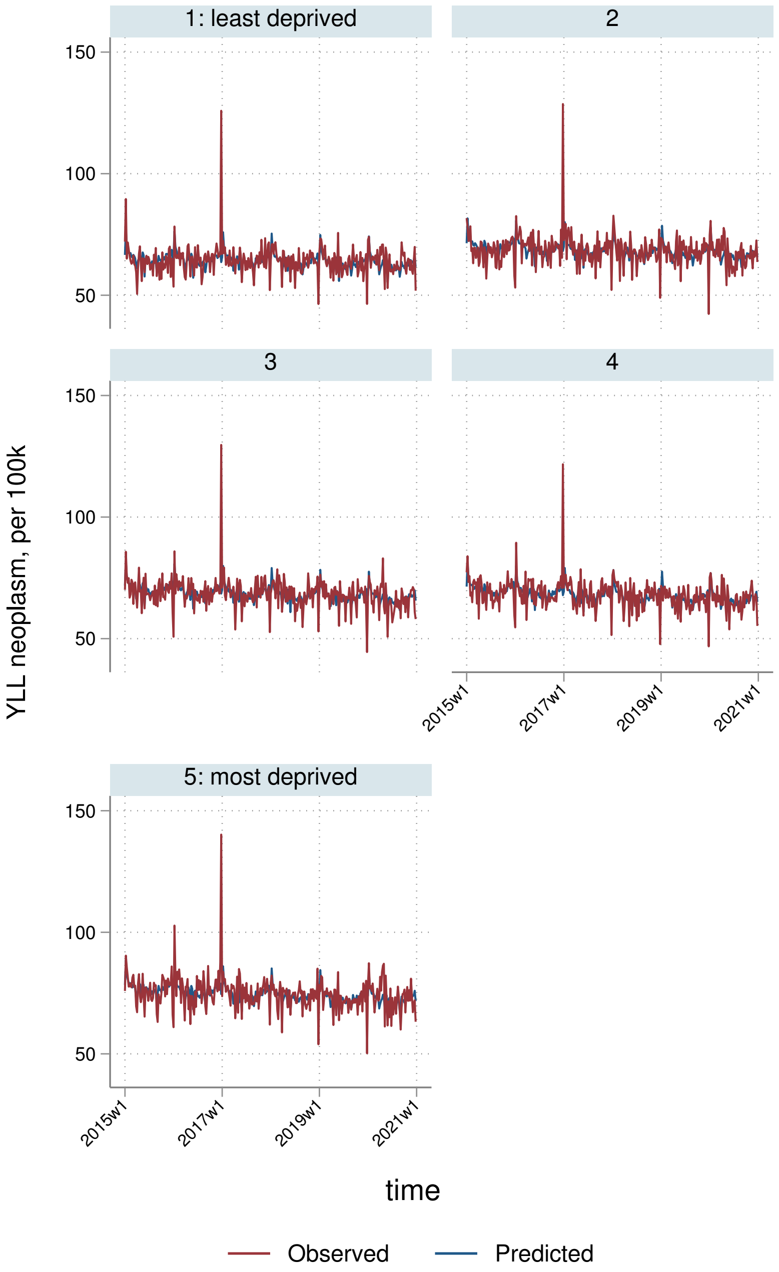

Figure 234: Observed vs Predicted Years of Life Lost trends per 100, 000 population, cancer deaths by deprivation quintile, 2019-2020

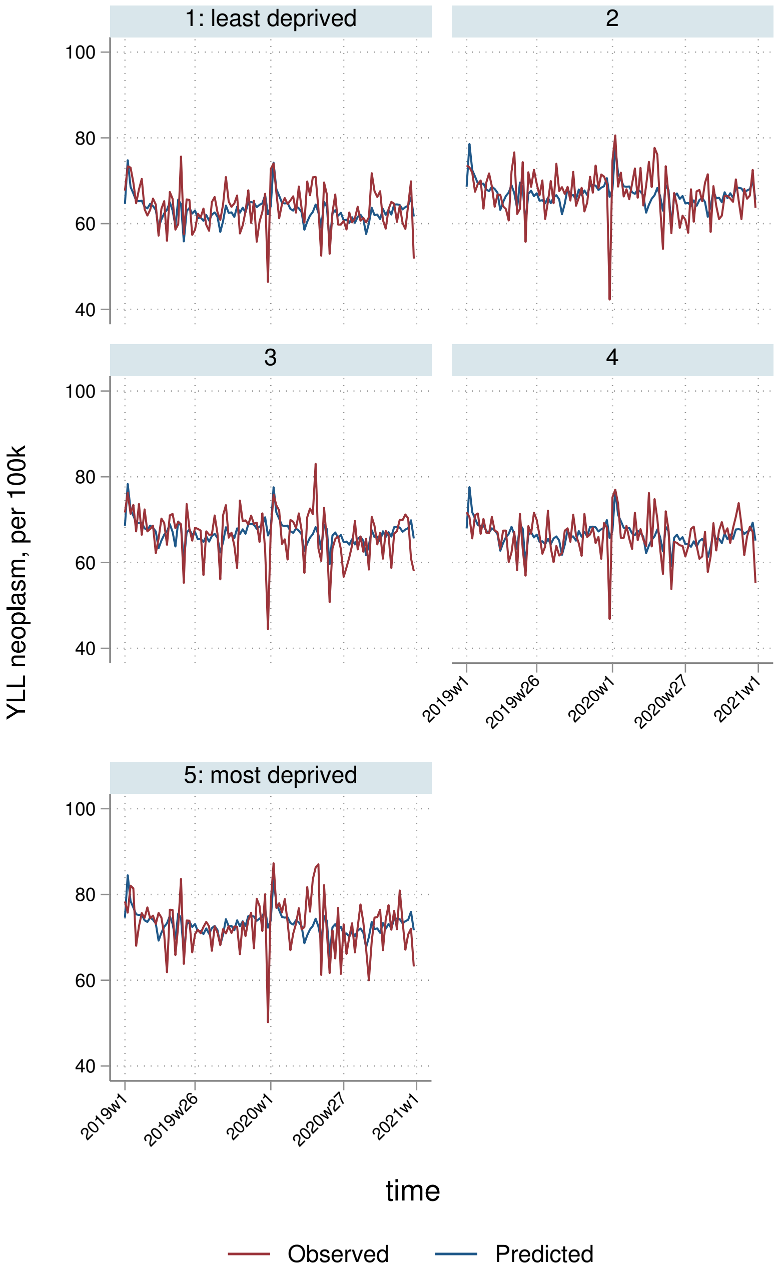

8.3.4 By Strategic Health Authority

Figure 235: Years of Life Lost trend per 100,000 population, cancer deaths by region, 2015-2020

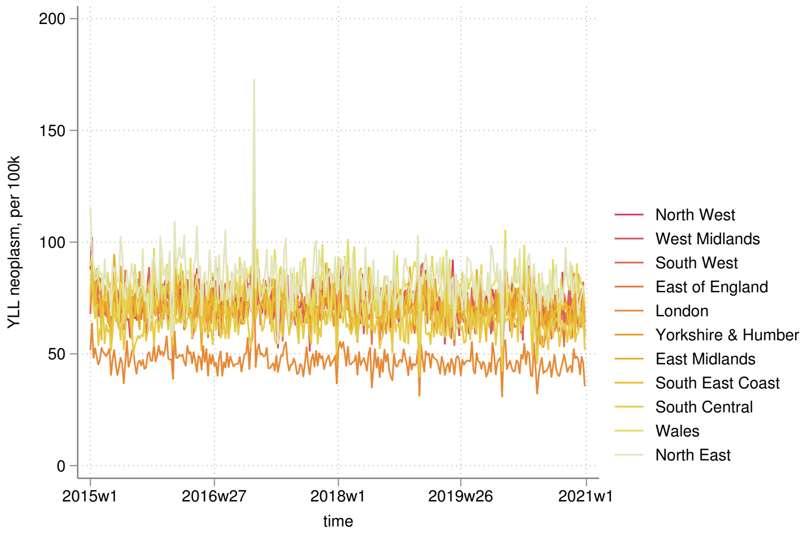

Figure 236: Years of Life Lost trend per 100,000 population, cancer deaths by region, 2019-2020

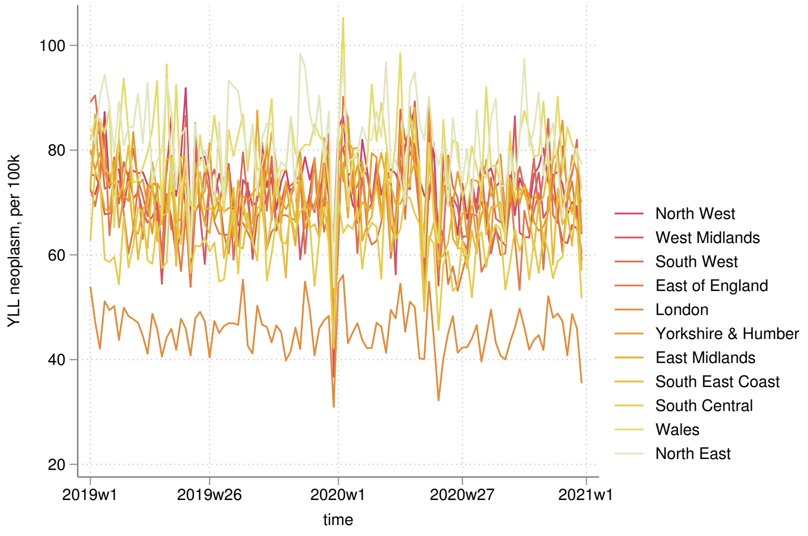

Figure 237: Excess Years of Life Lost trend per 100,000 population, cancer deaths by region, 2015-2020

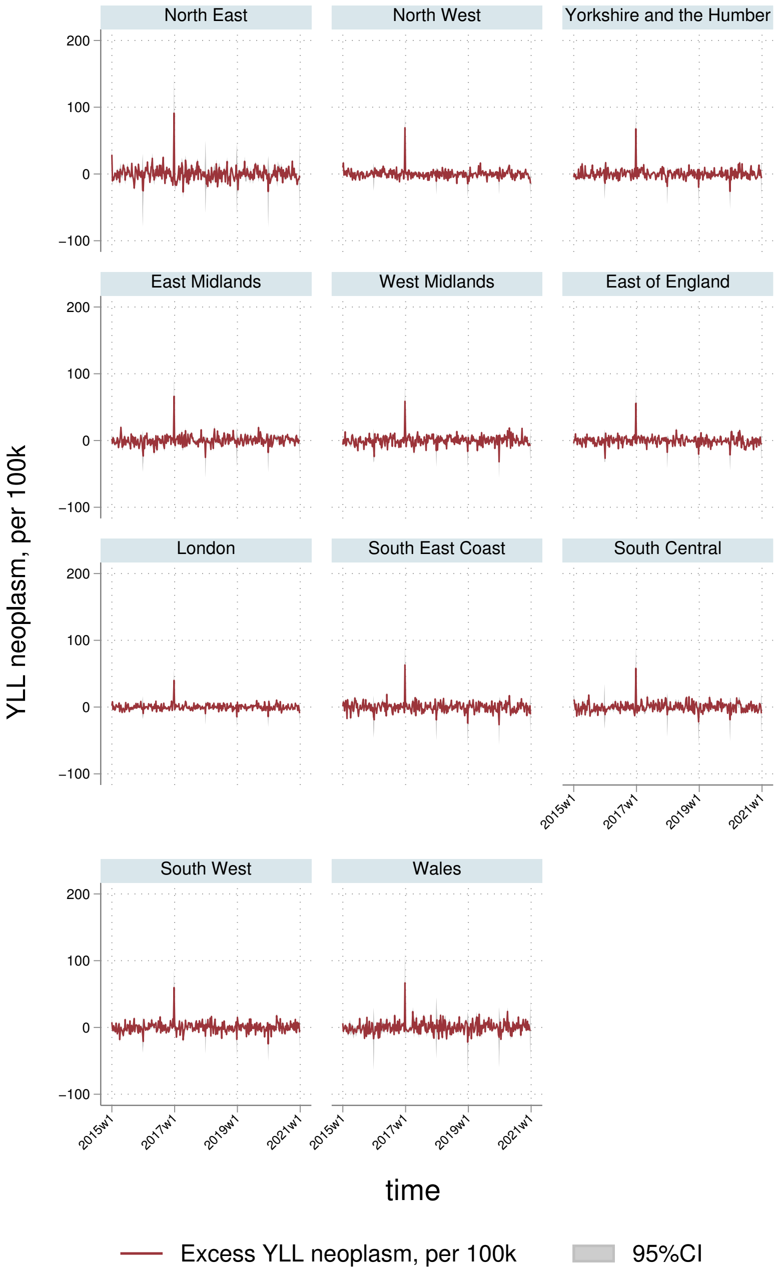

Figure 238: Excess Years of Life Lost trend per 100,000 population, cancer deaths by region, 2019-2020

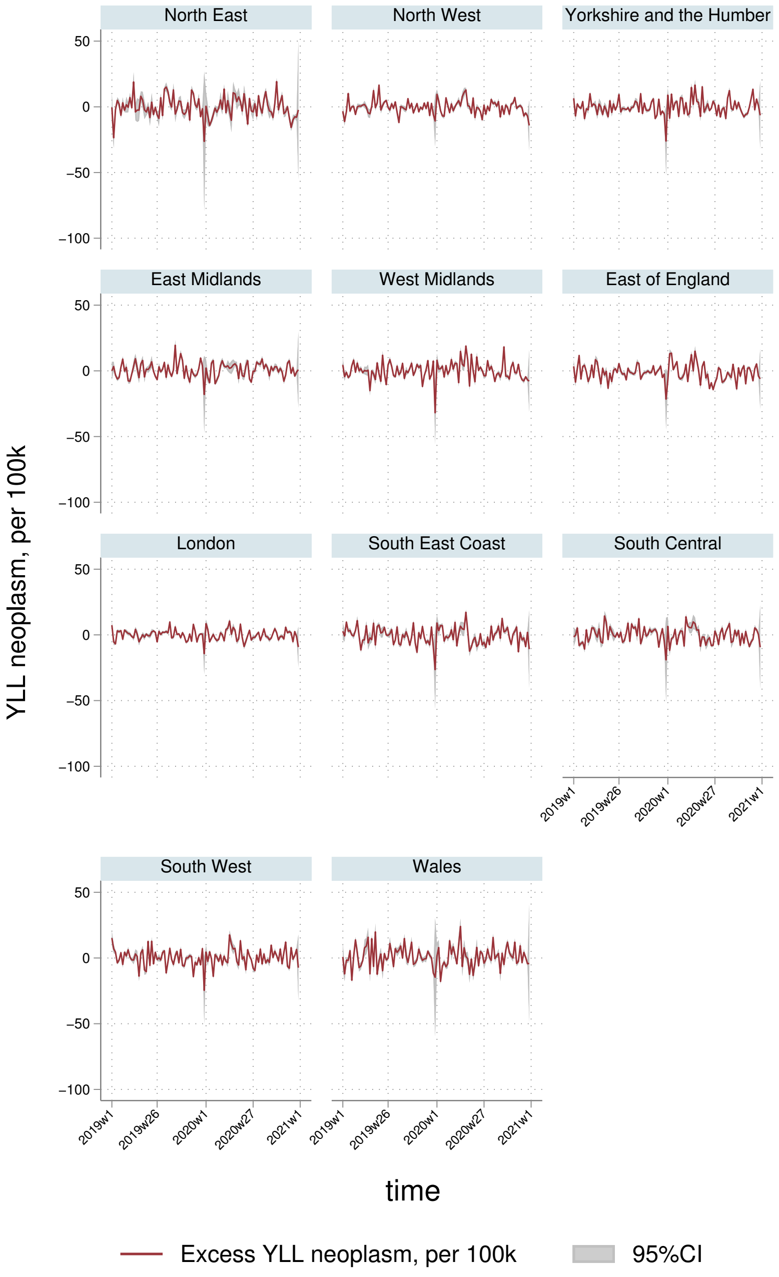

Figure 239: Observed vs Predicted Years of Life Lost trends per 100,000 population, cancer deaths by region, 2015-2020

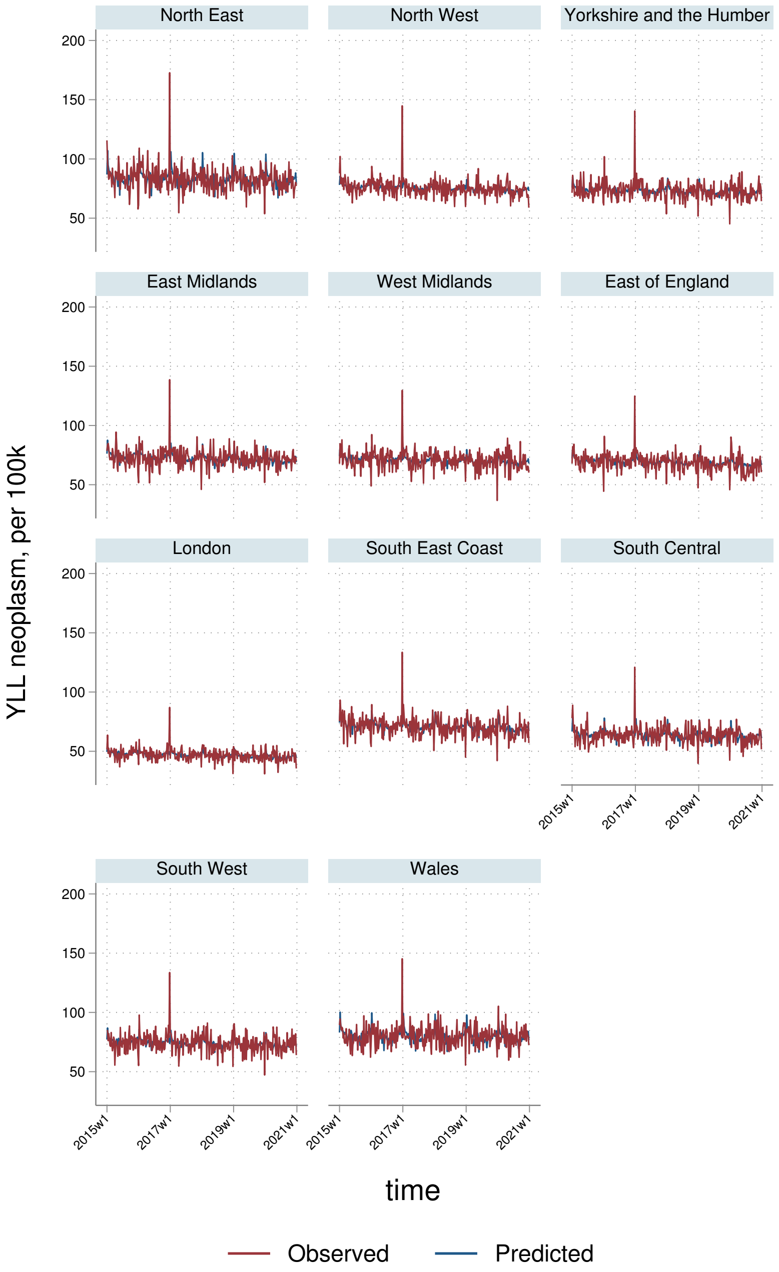

Figure 240: Observed vs Predicted Years of Life Lost trends per 100, 000 population, cancer deaths by region, 2019-2020

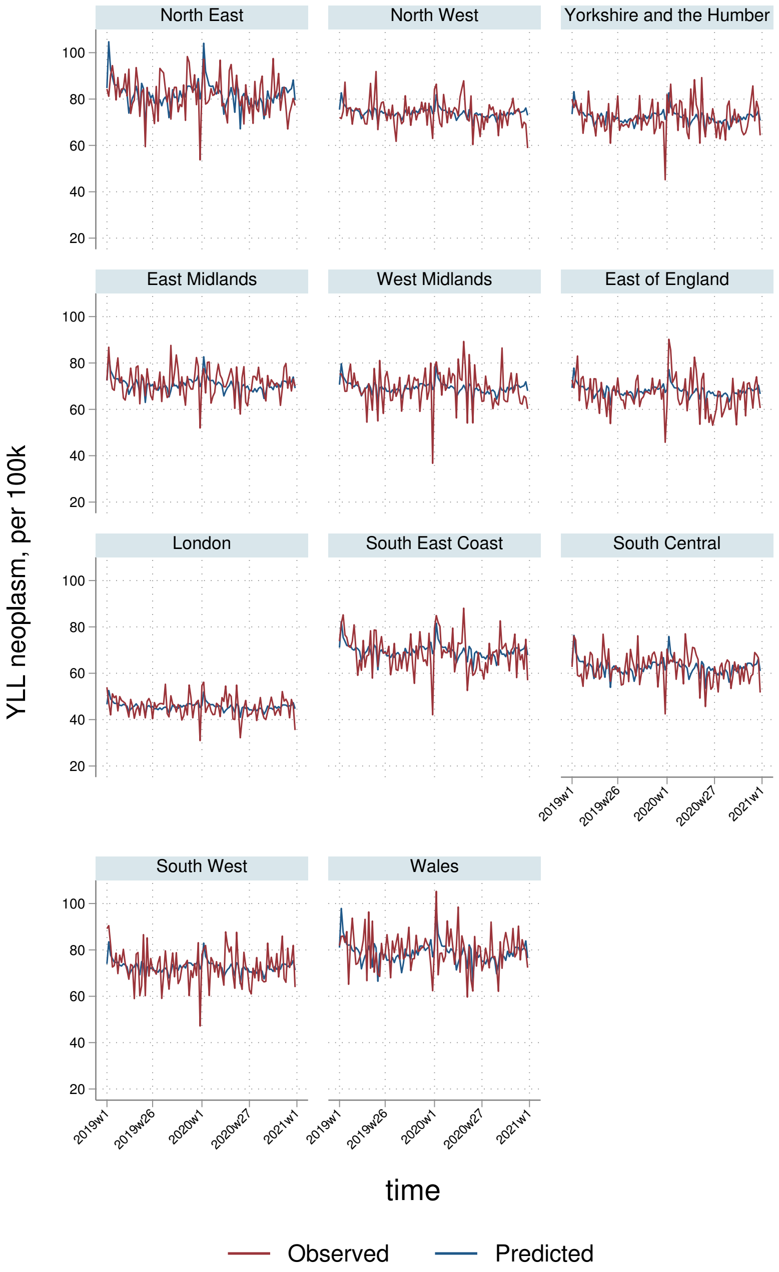

9 All other indirect

9.1 AASMRs

Figure 241: Age-standardised mortality trend, all other indirect deaths, 2015-2020

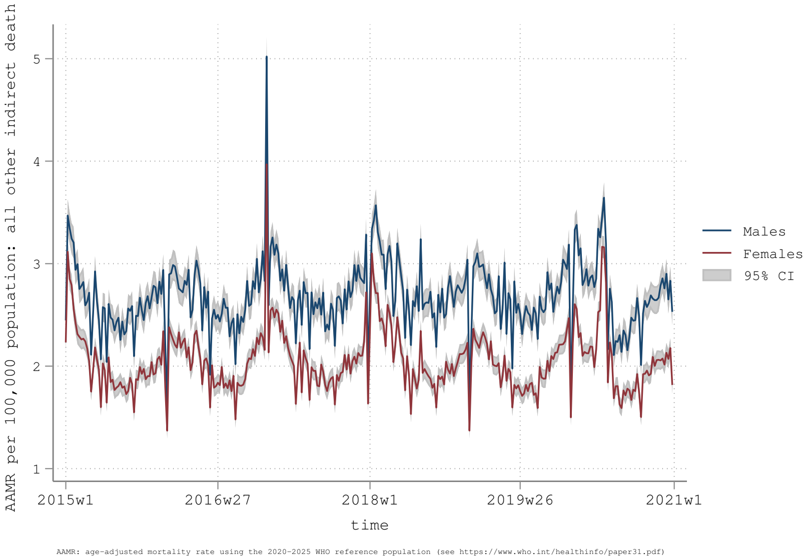

Figure 242: Age-standardised mortality trend, all other indirect deaths, 2019-2020

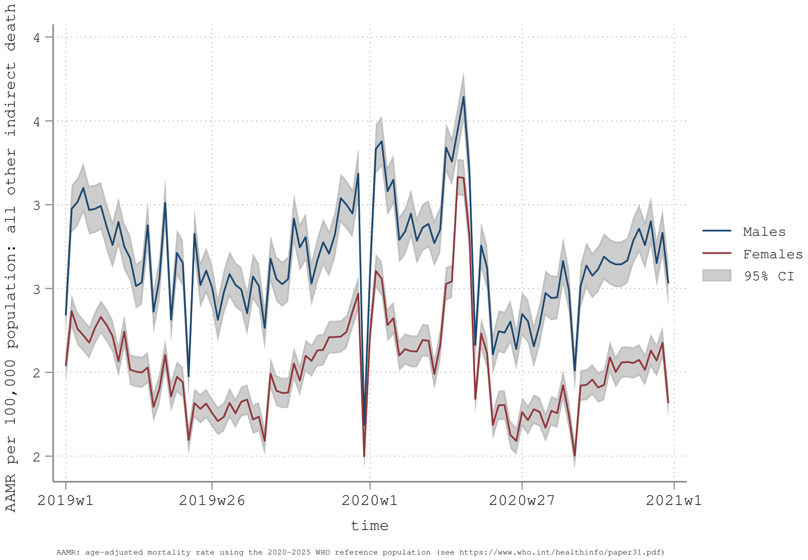

Figure 243: Age-standardised mortality trend, all other indirect deaths by deprivation quintile, 2015-2020

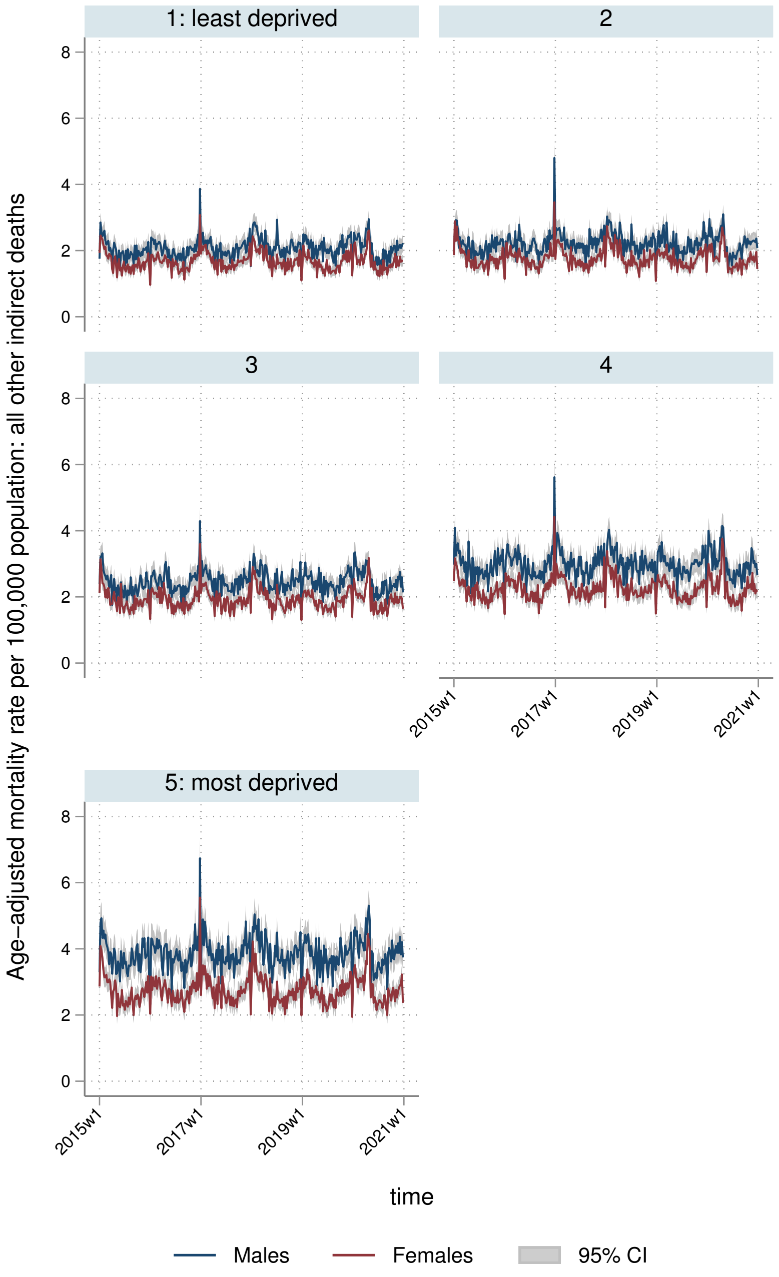

Figure 244: Age-standardised mortality trend, all other indirect deaths by deprivation quintile, 2019-2020

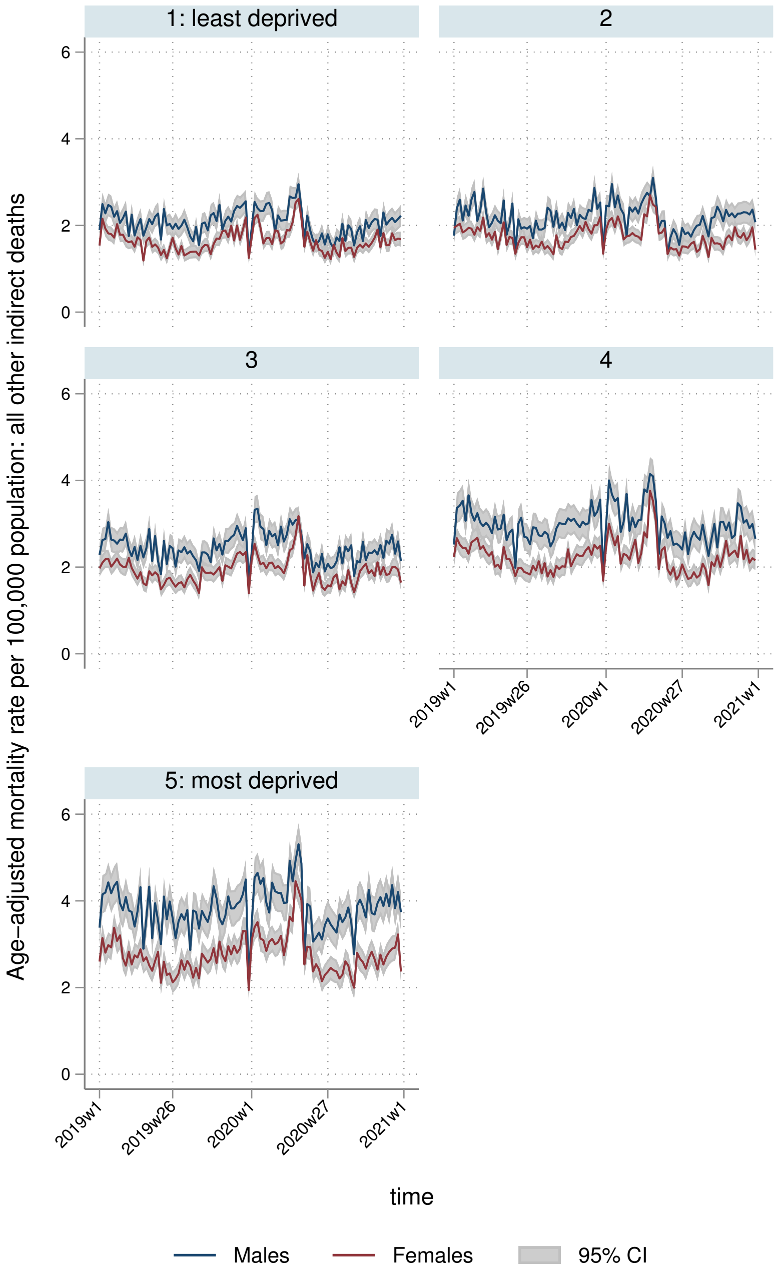

Figure 245: Age-standardised mortality trend, all other indirect deaths by region, 2015-2020

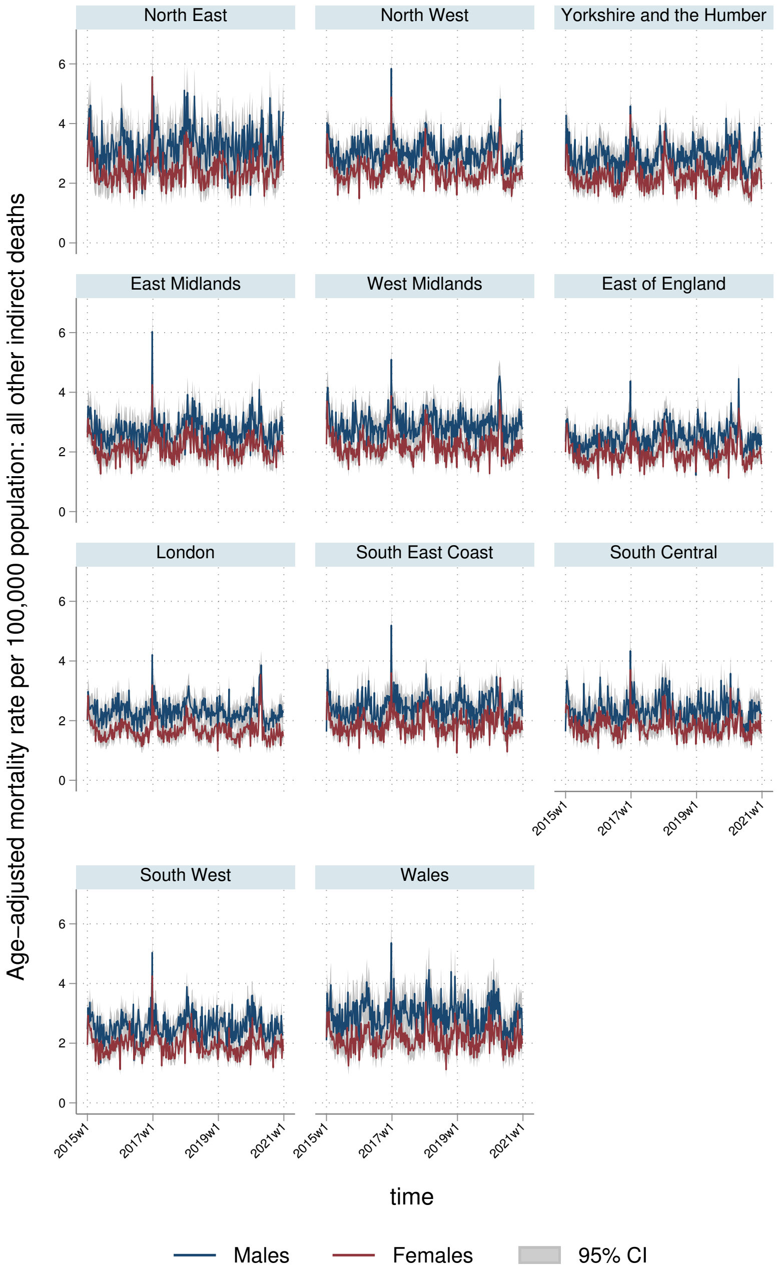

9.2 YLLs

9.2.1 England-Wales aggregate

Figure 246: Age-standardised mortality trend, all other indirect deaths by region, 2019-2020

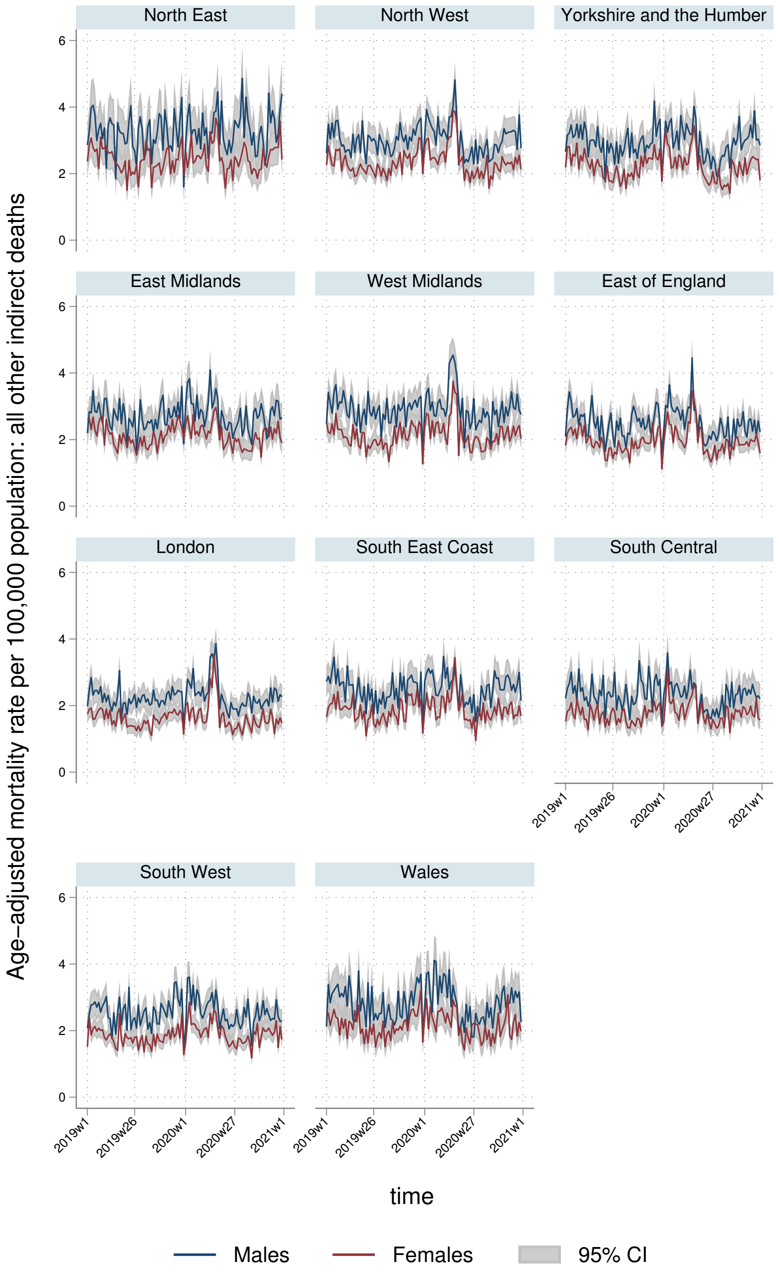

Figure 247: Years of Life Lost trend, all other indirect deaths, 2015-2020

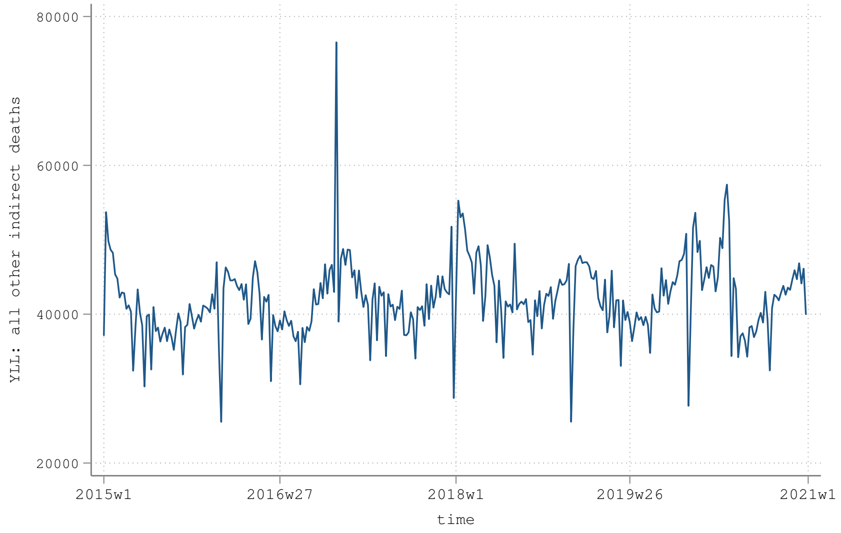

Figure 248: Years of Life Lost trend, all other indirect deaths, 2019-2020

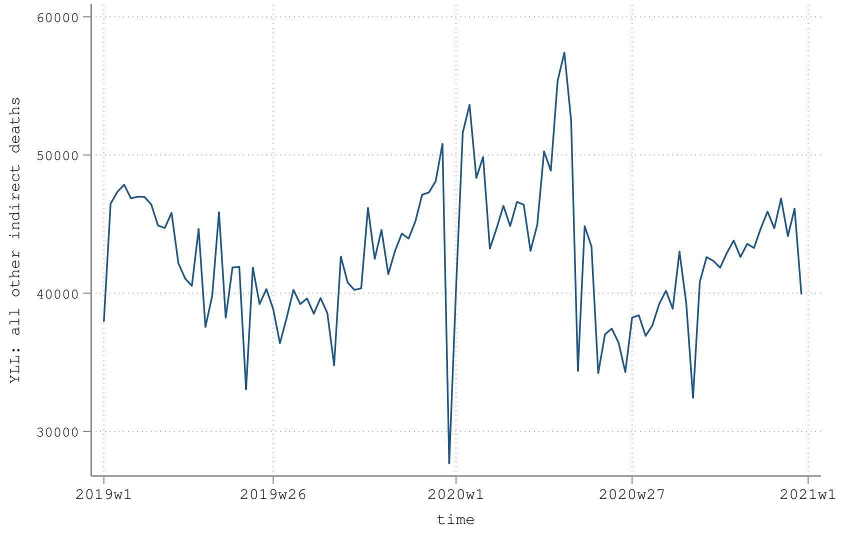

Figure 249: Excess Years of Life Lost trend, all other indirect deaths, 2015-2020

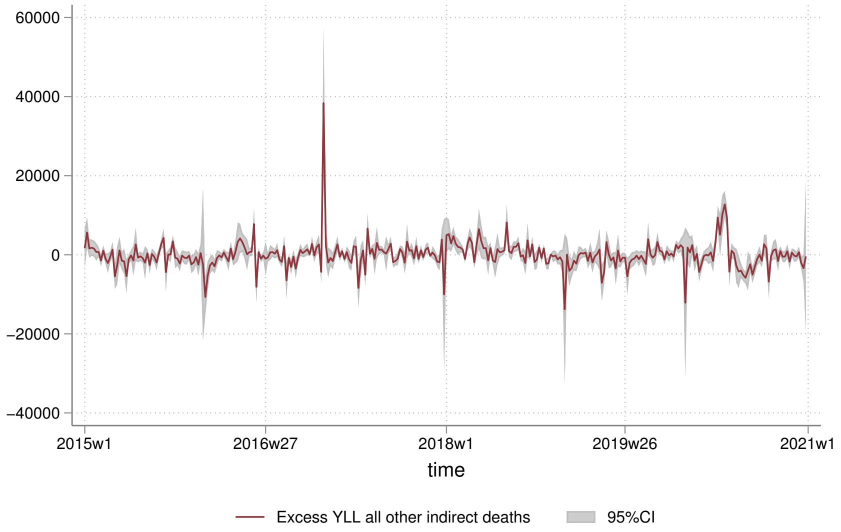

Figure 250: Excess Years of Life Lost trend, all other indirect deaths, 2019-2020

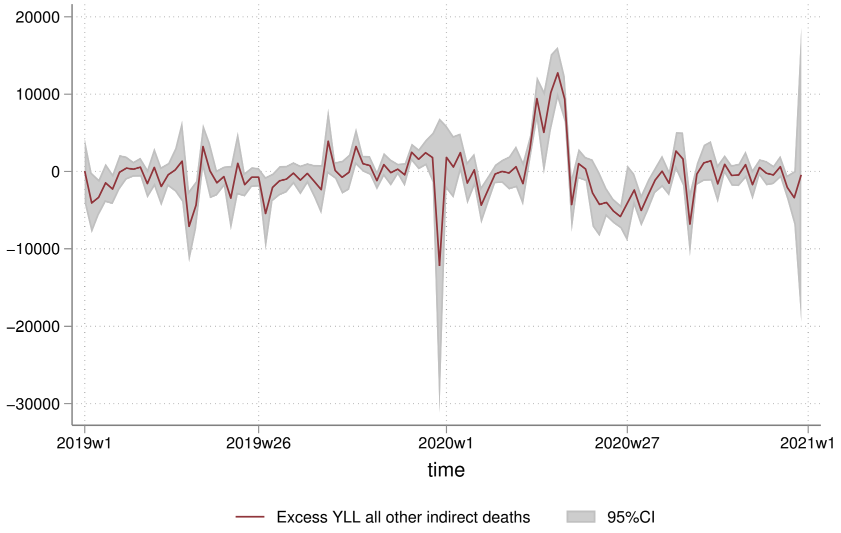

Figure 251: Observed vs Predicted Years of Life Lost trends, all other indirect deaths, 2015-2020

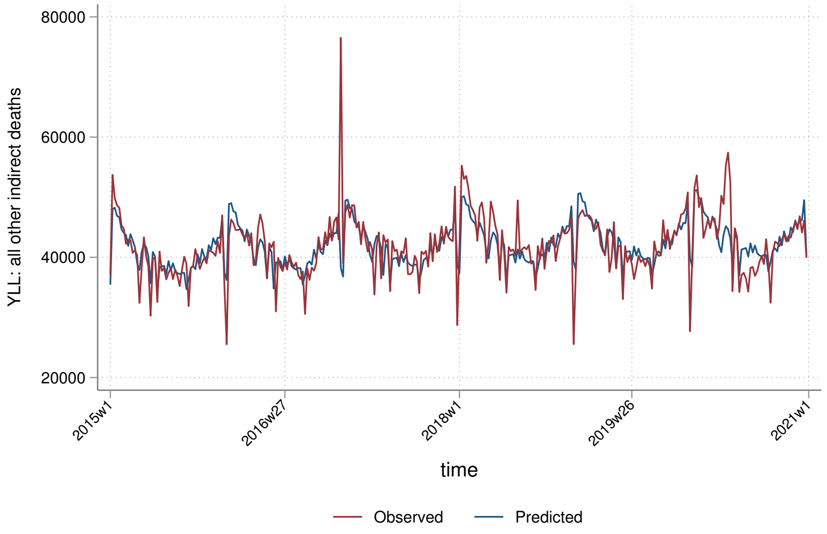

Figure 252: Observed vs Predicted Years of Life Lost trends, all other indirect deaths, 2019-2020

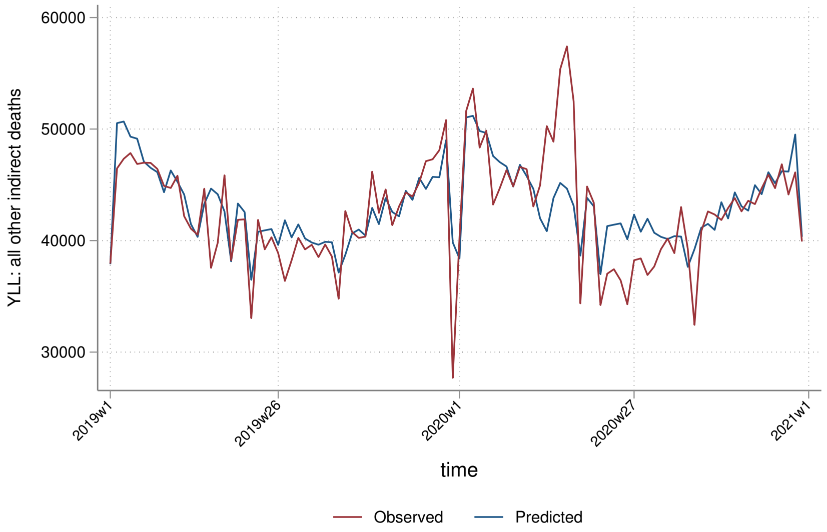

9.2.2 By sex

Figure 253: Years of Life Lost trend, all other indirect deaths by sex, 2015-2020

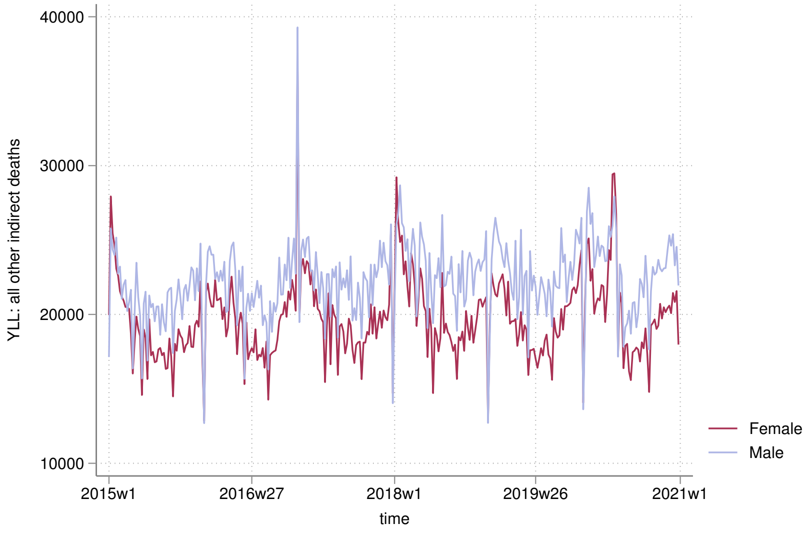

Figure 254: Years of Life Lost trend, all other indirect deaths by sex, 2019-2020

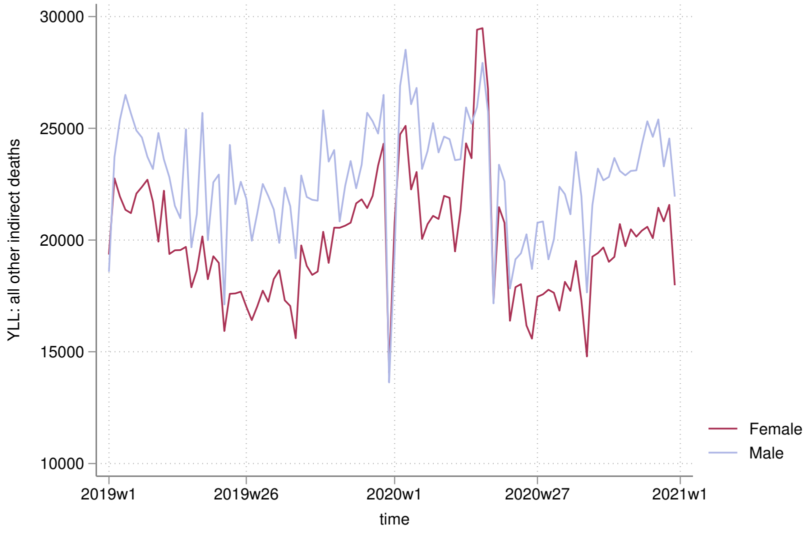

Figure 255: Excess Years of Life Lost trend, all other indirect deaths by sex, 2015-2020

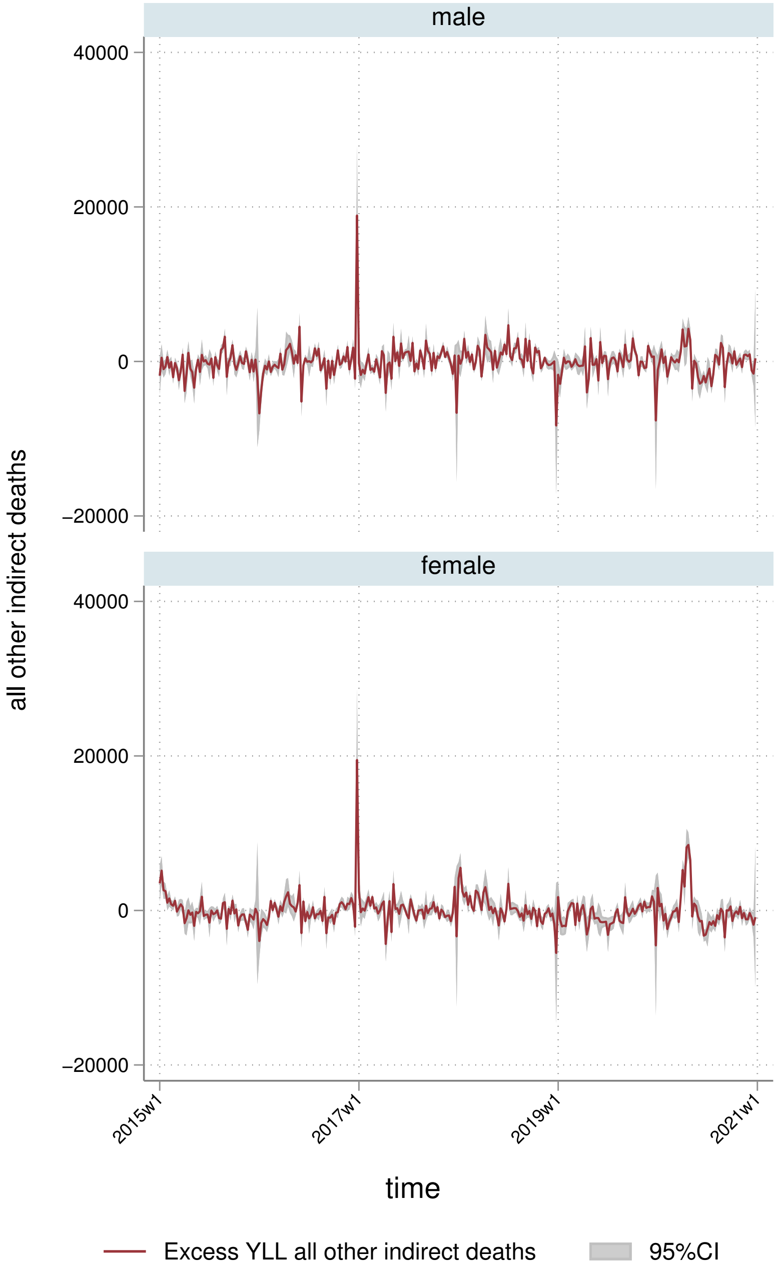

Figure 256: Excess Years of Life Lost trend, all other indirect deaths by sex, 2019-2020

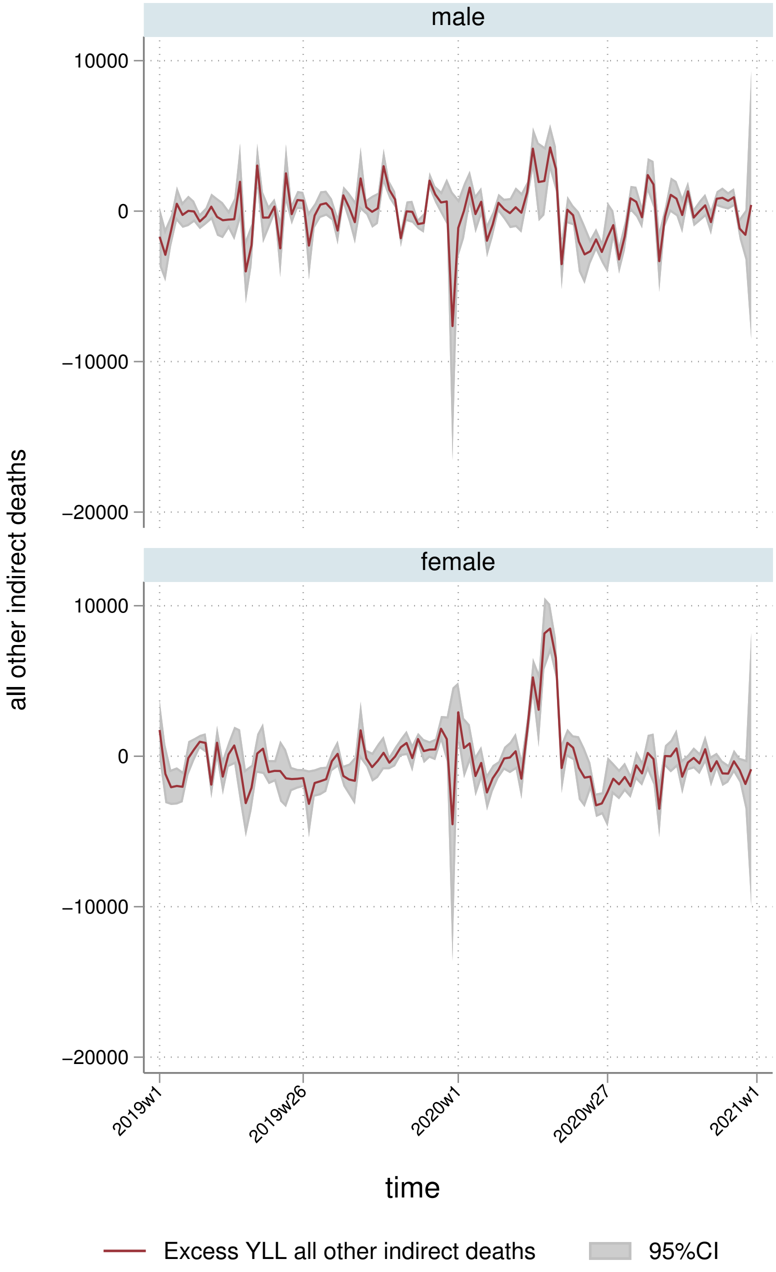

Figure 257: Observed vs Predicted Years of Life Lost trends, all other indirect deaths by sex, 2015-2020

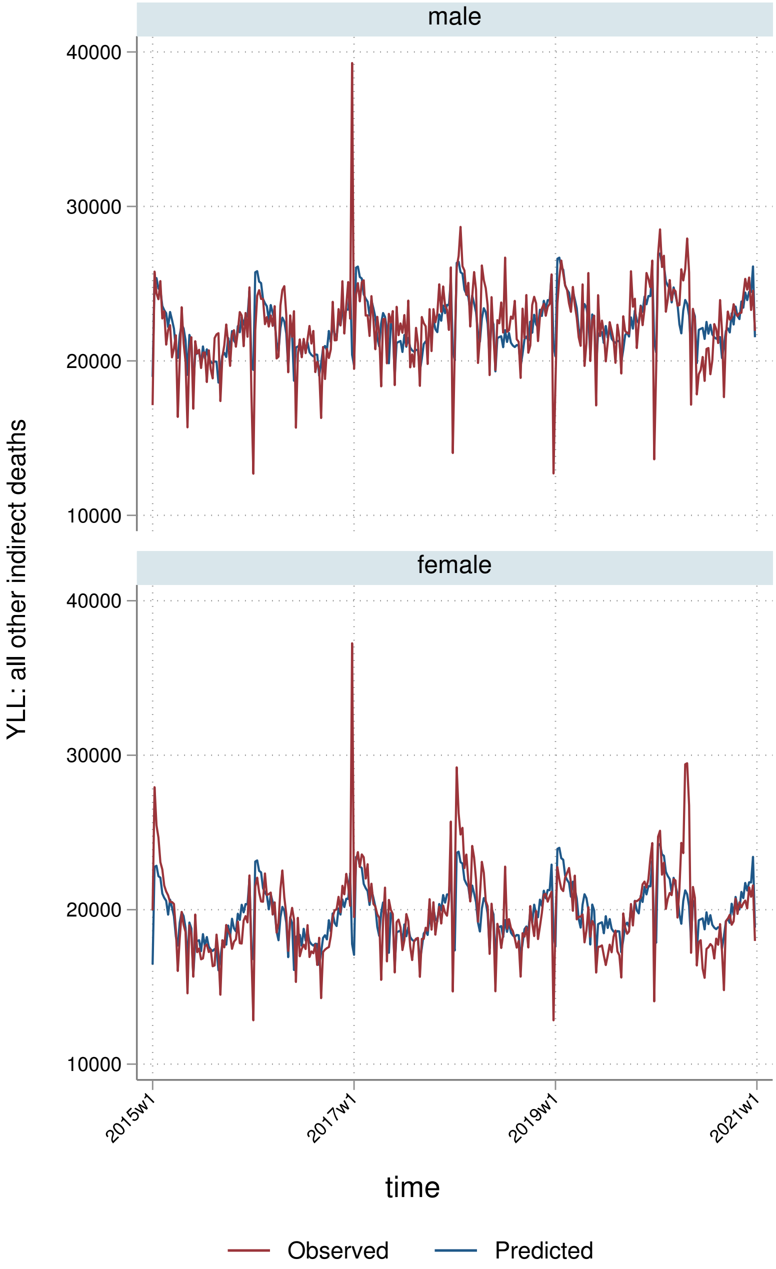

Figure 258: Observed vs Predicted Years of Life Lost trends, all other indirect deaths by sex, 2019-2020

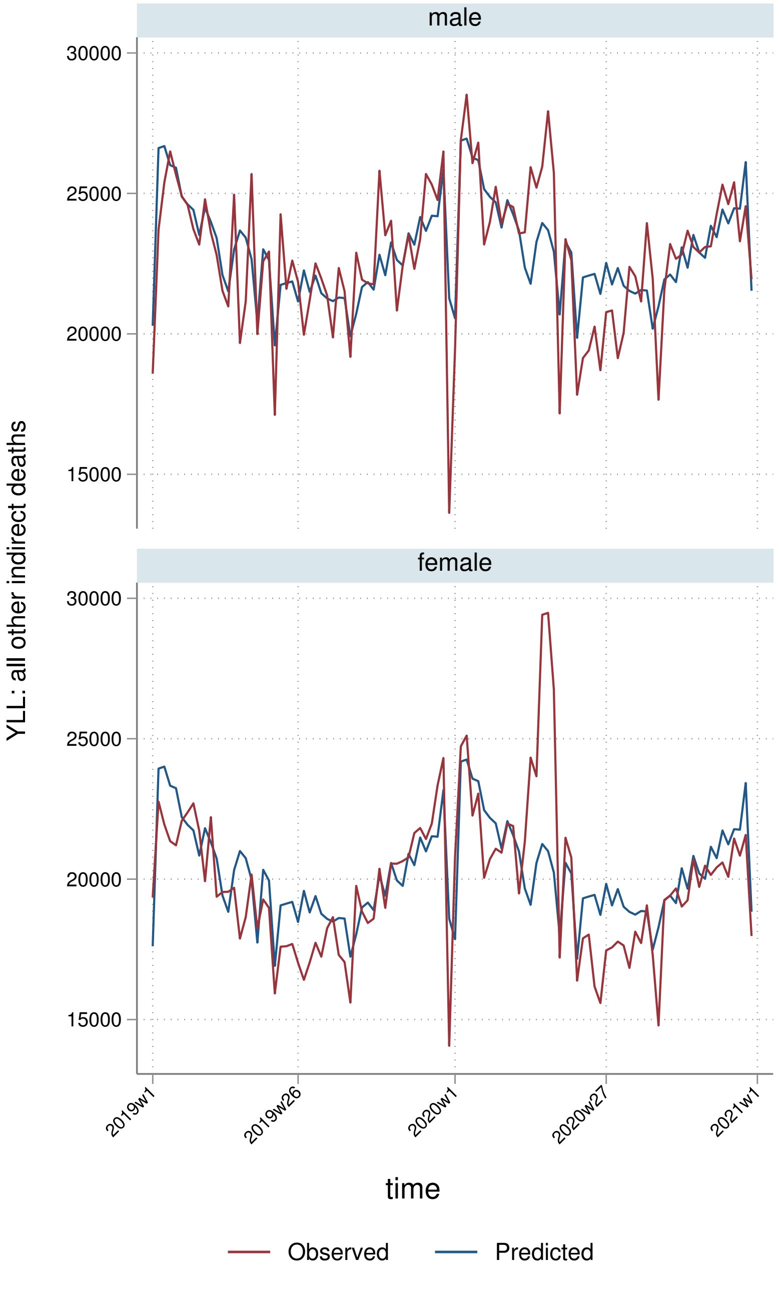

9.2.3 By deprivation quintile

Figure 259: Years of Life Lost trend, all other indirect deaths by deprivation quintile, 2015-2020

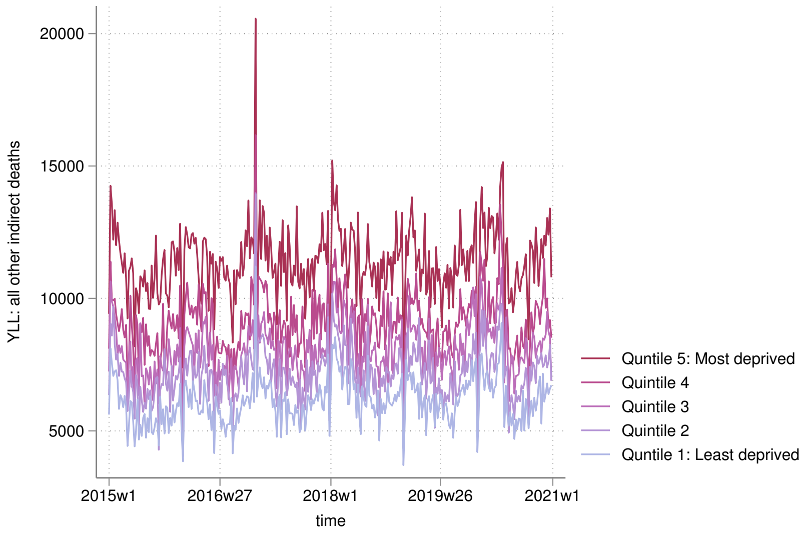

Figure 260: Years of Life Lost trend, all other indirect deaths by deprivation quintile, 2019-2020

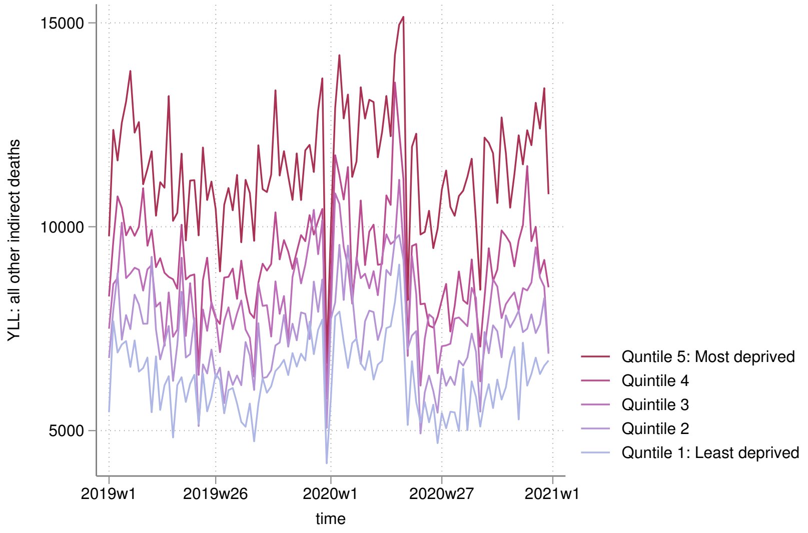

Figure 261: Excess Years of Life Lost trend, all other indirect deaths by deprivation quintile, 2015-2020

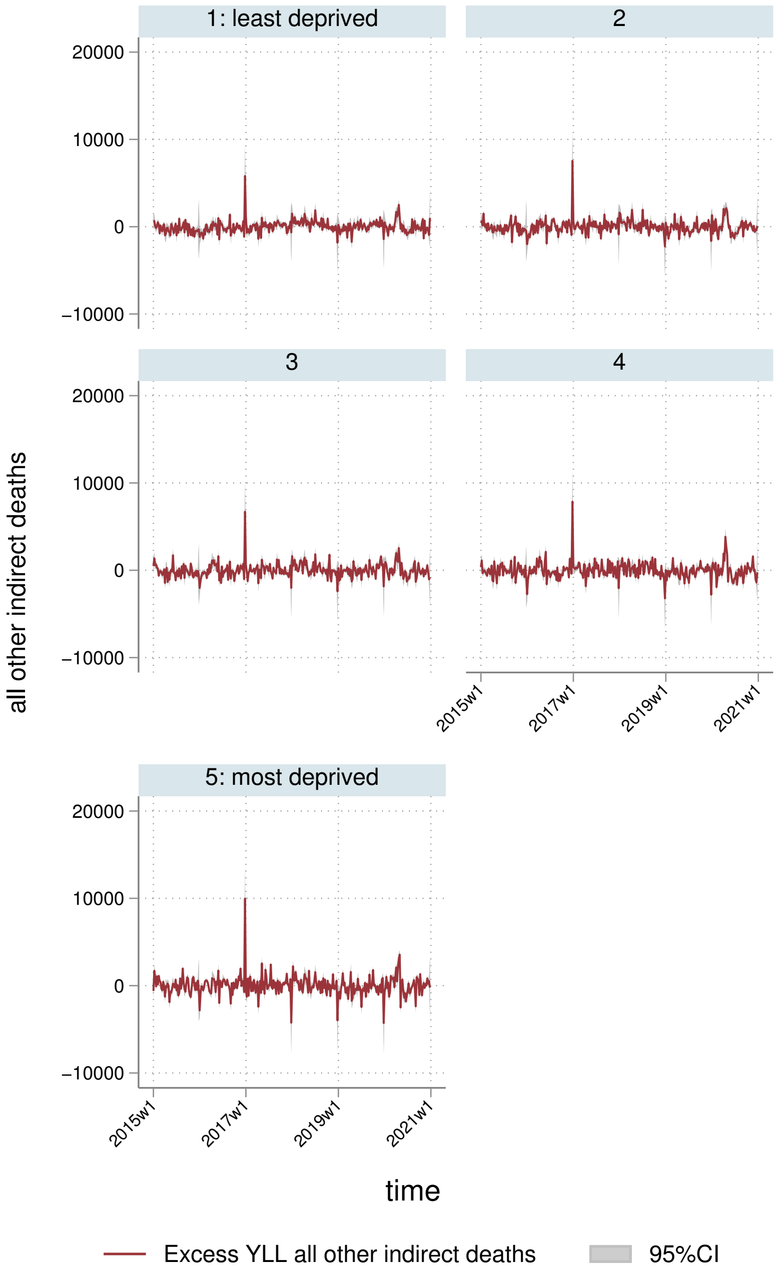

Figure 262: Excess Years of Life Lost trend, all other indirect deaths by deprivation quintile, 2019-2020

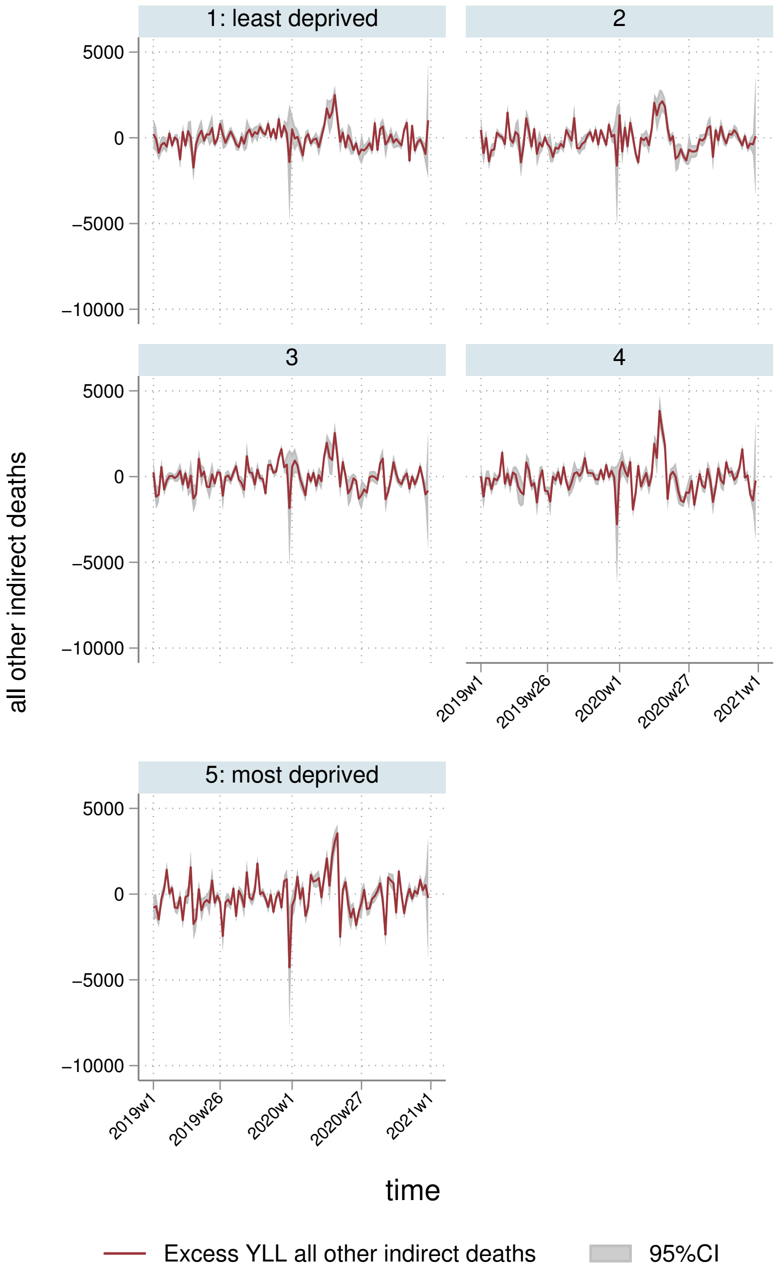

Figure 263: Observed vs Predicted Years of Life Lost trends, all other indirect deaths by deprivation quintile, 2015-2020

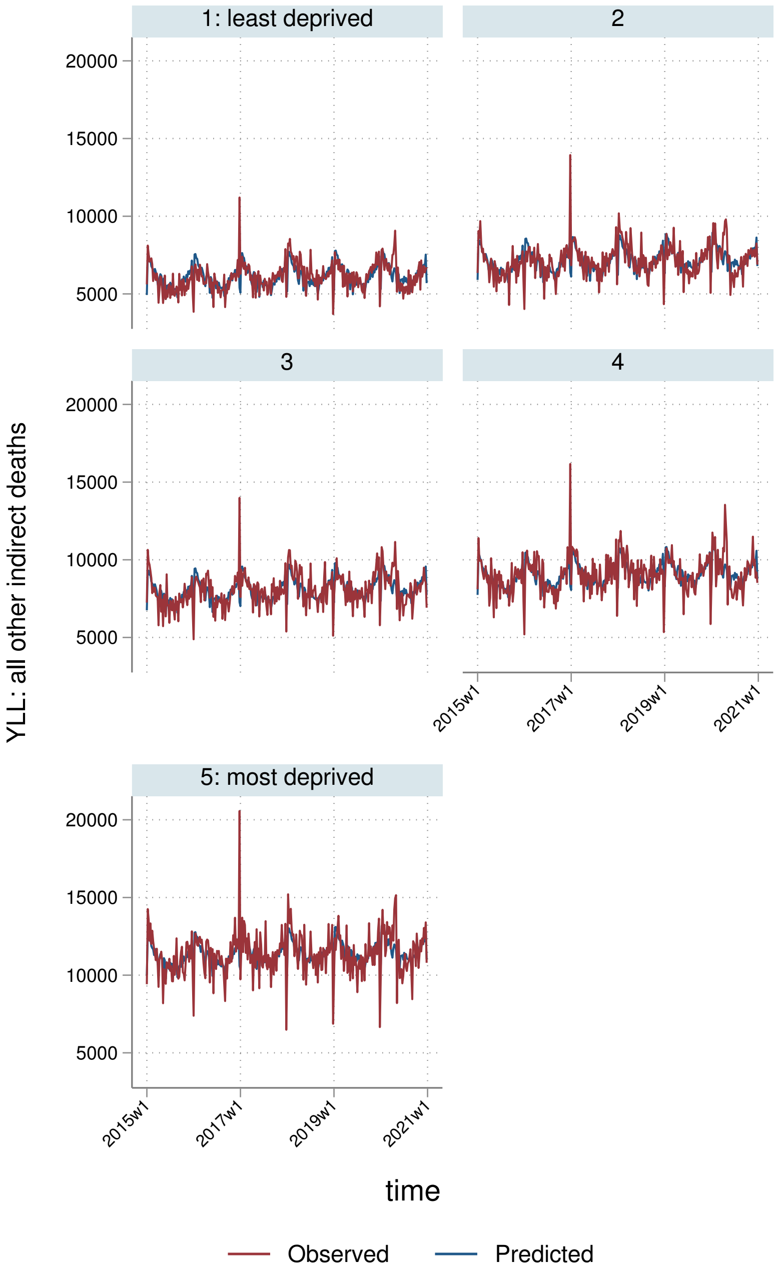

Figure 264: Observed vs Predicted Years of Life Lost trends, all other indirect deaths by deprivation quintile, 2019-2020

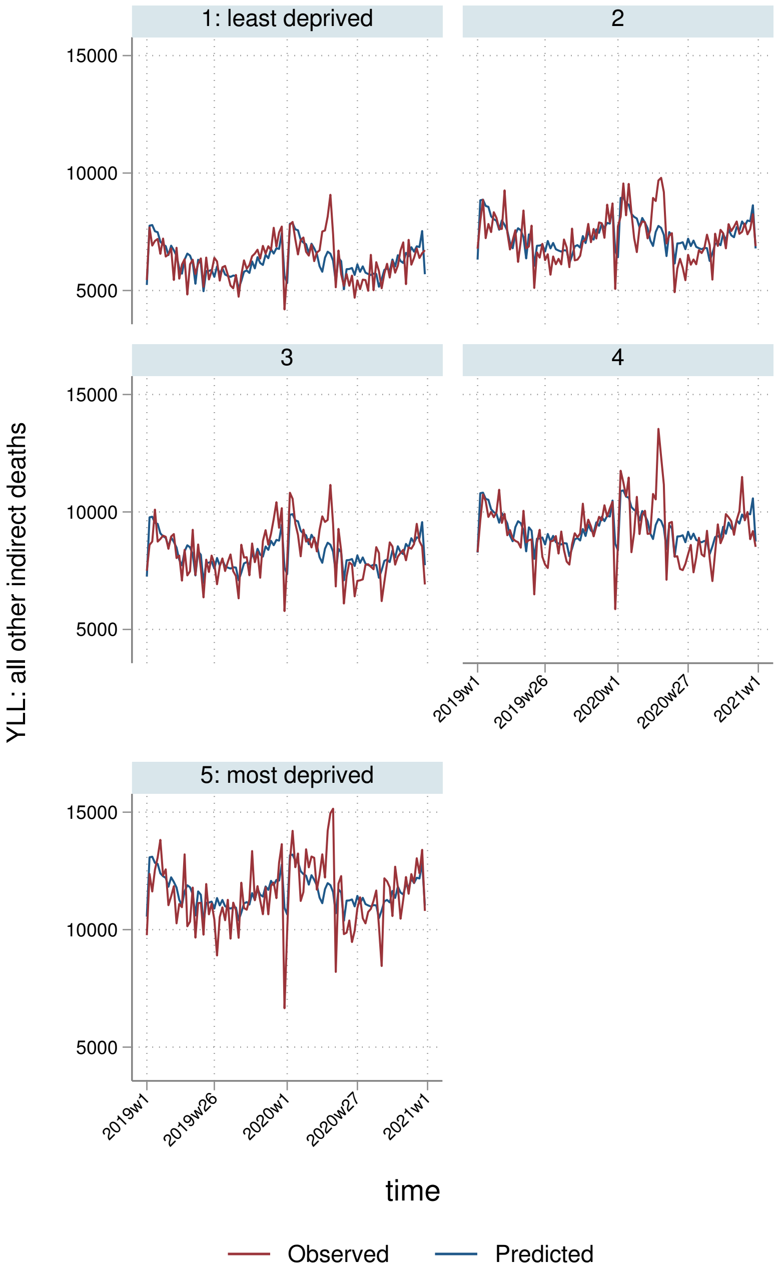

9.2.4 By Strategic Health Authority

Figure 265: Years of Life Lost trend, all other indirect deaths by region, 2015-2020

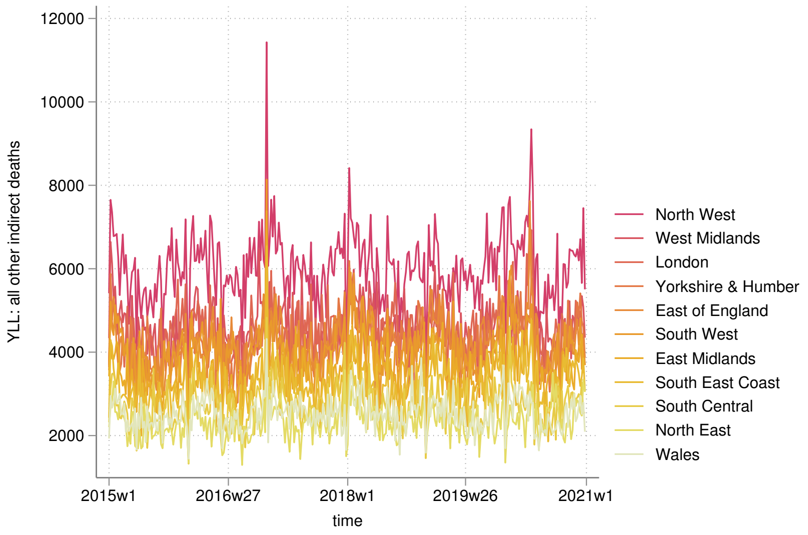

Figure 266: Years of Life Lost trend, all other indirect deaths by region, 2019-2020

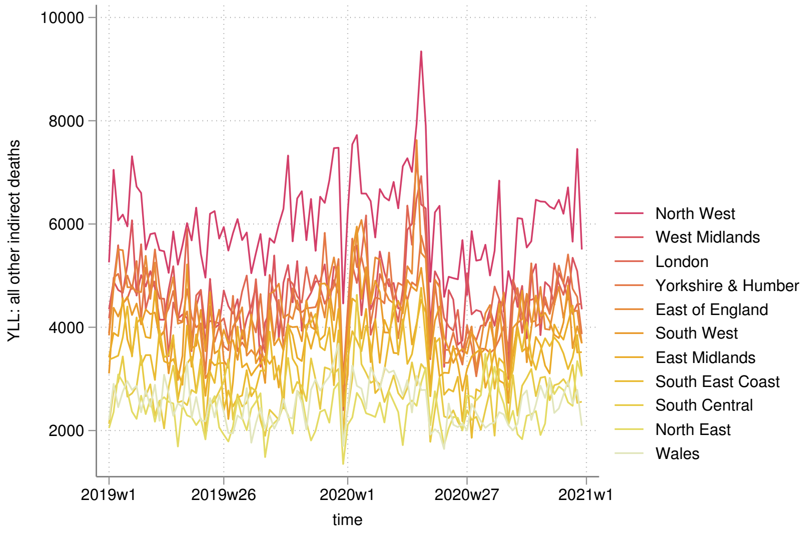

Figure 267: Excess Years of Life Lost trend, all other indirect deaths by region, 2015-2020

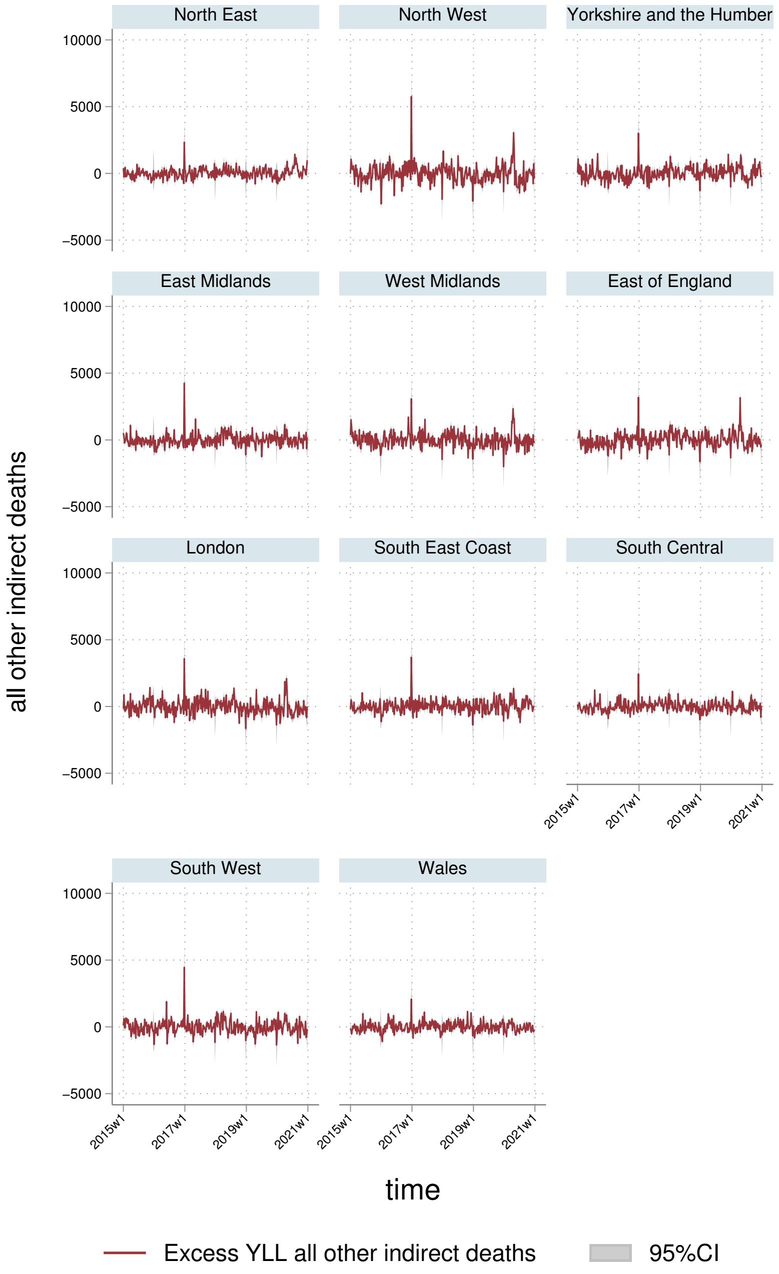

Figure 268: Excess Years of Life Lost trend, all other indirect deaths by region, 2019-2020

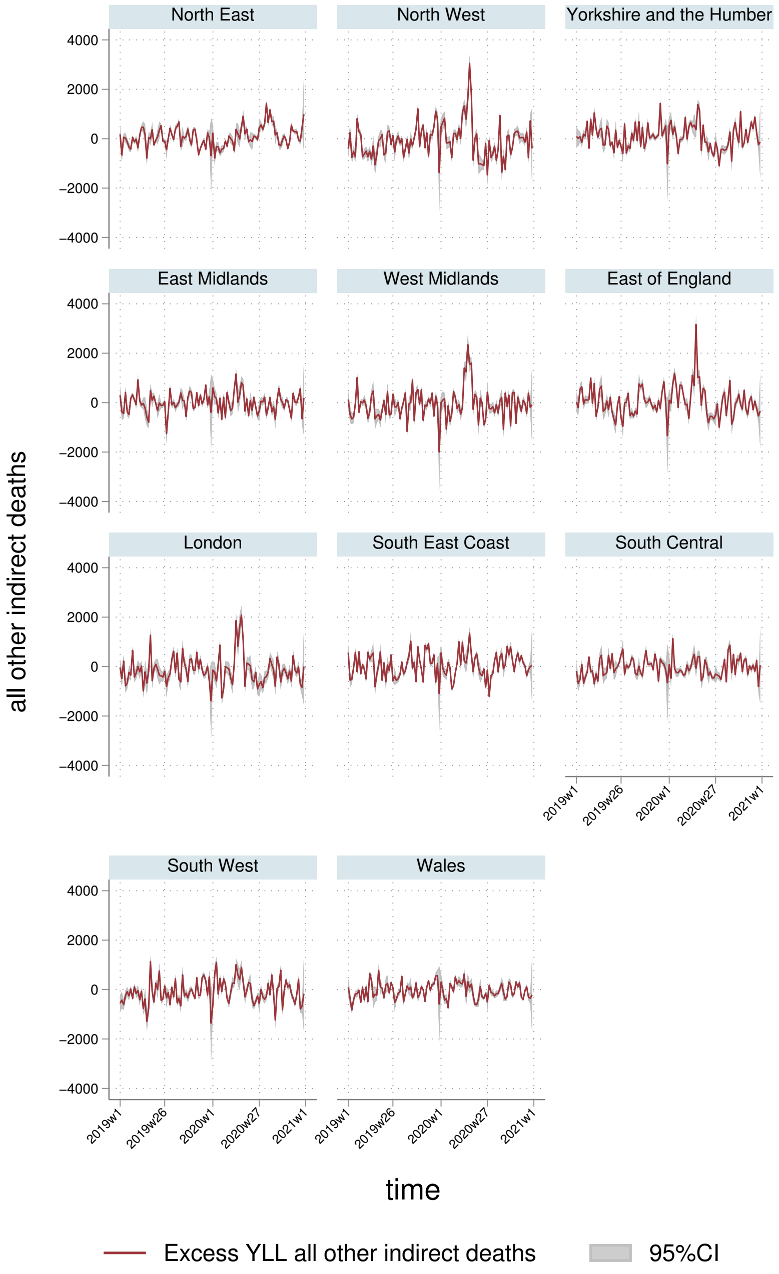

Figure 269: Observed vs Predicted Years of Life Lost trends, all other indirect deaths by region, 2015-2020

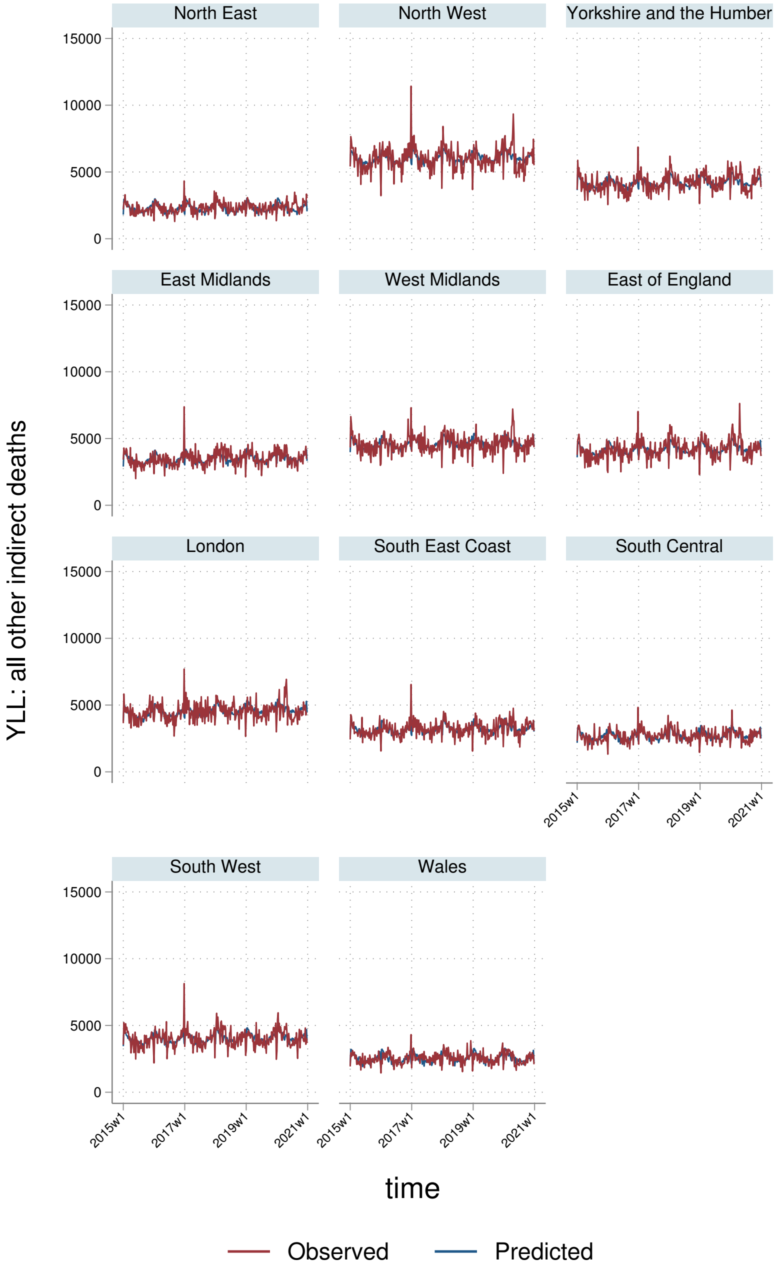

9.3 YLLs per $100,000$ population

9.3.1 England-Wales aggregate

Figure 270: Observed vs Predicted Years of Life Lost trends, all other indirect deaths by region, 2019-2020

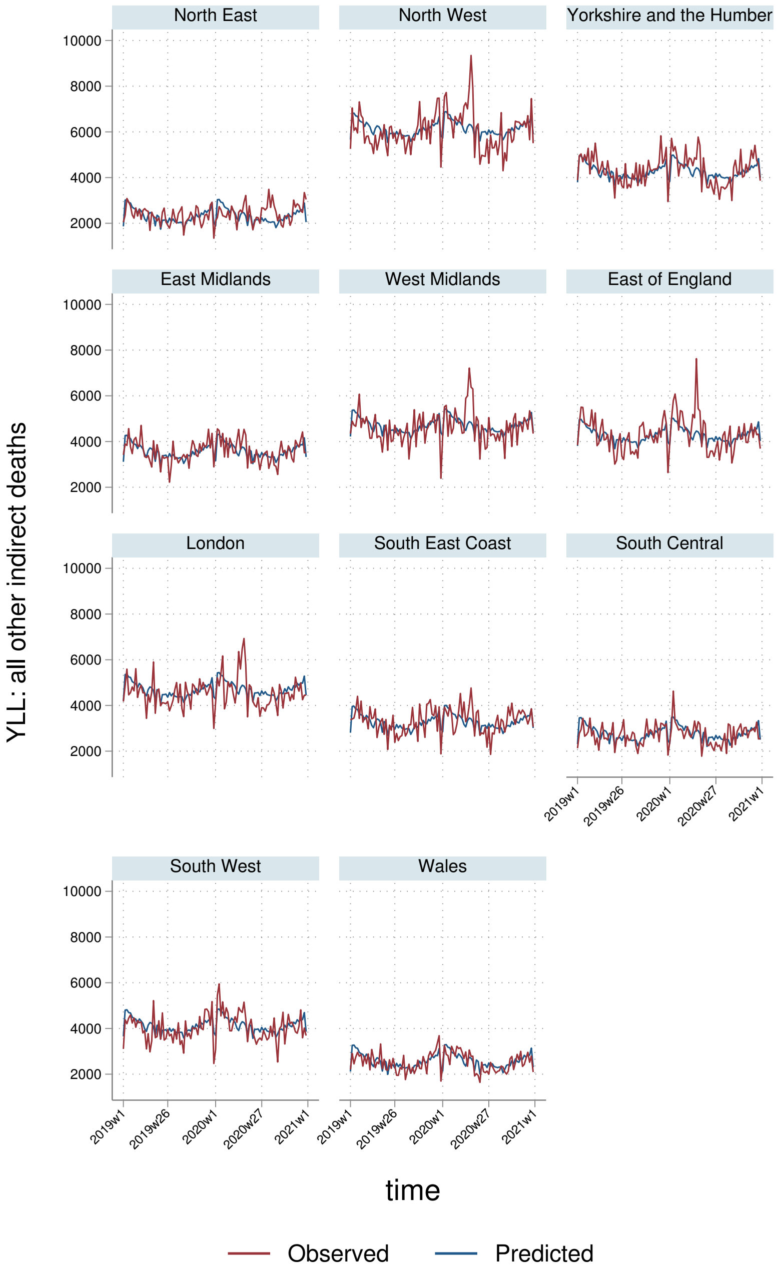

Figure 271: Years of Life Lost trend per 100,000 population, all other indirect deaths, 2015-2020

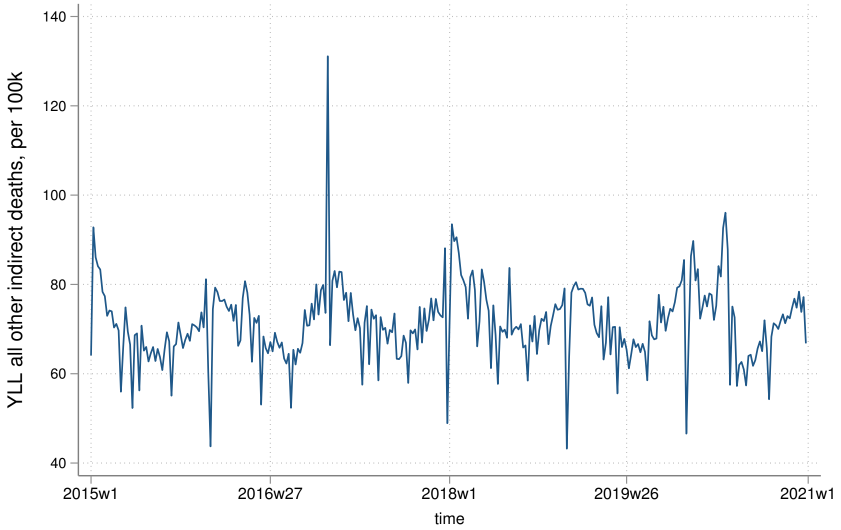

Figure 272: Years of Life Lost trend per 100,000 population, all other indirect deaths, 2019-2020

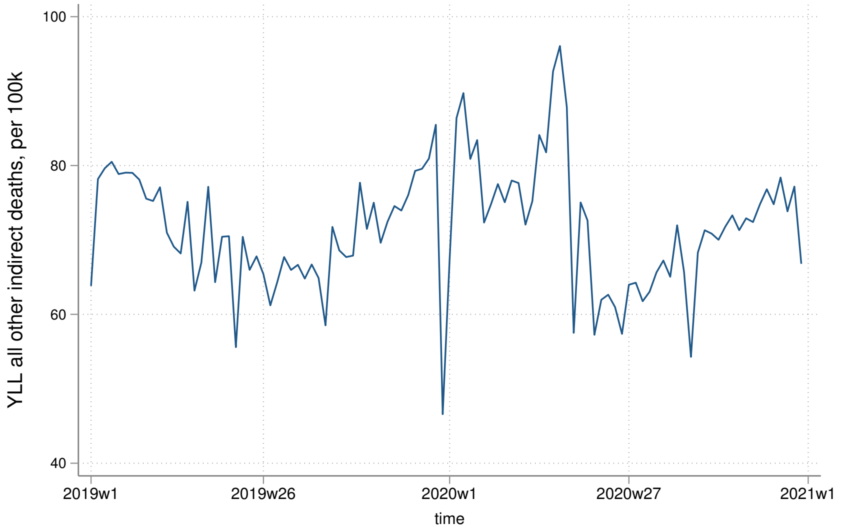

Figure 273: Excess Years of Life Lost trend per 100, 000 population, all other indirect deaths, 2015-2020

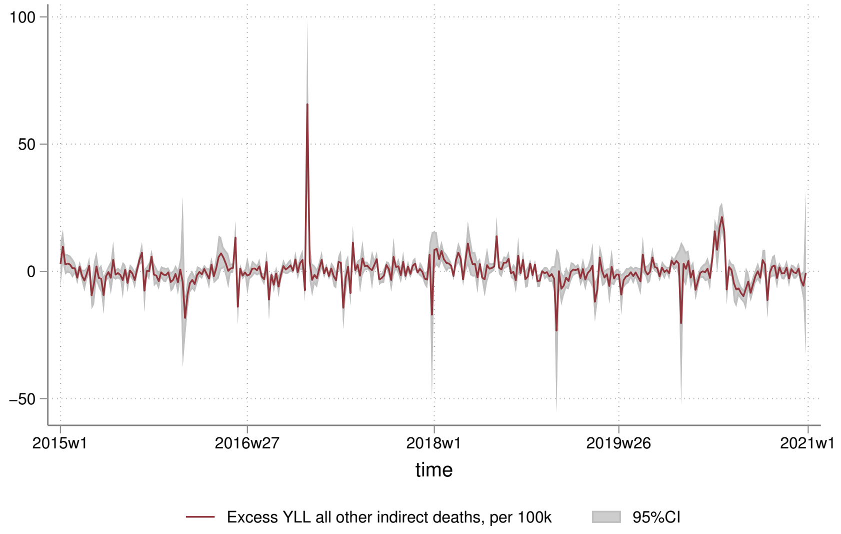

Figure 274: Excess Years of Life Lost trend per 100, 000 population, all other indirect deaths, 2019-2020

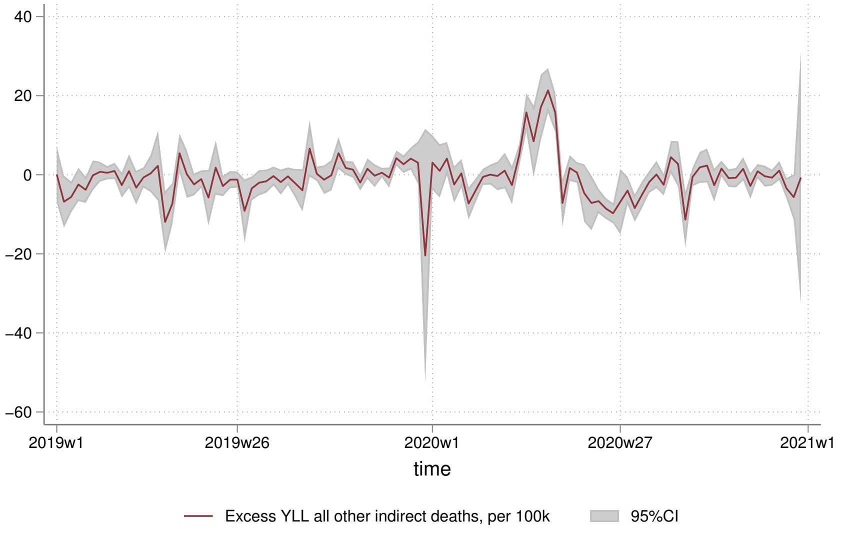

Figure 275: Observed vs Predicted Years of Life Lost trends per 100,000 population, all other indirect deaths, 2015-2020

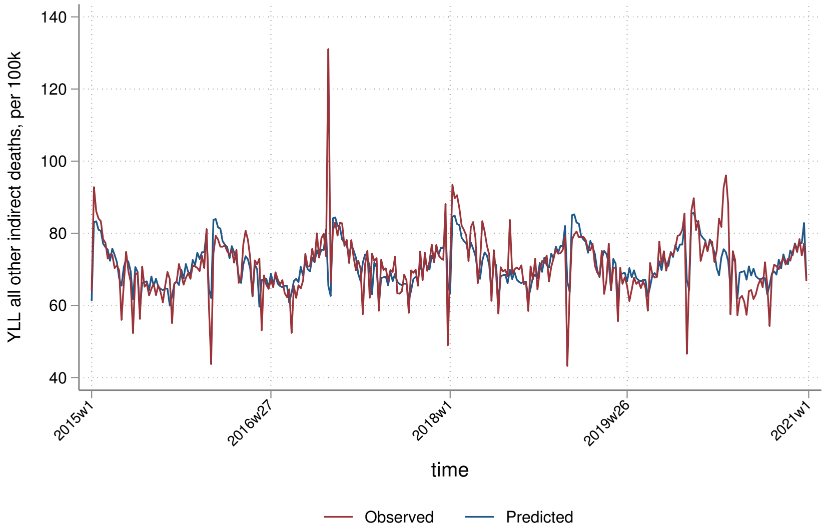

Figure 276: Observed vs Predicted Years of Life Lost trends per 100,000 population, all other indirect deaths, 2019-2020

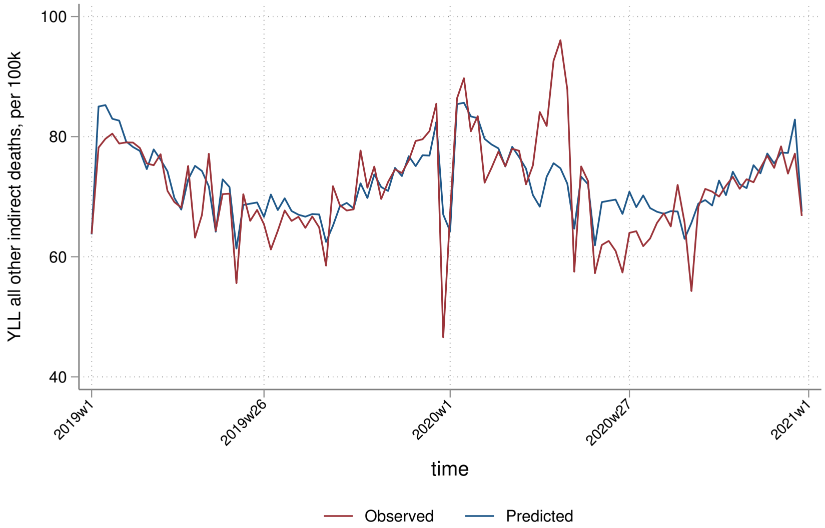

9.3.2 By sex

Figure 277: Years of Life Lost trend per 100,000 population, all other indirect deaths by sex, 2015-2020

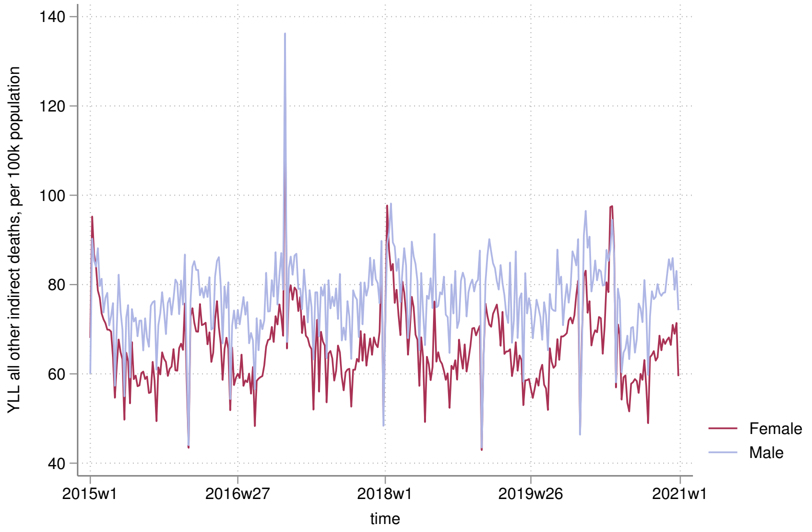

Figure 278: Years of Life Lost trend per 100,000 population, all other indirect deaths by sex, 2019-2020

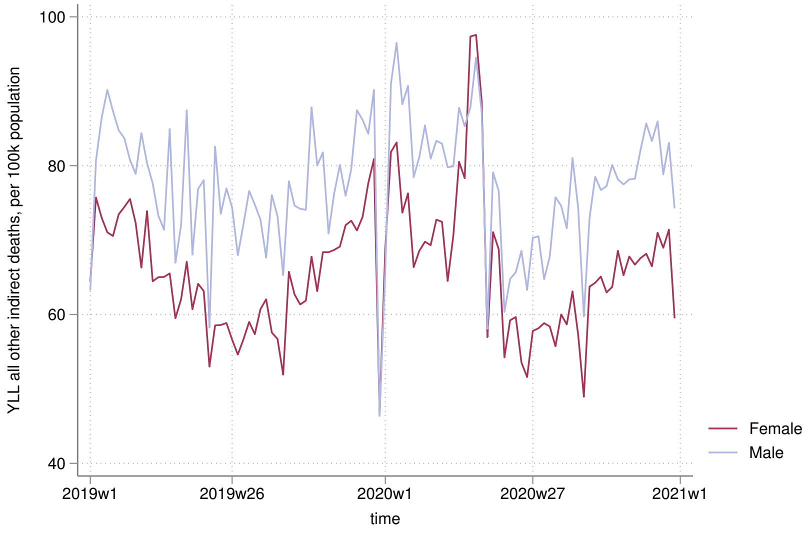

Figure 279: Excess Years of Life Lost trend per 100, 000 population, all other indirect deaths by sex, 2015-2020

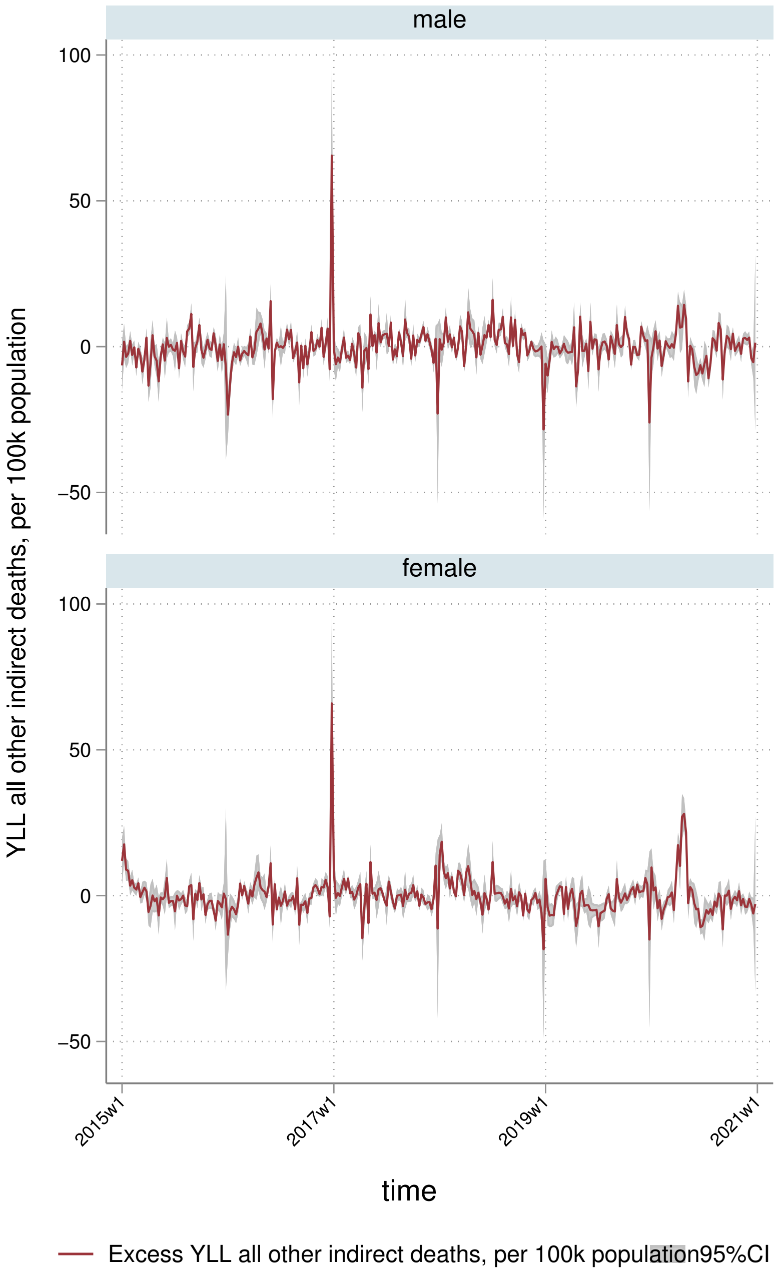

Figure 280: Excess Years of Life Lost trend per 100,000 population, all other indirect deaths by sex, 2019-2020

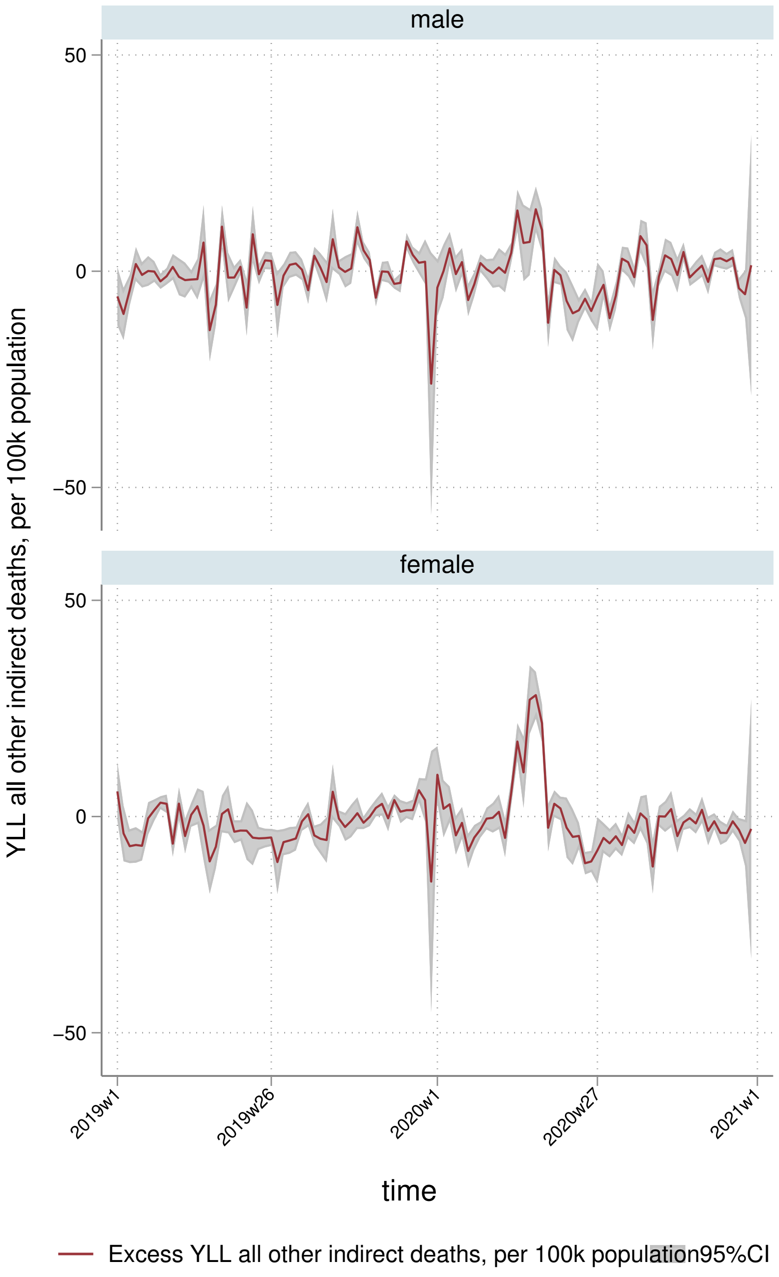

Figure 281: Observed vs Predicted Years of Life Lost trends per 100,000 population, all other indirect deaths by sex, 2015-2020

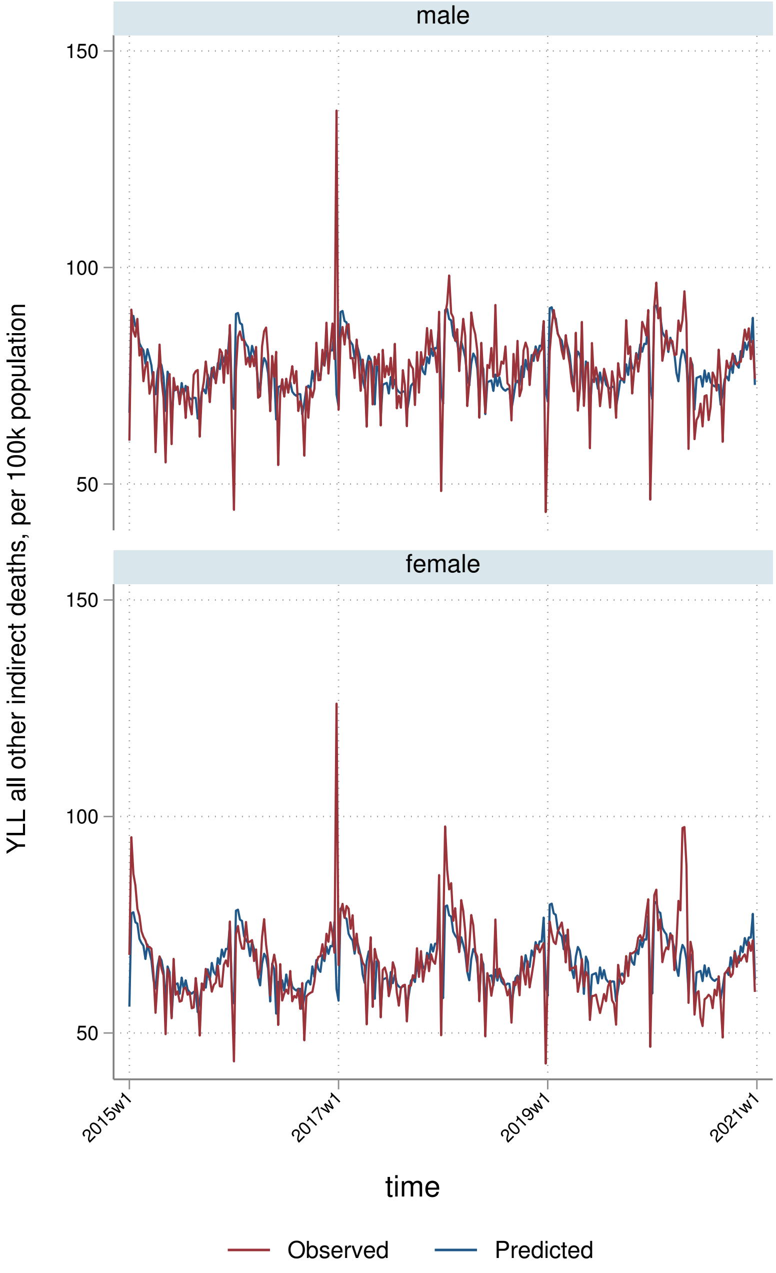

Figure 282: Observed vs Predicted Years of Life Lost trends per 100,000 population, all other indirect deaths by sex, 2019-2020

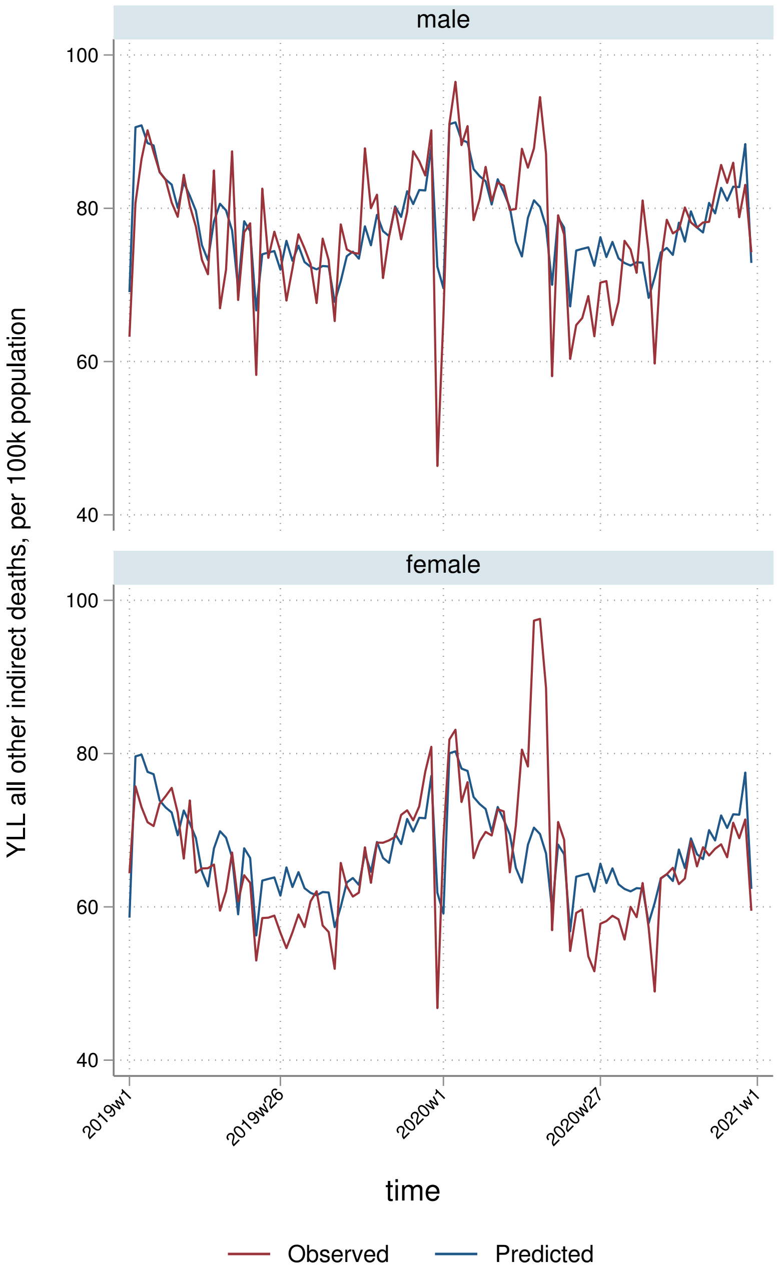

9.3.3 By deprivation quintile

Figure 283: Years of Life Lost trend per 100,000 population, all other indirect deaths by deprivation quintile, 2015-2020

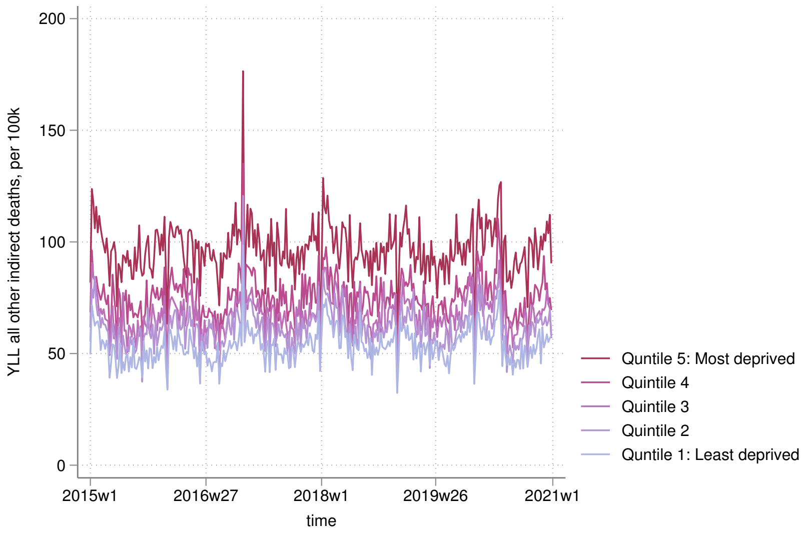

Figure 284: Years of Life Lost trend per 100,000 population, all other indirect deaths by deprivation quintile, 2019-2020

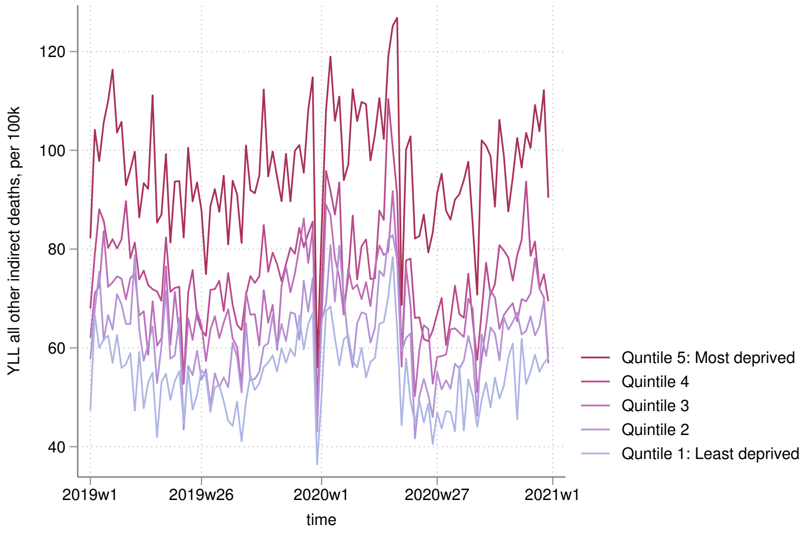

Figure 285: Excess Years of Life Lost trend per 100,000 population, all other indirect deaths by deprivation quintile, 2015-2020

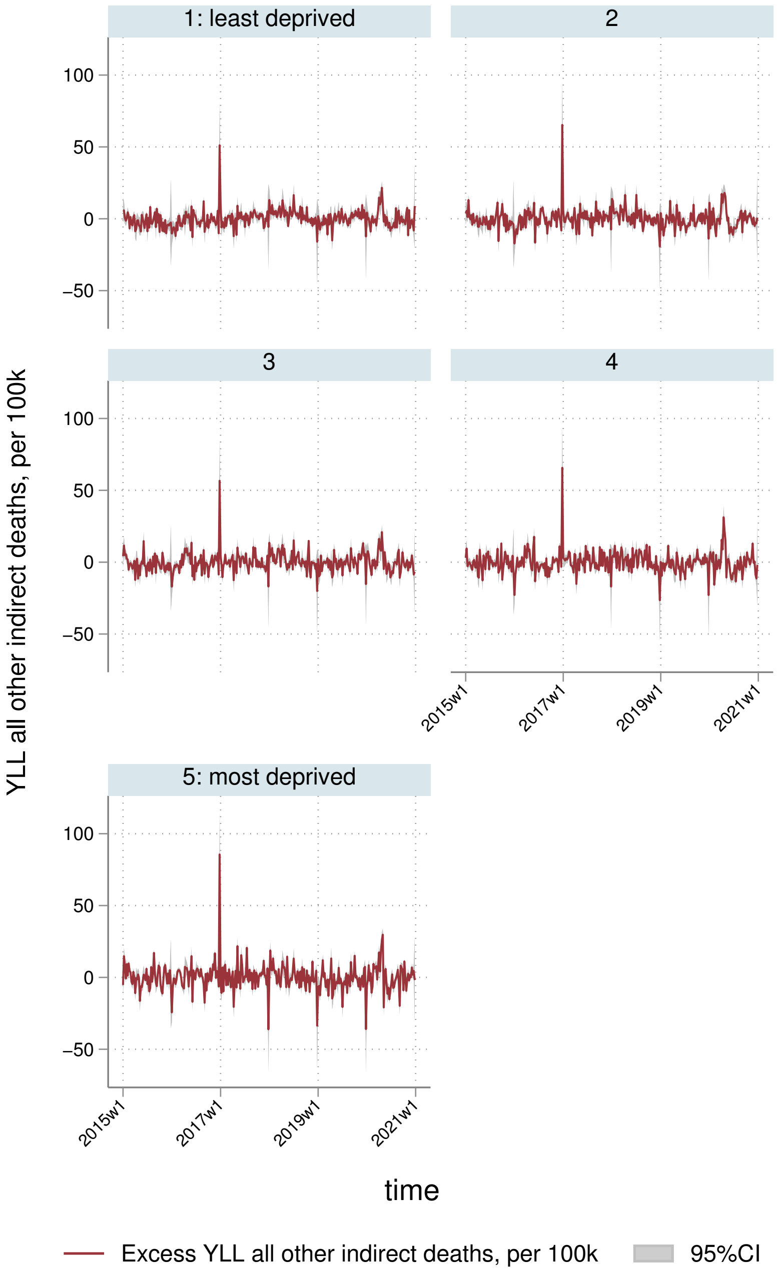

Figure 286: Excess Years of Life Lost trend per 100,000 population, all other indirect deaths by deprivation quintile, 2019-2020

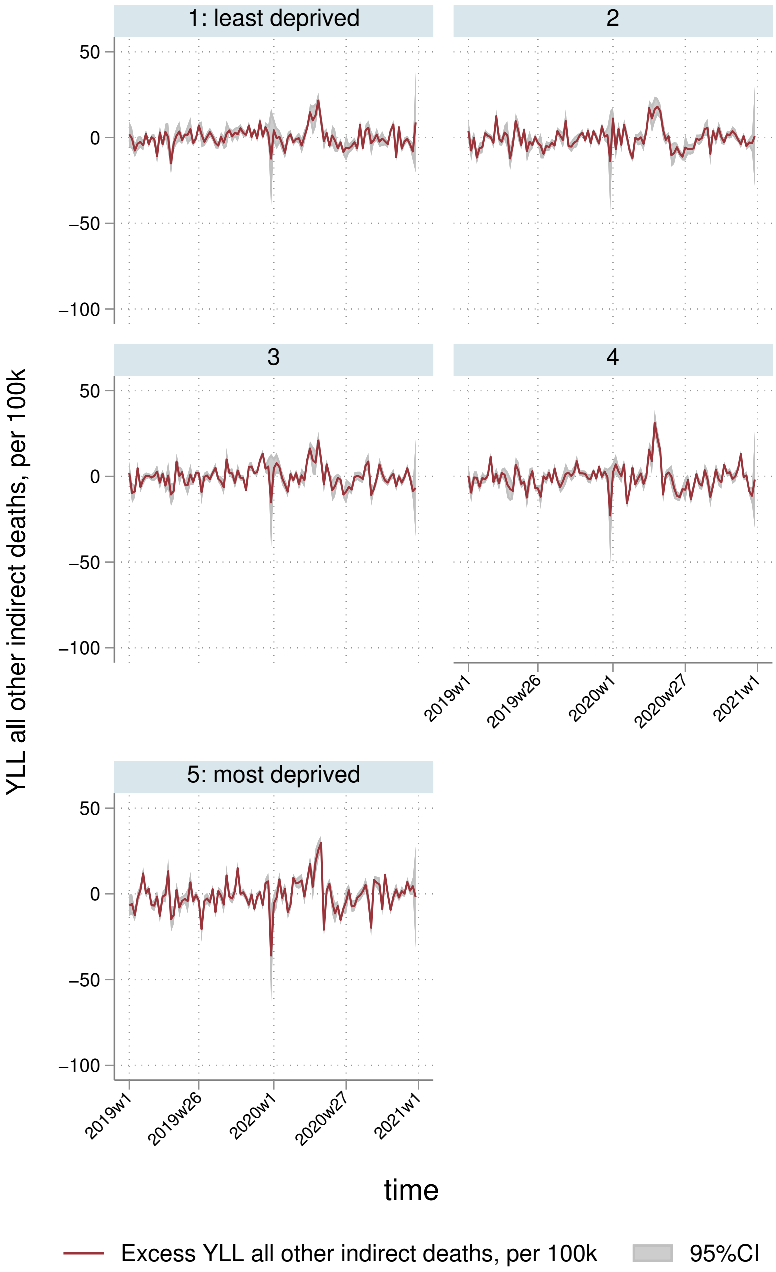

Figure 287: Observed vs Predicted Years of Life Lost trends per 100,000 population, all other indirect deaths by deprivation quintile, 2015-2020

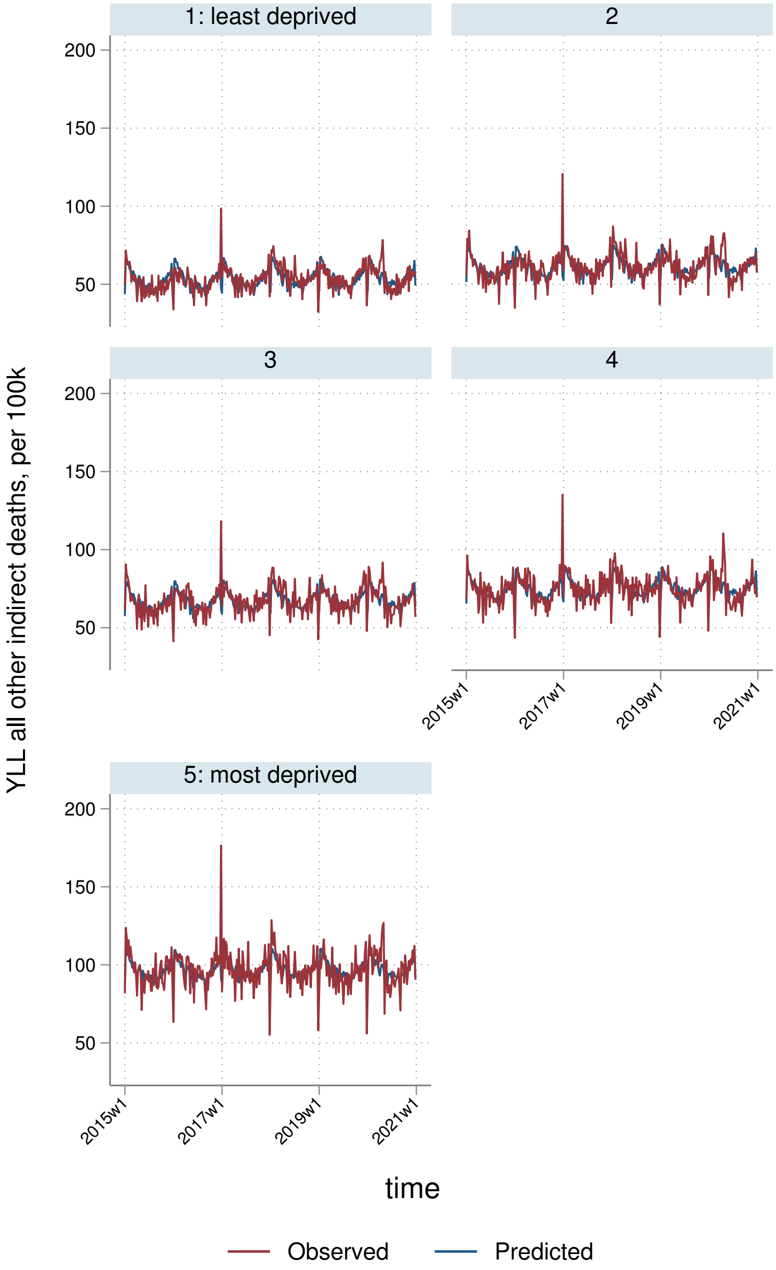

Figure 288: Observed vs Predicted Years of Life Lost trends per 100, 000 population, all other indirect deaths by deprivation quintile, 2019-2020

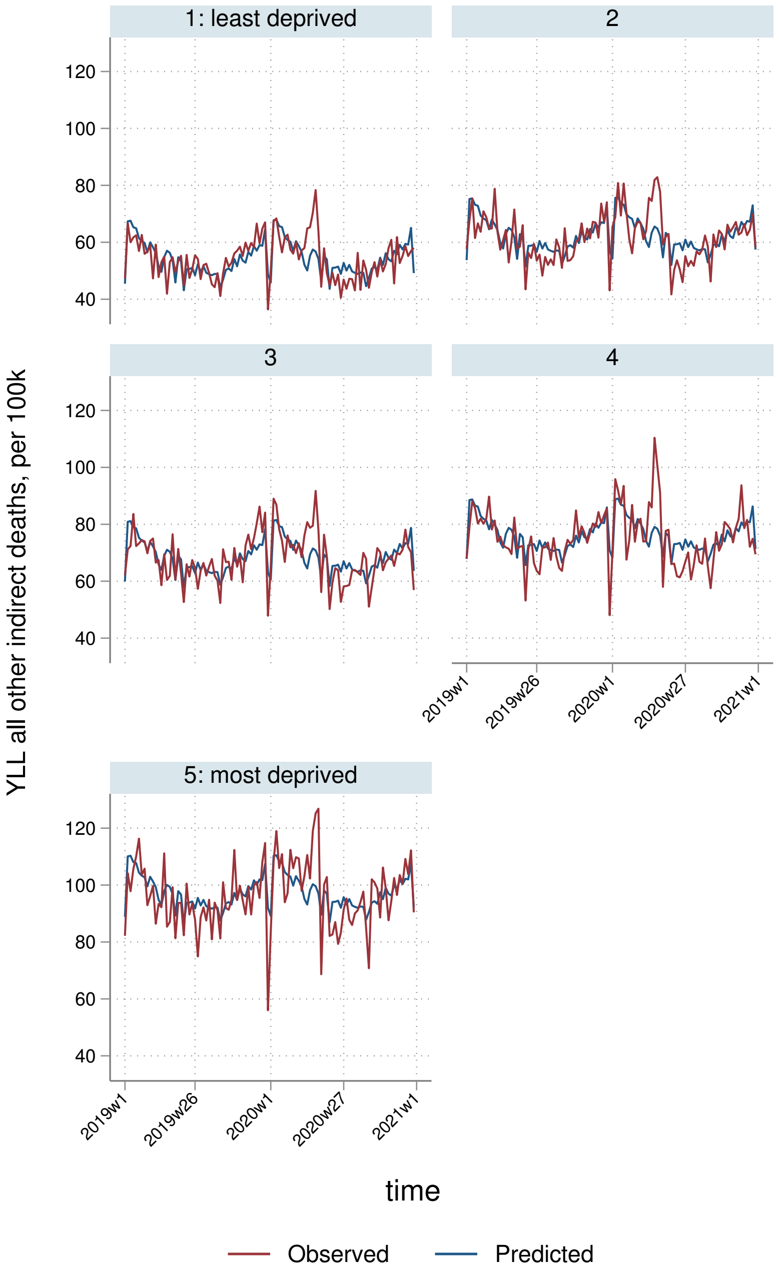

9.3.4 By Strategic Health Authority

Figure 289: Years of Life Lost trend per 100, 000 population, all other indirect deaths by region, 2015-2020

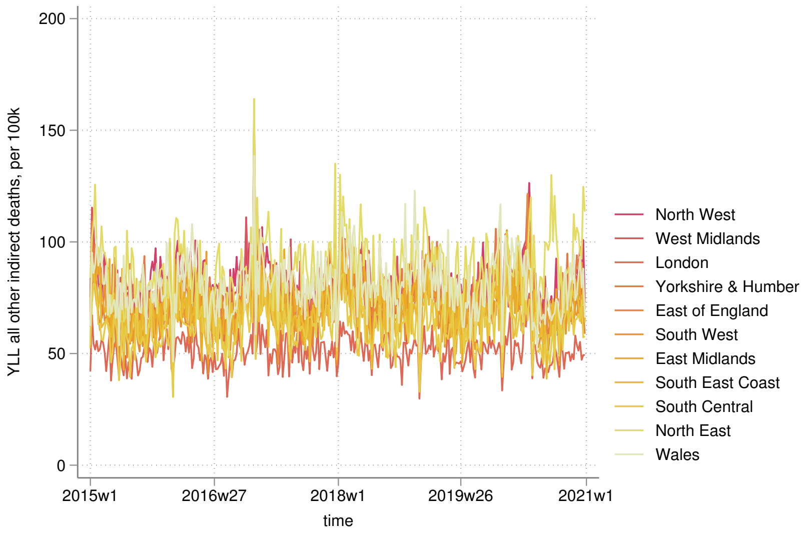

Figure 290: Years of Life Lost trend per 100, 000 population, all other indirect deaths by region, 2019-2020

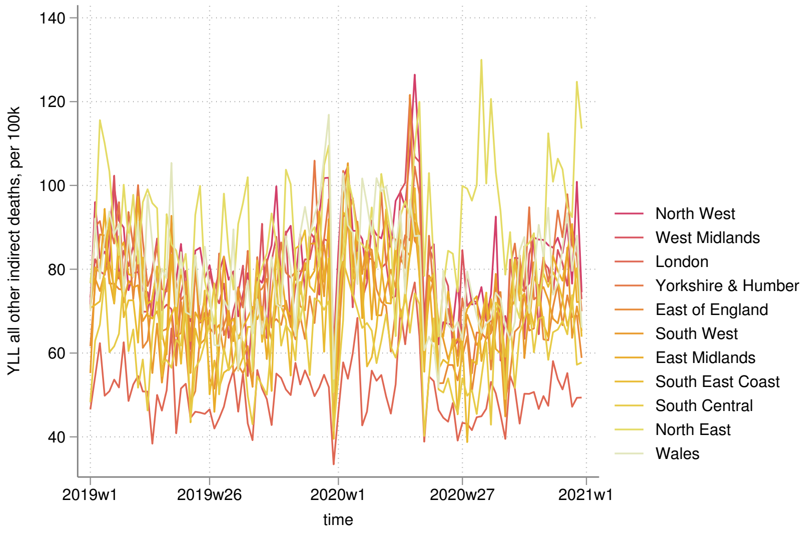

Figure 291: Excess Years of Life Lost trend per 100,000 population, all other indirect deaths by region, 2015-2020

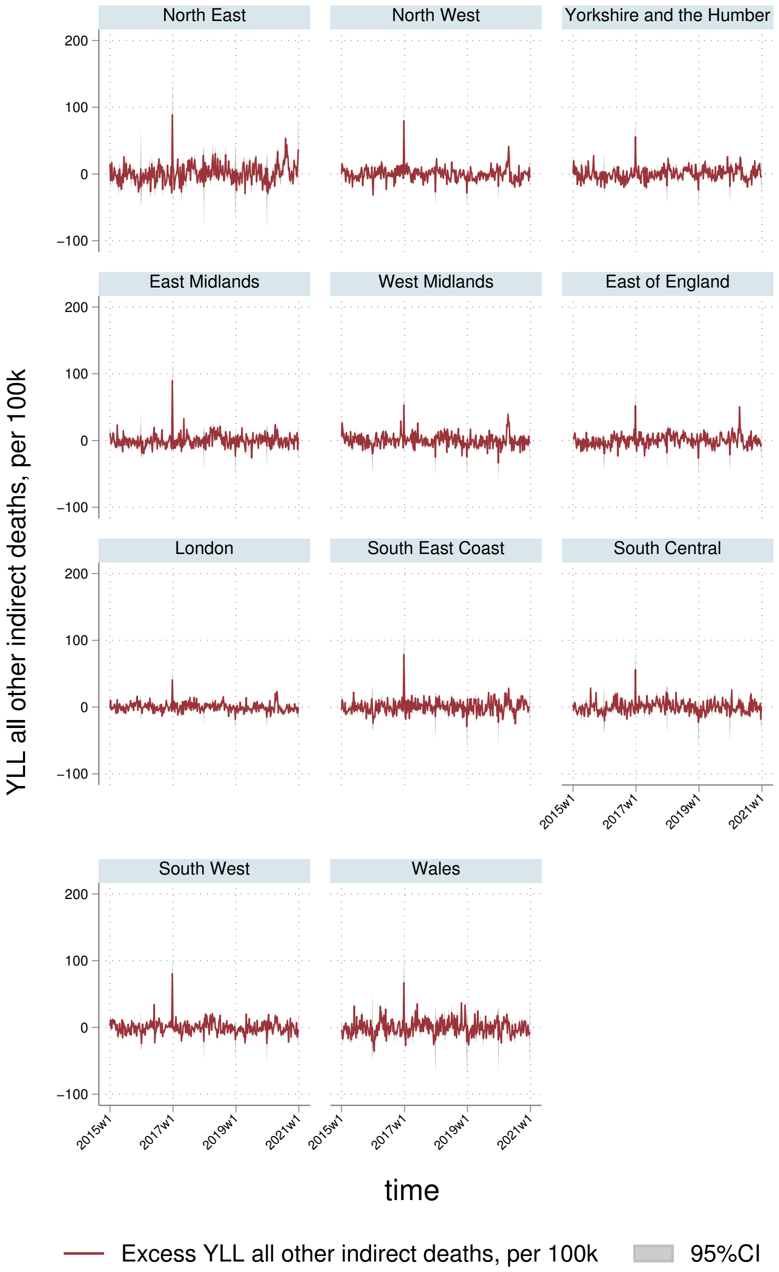

Figure 292: Excess Years of Life Lost trend per 100,000 population, all other indirect deaths by region, 2019-2020

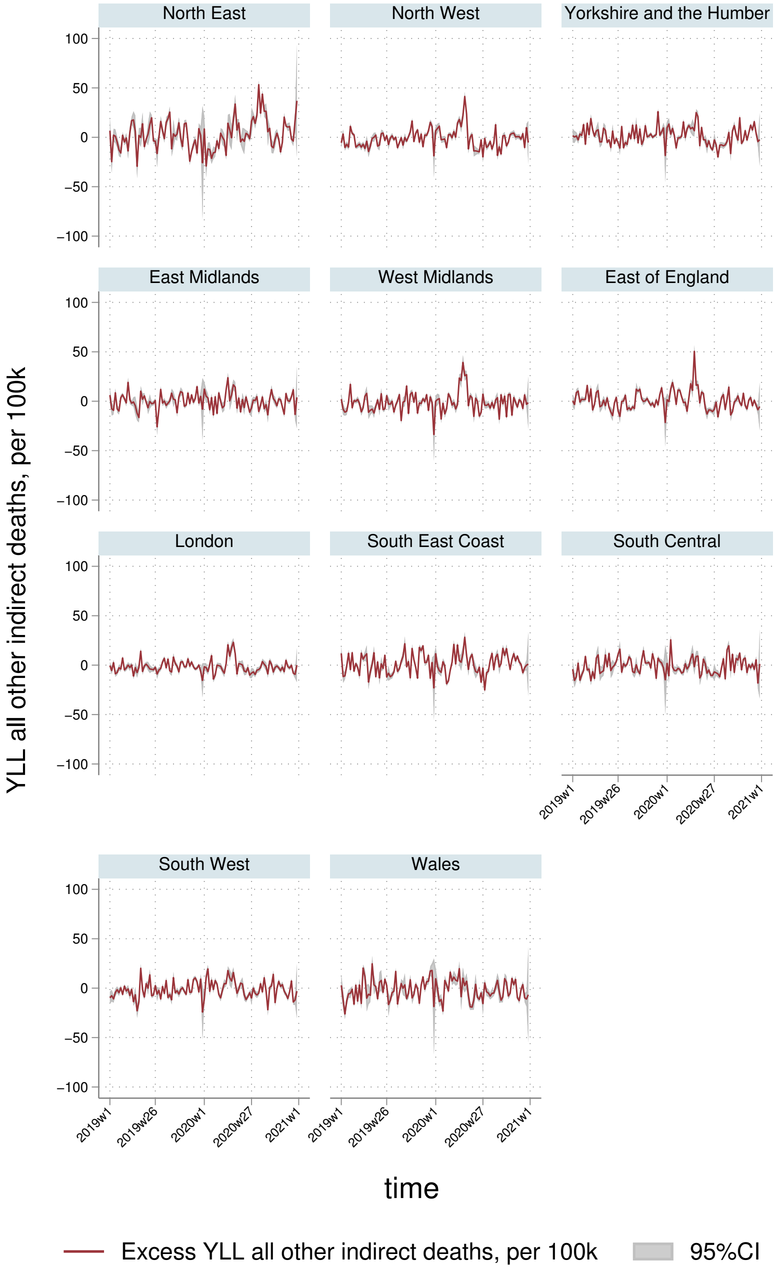

Figure 293: Observed vs Predicted Years of Life Lost trends per 100,000 population, all other indirect deaths by region, 2015-2020

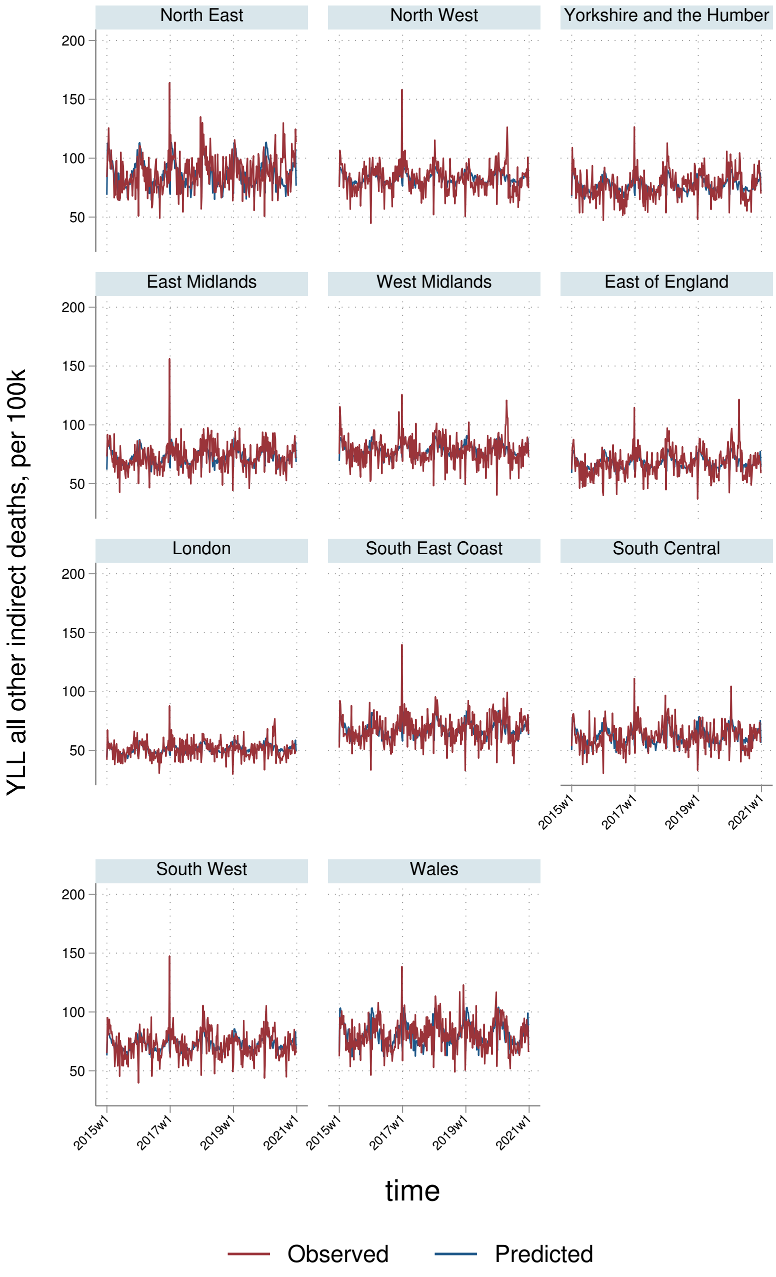

Figure 294: Observed vs Predicted Years of Life Lost trends per 100, 000 population, all other indirect deaths by region, 2019-2020

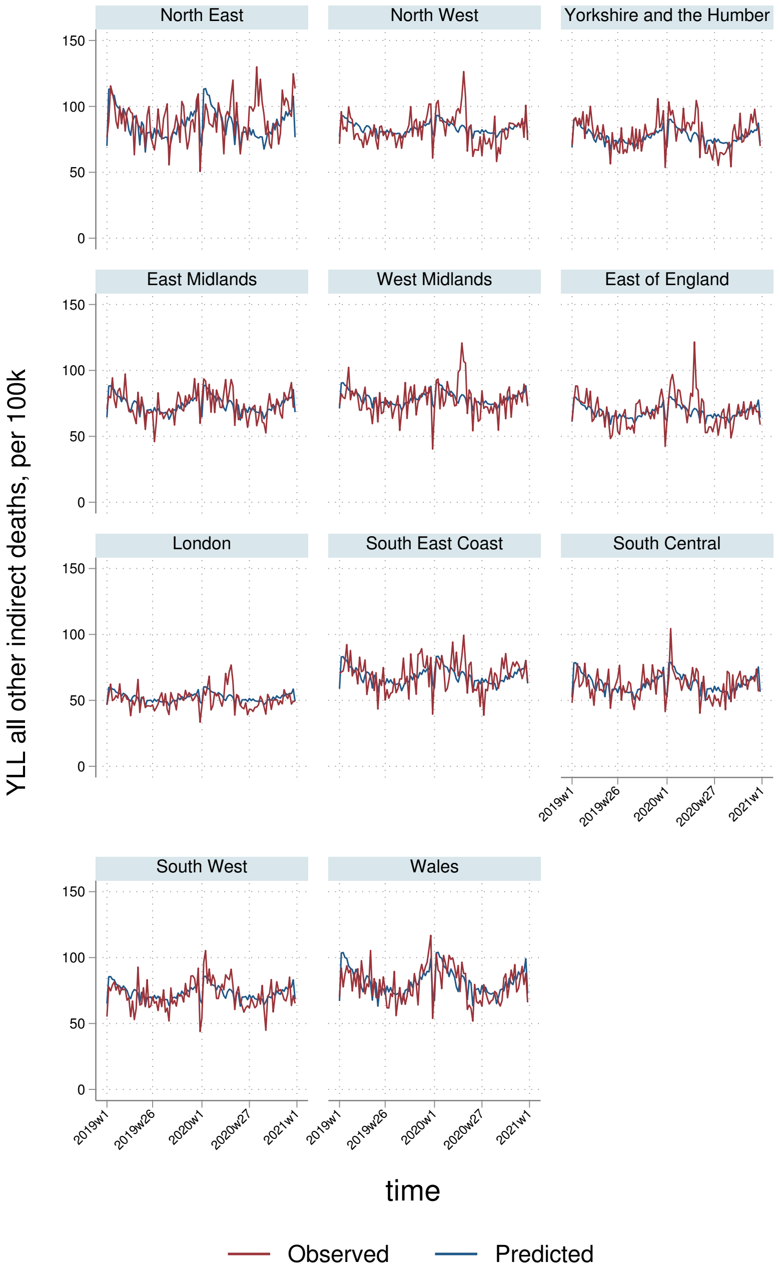

Supplement: S1 Appendix — (PDF) [file pmed.1003904.s002.pdf]
