# Supplementary material for: Excess years of life lost to COVID-19 and other causes of death by sex, neighbourhood deprivation, and region in England and Wales during 2020: A registry-based study
Source: PLoS Med. 2022 Feb 15;19(2):e1003904. doi: 10.1371/journal.pmed.1003904 (PMC8846534; doi:10.1371/journal.pmed.1003904)
Supplement: Attachment — Submitted filename: response.docx [file pmed.1003904.s017.docx]

We would like to thank the reviewer and the editor for their feedback. Please see point by point responses to all comments received. Resulting edits on the paper are highlighted to facilitate the review process.

**Requests from the editors:**

*General comments:*

*Please provide line numbers in your revised manuscript, ideally not beginning from 1 with each new page.*

*Throughout the paper, please adapt reference call-outs to the following style: "... decisions on seeking care [5,6]." (noting the absence of spaces within the square brackets).*

Response: Done

*Data availability:*

*PLOS Medicine requires that the de-identified data underlying the specific results in a published article be made available, without restrictions on access, in a public repository or as Supporting Information at the time of article publication, provided it is legal and ethical to do so.*

*Since some of the data are not freely available, please describe briefly the ethical, legal, or contractual restriction that prevents you from sharing it. Please also include an appropriate contact (web or email address) for inquiries - please note that a study author cannot be the contact person for the data.*

Response: Thank you. We have edited the data availability statement. Unfortunately, we are unable to share data in line with our data sharing agreements with NHS Digital due to low cell counts for some of the subgroups, which could identify some of the deaths.

*Title: Please revise your title according to PLOS Medicine's style. Please place the study design in the subtitle (ie, after a colon). We suggest “Excess years of life lost to COVID-19 and other causes of death by sex, neighbourhood deprivation and region in England & Wales during 2020: A registry-based study” or similar*

Response: Thank you, we have amended the title accordingly.

*Abstract:*

*Please structure your abstract using the PLOS Medicine headings (Background, Methods and Findings, Conclusions), combining Methods and Findings sections into one section.*

Response: Thank you, we have amended the section accordingly.

*Abstract Background: Please provide the context of why the study is important. The final sentence should clearly state the study question.*

Response: Thank you, we have now expanded the abstract both in terms of the background but also shortly expanded the methods and findings section.

*Abstract Methods and Findings:*

*Please provide brief demographic details of the study population (e.g. sex, age, ethnicity, etc) Please include the study design, number of individuals included, and main outcome measures.*

*Please comment on other findings from the study relating to age and region In the last sentence of the Abstract Methods and Findings section, please describe 2-3 of the main limitations of the study's methodology.*

Response: Thank you, we have expanded the abstract as much as possible to cover most of these. In terms of the demographics, we do not have that information for the whole population (only through predictions from the ONS, using the decennial censuses), we only have data on the deaths, and although some aspects of these estimates can be provided (in terms of age and sex, for example, but not ethnicity), they are not that relevant to the study we feel, and we opted to expand on other aspects of the aspects, especially considering the word limit regarding the abstract. We have added the number of deaths observed and clarified the main outcome measure (YLL). We are now discussing regional variability in the results, as well as age groups (rather than YLLs only). The study has numerous limitations which are clearly described in the relevant section in the discussion – we have now selected two and we have included them in the methods section of the abstract. The type of the study (mortality registers) and the design (interrupted time series) are also clearly mentioned now.

*Abstract Conclusions:*

*Please begin your Abstract Conclusions with "In this study, we observed ..." or similar, to summarize the main findings from your study, without overstating your conclusions. Please emphasize what is new and address the implications of your study, being careful to avoid assertions of primacy.*

Response: Thank you, done.

*Author Summary:*

*At this stage, we ask that you include a short, non-technical Author Summary of your research to make findings accessible to a wide audience that includes both scientists and non-scientists. The Author Summary should immediately follow the Abstract in your revised manuscript. This text is subject to editorial change and should be distinct from the scientific abstract. Please see our author guidelines for more information:* [*https://journals.plos.org/plosmedicine/s/revising-your-manuscript#loc-author-summary*](https://journals.plos.org/plosmedicine/s/revising-your-manuscript#loc-author-summary)

*In the final bullet point of ‘What Do These Findings Mean?’, please describe the main limitations of the study in non-technical language.*

Response: Thank you, we have included that section as requested.

*Introduction:*

*Please indicate whether your study is novel, being careful to temper assertions of primacy by adding ‘to the best of our knowledge’ or similar.*

*The terms gender and sex are not interchangeable (as discussed in* [*http://www.who.int/gender/whatisgender/en/*](http://www.who.int/gender/whatisgender/en/) *); please use the appropriate term.*

*Please conclude the Introduction with a clear description of the study question or hypothesis.*

Response: Thank you, one instance of “gender” was changed to “sex”. We have edited the introduction to explain our underlying hypothesis and highlight the novelty of the work.

*Methods:*

*Did your study have a prospective protocol or analysis plan? Please state this (either way) early in the Methods section. If a prospective analysis plan (from your funding proposal, IRB or other ethics committee submission, study protocol, or other planning document written before analyzing the data) was used in designing the study, please include the relevant prospectively written document with your revised manuscript as a Supporting Information file to be published alongside your study, and cite it in the Methods section. A legend for this file should be included at the end of your manuscript. If no such document exists, please make sure that the Methods section transparently describes when analyses were planned, and if/when reported analyses differed from those that were planned. Changes in the analysis-- including those made in response to peer review comments-- should be identified as such in the Methods section of the paper, with rationale. If a reported analysis was performed based on an interesting but unanticipated pattern in the data, please be clear that the analysis was data-driven.*

Response: Thank you. There was no funding or protocol. We feel the methods section is clear and allows replicability. There were no changes to the analyses informed by patterns in the data. We had an underlying hypothesis, which has now been added at the end of the introduction, and the analysis was not data-driven.

*Ethics statement: Please state in the methods why your study did not require an ethics statement.*

Response: Thank you, a sentence in relation to this has been added a the end of the methods section.

*Please ensure that the study is reported according to the RECORD guideline, and include the completed RECORD checklist as Supporting Information. Please add the following statement, or similar, to the Methods: "This study is reported as per the REporting of studies Conducted using Observational Routinely-collected Data (RECORD) guideline (S1 Checklist)." The RECORD guideline can be found here:* [*https://www.record-statement.org/*](https://www.record-statement.org/) *When completing the checklist, please use section and paragraph numbers, rather than page numbers which will likely no longer correspond to the appropriate sections after copy-editing.*

Response: Thank you, we are using the STROBE checklist for cohort studies since the RECORD statement is an extension of STROBE for studies using routinely-collected health data (such as health administrative data, electronic medical record data, primary care surveillance data, and disease registries) – and we do not use such data.

*“More details on the selected groups have been provided elsewhere” - please provide more details in the supplementary materials There is an unusually high degree of matching between your methods section and that of reference 2; please address this in your revision via rephrasing throughout the relevant section*

Response: Thank you, we have now expanded the methods section and no longer refer to the previous paper as a methods supplement. The high level of similarity is not due to the analysis plan, which has considerable differences, but it is due to the detailed description of the data environment, the data and the death groupings. We have now made some changes to try to address this, but we hope the editor appreciates that it is somewhat counter-productive, and that any level of similarity in this context does not constitute plagiarism.

*Results:*

*Please provide a table showing the baseline characteristics of the study population (Table 1).*

Response: Thank you, this relates to a similar point previously raised in relation to the abstract. This is not a typical case-control or cohort study so there are no baseline characteristics. We could provide age and sex for deaths over time, but we do not think that adds a value to the manuscript of help answering the research question in any way. Instead, we have reported the denominators for YLL in the appendix.

*Please report the number of individuals included in your analysis.*

Response: Thank you, we are now starting the results section with the number of observed deaths.

*Please refer to the relevant figures and tables to support the findings presented in the main text.*

Response: Thank you, all presented figures and tables are referenced in-text. Due to the large volume of outputs in the appendix, we do not reference every single one in the main paper, but we reference the appendix as a whole. We feel this is adequate since the appendix is organised in sections that correspond to the structure of the main paper to facilitate access, and all outputs are appropriately labelled.

*Discussion:*

*Please present and organize the Discussion as follows: a short, clear summary of the article's findings; what the study adds to existing research and where and why the results may differ from previous research; strengths and limitations of the study; implications and next steps for research, clinical practice, and/or public policy; one-paragraph conclusion.*

*Please remove all subheadings within your Discussion*

Response: Thank you, this was the structure we previously followed, and we have now removed all subheadings.

*Figures:*

*Please provide titles and legends for each individual figure*

Response: We can confirm that titles and legends were included in all figures. What the editor probably means is how the legends only appear at the bottom of the multi-panel figures. This is intended since the x-axes are the same across all panels. We would prefer not to change that aspect, since we think it is visually better, but are happy to make the changes if they are considered essential.

*Tables:*

*Please provide titles and legends for each individual table Tables 1, 2 Please clarify what the numbers in brackets represent in the respective table legends*

Response: We are not sure what the editors means by “legends” in the context of tables. Both tables 1 and 2 have titles. We have clarified that the 95% confidence intervals for the estimates are provided in brackets.

*References:*

*Please ensure that journal name abbreviations match those found in the National Center for Biotechnology Information (NCBI) databases, and are appropriately formatted and capitalised.*

*Please also see* [*https://journals.plos.org/plosmedicine/s/submission-guidelines#loc-references*](https://journals.plos.org/plosmedicine/s/submission-guidelines#loc-references) *for further details on reference formatting.*

*Where website addresses are cited, please specify the date of access.*

Response: We have provided access data information and have removed abbreviations (except for BMC Medicine). We have also corrected an instance where the journal name was not capitalised.

*Supplementary files:*

*Please provide titles and legends for each individual table and figure in the Supporting Information.*

*Please see* [*https://journals.plos.org/plosmedicine/s/supporting-information*](https://journals.plos.org/plosmedicine/s/supporting-information) *for our supporting information guidelines.*

Response: We have provided titles (legends were already there) for all figures in the supplementary file.

***Comments from the reviewers:***

***Reviewer #1****: The manuscript describes an interesting study, that is very relevant for both estimating the overall impact of COVID-19 on excess mortality in the UK, and more specifically its impact on socioeconomic and regional inequalities. The use of the years of life lost is fairly novel, and adds to the relevance of the work, as it makes it easier to interpret. Additionally, this takes into account the differential impact of COVID-19 by age groups. The paper is well written and generally provides enough information on the various steps taken in the analyses. However, I do have some issues that I would like to see addressed, detailed below.*

*Major issues*

*I find the classification of direct and indirect excess death somewhat problematic. Including non-COVID-19 specific respiratory deaths as direct COVID-19 deaths due to potential misclassification is understandable, and addressed in the limitations section. However, the inclusion of indirect deaths could use more elaboration, both in the introduction section (to justify including it), and in the methods section (how it is defined). That deaths due to cardiovascular disease and diabetes may be related to COVID-19 seems somewhat plausible. Although as far as I know, these are risk factors that make a person more susceptible to (severe outcomes) of COVID-19, which does not necessarily mean that dying from cardiovascular disease or diabetes is indirectly caused by COVID-19. How cancer deaths and all other deaths (including suicides and accidents) are counted as indirectly caused by COVID-19, I find harder to understand. Perhaps, it would make more sense to simply split the results into direct COVID-19 deaths, and all-cause deaths. Or use different terminology, as in the discussion, the authors both mention "indirect deaths, i.e. those not directly attributed to COVID-19", and causes both related and unrelated to COVID-19 infection. I personally think that unrelated seems to be more fitting for most of the categories that are currently defined as indirect.*

Response: Thank you for this comment. We have now expanded the introduction and methods sections to explain the reasoning behind the grouping (please see also our response to the editor in relation to the methods section). In short, we considered these underlying causes of death prevalent enough to be analysed separately and to investigate whether there was an excess for them during the pandemic. Although we would like to analyse suicides separately, for example, numbers are too low for this methodological approach (weekly time-series across strata). Thus, everything else, bar direct, cardiovascular & diabetes, and cancer, was aggregated into “other indirect” deaths. We start our reporting with all-cause deaths and continue with direct deaths, before analysing the remaining three groups, so to some extent we do already deliver what the reviewer requested. However, we also think that the three “indirect” groups add considerable value to the manuscript and the findings from these analyses will be of interest to most readers. We also accept the criticism regarding labelling them indirect, which may not be necessarily true, but we think it is preferable to unrelated. This is because we use an interrupted time series design to model excess deaths, during the pandemic, and the underlying assumption is that excess deaths during this period are attributed to the pandemic (<https://www.bmj.com/content/350/bmj.h2750>), for example due to changes in healthcare provision resulting delays in diagnosis / treatment of time sensitive conditions such as cancer. We feel this is a reasonable assumption, whatever the death category and whatever the underlying mechanism for any identified excess (e.g. limited access to secondary care or increased anxiety leading to alcohol/drug abuses or even suicide). The major limitation of an interrupted time series is an unidentified exogenous factor, being active in parallel with the one whose “effect” we are trying to quantify; however, we feel that the pandemic was such a high level and impactful event, that bias introduced by a secondary unrelated (not present because of the pandemic) event, e.g. a new intervention for elective cardiovascular management, is extremely unlikely to explain a substantial proportion of the observed changes.

*Minor issues*

*In the abstract and methods, the authors mention that data is obtained from the end of 2014 onwards, however they also mention that 2019 is used as the baseline period to which the 2020 data are compared. It is not clear to me whether data from 2014 to 2018 were used and if so, why? Please clarify which data period was used as the reference.*

Response: Thank you for raising this. The whole time series data were used to obtain estimates of excess deaths (observed minus predictions from all historical data), but we also use 2019 as a reference to quantify as a percentage increase (15%, in the abstract), from 2019 to 2020.

*In calculating the index of multiple deprivation, data were used for England from 2019, and for Wales from 2014. Does this not create some problem in comparing the two? The authors do state that these scores are relatively constant over time, would it then not make more sense to use data from 2014 for both countries?*

Response: This is a reasonable suggestion but there is never complete alignment in terms of times for when these measures are released in UK countries (NI, Scotland, England and Wales). The closest time point for the English IMD would be 2015. However, as the population of England is approximately 56m and the population of Wales is just over 3m, we feel the most conservative approach is to use the latest available IMD scores, even if they do not change much over time.

*In presenting the results, the authors first describe the years of life lost in absolute numbers, and then again, standardized per 100,000 people. The former might be relevant for policymakers, or people very familiar with the total number of people living in the various described regions. However, for the readership of a scientific journal such as this, it seems somewhat redundant, and the numbers per 100,000 are much easier to interpret and compare.*

Response: Thank you, we agree and that is why we have focused on the latter in the abstract. However, as the reviewer has highlighted, there is still some value in the absolute numbers, for policy makers and local authorities. Thus, we would prefer to keep them for completeness.

*In the results section, heading Respiratory diseases, the authors mention the number of YLL and state that this is 'an 123% increase … compared to the equivalent time period in 2019'. This comparison sound problematic to me, as there were 0 COVID-19 related deaths in 2019. If this is comparing all respiratory deaths to those of 2019, this should be specified more clearly.*

Response: Thank you for prompting us to clarify this. We have now made this clearer in the respective section in the results. In the methods section we previously said that we modelled COVID-19 and respiratory deaths together and also explain in the limitations section that this is a necessity to some extent, to allow us to model excess deaths for this group.

*In the sentence: "Excess respiratory deaths across age-groups, … in younger age groups (45-64: 2008/681; 65-74: 2774/1377; 75-84: 5069/3655; 85+: 5302/5636).", these ratios are not intuitive to interpret. It would be better to write these out as in done in the last paragraph of the results section, e.g. "ratio increased to 1.64 (1,501,323/913,418)."*

Response: Thank you for this suggestion – we have changed the results accordingly.

*"Assuming estimated excess respiratory YLL were only distributed amongst estimated excess respiratory deaths,…". It is unclear what is meant in this sentence, please reformulate it to improve clarity.*

Response: We appreciate the original wording was not ideal when what we were trying to express was a complicated notion. Basically, we obtained both a respiratory YLL estimate and an estimate of respiratory excess deaths. We can only calculate YLL per death under certain assumptions, mainly based on the number of deaths. Excess YLLs may not be solely attributable to more deaths – it could have been the case, for example, that people were dying younger but not more often (which is not the case here). Although we do see both excess deaths and excess YLLs, that does not mean that all YLLs are amongst the excess deaths – but that is an assumption we have to make if we are to quantify excess YLLs per death. And we also assume the number of excess deaths is an observation, rather than an estimate (we necessarily ignore the confidence intervals around the excess deaths estimate). Rather that complicate the results section, we have tried to clarify the issue in the limitations section.

*In the discussion, the authors mention that "in addition to excluding COVID-19 deaths from the total excess deaths, we also excluded other respiratory causes of death (the most likely source of 'missed' COVID-19 diagnoses)." Firstly, I think this should be mentioned under the methods section, and not only in the discussion section. Secondly, I understand that both COVID-19 deaths and respiratory deaths might be liable to 'missed' diagnoses, but in my opinion, that should not matter for reporting total excess deaths. When one read total excess deaths, one assumes this includes all causes, regardless of whether they were correctly classified or not.*

Response: Apologies, the wording for the statement was inappropriate and was conveying the wrong message. We were trying to say that when it came to all indirect deaths (not the total number of deaths), we did not include respiratory deaths. We have now edited the text for accuracy in the limitations section and we do not think anything needs to be added to the methods section.

*The authors state: "We did not control for population trends, assuming small increases over time …". Referring back to my point about which time period was used as comparison, if only 2019 data was used as baseline, then I agree that controlling for population trends is probably not necessary. However, if data from 2014 onwards was used, I would find this assumption more problematic, and would want to see some description of existing trends as justification for (not) controlling for them.*

Response: Thank you, as previously mentioned we did use data from 2014 in our models. The main reason we did not control for population sizes was technical: obtaining weekly estimates of excess YLLs and their SEs is straightforward, but not as an aggregate (over the whole 2020 period of interest). To be able to obtain the aggregate estimate, we could not control for population size (we would obtain an estimate for the NE, for example, set to the mean population of all regions). However, we did control for population in our previous analyses and we found that it had no substantive impact, and population sizes between census years are estimates in any case, which are retrospectively adjusted with each census (in this case the 2021 census). We have expanded that sentence in the limitations section to clarify our modelling choices.

*Tables 1 and 2 in their current form are very hard to read. Adding thousands separators would help, Moreover, there is no table legend to clarify which numbers are presented, and which are in parentheses. Also, referring back to my earlier comment, I think mostly table 2 with the results per 100,000 are useful for a broad audience.*

Response: Thank you, we appreciate there is a lot of information presented in the tables. As suggested, adding a thousands separator may help but then we would have to change the separator in the confidence intervals, reduce the size of the font or break down into multiple tables. So for that reason, we would prefer not to add a thousand separator at this stage, although that is something that could be addressed in the production stage, if the paper is accepted. Regarding the table legend, we do state that we present “Estimated excess years of life lost *(95% confidence intervals)*, of direct, indirect and total excess deaths, weeks 11 to 52, 7 Mar 2020 to 25 Dec 2020” while there are column headers with the death grouping (COVID or other respiratory, etc). Perhaps the title ended up in a different paper during the conversion to pdf during submission. We feel this is adequate but please let us know if you have something else in mind. Regarding the numbers in parentheses, apologies for that omission, we have now expanded the title to clarify that these are the 95% confidence intervals. As for the use of Table 1, we still think it adds value and we would prefer to present both sets of results as previously mentioned.

***Reviewer #2:*** *"Excess years of life lost to COVID-19 and other causes of death by sex, neighbourhood deprivation and region in England & Wales during 2020" focuses on the (excess) years of life lost (YLL) metric, for assessing mortality due to the coronavirus either directly or indirectly, in England and Wales across various sex, geographical region and deprivation demographics. For the period approximately between March and December 2020, a 15% excess YLL was found, with relatively more excess YLL amongst the more-deprived, and also for younger age groups across deprivation quintiles. Analyses were performed for all-cause, respiratory disease-based, cardiovascular disease/diabetes-based and cancer/other indirect deaths. It should be noted that deprivation was estimated based on residence by postcode, and not from actual individual information.*

*The results are largely devoted to describing observed trends by group category, in detail. The paper appears of potential significance in quantifying the burden of COVID-19 on relatively-deprived neighbourhoods/regions. However, a number of issues might be addressed:*

*1. It might be considered to emphasize the additional contributions of this manuscript over prior related work by much the same authors (cited as [2]), in the Introduction*

Response: Thank you, we have now expanded the introduction and methods to discuss our previous work in more detail and explain what this work adds, while also clarifying the underlying cause of death groupings and their relevance to the pandemic, as requested by reviewer #1.

*2. It is stated that YLLs are calculated against 2019 life expectancy (79.4 years for males, 83.1 years for females), and that "Any death over those age thresholds, for males and females respectively does not contribute to the YLL total for the particular stratum" (Appendix Section 1). While this appears a possible definition for YLL, a perhaps more appropriate definition for YLL would involve the difference between age at death, and the standard life expectancy (SLE) at that age (see for example "Reflection on modern methods: years of life lost due to premature mortality—a versatile and comprehensive measure for monitoring non-communicable disease mortality", Martinez et al., International Journal of Epidemiology 48:4). For example, if a male aged 80 years perishes, then YLL would be (SLE at 80)-80, instead of zero as appears to currently be the case. This would seem more accurate since the (possibly-mitigable) death of subjects above the (average) life expectancy should intuitively result in some appreciable YLL instead of having no impact as currently defined, and would appear to underplay the impact on deaths amongst the elderly, who have been widely recognized as being the age group most vulnerable to the coronavirus.*

Response: Thank you, for bringing this to our attention. The reviewer is quite right and that statement in the appendix reflected an earlier version of the manuscript, where we did not use life tables. Although the main paper was amended when we updated our methods, prior to the original submission, we overlooked updating that statement in the appendix. In the methods section we say that *“For each death, potential years of life lost (YLL) were calculated using 2019 single year sex-specific life tables for England & Wales”*, in agreement with that the reviewer’s position. We have now corrected that statement in the appendix.

*If the above is correct, it might therefore be considered to perform some sensitivity analyses taking SLE into consideration, and also to clarify the statement that "Those aged over 100 were assumed to have the life expectancy of centenarians of the same sex", since it is not immediately clear whether they contribute to the YLL total in the first place, from the Appendix definition.*

Response: As previously mentioned this was not the case and the statement has now been corrected. We hope this clarifies the issue with the few individuals aged over 100: since life tables stop at 100, we assumed they had the same life expectancies as those aged 100 of the same sex, rather than assume zero YLLs.

*3. Conventions for ICD-10 coding might be briefly clarified. Might it be possible to have a death attributed to both direct and indirect causes (relative to COVID-19)? If so, how would they be accounted for, e.g. direct cause taking precedence, or under both causes?*

Response: This is a valid point and our approach was not clear in the original submission – we have now expanded the methods section to make it clear. In short, we made sure that categories were mutually exclusive, otherwise we were at risk of double-counting and potentially providing biased estimates.

*4. "Maximum lag order" might be briefly clarified, and the significance of the chosen value (52 weeks) explained.*

Response: Thank you, we have tried to make this clearer by stating this related to the autocorrelation expected in the time series, to be accounted in the calculation of the standard errors for the estimates of interest.

*5. The correspondence between quintile number and deprivation order might be stated, at the first mention of quintile number.*

Response: This is a good suggestion and we have added that information in the first paragraph of the “Analyses” section.

*6. It might be interesting to briefly note any interaction effects between the major categories, e.g. for generally more-deprived regions, is the increased YLL ratio effect for more-deprived quintiles amplified, constant or attenuated?*

Response: The reviewer is quite right, and this is another valuable suggestion. We had analysed the interaction between SHA and deprivation quintile but because the manuscript is already data-heavy and the appendix is in excess of 200 pages we had decided against presenting these results. It is a complicated picture because of varying baselines by region, across the same deprivation strata. See below the observed vs predicted for all-cause and direct deaths in the two graphs that follow (with North East 1, for example, being the North East areas in the most affluent quintile, nationally). There is great variability, with the most deprived areas in the North West and West Midlands, followed by the Yorkshire & Humber and the North East reporting the highest excess YLLs. There is a strong socio-economic association for London as well, for deprivation quintiles 3 to 5. Numbers for the other groupings (cardiovascular & diabetes, cancer and other indirect) are arguably too small in the interaction strata to derive reliable conclusions from. We have now added a couple of sentences in the methods section to explain we performed this analysis, and we briefly present these patterns, for all-cause and direct deaths. We also now include these figures in the appendix.

*7. It might be considered to provide an example of the projected YLL vs. observed annual YLL for past registered deaths (i.e. for each year from 2015 onwards) on a single chart, towards illustrating/explaining expected YLL. Moreover, if possible, if might be considered to demonstrate the validity of this procedure, by also displaying the expected YLL for say 2019, as estimated by data from 2015-2018.*

Response: Thank you for these suggestions. We are not sure what the table of observed vs predicted table would add, that the graphs do not deliver already. This is the reason we report two sets of graphs, 2015 to 2020 (for people to see the whole time series and how well the prediction seems to be working, albeit in used data) and 2019 to 2020 (to be able to follow the whole of 2020). All these figures are in the appendix (for the graphs in the main paper, we truncate the time series from 2019, so that changes in 2020 can be visible). Thus, we hope the reviewer agrees that additional tables to aggregate these estimates vs observed are not really needed. Regarding the other suggestion we agree that it is important to validate the model, and we had already done that to confirm it is suitable, but we did not report that in the main paper (we do now). Below, please see some of the predictions for 2019, using data from 2015 to 2018, for direct deaths and all-cause deaths.

*8. In general, it would be strongly suggested to annotate/caption Appendix charts, on the same page as the chart itself. For example, under Section 5.3.4, groups of charts with the same axes (time/YLL grand total, per 100k) and regions (North East, North West, etc.) are displayed on consecutive pages (Page 47 to 50) without further indication as to what they represent. This has made evaluation of the Appendix data challenging.*

Response: We apologise for this. It was a challenge to produce the appendix and we took some shortcuts because of its size. We have now annotated all the graphs in the appendix, and thank you for your time going through it.

Reviewer #3: This research shows the behavior of excess YLL in England & Wales and how it was affected by socioeconomic conditions reflecting important inequities exacerbated of the SARS-CoV-2 pandemic. The article is well written and methodologically correct. Despite this, I wish to make a few recommendations that may be helpful to readers, particularly non-England & Wales readers. In the first place, it would be interesting to mention in the discussion what is the percentage of underreporting of mortality in England & Wales and to what extent this could affect the estimates of excess YLL. Second, that comparisons are made within England & Wales, it would be important to include in the discussion comparisons of the results with other European countries, in order to understand if the impact of the pandemic on the excess of YLL in England & Wales was higher or lower than other countries. A map showing the regions of England & Wales selected for analysis would be appreciated.

Response: Thank you for raising these points. As far as we know there is underreporting of mortality in England and Wales, and there may be a delay in cases where there is a coroner’s verdict pending, as discussed in the limitations section. So, in terms of all-cause deaths and direct deaths (where such a verdict is not needed), we are confident there are no lags in coding and thus death records are complete (assuming a 6-week lag in the data coming through, which we have accounted for). For the causes of death that may need a coroner’s verdict we have grouped them in a single group, including temporary ICD-10 codes (all other indirect deaths) and thus “movement” within that category does not affect our models. To summarise we are confident we are capturing all deaths in the time period of interest. In terms of the national comparison, the reviewer is quite right, and we have addressed that omission by including a sentence in the introduction that discusses excess deaths in other countries (USA, Germany, Italy and Brazil.).

Finally, unfortunately, we have very high-level regions (10 in England plus Wales) so we feel a spatial map will add very little at that level, and we feel the graphs we present currently can be more informative (allowing us to present variability in the estimates, for example, when that is not possible with spatial maps).
