# Supplementary material for: Excess years of life lost to COVID-19 and other causes of death by sex, neighbourhood deprivation, and region in England and Wales during 2020: A registry-based study
Source: PLoS Med. 2022 Feb 15;19(2):e1003904. doi: 10.1371/journal.pmed.1003904 (PMC8846534; doi:10.1371/journal.pmed.1003904)
Supplement: Attachment — Submitted filename: response.docx [file pmed.1003904.s018.docx]

Thank you for accepting our paper for publication. Below we address the outstanding editorial points:

*1. Data availability: PLOS Medicine requires a “minimal data set” which consists of the data set used to reach the conclusions drawn in the manuscript with related metadata and methods, and any additional data required to replicate the reported study findings in their entirety. Authors do not need to submit their entire data set, or the raw data collected during an investigation. Please submit the following data: The values behind the means, standard deviations and other measures reported; The values used to build graphs; The points extracted from images for analysis.*

**Response:** We have now included output log html files, which include the models and estimates from these models which are used in the figures and tables.

*2. Please relocate the limitations in the Abstract to the end of the Methods and Findings section.*

**Response:** Done

*3. Please avoid use of double negatives such as “losses in … life lost” (line 110 in the Abstract).*

**Response:** Done

*4. In the conclusion (abstract and main text), please focus on reporting what you have observed and the implications, rather than trying to conclude that “inequalities … were exacerbated"*

**Response:** Thank you, we have toned down this in the abstract but we do feel that this is exactly what we found, with the more deprived populations, reporting the highest YLLs; thus, we are observing exacerbated inequalities.

*5. Please add the following statement, or similar, to the Methods: "**This study is reported as per the Strengthening the Reporting of Observational Studies in Epidemiology (STROBE) guideline (S1 Checklist)."*

**Response:** Thank you, added.
